# Supplementary material for: Asymmetric synthesis of tetrazole and dihydroisoquinoline derivatives by isocyanide-based multicomponent reactions
Source: Nat Commun. 2019 May 9;10:2116. doi: 10.1038/s41467-019-09904-5 (PMC6509109; doi:10.1038/s41467-019-09904-5)
Supplement: Supplementary file 1 — Supporting Information [file 41467_2019_9904_MOESM1_ESM.pdf]

Supplementary Information

Asymmetric synthesis of tetrazole and dihydroisoquinoline derivatives by isocyanide-based multicomponent reactions

Xiong *et al*.

Supplementary Method

General remarks

^{1}H NMR spectra were recorded on commercial instruments (400 MHz). Chemical shifts were recorded in ppm relative to tetramethylsilane and with the solvent resonance as the internal standard ($CDCl_3$, δ = 7.26). Data were reported as follows: chemical shift, multiplicity (s = singlet, d = doublet, t = triplet, q = quartet, m = multiplet), coupling constants (Hz), integration. $^{13}C\{^{1}H\}$ NMR data were collected on commercial instruments (100 MHz) with complete proton decoupling. Chemical shifts were reported in ppm from the tetramethylsilane with the solvent resonance as internal standard ($CDCl_3$, δ = 77.16). Enantiomeric excesses were determined by chiral HPLC analysis on Daicel Chiralcel ADH, IA, IB, IC, IE, and Phenomenex Chiralcel Lux 5u Cellulose-2 at 23 °C with UV detector in comparison with the authentic racemates. Optical rotations were determined after flash column chromatography purification and reported as follows: $[\alpha]_D^T$ (*c*: g/100 mL, in CH_2Cl_2). HRMS were recorded on a commercial apparatus (ESI source). All the reactions were carried out under an atmosphere of nitrogen in over-dried apparatus. All the solvents were purified by usual methods before use. Chromatography: Qingdao Haiyang silica gel, HG/T2354-92, H CP. Reagents purchased from commercial suppliers were used: 2-naphthyl isocyanide (Aldrich), 4-nitrophenyl isocyanide (TCI), 4-methoxyphenyl isocyanide (Aldrich), 2,3-dihydro-6-isocyano-1,4-benzodioxine (Acros), *tert*-butyl isocyanide, magnesium trifluoromethanesulfonate (Alfa), trimethylsilyl azide (TCI). The *N,N'*-dioxide ligands[1-2] and alkylidene malonates[3-4] were synthesized according to known procedures.

General procedure for the synthesis of racemic product

General procedure 1: To an oven-dried tube were added $Mg(OTf)_2$ (0.014 mmol, 14 mol%), 2-(cyclohexylmethylene)malonate **3a** (0.20 mmol), $TMSN_3$ **2a** (0.24 mmol), 2-naphthyl isocyanide **1a** (0.20 mmol) and CH_2ClCH_2Cl (1.0 mL). The mixure was stirred in CH_2ClCH_2Cl at 30 °C for 3 h and directly purified by flash chromatography

on silica gel (eluent: petroleum ether/ethyl acetate = 9/1) to afford the racemic product **5a**. (For 2-benzylidenemalonate **3o**, 0.01 mmol $NaAr^F{}_4$ was added).

General procedure 2: To an oven-dried tube under nitrogen atmosphere were added $Mg(OTf)_2$ (0.010 mmol, 10 mol%), racemic **RaPr$_2$** (0.010 mmol, 10 mol%), dimethyl 2-benzylidenemalonate **3o** (0.10 mmol), 5 μL H_2O, $TMSN_3$ **2a** (0.15 mmol) 2-naphthyl isocyanide **1a** (0.10 mmol) and CH_2Cl_2 (1.0 mL). The mixture was stirred at 30 °C for 48 h, and directly purified by flash chromatography on silica gel (eluent: petroleum ether/ethyl acetate = 9/1) to afford the racemic product **4o**.

General procedure 3: To an oven-dried tube under nitrogen atmosphere were added $Mg(OTf)_2$ (0.010 mmol, 10 mol%), racemic **PiPr$_2$** (0.015 mmol, 15 mol%), 2-(cyclohexylmethylene)malonate **3a** (0.15 mmol), and 5Å MS (10 mg). The mixture was stirred in CH_2Cl_2 (1.0 mL) at 35 °C for 0.5 h. Subsequently, $TMSN_3$ **2a** (0.15 mmol) and 2-naphthyl isocyanide **1a** (0.10 mmol) were added at –40 °C. The mixture was stirred at –40 °C for 2 days then at –20 °C for 3 days, and directly purified by flash chromatography on silica gel (eluent: petroleum ether/ethyl acetate = 9/1) to afford the desired product **4a**.

General procedure 4: To an oven-dried tube were added $Mg(OTf)_2$ (0.010 mmol, 10 mol%), dimethyl 2-benzylidenemalonate **3o** (0.10 mmol), isoquinoline **7a** (0.10 mmol), *tert*-butyl isocyanide **1e** (0.10 mmol) and CH_2ClCH_2Cl (0.5 mL). The mixture was stirred in CH_2ClCH_2Cl (1.0 mL) at 35 °C for 48 h, and directly purified by flash chromatography on silica gel (eluent: petroleum ether/ethyl acetate = 9/1) to afford the racemic product **8a**.

Typical procedure for the asymmetric reaction

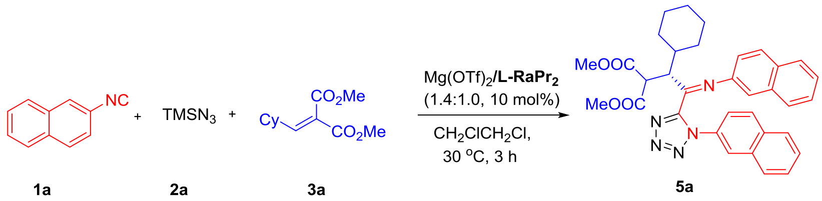

General procedure 1: To an oven-dried tube under nitrogen atmosphere were added $Mg(OTf)_2$ (0.014 mmol, 14 mol%), **L-RaPr$_2$** (0.010 mmol, 10 mol%),

2-(cyclohexylmethylene)malonate **3a** (0.20 mmol) and CH_2ClCH_2Cl (1.0 mL). The mixture were stirred in CH_2ClCH_2Cl (1.0 mL) at 35 °C for 0.5 h. Subsequently, $TMSN_3$ **2a** (0.24 mmol) and 2-naphthyl isocyanide **1a** (0.20 mmol) were added at 30 °C. The mixture was stirred at 30 °C for 3 h, and directly purified by flash chromatography on silica gel (eluent: petroleum ether/ethyl acetate = 9/1) to afford the desired product **5a** (91% yield, 94.5:5.5 e.r.).

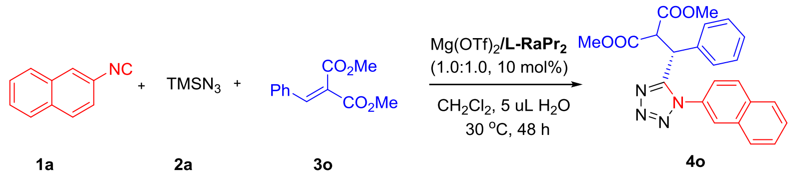

General procedure 2: To an oven-dried tube under nitrogen atmosphere were added $Mg(OTf)_2$ (0.010 mmol, 10 mol%), **L-RaPr$_2$** (0.010 mmol, 10 mol%), dimethyl 2-benzylidenemalonate **3o** (0.10 mmol), 5 μL H_2O and CH_2Cl_2 (1.0 mL). The mixture was stirred in CH_2Cl_2 (1.0 mL) at 35 °C for 0.5 h. Subsequently, $TMSN_3$ **2a** (0.15 mmol) and 2-naphthyl isocyanide **1a** (0.10 mmol) were added at 30 °C. The mixture was stirred at 30 °C for 48 h, and directly purified by flash chromatography on silica gel (eluent: petroleum ether/ethyl acetate = 9/1) to afford the desired product **4o** (50% yield, 90.5:9.5 e.r.).

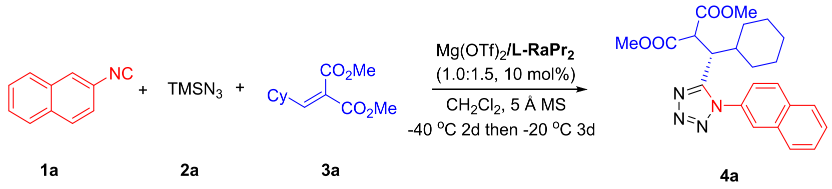

General procedure 3: To an oven-dried tube under nitrogen atmosphere were added $Mg(OTf)_2$ (0.010 mmol, 10 mol%), **L-RaPr$_2$** (0.015 mmol, 15 mol%), 2-(cyclohexylmethylene)malonate **3a** (0.15 mmol), 5Å MS (10 mg) and CH_2Cl_2 (1.0 mL). The mixture was stirred in CH_2Cl_2 (1.0 mL) at 35 °C for 0.5 h. Subsequently, $TMSN_3$ **2a** (0.15 mmol) and 2-naphthyl isocyanide **1a** (0.10 mmol) were added at –40 °C. The mixture was stirred at –40 °C for 2 days then at –20 °C for 3 days, and directly purified by flash chromatography on silica gel (eluent: petroleum ether/ethyl acetate = 9/1) to afford the desired product **4a** (91% yield, 95:5 e.r.).

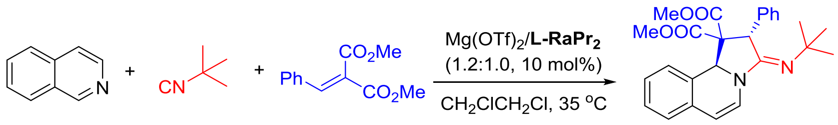

7a **1e** **3o** **8a**

General procedure 4: To an oven-dried tube were added $Mg(OTf)_2$ (0.012 mmol, 12 mol%), **L-RaPr$_2$** (0.010 mmol, 10 mol%), dimethyl 2-benzylidenemalonate **3o** (0.15 mmol) and CH_2ClCH_2Cl (0.5 mL). The mixture was stirred in CH_2ClCH_2Cl (0.5 mL) at 35 °C for 0.5 h. Subsequently, isoquinoline **7a** (0.10 mmol) and *tert*-butyl isocyanide **1e** (0.15 mmol) were added at 35 °C. The mixture was stirred at 35 °C for 48 h, and directly purified by flash chromatography on silica gel (eluent: petroleum ether/ethyl acetate = 9/1) to afford the desired product **8a** (85% yield, 96.5:3.5 e.r.).

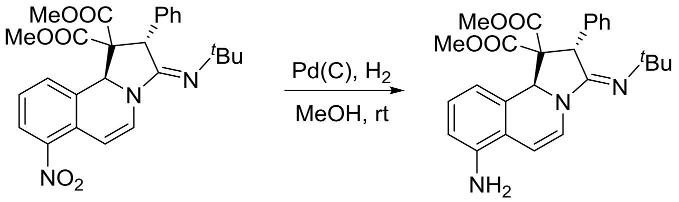

8a: 97:3 e.r. **8v**: 32%yield, 98:2 e.r.

To an oven-dried tube under hydrogen atmosphere were added **8a** (0.1 mmol), Pd-C (0.02 mmol) and MeOH (1 mL). The mixture was stirred for 2h, directly purified by flash chromatography on silica gel (eluent: petroleum ether/ethyl acetate = 1/1) to afford the desired product **8v** (32% yield, 98:2 e.r.).

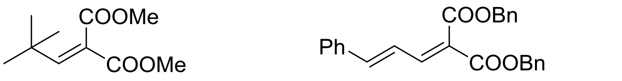

General procedure 3: nr **General procedure 1 and 2**: messy

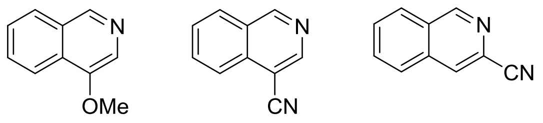

General procedure 4: not detected

Supplementary Figure 1. Unsuccessful substrate scope

Supplementary Tables

Supplementary Table 1. Screening of the metal salts[a]

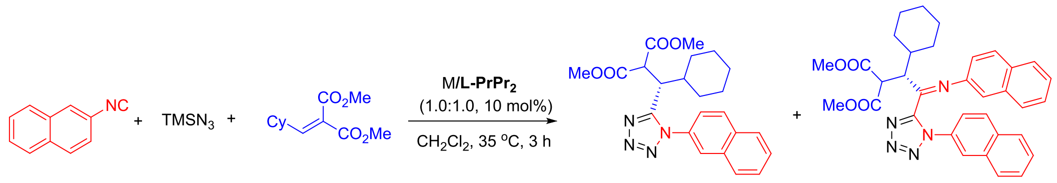

1a 2a 3a 4a 5a

Entry	Metal salts	Yield of **4a** (%)[b]	Yield of **5a** (%)[b]	e.r. of **4a**[c]	e.r. of **5a**[c]
1	$Sc(OTf)_3$	mess	mess	-	-
2	$Mg(OTf)_2$	29	53	82:18	92.5:7.5
3	$Ni(OTf)_2$	mess	mess	-	-
4	$Zn(OTf)_2$	mess	mess	-	-
5	$Yb(OTf)_3$	mess	mess	-	-

[a] Reaction conditions: unless otherwise noted, all reactions were carried out with **1a** (0.10 mmol), **2a** (0.12mmol), **3a** (0.10 mmol) and M/**L-PrPr$_2$** (1.0:1.0, 10 mol%) in CH_2Cl_2 (1.0 mL) at 35 °C. [b] Isolated yield. [c] Determined by chiral HPLC analysis.

Supplementary Table 2. Screening of the ligand[a]

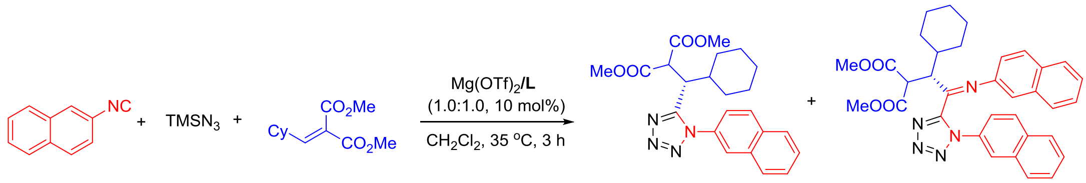

1a 2a 3a 4a 5a

Entry	Ligand	Yield of **4a** (%)[b]	Yield of **5a** (%)[b]	e.r. of **4a**[c]	e.r. of **5a**[c]
1	**L-PrPr$_2$**	29	53	82:18	92.5:7.5
2	**L-RaPr$_3$**	36	43	72:28	86.5:13.5
3	**L-RaPr$_2$**	38	46	83.5:16.5	96:4
4	**L-RaEt$_2$**	-	85	-	86.5:13.5
5	**L-RaEt$_2$Me**	16	72	55.5:44.5	89:11
6	**L-RaMe$_2$**	44	53	race	race
7	**L-RaBn**	-	99	-	race
8	**L-RaCy**	-	99		race
9	**L-RaAd**	-	99		45.5:54.5
10	**L-PiPr$_3$**	41	44	64:36	83.5:16.5
11	**L-PiPr$_2$**	-	97	-	82:18
12	**L-PiEt$_2$**	33	51	55.5:44.5	85.5:14.5
13	**L-PiMe$_2$**	15	77	44:56	66.5:33.5
14	**L-PiPh**	-	88	-	45:55

[a] Reaction conditions: unless otherwise noted, all reaction were carried out with **1a** (0.10 mmol), **2a** (0.12mmol), **3a** (0.10 mmol) and $Mg(OTf)_2$/**L** (1.0:1.0, 10 mol%) in CH_2Cl_2 (1.0 mL) at 35 °C. [b] Isolated yield. [c] Determined by chiral HPLC analysis.

Supplementary Table 3. Investigating the ratio of the metal salts and ligand[a]

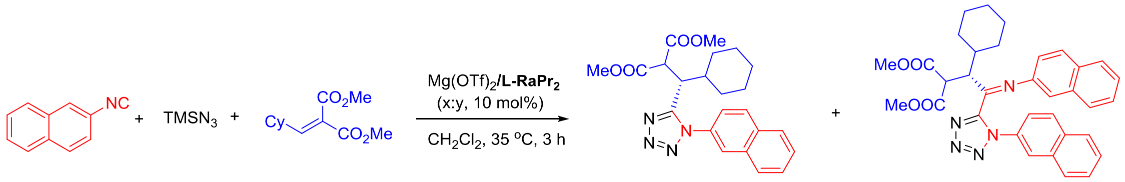

1a 2a 3a 4a 5a

Entry	Ratio of **M:L**	Yield of **4a** (%)[b]	Yield of **5a** (%)[b]	e.r. of **4a**[c]	e.r. of **5a**[c]
1	1.5:1.0	-	88	-	94:6
2	1.2:1.0	18	72	83.5:16.5	95.5:4.5
3	1.0:1.0	36	43	72:28	86.5:13.5
4	1.0:1.2	47	37	84:16	97:3
5	1.0:1.5	53	25	88:12	95.5:4.5

[a] Reaction conditions: unless otherwise noted, all reactions were carried out with **1a** (0.10 mmol), **2a** (0.12mmol), **3a** (0.10 mmol) and $Mg(OTf)_2$/**L-RaPr₂** (10 mol%) in CH_2Cl_2 (1.0 mL) at 35 °C. [b] Isolated yield. [c] Determined by chiral HPLC analysis.

Supplementary Table 4. Optimization of the solvents[a]

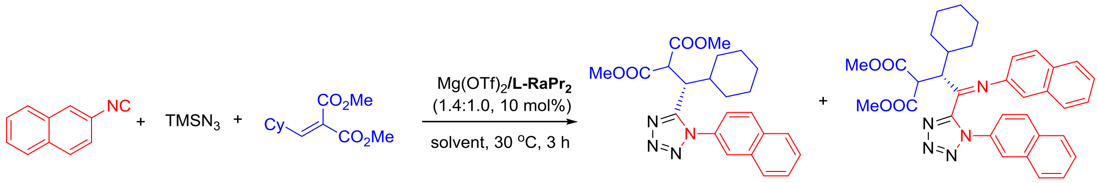

1a 2a 3a 4a 5a

Entry	Solvent	Yield of **4a** (%)[b]	Yield of **5a** (%)[b]	e.r. of **4a**[c]	e.r. of **5a**[c]
1	THF	-	32	-	race
2	Toluene	19	87	70:30	86.5:13.5
3	CH_2Cl_2	-	85	-	94.5:5.5
4	CH_2ClCH_2Cl	-	91	-	94.5:5.5
5	$CHCl_2CHCl_2$	-	88	-	94:6
6[d]	CH_2ClCH_2Cl	-	91	-	94.5:5.5

[a] Reaction conditions: unless otherwise noted, all reactions were carried out with **1a** (0.10 mmol), **2a** (0.12mmol), **3a** (0.10 mmol) and $Mg(OTf)_2$/**RaPr₂** (1.4:1.0, 10 mol%) in solvent (1.0 mL) at 30 °C. [b]

Isolated yield. [c] Determined by chiral HPLC analysis. [d] Reaction were carried out with **1a** (0.20 mmol), **2a** (0.24mmol), **3a** (0.20 mmol) and $Mg(OTf)_2$/**L-RaPr$_2$** (0.014 mol / 0.010 mmol, 1.4:1.0, 10 mol%) in CH_2ClCH_2Cl (1.0 mL) at 30 °C.

Supplementary Table 5. Screening of the reaction temperature[a]

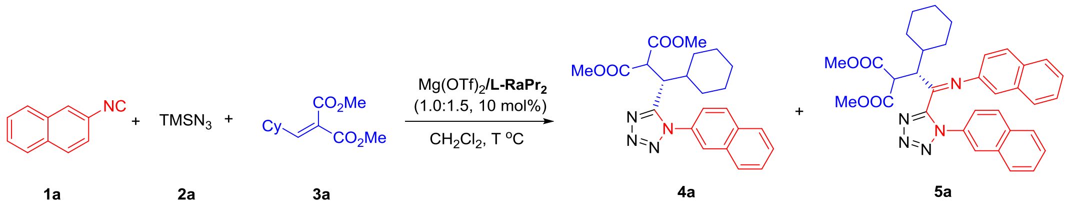

Entry	T (°C)	Yield of **4a** (%)[b]	Yield of **5a** (%)[b]	e.r. of **4a**[c]	e.r. of **5a**[c]
1[d]	35	53	25	88:12	95.5:4.5
2[e]	0	49	49	89:11	95.5:4.5
3[e]	–20	65	30	92.5:7.5	96.5:3.5
4[f]	–30	43	-	94:6	-
5[g]	–40	57	-	94.5:5.5	-
6[g,h]	–20	30 (**5o**)	-	93:7 (**5o**)	-

[a] Reaction conditions: unless otherwise noted, all reactions were carried out with **1a** (0.10 mmol), **2a** (0.12mmol), **3a** (0.10 mmol) and $Mg(OTf)_2$/**L-RaPr$_2$** (1.0:1.5, 10 mol%) in CH_2Cl_2 (1.0 mL) at T °C. [b] Isolated yield. [c] Determined by chiral HPLC analysis. [d] 3 h. [e] 3 days. [f] 4 days. [g] 7 days. [h] For dimethyl 2-benzylidenemalonate.

Supplementary Table 6. Screening of the reaction additions[a]

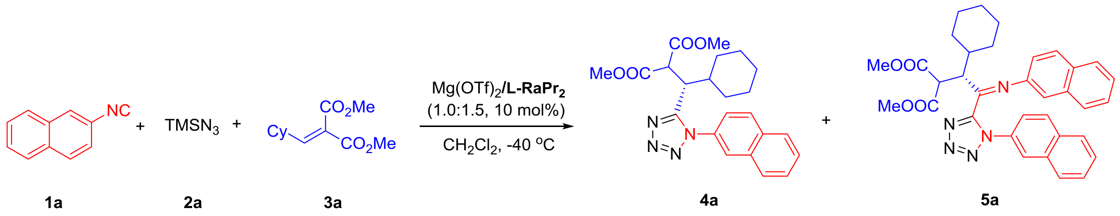

Entry	Additive	Yield of **4a** (%)[b]	Yield of **5a** (%)[b]	e.r. of **4a**[c]	e.r. of **5a**[c]
1[d]	$NaBAr^F_4$	47	53	92:8	98:2
2	3 Å MS	69	-	95:5	-
3	4 Å MS	72	-	95:5	-
4	5 Å MS	73	-	95:5	-
5[e]	5 Å MS	85	-	93:7	-
6[f]	5 Å MS	91	-	95:5	-

[a] Reaction conditions: unless otherwise noted, all reactions were carried out with **1a** (0.10 mmol), **2a**

(0.12mmol), **3a** (0.10 mmol) and Mg(OTf)$_2$/**L-RaPr$_2$** (1.0:1.5, 10 mol%) in CH_2Cl_2 (1.0 mL) at –40 °C for 7 days. [b] Isolated yield. [c] Determined by chiral HPLC analysis. [d] 2 days. [e] –20 °C for 4 days. [f] Reaction were carried out with **1a** (0.10 mmol), **2a** (0.15mmol), **3a** (0.15 mmol) and Mg(OTf)$_2$/**RaPr$_2$** (1.0:1.5, 10 mol%) in CH_2Cl_2 (1.0 mL) at –40 °C for 2 days, then –20 °C for 3 days.

Supplementary Table 7. Screening of the ligand[a]

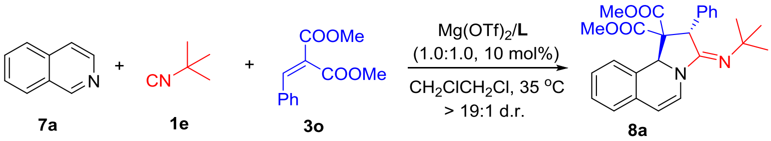

Entry	Ligand	Yield (%)[b]	e.r.[c]
1	**L-RaPr$_2$**	42	96:4
2	**L-PrPr$_2$**	19	71.5:28.5
3	**L-PiPr$_2$**	36	60:40
4	**L-RaPr$_3$**	12	95:5
5	**L-RaEt$_2$**	20	85:15
6	**L-RaMe$_2$**	37	race
7	**L-RaPh**	88	47.5:52.5
8	**L-RaAd**	68	43.5:56.5

[a] Unless otherwise noted, all reactions were carried out with **7e** (0.10 mmol), **1e** (0.15mmol), **3o** (0.10 mmol) and Mg(OTf)$_2$/**L-RaPr$_2$** (1.0:1.0, 10 mol%) in CH_2ClCH_2Cl (0.5 mL) at 35 °C for 2 days. [b] Isolated yield. [c] Determined by chiral HPLC analysis.

Supplementary Table 8. Optimization of the solvents[a]

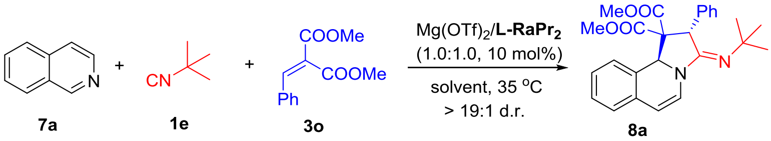

Entry	Solvent	Yield (%)[b]	e.r.[c]
1	CH_2Cl_2	41	95.5:4.5
2	CH_2ClCH_2Cl	42	96:4
3	$CHCl_2CHCl_2$	40	96:4
4	THF	nr	-
5	Toluene	trace	-
6	Ethyl acetate	trace	-

[a] Unless otherwise noted, all reactions were carried out with **7a** (0.10 mmol), **1e** (0.15 mmol), **3o** (0.10

mmol) and $Mg(OTf)_2$/**L-RaPr₂** (1.0:1.0, 10 mol%) in solvent (0.5 mL) at 35 °C for 2 days. [b] Isolated yield. [c] Determined by chiral HPLC analysis.

Supplementary Table 9. Investigating the ratio of the metal salts and ligand[a]

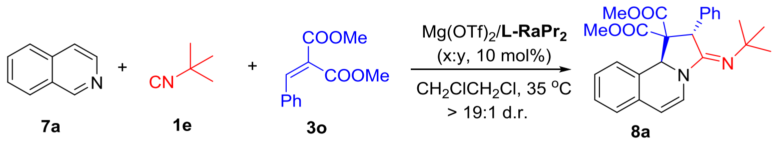

Entry	Ratio of **M:L**	Yield (%)[b]	e.r.[c]
1	1.5:1.0	61	90.5:9.5
2	1.2:1.0	57	95.5:4.5
3	1.0:1.0	42	96:4
4	1.0:1.2	40	96:4
5	1.0:1.5	31	96:4
6[d]	1.2:1.0	85	96.5:3.5

[a] Unless otherwise noted, all reactions were carried out with **7e** (0.10 mmol), **1e** (0.15mmol), **3o** (0.10 mmol) and $Mg(OTf)_2$/**L-RaPr₂** (x:y, 10 mol%) in CH_2ClCH_2Cl (0.5 mL) at 35 °C for 2 days. [b] Isolated yield. [c] Determined by chiral HPLC analysis. [d] **3o** was 0.15 mmol.

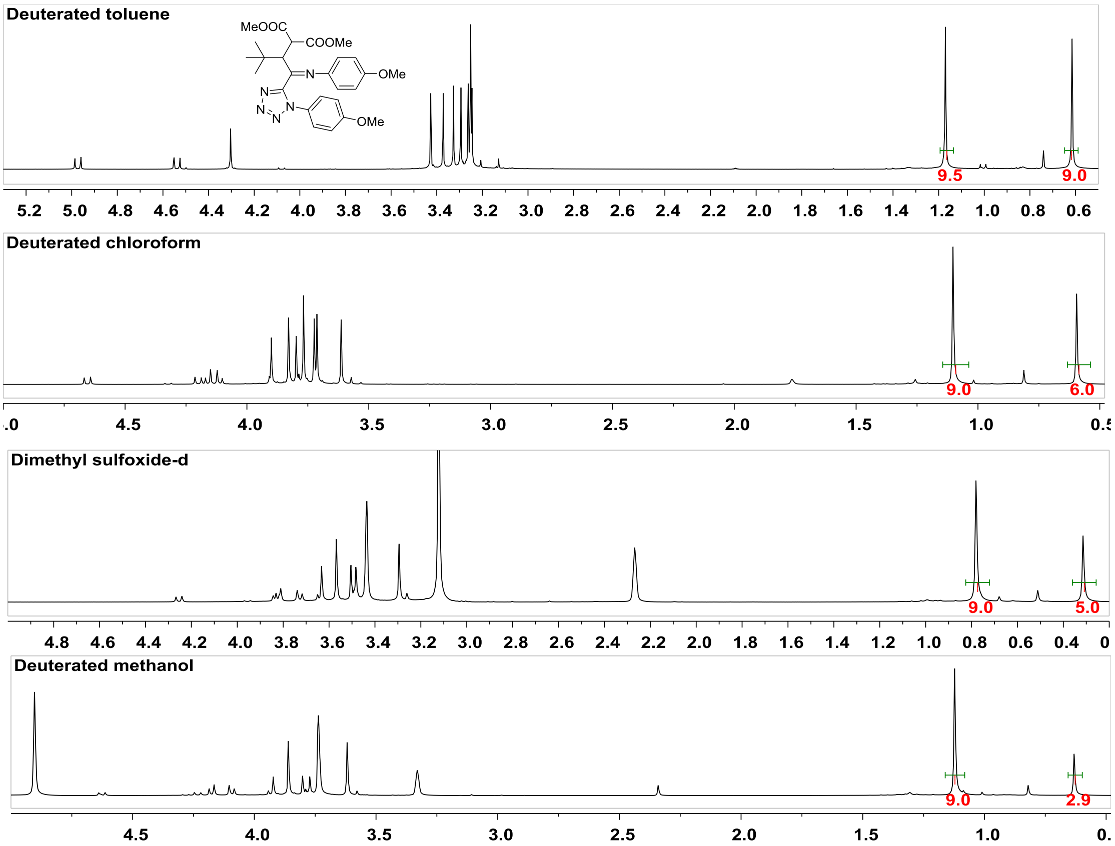

Supplementary Figure 2. NMR spectra of four-molecule tetrazole **5z** in different deuterium solvents

Supplementary Discussion 1. The NMR spectra could reversibly change in different solvents. It indicates rotamers exists in solution of four-molecule product.[5-6]

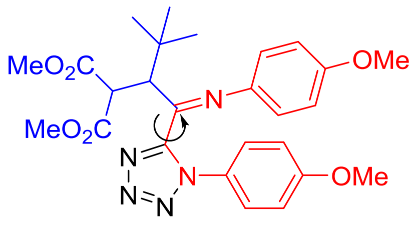

5z: 91% yield, 85:15 e.r.

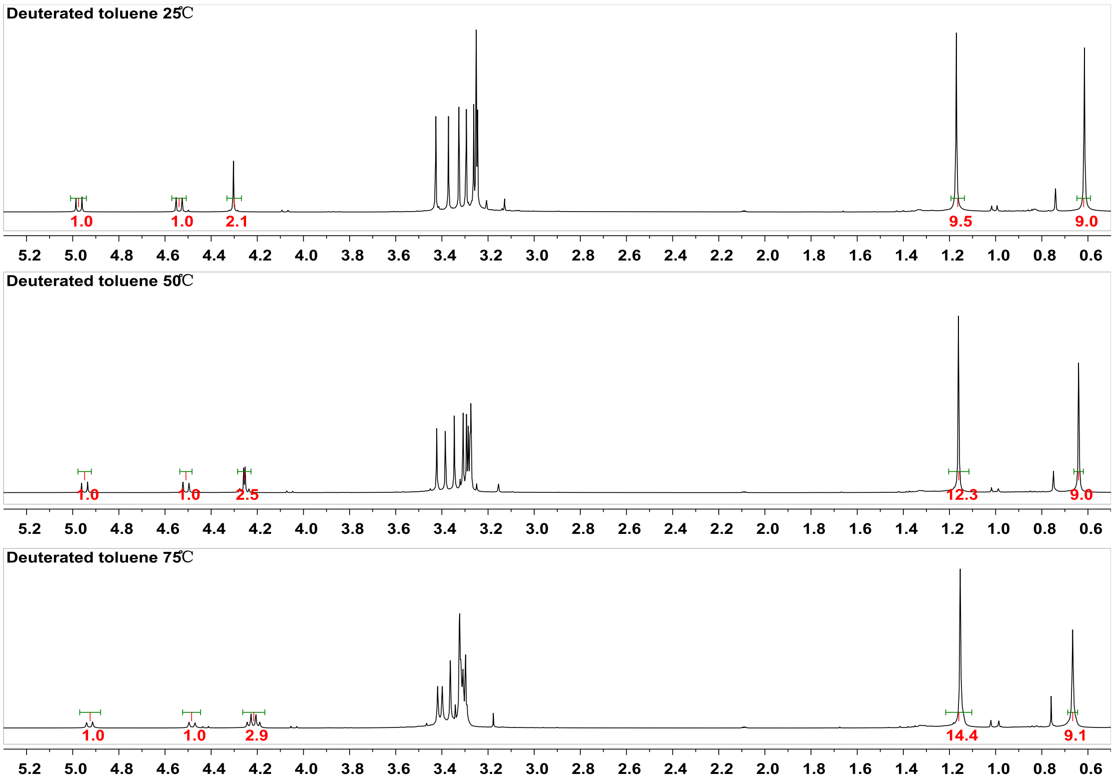

Supplementary Figure 3. NMR spectra of four-molecule tetrazole **5z** in different temperature

Supplementary Discussion 2. The NMR spectra could reversibly change in different temperature. It indicates rotamers exists in solution of four-molecule product.

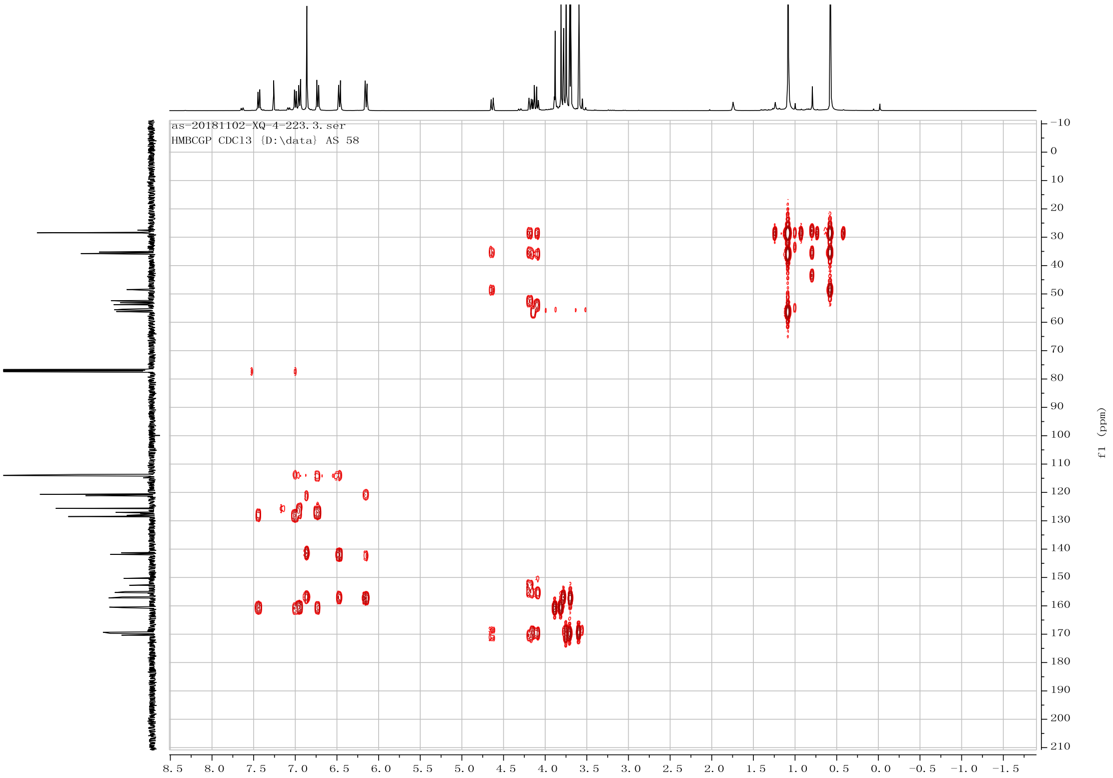

Supplementary Figure 4. HMBCGP of four-molecule tetrazole **5z** **Supplementary**

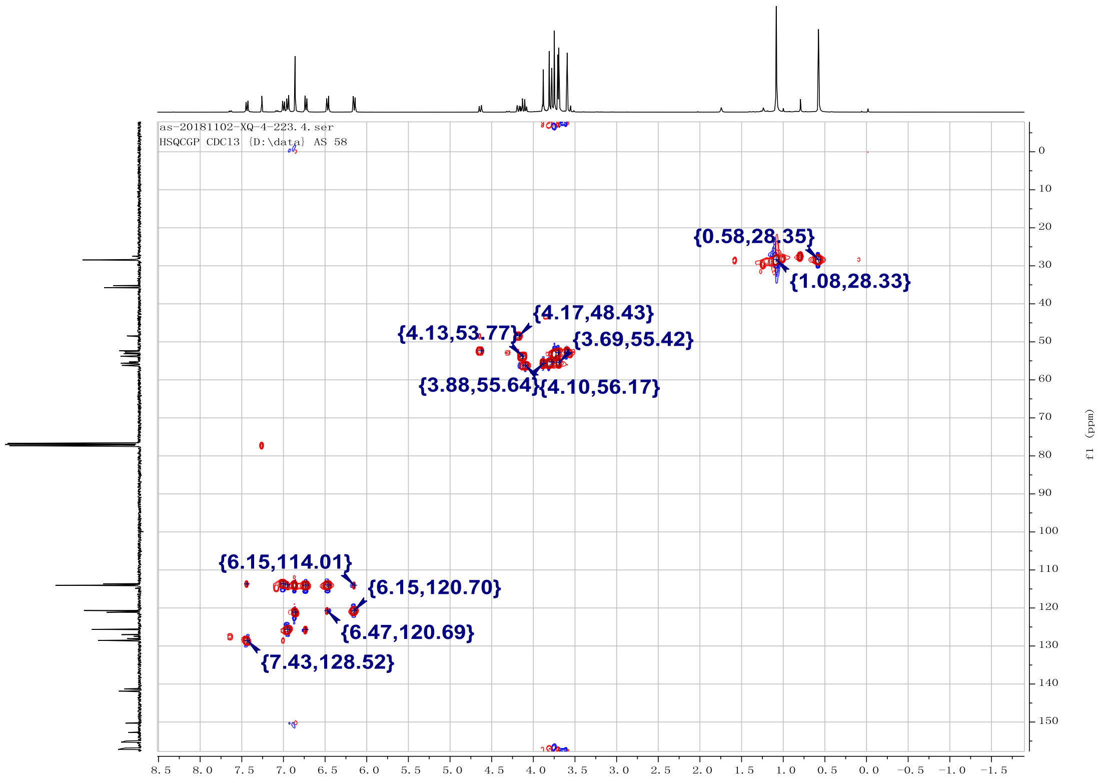

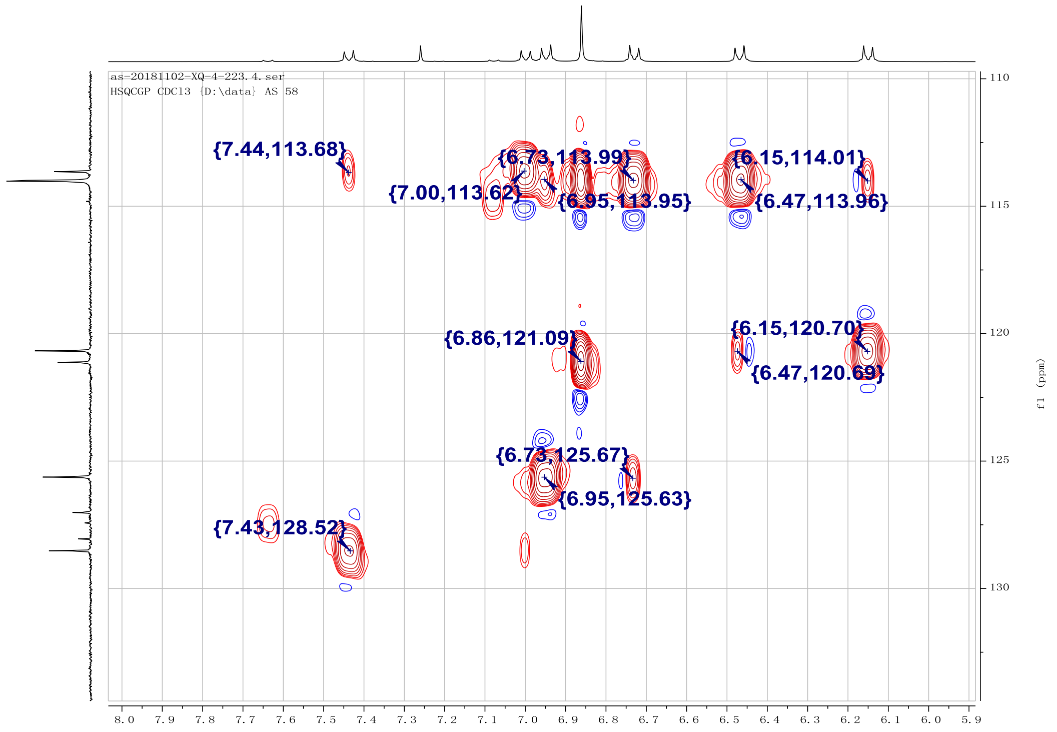

Figure 5. HSQCGP of four-molecule tetrazole **5z**

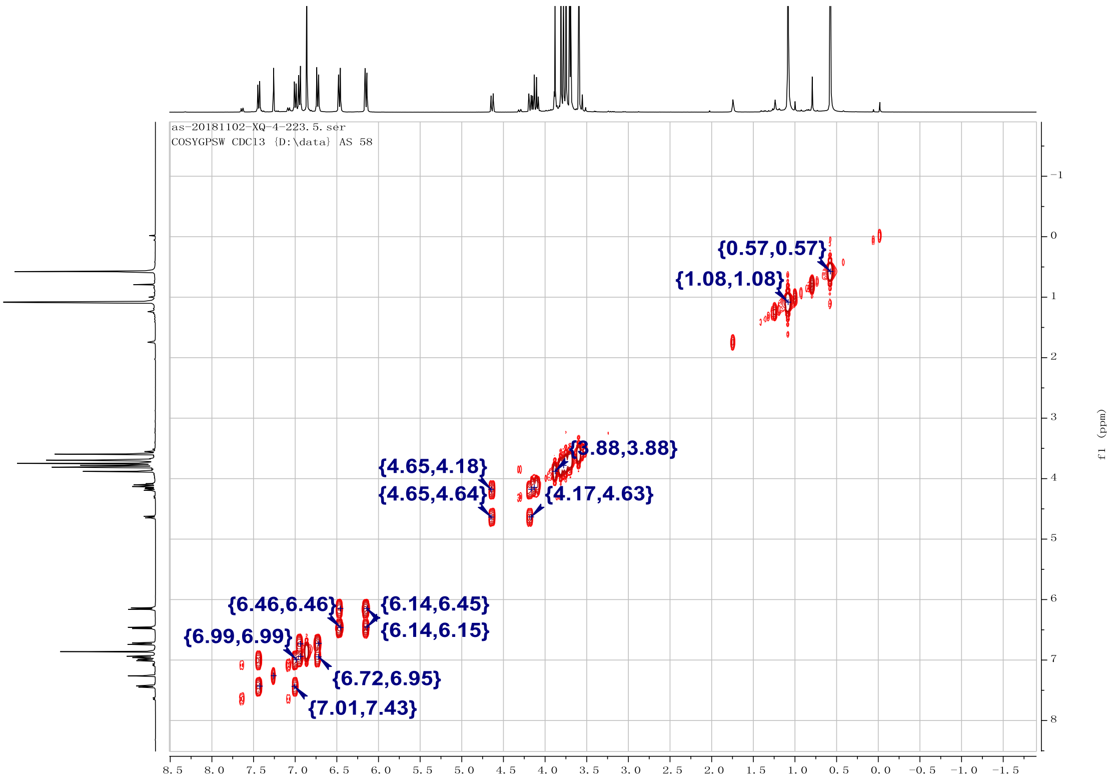

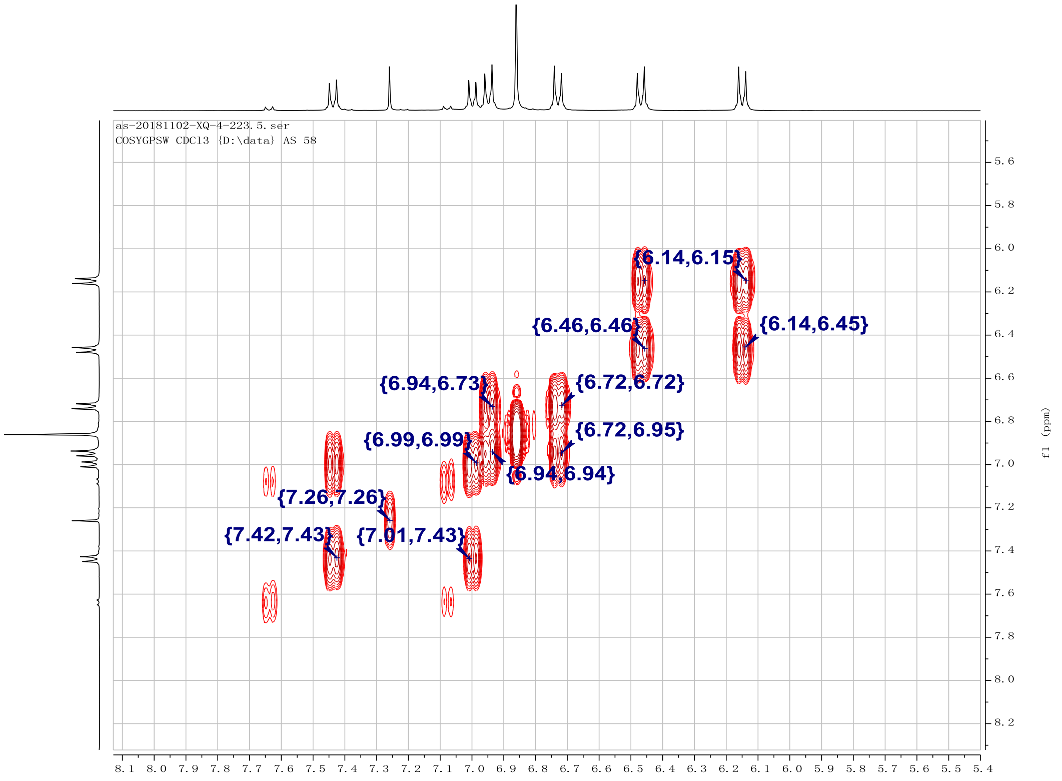

Supplementary Figure 6. COSYGPSW of four-molecule tetrazole **5z**

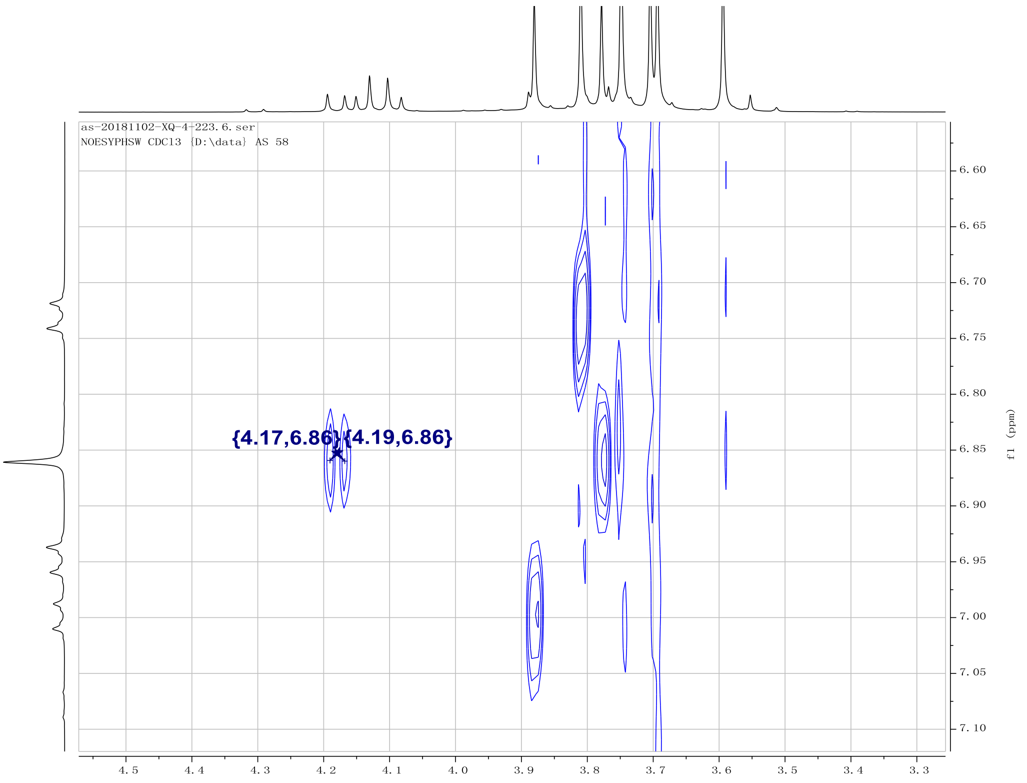

Supplementary Figure 7. NOESYPHSW of four-molecule tetrazole **5z**

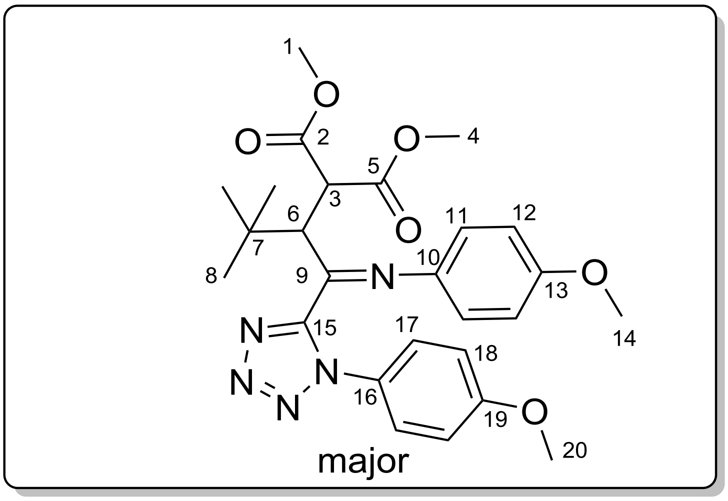

1: 3.59, 52.6;	2: 169.3;
3: 4.13, 53.8;	4: 3.70, 52.8;
5: 169.7;	6: 4.10, 56.2;
7: 35.8;	8: 1.08, 28.5;
9: 150.3;	10: 127.0;
11: 6.95, 125.6;	12: 6.73, 114.0;
13: 160.5;	14: 3.81, 55.6;
15: 155.3;	16: 141.9;
17: 6.15, 120.7;	18: 6.47, 114.0;
19: 157.2;	20: 3.69, 55.4;

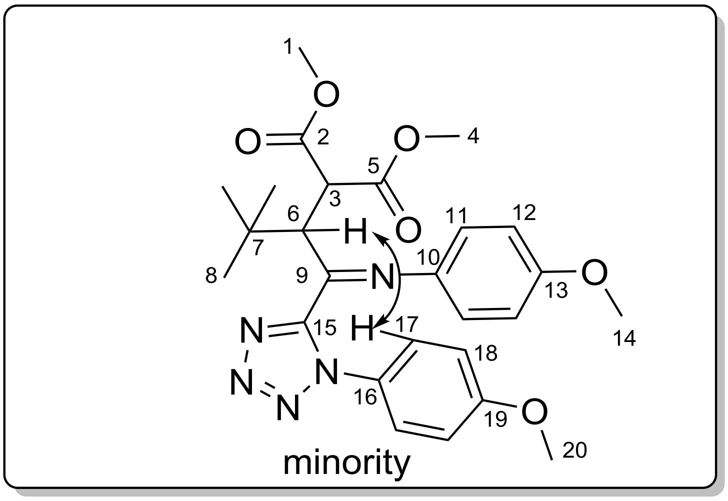

1: 3.75, 53.1; 2: 170.3;
3: 4.64, 52.4; 4: 3.75, 53.1;
5: 169.3; 6: 4.18, 48.4;
7: 35.2; 8: 0.58, 28.4;
9: 152.7; 10: 128.0;
11: 7.00, 113.7; 12: 7.44, 128.5;
13: 160.7; 14: 3.88, 55.6;
15: 155.0; 16: 141.3;
17: 6.86, 121.1; 18: 6.86, 114.0;
19: 156.8; 20: 3.78, 55.3;

The analytical and spectral characterization data of products

Dimethyl 2-{cyclohexyl[1-(naphthalen-2-yl)-1H-tetrazol-5-yl]methyl}malonate (4a)

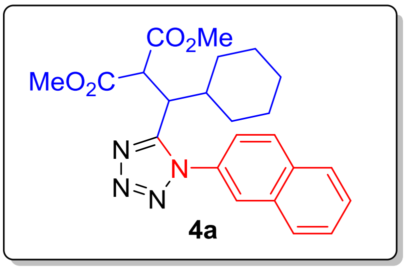

Coreless oil; 91% yield, 95:5 e.r.; $[\alpha]^{21}_{D}$ = –23.2 (c = 0.76 in CH_2Cl_2).

HPLC DAICEL CHIRALCEL IC, *n*-hexane/2-propanol = 70/30, flow rate = 1.0 mL/min, λ = 254 nm, t_R (minor) = 9.05 min, t_R (major) = 11.05 min.

IR (neat): 2928, 1744, 1441, 1263, 1159, 819 and 752 cm^{-1}.

^{1}H NMR (400 MHz, $CDCl_3$) δ = 8.26 (s, 1H), 8.10 – 8.02 (m, 1H), 8.01 – 7.92 (m, 2H), 7.81 – 7.82 (m, 1H), 7.67 – 7.55 (m, 2H), 4.52 (d, J = 11.6 Hz, 1H), 3.93 (dd, J = 11.6, 4.4 Hz, 1H), 3.79 (s, 3H), 3.66 (s, 3H), 1.89 – 1.61 (m, 2H), 1.56 – 1.43 (m, 3H), 1.13 – 0.82 (m, 5H), 0.56 – 0.34 (m, 1H).

^{13}C{^{1}H} NMR (101 MHz, $CDCl_3$) δ = 168.7, 168.3, 155.0, 133.6, 133.0, 131.7, 130.1, 128.8, 128.1, 128.0, 127.6, 125.4, 123.2, 53.5, 53.2, 40.4, 39.3, 31.7, 27.4, 26.3, 26.1, 25.8.

HRMS (ESI-FT) calcd for $C_{23}H_{27}N_4O_4^+$ ([M+H$^+$]) = 423.2027, Found 423.2021.

Chiral HPLC spectrum **4a**:

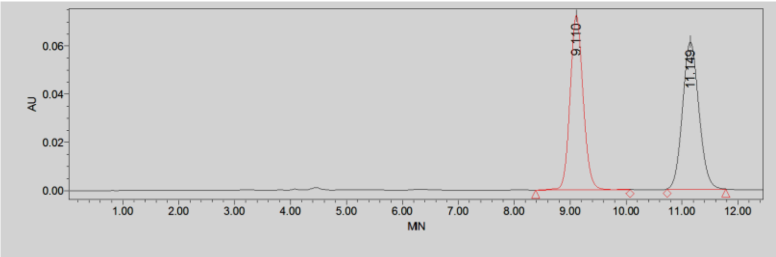

	Retention Time	Area	% Area
1	9.110	1187777	48.21
2	11.149	1275855	51.79

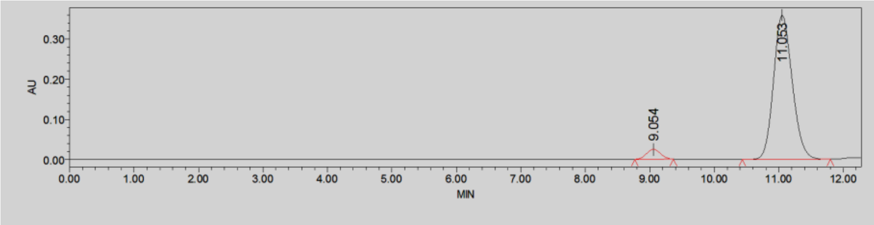

	Retention Time	Area	% Area
1	9.054	380179	4.99
2	11.053	7233340	95.01

Diethyl 2-{cyclohexyl[1-(naphthalen-2-yl)-1H-tetrazol-5-yl]methyl}malonate (4b)

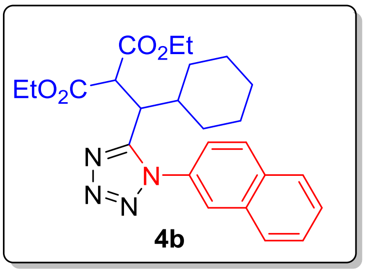

Coreless oil; 87% yield, 96.5:3.5 e.r.; $[\alpha]^{21}_{D}$ = –31.9 (c = 0.54 in CH_2Cl_2).

HPLC DAICEL CHIRALCEL IC, *n*-hexane/2-propanol = 70/30, flow rate = 1.0 mL/min, λ = 254 nm, t_R (minor) = 7.83 min, t_R (major) = 9.72 min.

IR (neat): 2932, 2857, 1742, 1445, 1306, 1101, 1026, 862, 819 and 752 cm^{-1}.

^{1}H NMR (400 MHz, $CDCl_3$) δ = 8.27 (s, 1H), 8.10 – 8.03 (m, 1H), 8.02 – 7.92 (m, 2H), 7.84 – 7.73 (m, 1H), 7.72 –7.44 (m, 2H), 4.47 (d, J = 11.6 Hz, 1H), 4.34 – 4.18 (m, 2H), 4.16 – 4.05 (m, 2H), 3.94 (dd, J = 11.6, 4.4 Hz, 1H), 1.81 (d, J = 11.8 Hz, 1H), 1.70 (d, J = 11.6 Hz, 1H), 1.58 –1.43 (m, 3H), 1.29 (t, J = 7.2 Hz, 3H), 1.19 (t, J = 7.2 Hz, 3H), 1.10 – 0.77 (m, 5H), 0.52 – 0.33 (m, 1H).

^{13}C{^{1}H} NMR (101 MHz, $CDCl_3$) δ = 168.3, 168.0, 155.1, 133.6, 133.1, 131.7, 130.1, 128.8, 128.1, 128.0, 127.5, 125.4, 123.2, 62.2, 62.1, 53.8, 40.4, 39.3, 31.8, 27.4, 26.4, 26.1, 25.8, 14.2, 14.0.

HRMS (ESI-FT) calcd for $C_{25}H_{31}N_4O_4^+$ ($[M+H^+]$) = 451.2340, Found 451.2336.

Chiral HPLC spectrum **4b**:

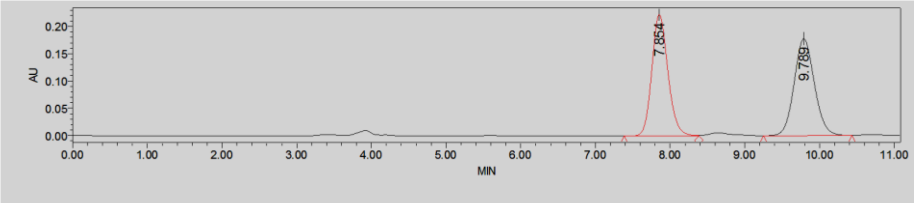

	Retention Time	Area	% Area
1	7.854	3337122	49.95
2	9.789	3343512	50.05

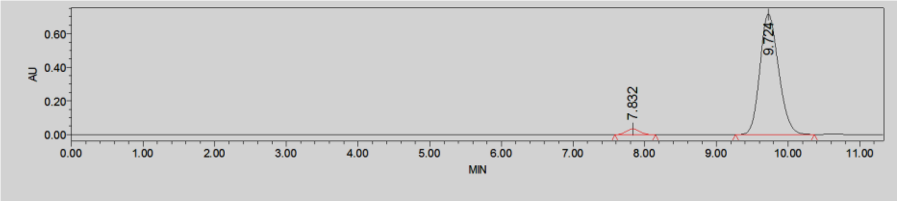

	Retention Time	Area	% Area
1	7.832	468136	3.48
2	9.724	12969465	96.52

Diisopropyl 2-{cyclohexyl[1-(naphthalen-2-yl)-1H-tetrazol-5-yl]methyl}malonate (4c)

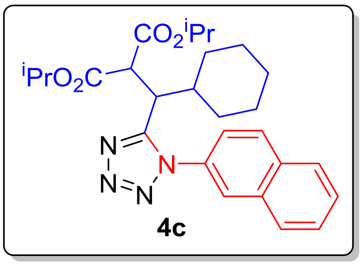

Coreless oil; 52% yield, 96.5:3.5 e.r.; $[\alpha]^{21}_{D}$ = –32.0 (*c* = 0.34 in CH_2Cl_2).

HPLC DAICEL CHIRALCEL IC, *n*-hexane/2-propanol = 70/30, flow rate = 1.0 mL/min, λ = 254 nm, t_R (minor) = 5.93 min, t_R (major) = 7.22 min.

IR (neat): 2982, 2932, 2857, 1740, 1512, 1447, 1370, 1267, 1103, 822 and 752 cm^{-1}.

^{1}H NMR (400 MHz, $CDCl_3$) δ = 8.29 (s, 1H), 8.10 – 8.03 (m, 1H), 8.02 – 7.93 (m, 2H), 7.85 – 7.86 (m, 1H), 7.69 – 7.58 (m, 2H), 5.21 – 5.03 (m, 1H), 5.01 – 4.84 (m, 1H), 4.41 (d, *J* = 11.6 Hz, 1H), 3.93 (dd, *J* = 11.6, 4.4 Hz, 1H), 1.85 – 1.63 (m, 2H), 1.57 – 1.42 (m, 3H), 1.28 (dd, *J* = 6.4, 3.2 Hz, 6H), 1.17 (dd, *J* = 6.4, 3.2 Hz, 6H), 1.11 – 0.80 (m, 5H), 0.50 – 0.34 (m, 1H).

^{13}C{^{1}H} NMR (101 MHz, $CDCl_3$) δ = 167.9, 167.7, 155.2, 133.6, 133.1, 131.8, 130.1, 128.9, 128.1, 127.9, 127.5, 125.3, 123.3, 69.9, 69.7, 54.3, 40.4, 39.2, 31.8, 27.4, 26.4, 26.1, 25.9, 21.8, 21.7, 21.6, 21.5.

HRMS (ESI-FT) calcd for $C_{27}H_{35}N_4O_4^+$ ([M+H$^+$]) = 479.2653, Found 479.2646.

Chiral HPLC spectrum **4c**:

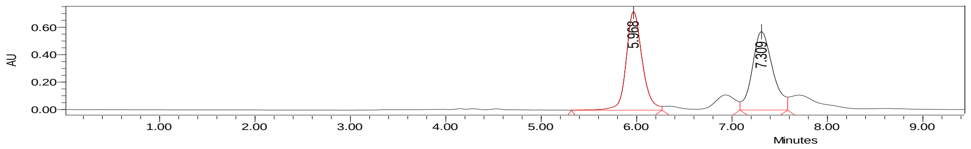

	Retention Time	Area	% Area
1	5.968	8328302	49.42
2	7.309	8524263	50.58

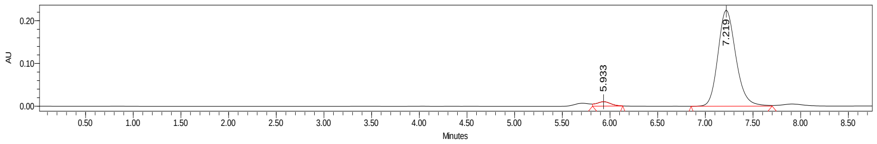

	Retention Time	Area	% Area
1	5.933	105489	3.48
2	7.219	2925446	96.52

Dibenzyl 2-{cyclohexyl[1-(naphthalen-2-yl)-1H-tetrazol-5-yl]methyl}malonate (4d)

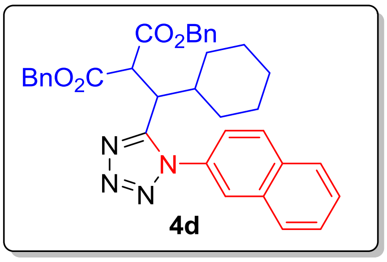

4d

Coreless oil; 80% yield, 97:3 e.r.; $[\alpha]^{21}_{D}$ = –15.0 (*c* = 0.67 in CH_2Cl_2).

HPLC DAICEL CHIRALCEL IC, *n*-hexane/2-propanol = 70/30, flow rate = 1.0 mL/min, λ = 254 nm, t_R (minor) = 11.52 min, t_R (major) = 12.60 min.

IR (neat): 2930, 2855, 1744, 1506, 1449, 1265, 905, 746 and 698 cm^{-1}.

^{1}H NMR (400 MHz, $CDCl_3$) δ = 8.18 (s, 1H), 8.07 – 8.00 (m, 1H), 8.00 – 7.91 (m, 2H), 7.75 – 7.70 (m, 1H), 7.68 – 7.58 (m, 2H), 7.37 – 7.28 (m, 8H), 7.24 – 7.19 (m, 2H), 5.20 (dd, *J* = 30.0, 12.0 Hz, 2H), 5.12 – 5.02 (m, 2H), 4.61 (d, *J* = 11.6 Hz, 1H), 3.95 (dd, *J* = 11.6, 4.4 Hz, 1H), 1.79 – 1.72 (m, 1H), 1.64 – 1.55 (m, 1H), 1.47 – 1.33 (m, 3H), 0.99 – 0.78 (m, 5H), 0.49 – 0.27 (m, 1H).

^{13}C{^{1}H} NMR (101 MHz, $CDCl_3$) δ = 168.0, 167.6, 154.9, 135.0, 134.9, 133.6, 133.0, 131.6, 130.1, 128.8, 128.7, 128.7, 128.6, 128.5, 128.1, 128.0, 127.5, 125.3, 123.2,

67.8, 53.8, 40.1, 39.4, 31.7, 27.3, 26.2, 25.8, 25.7.

HRMS (ESI-FT) calcd for $C_{35}H_{35}N_4O_4^+$ ([M+H$^+$]) = 575.2653, Found 575.2653.

Chiral HPLC spectrum **4d**:

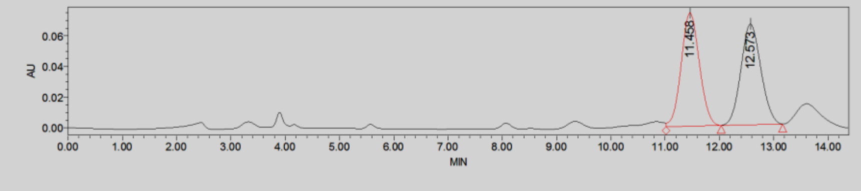

	Retention Time	Area	% Area
1	11.458	1721760	50.55
2	12.573	1684014	49.45

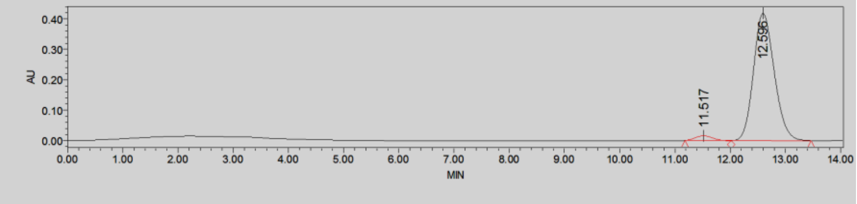

	Retention Time	Area	% Area
1	11.517	333334	2.96
2	12.596	10929321	97.04

Dimethyl 2-{cycloheptyl[1-(naphthalen-2-yl)-1H-tetrazol-5-yl]methyl}malonate (4e)

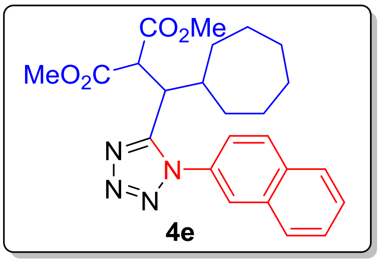

Coreless oil; 22% yield, 96:4 e.r.; $[\alpha]^{21}_{D}$ = –26.2 (*c* = 0.13 in CH_2Cl_2).

HPLC DAICEL CHIRALCEL IC, *n*-hexane/2-propanol = 70/30, flow rate = 1.0 mL/min, λ = 254 nm, t_R (minor) = 8.59 min, t_R (major) = 10.31 min.

IR (neat): 2928, 2857, 1746, 1603, 1512, 1439, 1312, 1184, 1024, 864 and 750 cm^{-1}.

^{1}H NMR (400 MHz, $CDCl_3$) δ = 8.29 (s, 1H), 8.10 – 8.03 (m, 1H), 8.03 – 7.93 (m, 2H), 7.82 – 7.84 (m, 1H), 7.69 – 7.56 (m, 2H), 4.52 (d, *J* = 11.6 Hz, 1H), 3.98 (dd, *J* = 11.6, 3.6 Hz, 1H), 3.79 (s, 3H), 3.67 (s, 3H), 1.99 – 1.86 (m, 1H), 1.68 – 1.50 (m, 2H), 1.29 – 1.20 (m, 9H), 0.80 – 0.62 (m, 1H).

^{13}C{^{1}H} NMR (101 MHz, $CDCl_3$) δ = 168.7, 168.4, 155.0, 133.6, 133.1, 131.9, 130.2,

128.9, 128.1, 128.0, 127.6, 125.3, 123.2, 77.48, 54.0, 53.2, 41.5, 40.2, 33.7, 28.6, 27.9, 27.6, 26.7, 26.3.

HRMS (ESI-FT) calcd for $C_{24}H_{29}N_4O_4^+$ ([M+H$^+$]) = 437.2183, Found 437.2180.

Chiral HPLC spectrum **4e**:

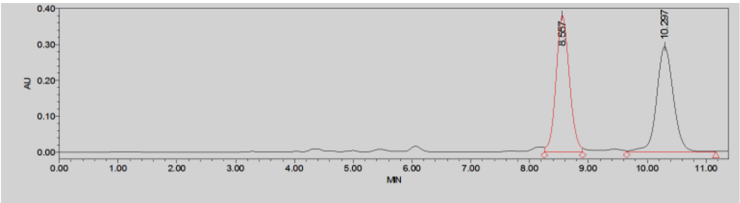

	Retention Time	Area	% Area
1	8.557	5983793	50.94
2	10.297	5762979	49.06

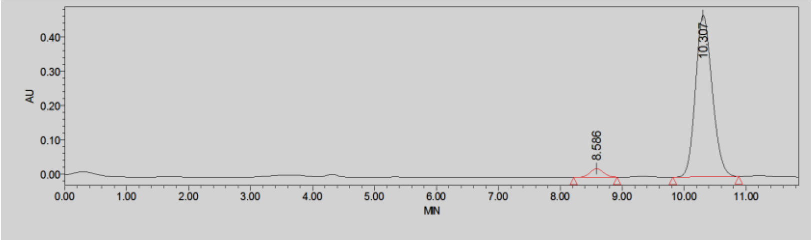

	Retention Time	Area	% Area
1	8.586	368369	3.99
2	10.307	8868054	96.01

Dimethyl 2-{cyclopentyl[1-(naphthalen-2-yl)-1H-tetrazol-5-yl]methyl}malonate (4f)

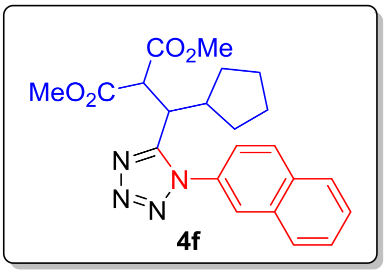

Coreless oil; 90% yield, 94:6 e.r.; $[\alpha]^{21}_D$ = –35.5 (c = 0.54 in CH_2Cl_2).

HPLC DAICEL CHIRALCEL IC, *n*-hexane/2-propanol = 70/30, flow rate = 1.0 mL/min, λ = 254 nm, t_R (minor) = 10.97 min, t_R (major) = 11.78 min.

IR (neat): 2928, 2857, 1746, 1506, 1450, 1312, 1024, 865, 819 and 749 cm^{-1}.

^{1}H NMR (400 MHz, $CDCl_3$) δ = 8.26 (s, 1H), 8.10 – 8.04 (m, 1H), 8.03 – 7.93 (m, 2H), 7.81 – 7.75 (m, 1H), 7.69 – 7.59 (m, 2H), 4.40 (d, J = 11.2 Hz, 1H), 4.02 (dd, J = 11.2, 6.4 Hz, 1H), 3.79 (s, 3H), 3.67 (s, 3H), 2.12 – 1.91 (m, 1H), 1.64 – 1.52 (m, 1H),

1.46 – 1.29 (m, 6H), 0.75 – 0.51 (m, 1H).

^{13}C{^{1}H} NMR (101 MHz, $CDCl_3$) δ = 168.5, 168.4, 155.6, 133.7, 133.1, 131.5, 130.2, 128.8, 128.1, 128.0, 127.6, 125.4, 123.2, 55.5, 53.2, 43.0, 37.5, 30.6, 27.6, 24.7, 24.0.

HRMS (ESI-FT) calcd for $C_{22}H_{25}N_4O_4^+$ ([M+H$^+$]) = 409.1870, Found 409.1866.

Chiral HPLC spectrum **4f**:

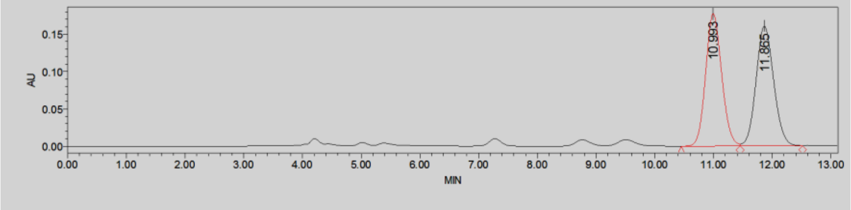

	Retention Time	Area	% Area
1	10.993	3366363	50.18
2	11.865	3342596	49.82

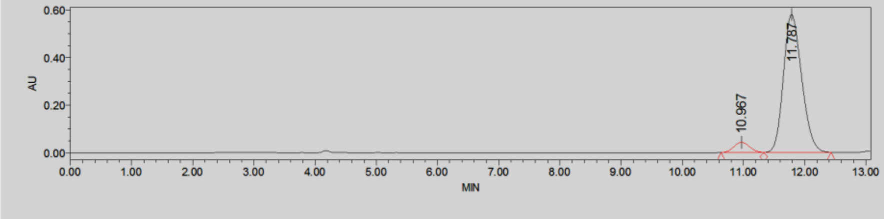

	Retention Time	Area	% Area
1	10.967	765998	6.00
2	11.787	11997390	94.00

Dimethyl 2-{cyclobutyl[1-(naphthalen-2-yl)-1H-tetrazol-5-yl]methyl}malonate (4g)

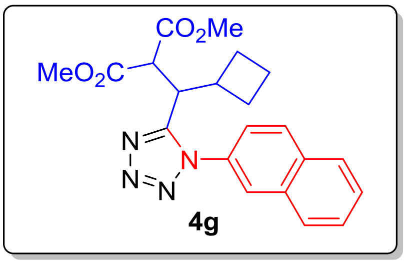

Coreless oil; 51% yield, 90:10 e.r.; $[\alpha]^{21}{}_D$ = –59.7 (c = 0.51 in CH_2Cl_2).

UPC^2 Phenomenex CHIRALCEL IC-3, CO_2/ CH_3OH = 90/10, flow rate = 2.0 mL/min, λ = 254 nm, t_R (minor) = 4.73 min, t_R (major) = 5.98 min.

IR (neat): 2955, 1746, 1512, 1439, 1314, 1250, 820 and 752 cm^{-1}.

^{1}H NMR (400 MHz, $CDCl_3$) δ = 8.22 (s, 1H), 8.12 – 8.05 (m, 1H), 8.05 – 7.91 (m, 2H), 7.78 – 7.72 (m, 1H), 7.72 – 7.54 (m, 2H), 4.26 (d, J = 11.2 Hz, 1H), 3.86 (dd, J =

10.8, 8.8 Hz, 1H), 3.75 (s, 3H), 3.66 (s, 3H), 2.62 (dd, J = 16.4, 8.0 Hz, 1H), 1.82 – 1.29 (m, 6H).

^{13}C{^{1}H} NMR (101 MHz, $CDCl_3$) δ = 168.4, 168.1, 155.7, 133.7, 133.1, 131.4, 130.3, 128.9, 128.2, 128.1, 127.7, 125.5, 123.2, 54.3, 53.1, 39.1, 38.5, 27.4, 25.4, 18.2.

HRMS (ESI-FT) calcd for $C_{21}H_{23}N_4O_4^+$ ([M+H$^+$]) = 395.1714, Found 395.1710.

Chiral HPLC spectrum **4g**:

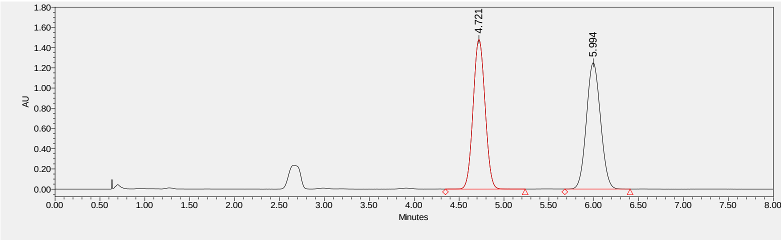

	Retention Time	% Area
1	4.721	50.05
2	5.994	49.95

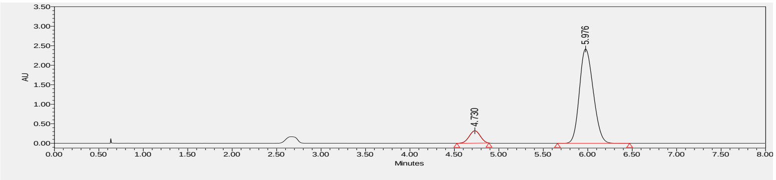

	Retention Time	% Area
1	4.730	9.43
2	5.976	90.57

Dimethyl 2-{cyclopropyl[1-(naphthalen-2-yl)-1H-tetrazol-5-yl]methyl}malonate (4h)

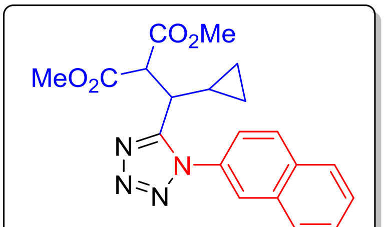

4h

Coreless oil; 30% yield, 55:45 e.r.; $[\alpha]^{21}_{D}$ = –17.0 (c = 0.19 in CH_2Cl_2).

HPLC DAICEL CHIRALCEL IC, n-hexane/2-propanol = 70/30, flow rate = 1.0 mL/min, λ = 254 nm, t_R (minor) = 12.58 min, t_R (major) = 20.87 min.

IR (neat): 2957, 1744, 1440, 1315, 1267, 1026 and 752 cm^{-1}.

^{1}H NMR (400 MHz, $CDCl_3$) δ = 8.23 –8.16 (m, 1H), 8.10 – 8.04 (m, 1H), 8.04 –7.91

(m, 2H), 7.73 (dd, J = 8.8, 2.0 Hz, 1H), 7.70 – 7.53 (m, 2H), 4.47 (d, J = 11.2 Hz, 1H), 3.79 (s, 3H), 3.69 (s, 3H), 3.29 – 3.18 (m, 1H), 1.11 – 0.98 (m, 1H), 0.52 – 0.41 (m, 1H), 0.33 – 0.23 (m, 1H), 0.00 – -0.06 (m, 1H), -0.21 – -0.33 (m, 1H).

$^{13}C\{^1H\}$ NMR (101 MHz, $CDCl_3$) δ = 168.4, 168.3, 156.8, 133.8, 133.1, 131.3, 130.3, 128.8, 128.2, 128.1, 127.7, 125.4, 123.1, 77.48, 55.7, 53.1, 53.0, 38.6, 14.3, 5.2, 2.3.

HRMS (ESI-FT) calcd for $C_{20}H_{21}N_4O_4^+$ ($[M+H^+]$) = 381.1557, Found 381.1554.

Chiral HPLC spectrum **4h**:

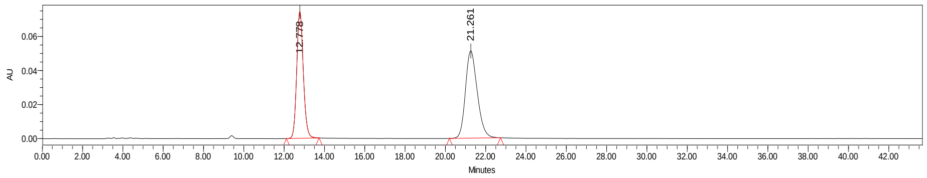

	Retention Time	Area	% Area
1	12.778	2085560	50.02
2	21.261	2083344	49.98

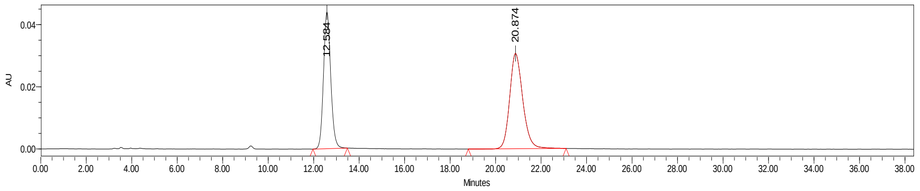

	Retention Time	Area	% Area
1	12.584	973348	44.28
2	20.874	1224872	55.72

Dimethyl (*S*)-2-{2-methyl-1-[1-(naphthalen-2-yl)-1H-tetrazol-5-yl]propyl}malonate (4i)

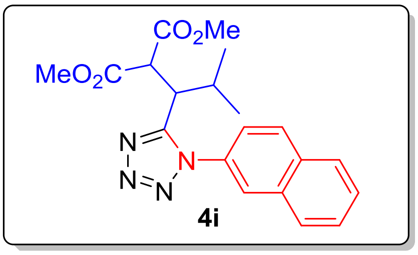

White solid; m.p. 124-126 °C; 72% yield, 96:4 e.r.; $[\alpha]^{21}_D$ = –78.0 (c = 0.47 in CH_2Cl_2).

HPLC DAICEL CHIRALCEL IC, *n*-hexane/2-propanol = 70/30, flow rate = 1.0 mL/min, λ = 254 nm, t_R (minor) = 9.06 min, t_R (major) = 11.70 min.

IR (neat): 2960, 1746, 1512, 1440, 1308, 1265, 864, 814 and 752 cm^{-1}.

^{1}H NMR (400 MHz, $CDCl_3$) δ = 8.27 (s, 1H), 8.10 – 8.02 (m, 1H), 8.02 – 7.89 (m, 2H), 7.81 – 7.74 (m, 1H), 7.71 – 7.57 (m, 2H), 4.49 (d, *J* = 11.6 Hz, 1H), 3.96 (dd, *J* = 11.6, 4.0 Hz, 1H), 3.79 (s, 3H), 3.69 (s, 3H), 2.01 – 1.79 (m, 1H), 0.93 (d, *J* = 6.8 Hz, 3H), 0.50 (d, *J* = 6.8 Hz, 3H).

^{13}C{^{1}H} NMR (101 MHz, $CDCl_3$) δ = 168.6, 168.3, 154.5, 133.7, 133.1, 131.7, 130.2, 128.8, 128.1, 128.0, 127.6, 125.5, 123.2, 54.1, 53.2, 39.6, 30.2, 21.3, 16.9.

HRMS (ESI-FT) calcd for $C_{20}H_{23}N_4O_4^+$ ([M+H$^+$]) = 383.1714, Found 383.1708.

Chiral HPLC spectrum **4i**:

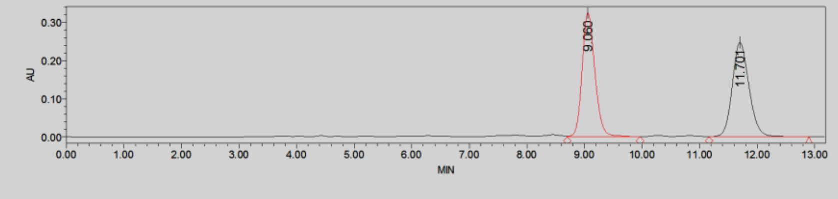

	Retention Time	Area	% Area
1	9.060	5029268	50.13
2	11.701	5003981	49.87

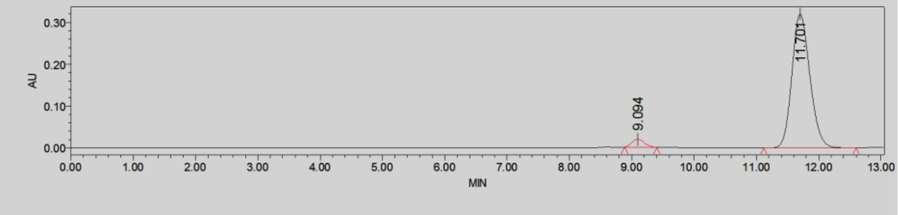

	Retention Time	Area	% Area
1	9.094	265343	3.97
2	11.701	6410010	96.03

Dibenzyl 2-{2-methyl-1-[1-(naphthalen-2-yl)-1H-tetrazol-5-yl]propyl}malonate (4j)

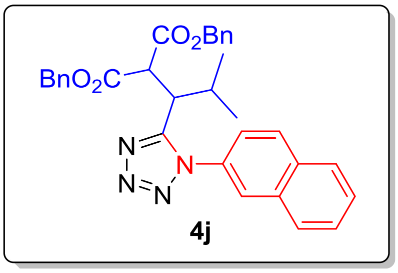

Coreless oil; 84% yield, 96:4 e.r.; $[\alpha]^{21}_{D}$ = –71.8 (*c* = 0.62 in CH_2Cl_2).

HPLC DAICEL CHIRALCEL IC, *n*-hexane/2-propanol = 70/30, flow rate = 1.0

mL/min, λ = 254 nm, t_R (minor) = 10.85 min, t_R (major) = 13.06 min.

IR (neat): 2967, 1746, 1504, 1462, 1377, 1265, 1175, 748 and 698 cm^{-1}.

^{1}H NMR (400 MHz, $CDCl_3$) δ = 8.18 (s, 1H), 8.05 – 8.00 (m, 1H), 8.00 – 7.88 (m, 2H), 7.75 – 7.68 (m, 1H), 7.68 – 7.54 (m, 2H), 7.37 – 7.13 (m, 10H), 5.18 (s, 2H), 5.10 – 4.99 (m, 2H), 4.57 (d, *J* = 11.6 Hz, 1H), 3.98 (dd, *J* = 11.6, 4.0 Hz, 1H), 1.91 – 1.75 (m, 1H), 0.88 (d, *J* = 6.8 Hz, 3H), 0.42 (d, *J* = 6.8 Hz, 3H).

^{13}C{^{1}H} NMR (101 MHz, $CDCl_3$) δ = 167.9, 167.6, 154.4, 135.0, 134.9, 133.6, 133.0, 131.6, 130.1, 128.8, 128.7, 128.7, 128.6, 128.3, 128.1,, 128.0, 127.6, 125.3, 123.2, 67.9, 67.8, 54.4, 39.6, 30.2, 21.3, 16.9.

HRMS (ESI-FT) calcd for $C_{32}H_{31}N_4O_4^+$ ([M+H$^+$]) = 535.2340, Found 535.2335.

Chiral HPLC spectrum **4j**:

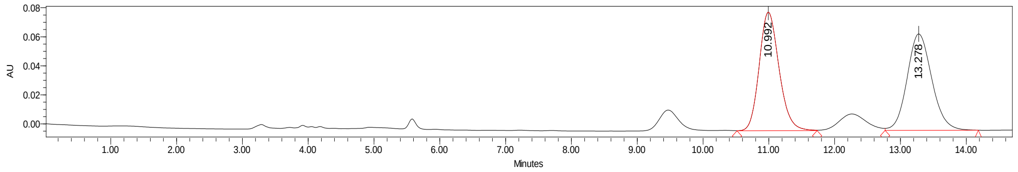

	Retention Time	Area	% Area
1	10.992	1708633	49.73
2	13.278	1727369	50.27

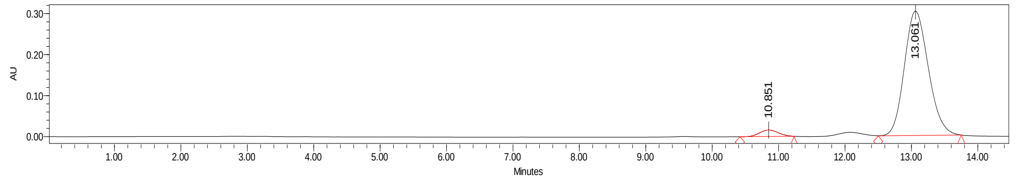

	Retention Time	Area	% Area
1	10.851	303703	3.83
2	13.061	7621541	96.17

Dimethyl 2-{1-[1-(naphthalen-2-yl)-1H-tetrazol-5-yl]ethyl}malonate (4k)

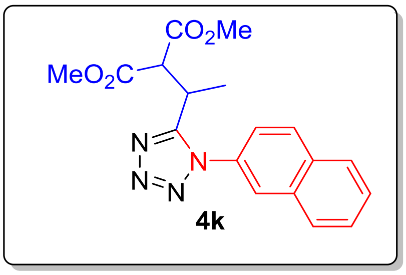

Coreless oil; 70% yield, 86.5:13.5 e.r.; $[\alpha]^{21}_D$ = –66.0 (*c* = 0.34 in CH_2Cl_2).

HPLC DAICEL CHIRALCEL IC, *n*-hexane/2-propanol = 70/30, flow rate = 1.0 mL/min, λ = 254 nm, t_R (minor) = 15.30 min, t_R (major) = 24.69 min.

IR (neat): 2961, 1744, 1510, 1439, 1246, 1036 and 825 cm^{-1}.

^{1}**H NMR** (400 MHz, $CDCl_3$) δ = 8.12 – 8.04 (m, 2H), 8.01 – 7.89 (m, 2H), 7.74 – 7.59 (m, 3H), 4.32 (d, *J* = 11.2 Hz, 1H), 3.88 – 3.82 (m, 1H), 3.77 (s, 3H), 3.66 (s, 3H), 1.23 (d, *J* = 6.8 Hz, 3H).

^{13}**C{**^{1}**H} NMR** (101 MHz, $CDCl_3$) δ = 168.1, 158.0, 133.8, 133.1, 131.0, 130.5, 128.7, 128.2, 127.8, 125.1, 122.7, 56.0, 53.2, 53.1, 29.4, 18.3.

HRMS (ESI-FT) calcd for $C_{18}H_{19}N_4O_4^+$ ([M+H$^+$]) = 355.1401, Found 355.1404.

Chiral HPLC spectrum **4k**:

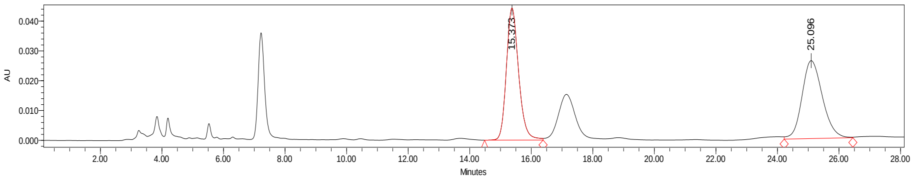

	Retention Time	Area	% Area
1	15.373	1225668	50.86
2	25.096	1184172	49.14

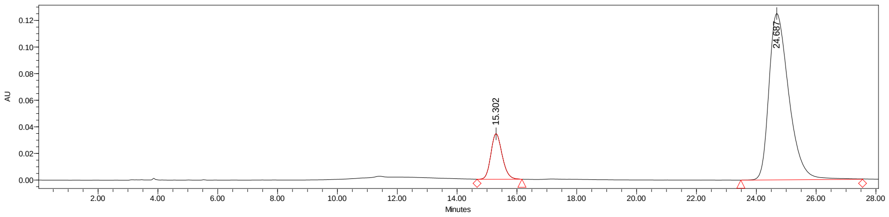

	Retention Time	Area	% Area
1	15.302	901217	13.51
2	24.687	5768336	86.49

Dibenzyl 2-{3-methyl-1-[1-(naphthalen-2-yl)-1H-tetrazol-5-yl]butyl}malonate (4l)

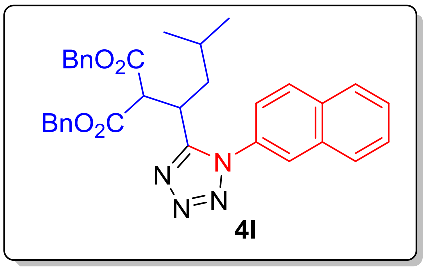

Coreless oil; 93% yield, 86:14 e.r.; $[\alpha]^{21}_{D}$ = −34.8 (*c* = 0.81 in CH_2Cl_2).

HPLC DAICEL CHIRALCEL IC, *n*-hexane/2-propanol = 70/30, flow rate = 1.0 mL/min, λ = 254 nm, t_R (minor) = 18.17 min, t_R (major) = 13.15 min.

IR (neat): 2961, 1750, 1601, 1506, 1450, 1377, 1258, 1175, 820, 746 and 698 cm^{-1}.

^{1}**H NMR** (400 MHz, $CDCl_3$) δ = 8.15 (s, 1H), 8.08 – 8.00 (m, 1H), 7.99 – 7.88 (m, 2H), 7.74 – 7.56 (m, 3H), 7.37 – 7.15 (m, 10H), 5.26 – 4.98 (m, 4H), 4.39 (d, *J* = 11.2 Hz, 1H), 3.99 – 3.84 (m, 1H), 1.76 – 1.66 (m, 1H), 1.29 – 1.18 (m, 1H), 1.00 – 0.81 (m, 1H), 0.60 (d, *J* = 6.8 Hz, 3H), 0.17 (d, *J* = 6.8 Hz, 3H).

^{13}**C{**^{1}**H} NMR** (101 MHz, $CDCl_3$) δ = 167.6, 167.4, 157.3, 135.0, 134.9, 133.6, 133.0, 131.3, 130.2, 128.8, 128.7, 128.6, 128.1, 128.1, 128.0, 127.6, 125.1, 122.9, 77.48, 67.8, 56.6, 43.1, 32.8, 25.3, 23.5, 20.7.

HRMS (ESI-FT) calcd for $C_{33}H_{33}N_4O_4^+$ ([M+H$^+$]) = 549.2496, Found 594.2502.

Chiral HPLC spectrum **4l**:

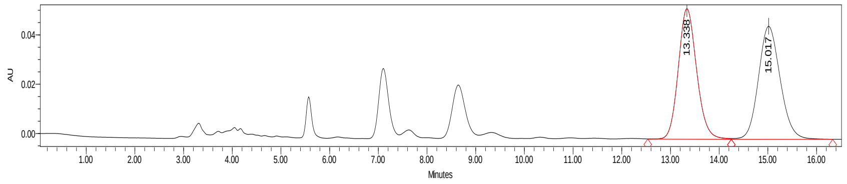

	Retention Time	Area	% Area
1	13.338	1380363	49.41
2	15.017	1413330	50.59

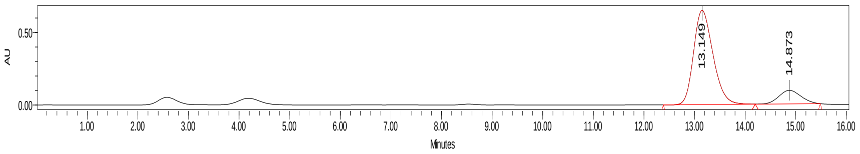

	Retention Time	Area	% Area
1	13.149	17340974	85.83
2	14.873	2862307	14.17

Dibenzyl 2-{1-[1-(naphthalen-2-yl)-1H-tetrazol-5-yl]pentyl}malonate (4m)

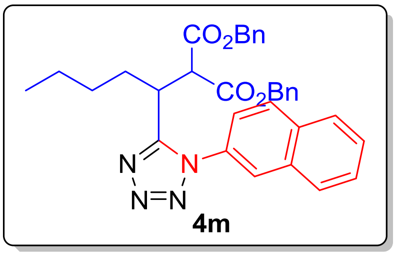

Coreless oil; 56% yield, 88.5:11.5 e.r.; $[\alpha]^{21}_{D}$ = –38.8 (*c* = 0.45 in CH_2Cl_2).

HPLC DAICEL CHIRALCEL IA, *n*-hexane/2-propanol = 70/30, flow rate = 1.0 mL/min, λ = 254 nm, t_R (minor) = 6.36 min, t_R (major) = 8.32 min.

IR (neat): 2957, 1746, 1504, 1452, 1377, 1174, 746 and 698 cm^{-1}.

^{1}H NMR (400 MHz, $CDCl_3$) δ = 8.08 (s, 1H), 8.06 – 8.00 (m, 1H), 7.95 (dd, *J* = 17.2, 7.6 Hz, 2H), 7.71 – 7.57 (m, 3H), 7.38 – 7.14 (m, 10H), 5.19 (d, *J* = 12.8 Hz, 2H), 5.11 – 4.99 (m, 2H), 4.44 (d, *J* = 11.2 Hz, 1H), 3.93 – 3.77 (m, 3.8 Hz, 1H), 1.73 – 1.58 (m, 1H), 1.50 – 1.40 (m, 1H), 0.99 – 0.67 (m, 4H), 0.61 (t, *J* = 6.8 Hz, 3H).

^{13}C{^{1}H} NMR (101 MHz, $CDCl_3$) δ = 167.6, 167.5, 156.9, 135.0, 134.9, 133.7, 133.0, 131.2, 130.2, 128.8, 128.7, 128.6, 128.5, 128.1, 127.7, 125.3, 123.0, 67.8, 55.8, 34.3, 32.7, 28.2, 22.2, 13.6.

HRMS (ESI-FT) calcd for $C_{33}H_{33}N_4O_4^+$ ([M+H$^+$]) = 549.2496, Found 549.2494.

Chiral HPLC spectrum **4m**:

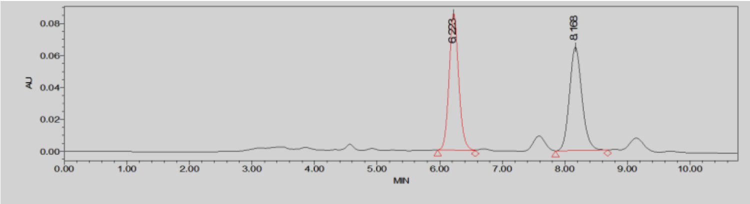

	Retention Time	Area	% Area
1	6.223	910227	50.37
2	8.168	896918	49.63

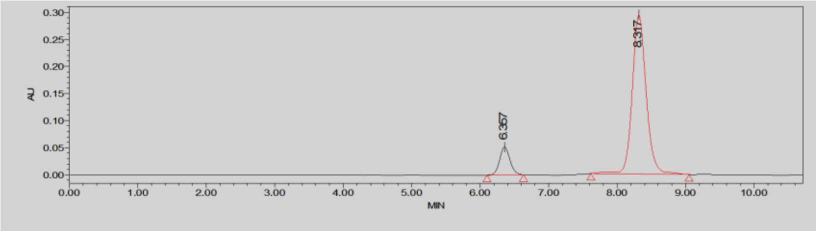

	Retention Time	Area	% Area
1	6.357	539083	11.48
2	8.317	4154746	88.52

Dimethyl 2-{cyclohexyl[1-(4-nitrophenyl)-1H-tetrazol-5-yl]methyl}malonate (4n)

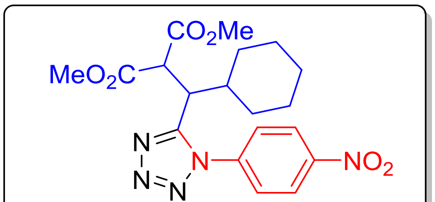

4n

White solid; m.p. 92-96 °C; 50% yield, 90.5:9.5 e.r.; $[\alpha]^{21}_{D}$ = –97.0 (c = 0.27 in CH_2Cl_2).

HPLC DAICEL CHIRALCEL IC, *n*-hexane/2-propanol = 70/30, flow rate = 1.0 mL/min, λ = 254 nm, t_R (minor) = 14.14 min, t_R (major) = 15.73 min.

IR (neat): 2930, 1744, 1605, 1531, 1443, 1346 and 860 cm^{-1}.

^{1}H NMR (400 MHz, $CDCl_3$) δ = 8.61 – 8.34 (m, 2H), 8.18 – 7.86 (m, 2H), 4.49 (dd, J = 11.6, 1.6 Hz, 1H), 3.95 – 3.74 (m, 4H), 3.65 (s, 3H), 1.75 (dd, J = 26.0, 12.8 Hz, 2H), 1.59 – 1.47 (m, 3H), 1.14 – 0.80 (m, 5H), 0.44 – 0.27 (m, 1H).

^{13}C{^{1}H} NMR (101 MHz, $CDCl_3$) δ = 168.9, 168.1, 155.1, 148.7, 139.3, 127.2, 125.4, 53.4, 40.4, 39.6, 31.9, 27.3, 26.3, 26.0, 25.7.

HRMS (ESI-FT) calcd for $C_{19}H_{24}N_5O_6^+$ ([M+H$^+$]) = 418.1721, Found 418.1719.

Chiral HPLC spectrum **4n**:

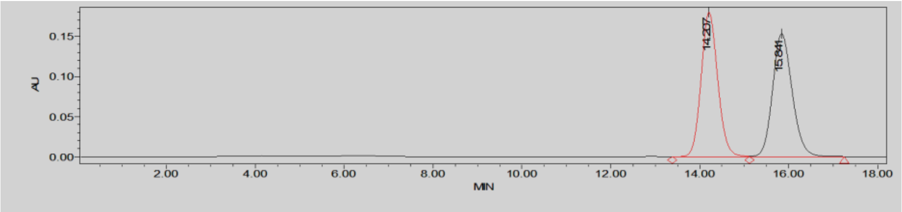

	Retention Time	Area	% Area
1	14.207	4694704	49.89
2	15.841	4714854	50.11

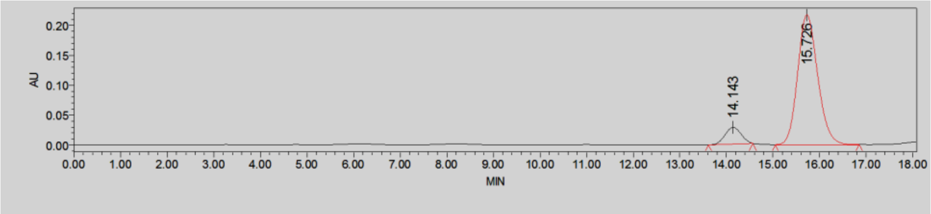

	Retention Time	Area	% Area
1	14.143	668070	9.34
2	15.726	6482836	90.66

Dimethyl 2-{[1-(naphthalen-2-yl)-1H-tetrazol-5-yl](phenyl)methyl}malonate (4o)

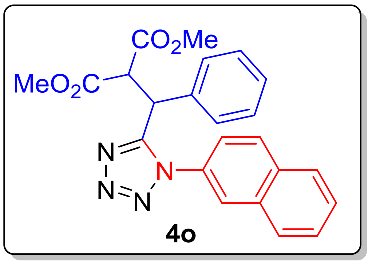

Coreless oil; 50% yield, 90.5:9.5 e.r.; $[\alpha]^{26}_{D}$ = –25.6 (c = 0.75 in CH_2Cl_2).

HPLC DAICEL CHIRALCEL IA, *n*-hexane/2-propanol = 80/20, flow rate = 1.0 mL/min, λ = 254 nm, t_R (minor) = 7.70 min, t_R (major) = 7.77 min.

IR (neat): 1746, 1597,1440, 1307, 1261, 1184, 864,820 and 750 cm^{-1}.

^{1}H NMR (400 MHz, $CDCl_3$) δ = 8.02 – 7.93 (m, 2H), 7.86 – 7.81 (m, J = 7.7, 1H), 7.78 – 7.74 (m, 1H), 7.67 – 7.58 (m, 2H), 7.40 (dd, J = 8.8, 2.4 Hz, 1H), 7.27 – 7.22 (m, 3H), 7.19 – 7.13 (m, 2H), 4.88 – 4.82 (m, 1H), 4.79 – 4.70 (m, 1H), 3.71 (s, 3H), 3.42 (s, 3H).

^{13}C{^{1}H} NMR (101 MHz, $CDCl_3$) δ = 167.9, 167.2, 155.9, 134.7, 133.8, 132.9, 130.7, 130.2, 129.1, 128.8, 128.7, 128.6, 128.1, 127.7, 125.2, 122.8, 57.0, 53.3,, 52.8, 40.8.

HRMS (ESI-FT) calcd for $C_{23}H_{21}N_4O_4^+$ ($[M+H^+]$) = 417.1557, Found 417.1551.

Chiral HPLC spectrum **4o**:

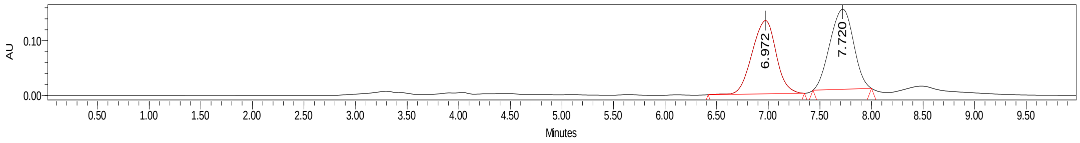

	Retention Time	Area	% Area
1	6.972	2165995	48.25
2	7.720	2322819	51.75

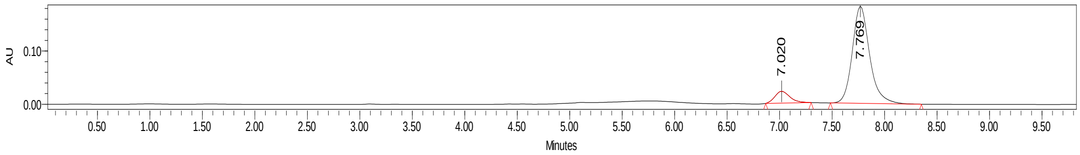

	Retention Time	Area	% Area
1	7.020	218983	9.46
2	7.769	2096223	90.54

Dimethyl 2-{[1-(naphthalen-2-yl)-1H-tetrazol-5-yl](p-tolyl)methyl}malonate (4p)

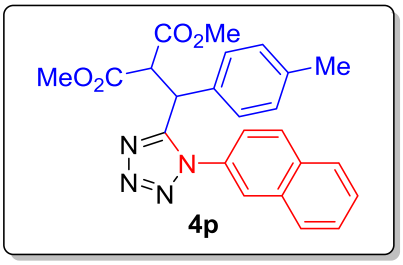

Coreless oil; 51% yield, 90.5:9.5 e.r.; $[\alpha]^{26}_{D}$ = +63.7 (*c* = 0.35 in CH_2Cl_2, λ = 405 nm).

HPLC DAICEL CHIRALCEL IA, *n*-hexane/2-propanol = 80/20, flow rate = 1.0 mL/min, λ = 254 nm, t_R (minor) = 7.21 min, t_R (major) = 7.90 min.

IR (neat): 1746, 1516, 1439, 1305, 1261, 1163, 812 and 750 cm^{-1}.

^{1}H NMR (400 MHz, $CDCl_3$) δ = 8.03 – 7.93 (m, 2H), 7.87 – 7.83 (m, 1H), 7.80 – 7.78 (m, 1H), 7.69 – 7.56 (m, 2H), 7.42 (dd, *J* = 8.4, 2.0 Hz, 1H), 7.05 (s, 4H), 4.84 – 4.78 (m, 1H), 4.78 – 4.69 (m, 1H), 3.70 (s, 3H), 3.44 (s, 3H), 2.29 (s, 3H).

^{13}C{^{1}H} NMR (101 MHz, $CDCl_3$) δ = 168.0, 167.3, 156.1, 138.6, 133.8, 132.9, 131.6, 130.8, 130.2, 129.9, 128.6, 128.2, 128.1, 127.7, 125.2, 122.9, 57.1, 53.3, 53.2, 52.8, 40.5, 21.2.

HRMS (ESI-FT) calcd for $C_{24}H_{23}N_4O_4^+$ ($[M+H^+]$) = 431.1714, Found 431.1709.

Chiral HPLC spectrum **4p**:

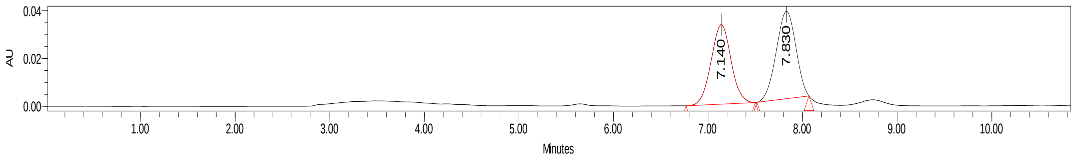

	Retention Time	Area	% Area
1	7.140	489335	49.01
2	7.830	509035	50.99

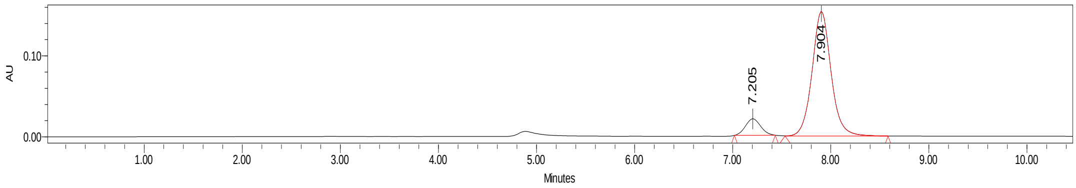

	Retention Time	Area	% Area
1	7.205	219193	9.57
2	7.904	2070546	90.43

Dimethyl 2-{[1-(naphthalen-2-yl)-1H-tetrazol-5-yl](m-tolyl)methyl}malonate (4q)

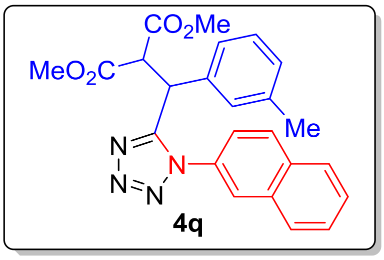

Coreless oil; 45% yield, 89:11 e.r.; $[\alpha]^{26}_{D}$ = –24.0 (*c* = 0.34 in CH_2Cl_2).

HPLC DAICEL CHIRALCEL IA, *n*-hexane/2-propanol = 80/20, flow rate = 1.0 mL/min, λ = 254 nm, t_R (minor) = 6.28 min, t_R (major) = 6.52 min.

IR (neat): 1746, 1439, 1306, 1261, 1161 and 822 cm^{-1}.

^{1}**H NMR** (400 MHz, $CDCl_3$) δ = 8.02 – 7.95 (m, 2H), 7.86 – 7.81 (m, 1H), 7.76 – 7.73 (m, 1H), 7.68 – 7.58 (m, 2H), 7.41 (dd, *J* = 8.4, 2.0 Hz, 1H), 7.16 – 7.04 (m, 2H), 6.99 – 6.96 (m, 1H), 6.94 – 6.89 (m, 1H), 4.82 – 4.77 (m, 1H), 4.77 – 4.71 (m, 1H), 3.70 (s, 3H), 3.43 (s, 3H), 2.23 (s, 3H).

^{13}**C{^{1}H} NMR** (101 MHz, $CDCl_3$) δ = 168.0, 167.3, 156.0, 139.0, 134.6, 133.8, 132.9, 130.8, 130.1, 129.5, 129.3, 129.0, 128.6, 128.2, 128.1, 127.7, 125.9, 125.3, 122.9, 57.01, 53.3, 52.8, 40.8, 21.4.

HRMS (ESI-FT) calcd for $C_{24}H_{23}N_4O_4^+$ ([M+H$^+$]) = 431.1714, Found 431.1707.

Chiral HPLC spectrum **4q**:

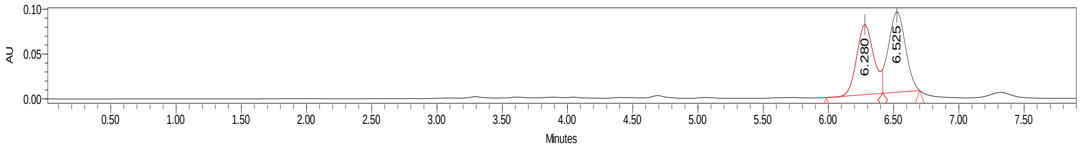

	Retention Time	Area	% Area
1	6.280	716729	47.16
2	6.525	803060	52.84

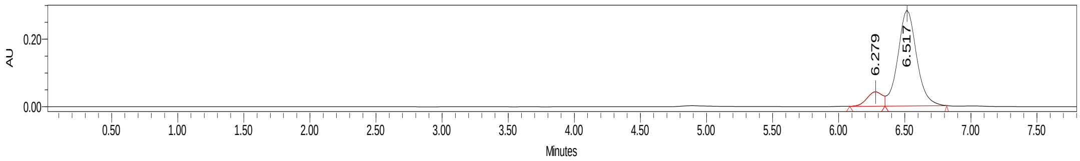

	Retention Time	Area	% Area
1	6.279	349428	11.12
2	6.517	2794153	88.88

Dimethyl 2-{(4-bromophenyl)[1-(naphthalen-2-yl)-1H-tetrazol-5-yl]methyl}malonate (4r)

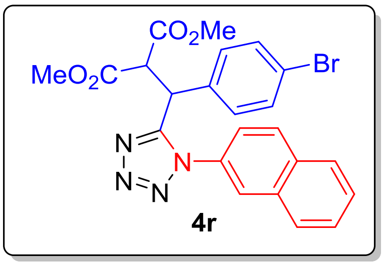

Coreless oil; 61% yield, 92.5:7.5 e.r.; $[\alpha]^{26}_{D}$ = +63.3 (c = 0.51 in CH_2Cl_2).

HPLC DAICEL CHIRALCEL IA, *n*-hexane/2-propanol = 80/20, flow rate = 1.0 mL/min, λ = 254 nm, t_R (minor) = 7.69 min, t_R (major) = 8.33 min.

IR (neat): 1746, 1483, 1439, 1306, 1258, 1184, 1018, 860, 812 and 750 cm^{-1}.

^{1}H NMR (400 MHz, $CDCl_3$) δ = 8.04 – 7.96 (m, 2H), 7.90 – 7.85 (m, 1H), 7.81 – 7.78 (m, 1H), 7.70 – 7.60 (m, 2H), 7.43 – 7.35 (m, 3H), 7.07 – 7.01 (m, 2H), 4.85 – 4.80 (m, 1H), 4.76 – 4.68 (m, 1H), 3.71 (s, 3H), 3.47 (s, 3H).

^{13}C{^{1}H} NMR (101 MHz, $CDCl_3$) δ = 167.7, 167.1, 155.6, 133.9, 133.7, 132.9, 132.4, 130.6, 130.4, 130.3, 128.6, 128.3, 128.2, 127.9, 125.3, 123.0, 122.7, 56.8, 53.4, 53.0, 40.2.

HRMS (ESI-FT) calcd for $C_{23}H_{20}{}^{79}BrN_4O_4{}^+$ ([M+H$^+$]) = 495.0062, Found 495.0658; $C_{23}H_{20}{}^{81}BrN_4O_4{}^+$ ([M+H$^+$]) = 497.0642, Found 497.0638.

Chiral HPLC spectrum **4r**:

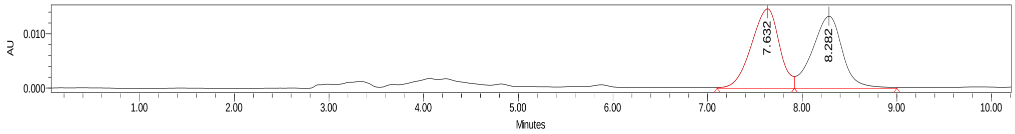

	Retention Time	Area	% Area
1	7.632	301717	50.73
2	8.282	293056	49.27

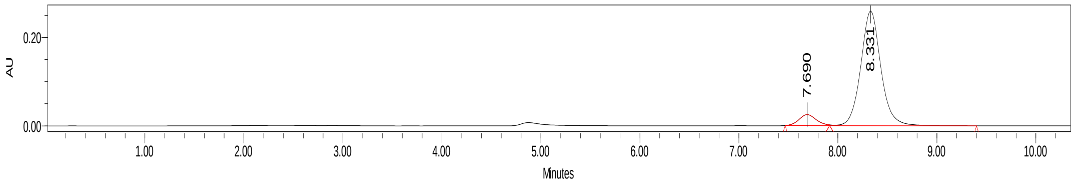

	Retention Time	Area	% Area
1	7.690	298305	7.56
2	8.331	3645054	92.44

Dimethyl 2-{(4-chlorophenyl)[1-(naphthalen-2-yl)-1H-tetrazol-5-yl]methyl}malonate (4s)

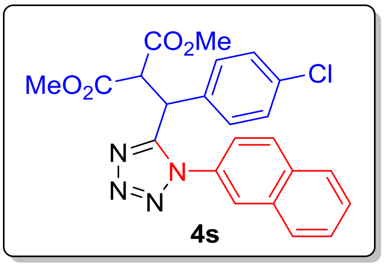

Coreless oil; 56% yield, 91.5:8.5 e.r.; $[\alpha]^{26}_{D}$ = +27.5 (*c* = 0.35 in CH_2Cl_2).

HPLC DAICEL CHIRALCEL IA, *n*-hexane/2-propanol = 70/30, flow rate = 1.0 mL/min, λ = 254 nm, t_R (minor) = 6.18 min, t_R (major) = 6.62 min.

IR (neat): 1746, 1483, 1439, 1306, 1258, 1184, 812 and 750 cm^{-1}.

^{1}H NMR (400 MHz, $CDCl_3$) δ = 8.05 – 7.95 (m, 2H), 7.91 – 7.85 (m, 1H), 7.79 (d, *J* = 2.0 Hz, 1H), 7.70 – 7.60 (m, 2H), 7.40 (dd, *J* = 8.8, 2.0 Hz, 1H), 7.25 – 7.19 (m, 2H), 7.16 – 7.05 (m, 2H), 4.86 – 4.80 (m, 1H), 4.76 – 4.68 (m, 1H), 3.71 (s, 3H), 3.47 (s, 3H).

^{13}C{^{1}H} NMR (101 MHz, $CDCl_3$) δ = 167.7, 167.1, 155.7, 134.9, 133.9, 133.2, 132.9, 130.6, 130.4, 130.1, 129.4, 128.6, 128.3, 128.2, 127.9, 125.3, 122.7, 56.9, 53.4, 53.4, 53.0, 40.1.

HRMS (ESI-FT) calcd for $C_{23}H_{20}{}^{35}ClN_4O_4^+$ ([M+H$^+$]) = 451.1168, Found 451.1168; $C_{23}H_{20}{}^{37}ClN_4O_4^+$ ([M+H$^+$]) = 453.1138, Found 453.1139.

Chiral HPLC spectrum **4s**:

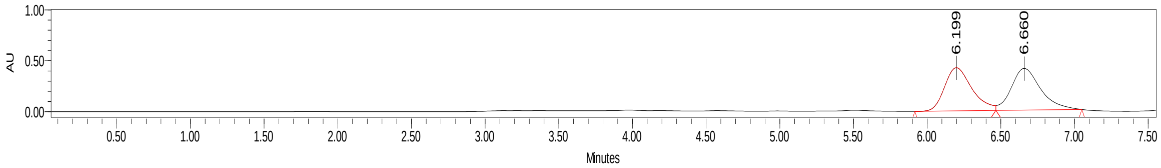

	Retention Time	% Area	% Height
1	6.199	48.99	50.85
2	6.660	51.01	49.15

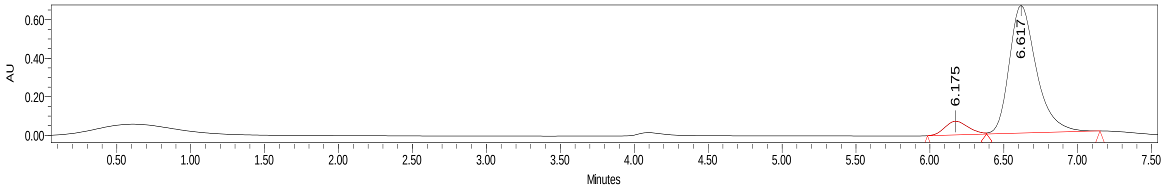

	Retention Time	% Area	% Height
1	6.175	8.49	9.68
2	6.617	91.51	90.32

Dimethyl 2-{(3-chlorophenyl)[1-(naphthalen-2-yl)-1H-tetrazol-5-yl]methyl}malonate (4t)

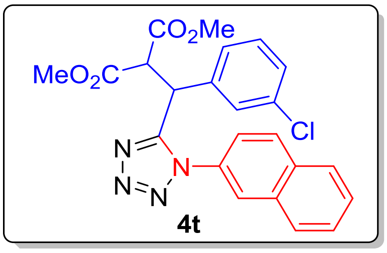

Coreless oil; 40% yield, 88.5:11.5 e.r.; $[\alpha]^{26}_{D}$ = –11.1 (c = 0.25 in CH_2Cl_2).

HPLC DAICEL CHIRALCEL IC, *n*-hexane/2-propanol = 70/30, flow rate = 1.0 mL/min, λ = 254 nm, t_R (minor) = 9.02 min, t_R (major) = 14.87 min.

IR (neat): 1748, 1439, 1302, 1260, 1186, 1161, 820, 754 and 696 cm^{-1}.

^{1}H NMR (400 MHz, $CDCl_3$) δ = 8.06 – 7.94 (m, 2H), 7.90 – 7.86 (m, 1H), 7.80 – 7.77 (m, 1H), 7.70 – 7.60 (m, 2H), 7.40 (dd, J = 8.4, 2.0 Hz, 1H), 7.28 – 7.24 (m, 1H), 7.22 – 7.16 (m, 2H), 7.10 – 7.04 (m, 1H), 4.86 – 4.78 (m, 1H), 4.77 – 4.67 (m, 1H), 3.71 (s, 3H), 3.47 (s, 3H).

^{13}C{^{1}H} NMR (101 MHz, $CDCl_3$) δ = 167.7, 167.0, 155.5, 136.7, 135.1, 133.9, 132.9, 130.6, 130.4, 129.1, 129.0, 128.6, 128.3, 128.2, 127.9, 127.0, 125.3, 122.7, 56.9, 53.4, 53.0, 40.4.

HRMS (ESI-FT) calcd for $C_{23}H_{20}{}^{35}ClN_4O_4^+$ ([M+H$^+$]) = 451.1168, Found 451.1161; $C_{23}H_{20}{}^{37}ClN_4O_4^+$ ([M+H$^+$]) = 453.1138, Found 453.1138.

Chiral HPLC spectrum **4t**:

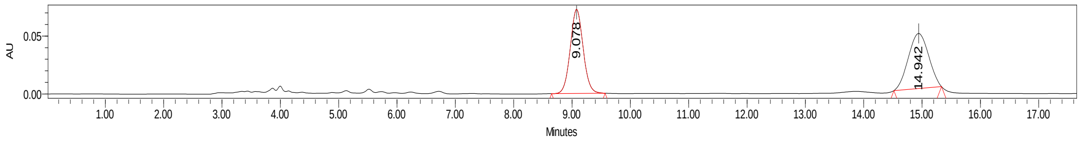

	Retention Time	Area	% Area
1	9.078	1111542	49.29
2	14.942	1143696	50.71

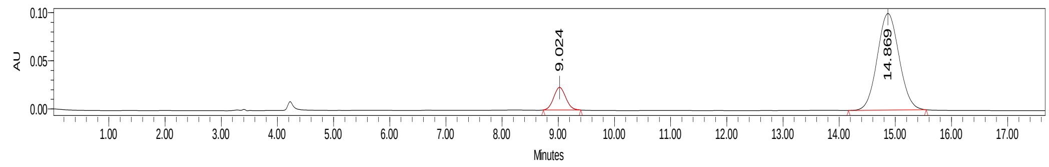

	Retention Time	Area	% Area
1	9.024	350564	11.48
2	14.869	2702253	88.52

Dimethyl 2-{(2-chlorophenyl)[1-(naphthalen-2-yl)-1H-tetrazol-5-yl]methyl}malonate (4u)

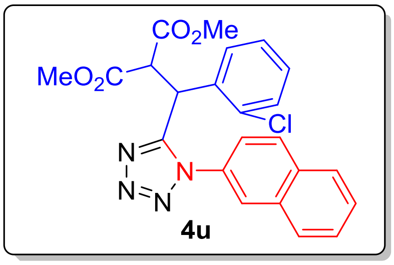

Coreless oil; 33% yield, 77:23 e.r.; $[\alpha]^{26}_{D}$ = –55.7 (c = 0.34 in CH_2Cl_2).

HPLC DAICEL CHIRALCEL IA, *n*-hexane/2-propanol = 80/20, flow rate = 1.0 mL/min, λ = 254 nm, t_R (minor) = 8.16 min, t_R (major) = 9.53 min.

IR (neat): 1746, 1439, 1303, 1250, 1184, 1163, 1036, 862, 820 and 756 cm^{-1}.

^{1}H NMR (400 MHz, $CDCl_3$) δ = 8.03 – 7.92 (m, 2H), 7.90 – 7.85 (m, 1H), 7.83 – 7.79 (m, 1H), 7.67 – 7.57 (m, 3H), 7.43 (dd, *J* = 8.8, 2.4 Hz, 1H), 7.28 – 7.13 (m, 3H), 5.60 (d, *J* = 12.0 Hz, 1H), 4.75 (d, *J* = 11.8 Hz, 1H), 3.74 (s, 3H), 3.45 (s, 3H).

^{13}C{^{1}H} NMR (101 MHz, $CDCl_3$) δ = 167.9, 166.6, 156.1, 133.9, 133.8, 133.0, 132.5, 130.8, 130.6, 130.2, 130.0, 129.9, 128.7, 128.1, 127.9, 127.6, 125.5, 122.9, 56.8, 53.3, 52.8, 36.0.

HRMS (ESI-FT) calcd for $C_{23}H_{20}{}^{35}ClN_4O_4^+$ ([M+H$^+$]) = 451.1168, Found 451.1163; $C_{23}H_{20}{}^{37}ClN_4O_4^+$ ([M+H$^+$]) = 453.1138, Found 453.1140.

Chiral HPLC spectrum **4u**:

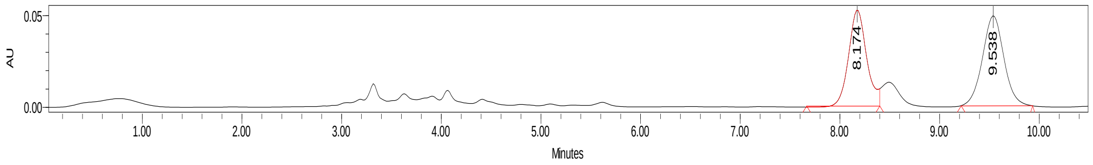

	Retention Time	Area	% Area
1	8.174	661933	47.43
2	9.538	733629	52.57

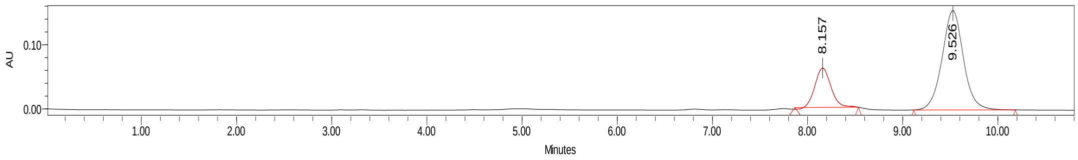

	Retention Time	Area	% Area
1	8.157	726705	23.16
2	9.526	2411451	76.84

Dimethyl 2-{naphthalen-2-yl[1-(naphthalen-2-yl)-1H-tetrazol-5-yl]methyl}malonate (4v)

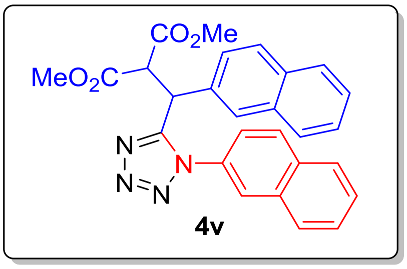

Coreless oil; 39% yield, 88:12 e.r.; $[\alpha]^{26}_{D}$ = +73.0 (c = 0.22 in CH_2Cl_2).

HPLC DAICEL CHIRALCEL IA, *n*-hexane/2-propanol = 80/20, flow rate = 1.0 mL/min, λ = 254 nm, t_R (minor) = 8.71 min, t_R (major) = 9.96 min.

IR (neat): 1746, 1439, 1312, 1265, 1161, 816 and 748 cm^{-1}.

^{1}H NMR (400 MHz, $CDCl_3$) δ = 8.02 – 7.94 (m, 2H), 7.82 – 7.73 (m, 4H), 7.70 – 7.57 (m, 4H), 7.72 – 7.44 (m, 2H), 7.39 (dd, J = 8.8, 2.0 Hz, 1H), 7.33 (dd, J = 8.8, 2.0 Hz, 1H), 5.05 – 4.97 (m, 1H), 4.92 – 4.81 (m, 1H), 3.73 (s, 3H), 3.36 (s, 3H).

^{13}C{^{1}H} NMR (101 MHz, $CDCl_3$) δ = 167.9, 167.3, 156.0, 133.9, 133.3, 133.1, 132.9, 132.1, 130.8, 130.2, 129.1, 128.6, 128.4, 128.2, 128.1, 127.8, 127.8, 126.9, 126.8, 125.8, 125.4, 122.9, 57.1, 53.4, 52.9, 41.0.

HRMS (ESI-FT) calcd for $C_{27}H_{23}N_4O_4^+$ ([M+H$^+$]) = 467.1714, Found 467.1709.

Chiral HPLC spectrum **4v**:

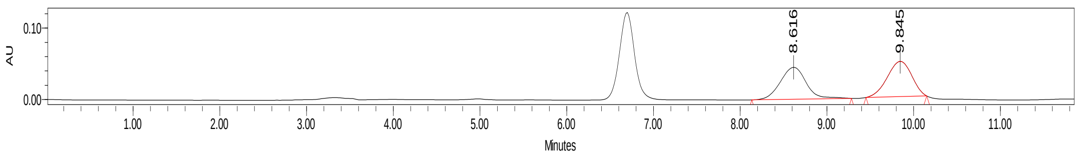

	Retention Time	Area	% Area
1	8.616	925951	49.02
2	9.845	963032	50.98

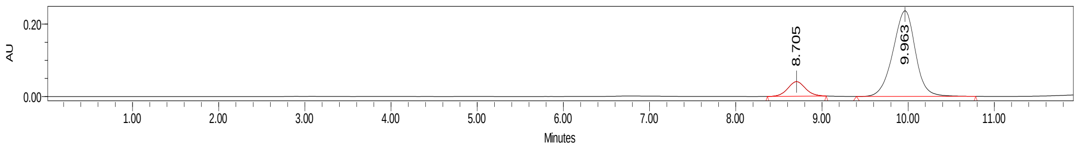

	Retention Time	Area	% Area
1	8.705	586045	12.08
2	9.963	4265645	87.92

Dimethyl 2-{(1*S*,3*S*)-3,7-dimethyl-1-[1-(naphthalen-2-yl)-1H-tetrazol-5-yl]oct-6-en-1-yl} malonate (4w)

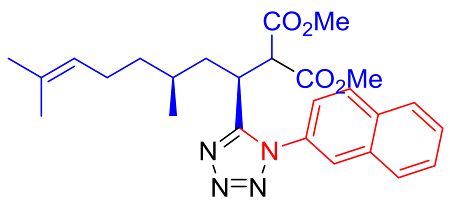

4w

Coreless oil; 47% yield, 2.8:1 d.r.; $[\alpha]^{25}_{D}$ = –59.51 (*c* = 0.37 in CH_2Cl_2).

IR (neat): 1752, 1737, 1437, 1274, 1254, 1155, 1104, 818 and 750 cm^{-1}.

^{1}H NMR (400 MHz, $CDCl_3$) δ = 8.28 – 8.20 (m, 1H), 8.10 – 8.04 (m, 1H), 8.01 – 7.92 (m, 2H), 7.81 (dd, *J* = 8.8, 2.2 Hz, 0.74H), 7.75 (dd, *J* = 8.8, 2.2 Hz, 0.29H), 7.69 – 7.58 (m, 2H), 4.82 (td, *J* = 7.0, 3.4 Hz, 0.26H), 4.35 – 4.28 (m, 1H), 4.23 (td, *J* = 7.0, 6.2, 3.4 Hz, 1H), 4.05 – 3.90 (m, 1H), 3.78 (s, 3H), 3.67 (s, 3H), 1.87 – 1.60 (m, 4H), 1.53 – 1.40 (m, 3H), 1.30 (s, 2H), 1.23 – 1.13 (m, 1H), 0.88 – 0.71 (m, 1H), 0.62 (d, *J* = 6.4 Hz, 2H), 0.24 (d, *J* = 6.4 Hz, 0.78H).

^{13}C{^{1}H} NMR (101 MHz, $CDCl_3$) δ = 168.4, 168.2, 157.4, 157.3, 133.7, 133.1, 131.8, 131.4, 131.2, 130.4, 130.3, 128.8, 128.2, 128.1, 127.7, 125.3, 124.9, 124.0, 123.9,

123.0, 122.7, 56.3, 53.2, 41.9, 41.4, 37.6, 35.4, 32.5, 30.4, 29.6, 25.8, 25.6, 25.1, 25.0, 20.2, 18.4, 17.8, 17.4.

HRMS (ESI-FT) calcd for $C_{26}H_{33}N_4O_4^+$ ([M+H$^+$]) = 465.2496, Found 465.2496.

Dimethyl 2-{1-cyclohexyl-2-[1-(naphthalen-2-yl)-1H-tetrazol-5-yl]-2-(naphthalen-2-ylimino) ethyl}malonate (5a)

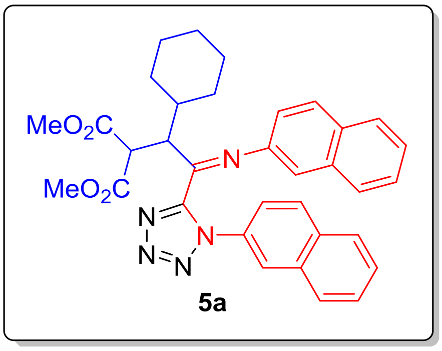

Yellow solid; m.p. 54-56 °C; 91% yield, 94.5:5.5 e.r.; $[\alpha]^{21}_{D}$ = –158.8 (*c* = 0.67 in CH_2Cl_2).

HPLC DAICEL CHIRALCEL IC, *n*-hexane/2-propanol = 70/30, flow rate = 1.0 mL/min, λ = 254 nm, t_R (minor) = 9.13 min, t_R (major) = 11.64 min.

IR (neat): 2930, 2855, 1738, 1630, 1510, 1439, 1265, 1155, 856, 814 and 745 cm^{-1}.

^{1}H NMR (400 MHz, $CDCl_3$) δ = 7.70 – 7.69(m, 1H), 7.57 – 7.46 (m, 4H), 7.36 – 7.27 (m, 3H), 7.26 – 7.20 (m, 1H), 7.03 (dd, *J* = 18.4, 8.4 Hz, 2H), 6.73 (d, *J* = 8.8 Hz, 1H), 6.27 (s, 1H), 6.07 (d, *J* = 8.6 Hz, 1H), 4.35 (d, *J* = 8.8 Hz, 1H), 4.13 (dd, *J* = 8.8, 4.8 Hz, 1H), 3.79 (s, 3H), 3.72 (s, 3H), 2.21 (s, 1H), 2.06 – 1.85 (m, 4H), 1.78 – 1.72 (m, 1H), 1.41 – 1.19 (m, 5H).

^{13}C{^{1}H} NMR (101 MHz, $CDCl_3$) δ = 169.8, 169.3, 156.7, 150.1, 145.7, 133.3, 133.2, 132.6, 131.0, 129.1, 128.5, 128.3, 127.7, 127.6, 127.4, 127.4, 127.2, 126.4, 125.5, 122.9, 121.0, 119.3, 115.9, 53.3, 53.0, 52.8, 52.5, 40.0, 31.3, 29.3, 27.0, 26.9, 26.4.

HRMS (ESI-FT) calcd for $C_{34}H_{34}N_5O_4^+$ ([M+H$^+$]) = 576.2605, Found 576.2604.

Chiral HPLC spectrum **5a**:

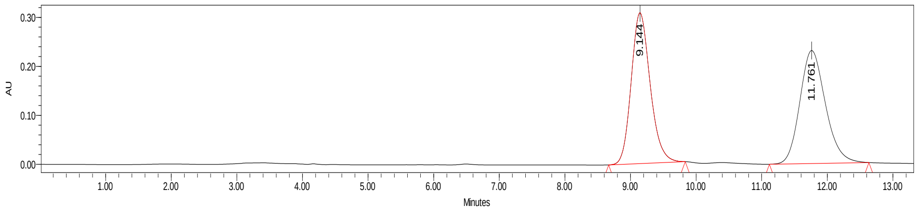

	Retention Time	Area	% Area
1	9.144	6183501	49.78
2	11.761	6238907	50.22

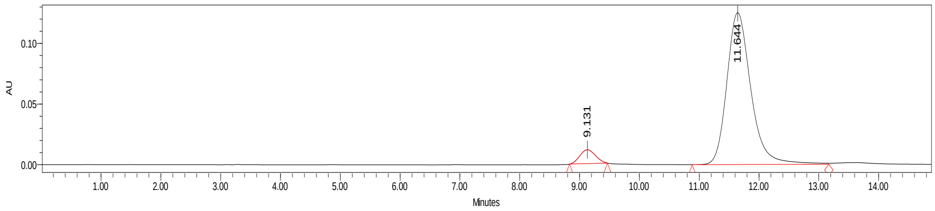

	Retention Time	Area	% Area
1	9.131	206953	5.59
2	11.644	3495720	94.41

Dimethyl 2-{1-cycloheptyl-2-[1-(naphthalen-2-yl)-1H-tetrazol-5-yl]-2-(naphthalen-2-ylimino) ethyl}malonate (5b)

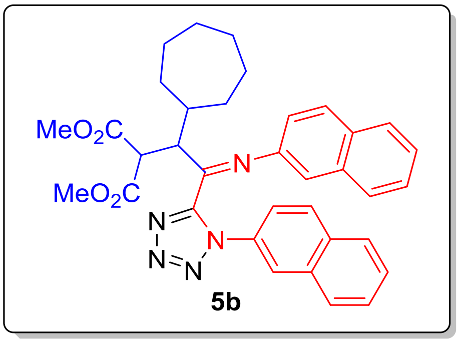

Yellow solid; m.p. 100-102 °C; 84% yield, 93.5:6.5 e.r.; $[\alpha]^{21}_D$ = –184.3 (*c* = 0.78 in CH_2Cl_2).

HPLC DAICEL CHIRALCEL IC, *n*-hexane/2-propanol = 70/30, flow rate = 1.0 mL/min, λ = 254 nm, t_R (minor) = 8.64 min, t_R (major) = 12.34 min.

IR (neat): 2926, 1738, 1510, 1439, 1265, 1152, 1028, 858, 814 and 746 cm^{-1}.

^{1}H NMR (400 MHz, $CDCl_3$) δ = 7.74 – 7.70 (m, 1H), 7.57 – 7.44 (m, 4H), 7.39 – 7.27 (m, 3H), 7.12 – 7.00 (m, 1H), 7.10 – 7.01 (m, 2H), 6.28 (s, 1H), 6.08 (d, *J* = 8.2 Hz, 1H), 4.30 – 4.40 (m, 1H), 4.28– 4.16 (m, 1H), 3.80 (s, 3H), 3.69 (s, 3H), 2.40 (s, 1H), 2.00 (s, 2H), 1.82 (s, 2H), 1.69 – 1.45 (m, 8H).

^{13}C{^{1}H} NMR (101 MHz, $CDCl_3$) δ = 169.6, 169.3, 156.8, 149.9, 145.5, 133.3, 133.2, 132.6, 130.9, 130.9, 129.2, 128.5, 128.3, 127.7, 127.6, 127.5, 127.4, 127.3, 126.4, 125.5, 122.9, 121.0, 119.3, 116.0, 54.2, 52.9, 52.8, 52.6, 41.2, 32.4, 30.1, 27.7, 27.6, 27.5, 27.5.

HRMS (ESI-FT) calcd for $C_{35}H_{36}N_5O_4^+$ ([M+H$^+$]) = 590.2762, Found 590.2756.

Chiral HPLC spectrum **5b**:

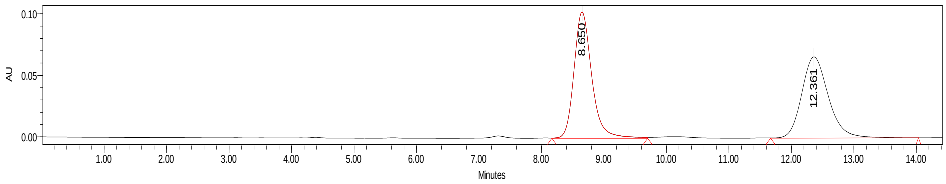

	Retention Time	Area	% Area
1	8.650	2025687	50.90
2	12.361	1953803	49.10

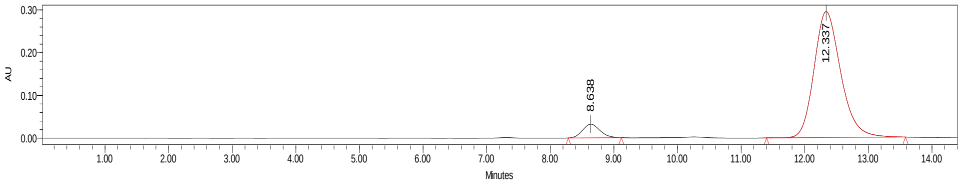

	Retention Time	Area	% Area
1	8.638	605803	6.60
2	12.337	8566409	93.40

Dimethyl 2-{1-cyclopentyl-2-[1-(naphthalen-2-yl)-1H-tetrazol-5-yl]-2-(naphthalen-2-ylimino) ethyl}malonate (5c)

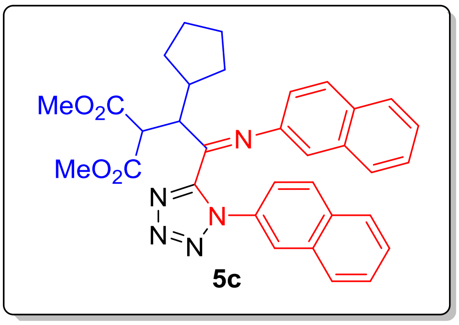

Yellow solid; m.p. 52-54 °C; 98% yield, 90:10 e.r.; $[\alpha]^{21}_D$ = +10.0 (*c* = 1.02 in CH_2Cl_2).

HPLC DAICEL CHIRALCEL IE, *n*-hexane/2-propanol = 70/30, flow rate = 1.0

mL/min, λ = 254 nm, t_R (minor) = 11.00 min, t_R (major) = 11.48 min.

IR (neat): 2955, 1740, 1510, 1439, 1267, 1157, 858, 816 and 746 cm^{-1}.

^{1}H NMR (400 MHz, $CDCl_3$) δ = 8.12 (s, 0.24H), 7.94 – 7.84 (m, 1H), 7.75 – 7.62 (m, 2.52H), 7.58 – 7.12 (m, 9.26H), 7.01 (d, *J* = 8.0 Hz, 1H), 6.94 (d, *J* = 8.4 Hz, 1H), 6.78 (d, *J* = 8.4 Hz, 1H), 6.25 (s, 1H), 6.05 (d, *J* = 8.8 Hz, 1H), 4.59 (d, *J* = 9.6 Hz, 0.22H), 4.20 (d, *J* = 4.4 Hz, 1H), 4.00 (dd, *J* = 9.6, 4.4 Hz, 1.25H), 3.80 (s, 3H), 3.72 (m, 3.76 – 3.67, 1.32H), 2.84 – 2.46 (m, 1H), 2.61 – 2.55 (m, 0.21H), 2.18 – 2.06 (m, 1H), 2.02 – 1.92 (m, 1H), 1.87 – 1.75 (m, 2.75H), 1.72 – 1.59 (m, 2.97H), 1.41 (m, 1H), 0.99 – 0.69 (m, 1.5H).

^{13}C{^{1}H} NMR (101 MHz, $CDCl_3$) δ = 170.1, 169.0, 157.3, 150.5, 146.0, 133.2, 133.1, 132.7, 131.2, 131.0, 128.9, 128.5, 128.4, 127.6, 127.4, 127.4, 126.3, 125.5, 123.0, 121.1, 119.1, 115.6, 54.9, 53.0, 43.2, 31.2, 30.9, 25.3, 25.1.

HRMS (ESI-FT) calcd for $C_{33}H_{32}N_5O_4^+$ ([M+H$^+$]) = 562.2449, Found 562.2445.

Chiral HPLC spectrum **5c**:

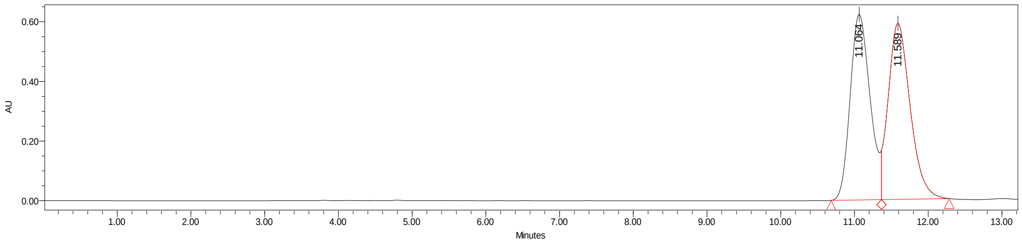

	Retention Time	Area	% Area
1	11.064	11749887	49.13
2	11.589	12164698	50.87

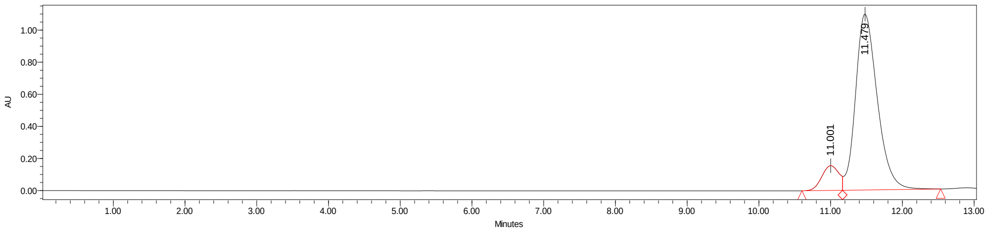

	Retention Time	Area	% Area
1	11.001	2542839	9.93
2	11.479	23068735	90.07

Dimethyl 2-{1-cyclobutyl-2-[1-(naphthalen-2-yl)-1H-tetrazol-5-yl]-2-(naphthalen-2-ylimino) ethyl}malonate (5d)

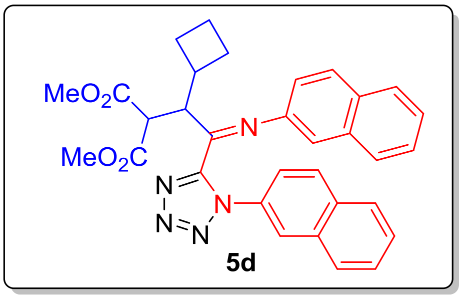

Yellow solid; m.p. 48-52 °C; 91% yield, 83:17 e.r.; $[\alpha]^{21}_{D}$ = –93.12 (c = 0.87 in CH_2Cl_2).

HPLC DAICEL CHIRALCEL IC, *n*-hexane/2-propanol = 70/30, flow rate = 1.0 mL/min, λ = 254 nm, t_R (minor) = 9.93 min, t_R (major) = 13.45 min.

IR (neat): 2953, 1740, 1508, 1437, 1267, 1157, 1022, 858, 816 and 746 cm^{-1}.

^{1}H NMR (400 MHz, $CDCl_3$) δ = 8.11 (s, 0.5H), 7.93 – 7.81 (m, 1.5H), 7.78 – 7.66 (m, 3H), 7.57 – 7.24 (m, 9.5H), 7.23 – 7.15 (m, 1.5H), 7.04 – 6.91 (m, 2.5H), 6.85 – 6.75 (m, 1H), 6.25 (s, 1H), 6.09 – 6.00 (m, 1H), 4.55 (d, J = 10.8 Hz, 0.5H), 4.20 – 4.13 (m, 1H), 4.08 (d, J = 6.6 Hz, 1H), 3.96 (t, J = 10.8 Hz, 0.5H), 3.87 – 3.63 (m, 9H), 3.27 – 3.09 (m, 1.5H), 2.27 – 2.11 (m, 2H), 2.08 – 1.76 (m, 5H), 1.76 – 1.66 (m, 1H), 1.54 – 1.45 (m, 3H).

^{13}C{^{1}H} NMR (101 MHz, $CDCl_3$) δ = 169.4, 169.1, 169.0, 156.4, 155.2, 150.9, 149.9, 145.7, 133.8, 133.4, 133.3, 133.2, 133.1, 132.8, 132.6, 131.0, 130.9, 129.1, 128.9, 128.5, 128.4, 128.0, 127.8, 127.6, 127.5, 127.4, 127.2, 126.6, 126.3, 125.5, 125.2, 124.8, 123.3, 122.8, 120.8, 120.1, 119.2, 116.0, 115.2, 53.9, 53.6, 53.4, 53.1, 52.9, 47.5, 38.3, 38.2, 28.6, 28.1, 27.4, 27.3, 27.0, 18.4, 18.2.

HRMS (ESI-FT) calcd for $C_{32}H_{30}N_5O_4^+$ ([M+H$^+$]) = 548.2292, Found 548.2299.

Chiral HPLC spectrum **5d**:

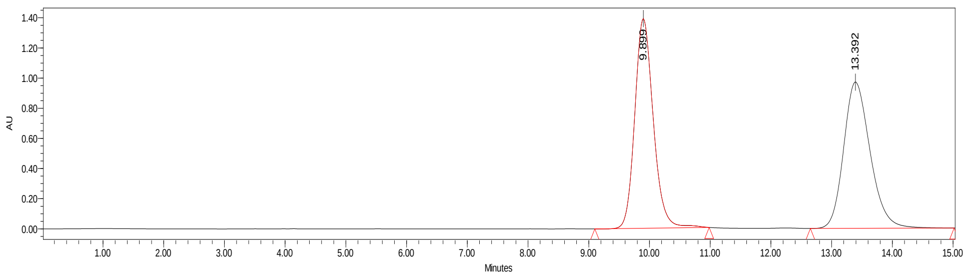

	Retention Time	Area	% Area
1	9.899	29427940	50.04
2	13.392	29383609	49.96

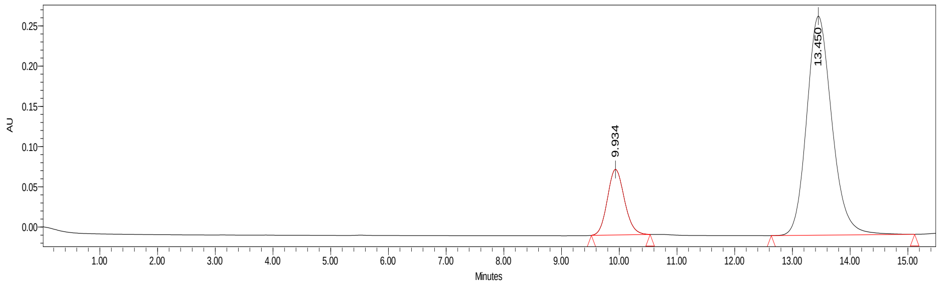

	Retention Time	Area	% Area
1	9.934	1671274	16.85
2	13.450	8248649	83.15

Dimethyl 2-{3,3-dimethyl-1-[1-(naphthalen-2-yl)-1H-tetrazol-5-yl]-1-(naphthalen-2-ylimino) butan-2-yl}malonate (5e)

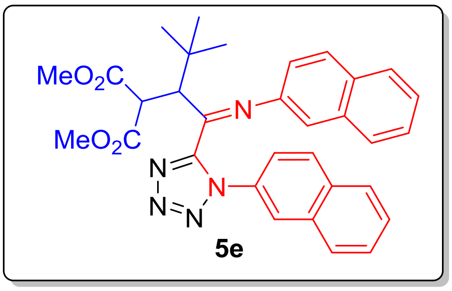

Yellow solid; m.p. 50-54 °C; 92% yield, 87:13 e.r.; $[\alpha]^{21}_{D}$ = –330.56 (c = 0.67 in CH_2Cl_2).

HPLC DAICEL CHIRALCEL IC, *n*-hexane/2-propanol = 70/30, flow rate = 1.0 mL/min, λ = 254 nm, t_R (minor) = 8.88 min, t_R (major) = 12.04 min.

IR (neat): 2961, 1740, 1506, 1292, 1217, 1157, 858, 816 and 745 cm^{-1}.

^{1}H NMR (400 MHz, $CDCl_3$) δ = 8.11 (s, 0.38H), 8.03 – 7.89 (m, 1.36H), 7.78 – 7.57 (m, 3.73H), 7.57 – 7.19 (m, 9.42H), 7.14 – 7.08 (m, 1H), 7.00 (d, J = 8.6 Hz, 1H),

6.92 – 6.84 (m, 1.33H), 6.40 (s, 1H), 6.20 (dd, *J* = 8.6, 2.0 Hz, 1H), 4.73 (d, *J* = 10.4 Hz, 0.36H), 4.33 (d, *J* = 8.0 Hz, 1H), 4.28 – 4.23 (m, 1.36H), 3.82 (s, 1H), 3.76 – 3.70 (m, 4H), 3.65 (s, 3H), 1.22 (s, 9H), 0.65 (s, 3.35H).

$^{13}C\{^1H\}$ NMR (101 MHz, $CDCl_3$) δ = 170.3, 169.9, 169.6, 156.7, 155.8, 152.8, 150.7, 146.1, 145.6, 133.8, 133.6, 133.4, 133.1, 133.0, 132.6, 132.6, 131.4, 131.1, 131.0, 129.0, 128.8, 128.7, 128.6, 128.5, 128.4, 128.1, 127.9, 127.7, 127.6, 127.6, 127.5, 127.4, 127.2, 126.4, 126.3, 125.5, 125.2, 124.5, 123.5, 121.3, 120.5, 119.4, 115.9, 115.5, 56.5, 54.1, 53.4, 53.3, 53.0, 52.9, 52.8, 49.1, 36.1, 35.4, 28.8, 27.0.

HRMS (ESI-FT) calcd for $C_{32}H_{32}N_5O_4^+$ ($[M+H^+]$) = 550.2449, Found 550.2444.

Chiral HPLC spectrum **5e**:

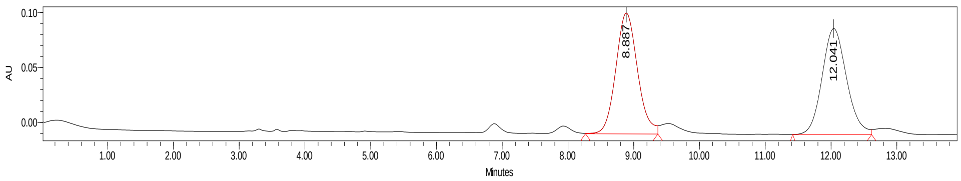

	Retention Time	Area	% Area
1	8.887	2524545	50.22
2	12.041	2502398	49.78

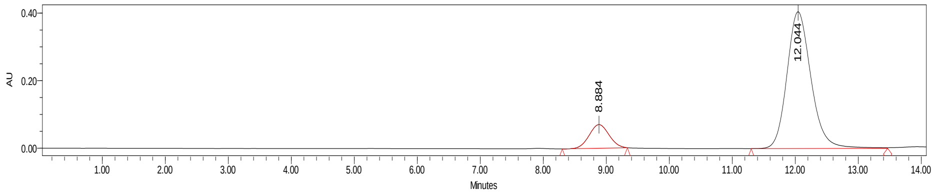

	Retention Time	Area	% Area
1	8.884	1587452	12.81
2	12.044	10803756	87.19

Dimethyl 2-{4-methyl-1-[1-(naphthalen-2-yl)-1H-tetrazol-5-yl]-1-(naphthalen-2-ylimino) pentan-2-yl}malonate (5f)

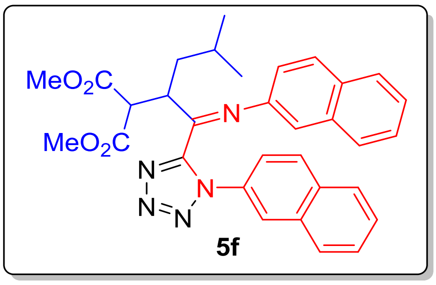

Yellow solid; m.p. 40-42 °C; 99% yield, 87:13 e.r.; $[\alpha]^{21}_{D}$ = –141.5 (c = 0.92 in CH_2Cl_2).

HPLC DAICEL CHIRALCEL IC, *n*-hexane/2-propanol = 70/30, flow rate = 1.0 mL/min, λ = 254 nm, t_R (minor) = 6.93 min, t_R (major) = 8.00 min.

IR (neat): 3057, 2957, 1738, 1597, 1508, 1437, 1265, 1159, 858, 816, 742 and 476 cm^{-1}.

^{1}H NMR (400 MHz, $CDCl_3$) δ = 8.13 (s, 0.40H), 7.98 – 7.84 (m, 1.40H), 7.81 – 7.68 (m, 3H), 7.62 – 7.21 (m, 10.60H), 7.16 – 7.04 (m, 2.40H), 6.93 (d, J = 8.4 Hz, 0.40H), 6.78 (d, J = 8.4 Hz, 1H), 6.31 (s, 1H), 6.10 (d, J = 8.4 Hz, 1H), 4.64 (d, J = 11.0 Hz, 0.40H), 4.31 (d, J = 7.6 Hz, 1H), 4.20 – 4.12 (m, 1.40H), 3.84 – 3.74 (m, 8.40H), 2.20 – 2.11 (m, 1.40H), 1.99 – 1.88 (m, 1H), 1.85 – 1.73 (m, 1H), 1.33 – 1.29 (m, 0.80H), 1.11 (d, J = 6.4 Hz, 3H), 1.04 (d, J = 6.4 Hz, 1H), 0.75 (d, J = 5.6 Hz, 1.20H), 0.26 (d, J = 5.6 Hz, 1.20H).

^{13}C{^{1}H} NMR (101 MHz, $CDCl_3$) δ = 169.1, 168.9, 157.3, 157.0, 150.9, 149.6, 145.5, 145.4, 133.7, 133.5, 133.3, 133.2, 132.8, 132.6, 131.0, 130.9, 130.7, 129.2, 128.9, 128.5, 128.4, 128.0, 127.8, 127.7, 127.6, 127.5, 127.4, 127.2, 126.6, 126.4, 125.6, 125.2, 124.8, 123.4, 122.8, 120.8, 119.8, 119.3, 116.3, 115.1, 54.8, 54.6, 53.1, 52.8, 46.5, 41.5, 40.2, 39.6, 27.0, 26.2, 25.3, 23.4, 23.1, 22.7, 21.6.

HRMS (ESI-FT) calcd for $C_{32}H_{32}N_5O_4^+$ ($[M+H^+]$) = 550.2449, Found 550.2446.

Chiral HPLC spectrum **5f**:

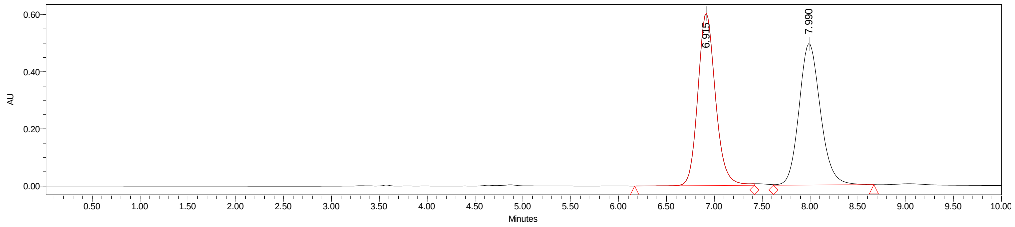

	Retention Time	Area	% Area
1	6.915	7697531	50.02
2	7.990	7690613	49.98

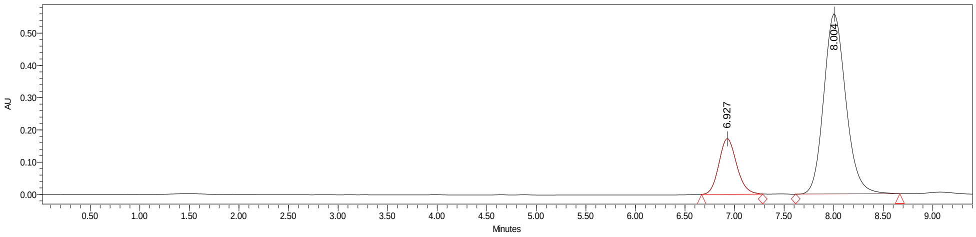

	Retention Time	Area	% Area
1	6.927	2155528	20.00
2	8.004	8623933	80.00

Dimethyl 2-{3-methyl-1-[1-(naphthalen-2-yl)-1H-tetrazol-5-yl]-1-(naphthalen-2-ylimino) butan-2-yl}malonate (5g)

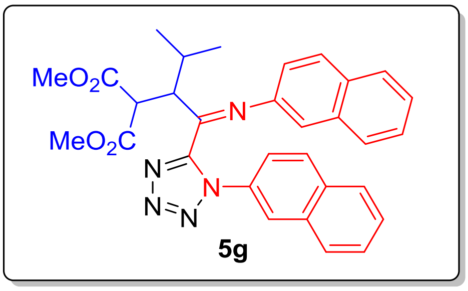

Yellow solid; m.p. 88-90 °C; 93% yield, 93:7 e.r.; $[\alpha]^{21}_{D}$ = –132.8 (*c* = 0.81 in CH_2Cl_2).

HPLC DAICEL CHIRALCEL IC, *n*-hexane/2-propanol = 70/30, flow rate = 1.0 mL/min, λ = 254 nm, t_R (minor) = 9.79 min, t_R (major) = 12.44 min.

IR (neat): 2963, 1740, 1263, 1155, 1026, 858, 814, 750 and 474 cm^{-1}.

^{1}H NMR (400 MHz, $CDCl_3$) δ = 7.72 – 7.68 (m, 1H), 7.56 – 7.53 (m, 4H), 7.38 – 7.27 (m, 3H), 7.25 – 7.18 (m, 1H), 7.03 (dd, *J* = 18.8, 8.0 Hz, 2H), 6.84 – 6.57 (m, 1H), 6.27 (s, 1H), 6.18 – 5.90 (m, 1H), 4.32 (d, *J* = 8.8 Hz, 1H), 4.11 (dd, *J* = 8.8, 4.8 Hz, 1H), 3.79 (s, 3H), 3.73 (s, 3H), 2.80 – 2.51 (m, 1H), 1.23 (dd, *J* = 16.4, 6.8 Hz, 6H).

^{13}C{^{1}H} NMR (101 MHz, $CDCl_3$) δ = 169.7, 169.3, 156.7, 150.1, 145.6, 133.3, 133.2, 132.6, 131.0, 130.9, 129.1, 128.5, 128.4, 127.7, 127.6, 127.4, 127.2, 126.4, 125.6, 123.1, 121.0, 119.2, 115.9, 53.7, 53.0, 52.8, 52.5, 29.9, 27.0, 20.8, 18.8.

HRMS (ESI-FT) calcd for $C_{31}H_{30}N_5O_4^+$ ([M+H$^+$]) = 536.2292, Found 536.2289.

Chiral HPLC spectrum **5g**:

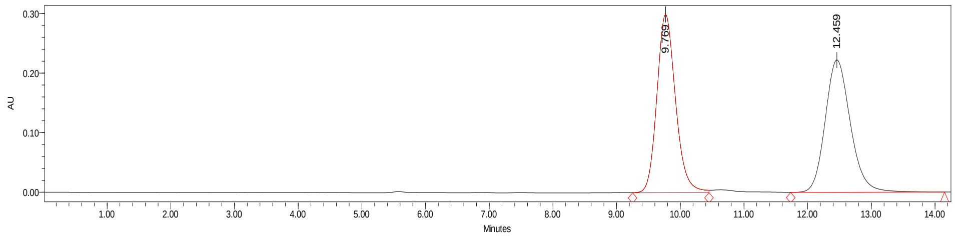

	Retention Time	Area	% Area
1	9.769	6106548	49.95
2	12.459	6118094	50.05

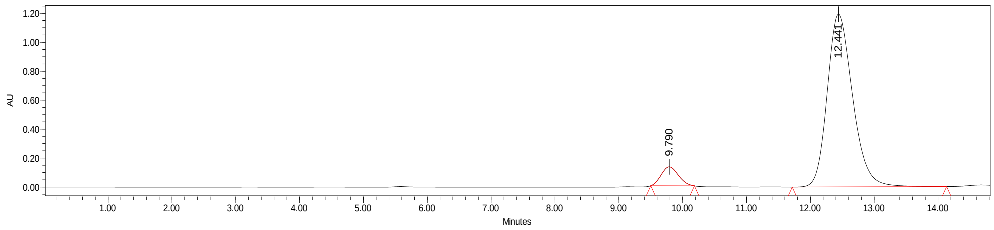

	Retention Time	Area	% Area
1	9.790	2452059	6.92
2	12.441	33004979	93.08

Dibenzyl 2-{1-[1-(2,3-dihydrobenzo[b][1,4]dioxin-6-yl)-1H-tetrazol-5-yl]-1-[(2,3-dihydrobenzo[b][1,4]dioxin-6-yl)imino]-3-methylbutan-2-yl}malonate (5h)

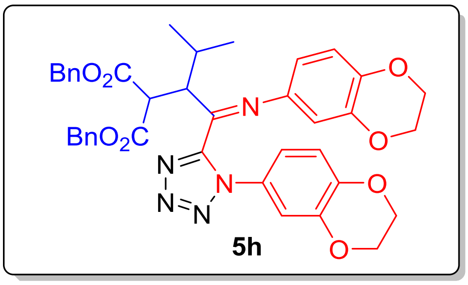

Yellow solid; m.p. 42-44 °C; 92% yield, 91:9 e.r.; $[\alpha]^{21}_{D}$ = –110.1 (c = 0.76 in CH_2Cl_2).

HPLC DAICEL CHIRALCEL IA, *n*-hexane/2-propanol = 70/30, flow rate = 1.0 mL/min, λ = 254 nm, t_R (minor) = 12.03 min, t_R (major) = 18.20 min.

IR (neat): 2970, 1734, 1587, 1504, 1460, 1377, 1306, 1064, 895, 812, 743 and 698 cm^{-1}.

^{1}H NMR (400 MHz, $CDCl_3$) δ = 7.34 – 7.22 (m, 10H), 6.71 – 6.65 (m, 1H), 6.59 – 6.47 (m, 2H), 6.39 (d, J = 8.4 Hz, 1H), 5.76 – 5.63 (m, 1H), 5.56 – 5.43 (m, 1H), 5.19

– 5.00 (m, 4H), 4.37 – 4.10 (m, 9H), 4.03 – 3.92 (m, 1H), 2.53 – 2.35 (m, 1H), 1.06 (dd, *J* = 20.4, 7.0 Hz, 6H).

^{13}C{^{1}H} NMR (101 MHz, $CDCl_3$) δ = 168.8, 168.3, 155.3, 149.8, 145.0, 143.6, 142.1, 141.6, 135.5, 135.1, 128.7, 128.5, 128.5, 128.3, 128.2, 128.0, 127.0, 117.7, 117.2, 117.0, 113.5, 112.6, 109.3, 67.6, 67.5, 67.3, 64.4, 64.3, 64.2, 53.2, 53.0, 29.8, 20.3, 18.8.

HRMS (ESI-FT) calcd for $C_{39}H_{38}N_5O_8^+$ ([M+H$^+$]) = 704.2715, Found 704.2711.

Chiral HPLC spectrum **5h**:

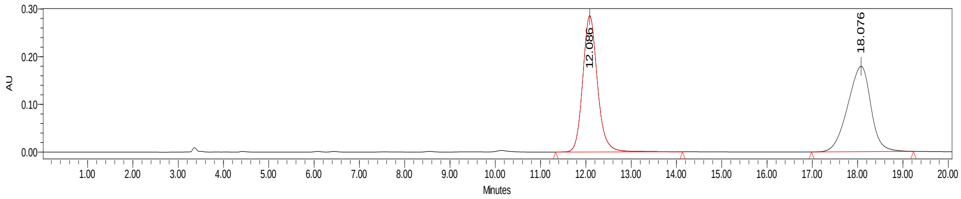

	Retention Time	Area	% Area
1	12.086	6428700	50.31
2	18.076	6349209	49.69

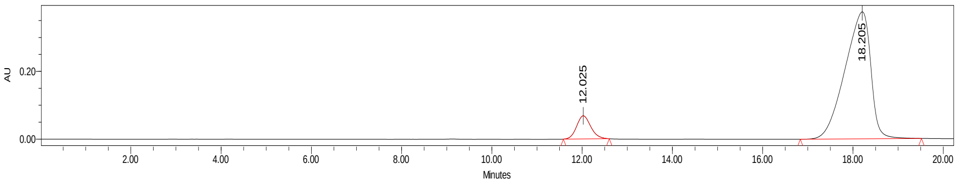

	Retention Time	Area	% Area
1	12.025	1485645	9.07
2	18.205	14900452	90.93

Dimethyl 2-{1-cyclohexyl-2-[1-(2,3-dihydrobenzo[b][1,4]dioxin-6-yl)-1H-tetrazol-5-yl]-2-[(2,3-dihydrobenzo[b][1,4]dioxin-6-yl)imino]ethyl}malonate (5i)

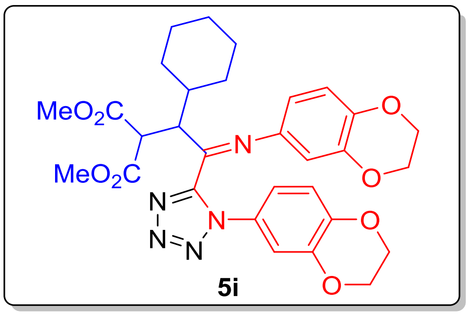

5i

Yellow solid; m.p. 58-62 °C; 93% yield, 90.5:9.5 e.r.; $[\alpha]^{21}_D$ = –103.7 (*c* = 0.50 in

CH_2Cl_2).

HPLC DAICEL CHIRALCEL IC, *n*-hexane/2-propanol = 70/30, flow rate = 1.0 mL/min, λ = 254 nm, t_R (minor) = 21.55 min, t_R (major) = 25.52 min.

IR (neat): 2932, 1738, 1587, 1505, 1306, 1064, 893, 812 and 737 cm^{-1}.

^{1}H NMR (400 MHz, $CDCl_3$) δ = 6.76 – 6.68 (m, 1H), 6.61 – 6.45 (m, 2H), 6.41 (d, *J* = 8.6 Hz, 1H), 5.72 (s, 1H), 5.58 (d, *J* = 8.6 Hz, 1H), 4.31 – 4.23 (m, 5H), 4.15 (d, *J* = 6.2 Hz, 4H), 3.94 (dd, *J* = 8.8, 3.8 Hz, 1H), 3.74 (s, 3H), 3.70 (s, 3H), 2.01 (s, 1H), 1.93 – 1.74 (m, 4H), 1.35 – 1.06 (m, 6H).

^{13}C{^{1}H} NMR (101 MHz, $CDCl_3$) δ = 169.6, 169.1, 155.5, 149.9, 145.1, 143.7, 143.6, 142.2, 141.6, 127.1, 117.8, 117.2, 117.1, 113.5, 112.5, 109.3, 64.5, 64.4, 64.3, 53.2, 52.8, 52.5, 40.1, 31.0, 29.3, 27.0, 26.8, 26.4.

HRMS (ESI-FT) calcd for $C_{30}H_{34}N_5O_8^+$ ([M+H$^+$]) = 592.2402, Found 592.2396.

Chiral HPLC spectrum **5i**:

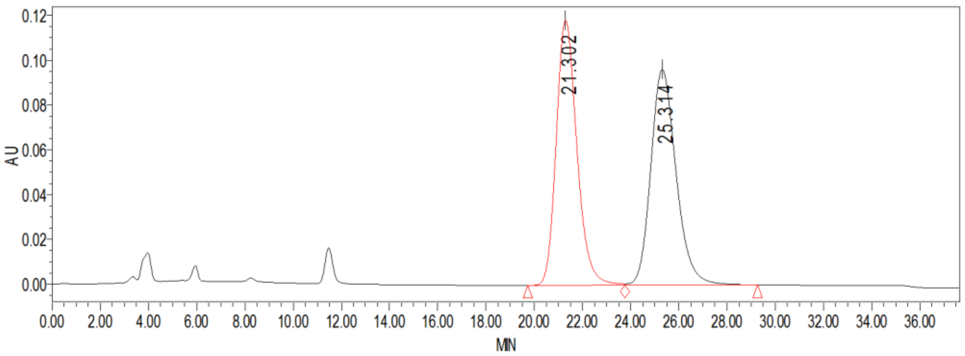

	Retention Time	Area	% Area
1	21.302	7049224	50.12
2	25.314	7016275	49.88

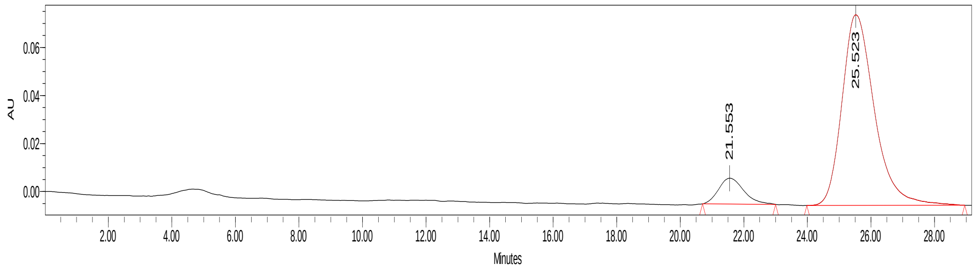

	Retention Time	Area	% Area
1	21.553	593020	9.55
2	25.523	5615897	90.45

Diethyl 2-{1-cyclohexyl-2-[1-(2,3-dihydrobenzo[b][1,4]dioxin-6-yl)-1H-tetrazol-5-yl]-2-[(2,3-dihydrobenzo[b][1,4]dioxin-6-yl)imino]ethyl}malonate (5j)

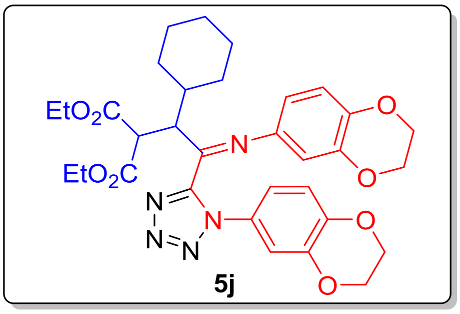

Yellow solid; m.p. 50-52 °C; 91% yield, 93:7 e.r.; $[\alpha]^{21}_{D}$ = –119.7 (*c* = 0.89 in CH_2Cl_2).

HPLC DAICEL CHIRALCEL IA, *n*-hexane/2-propanol = 70/30, flow rate = 1.0 mL/min, λ = 254 nm, t_R (minor) = 6.97 min, t_R (major) = 7.39 min.

IR (neat): 2932, 1732, 1587, 1504, 1373, 1306, 1065, 891, 864, 810, 739 and 615 cm^{-1}.

^{1}H NMR (400 MHz, $CDCl_3$) δ = 6.72 (d, *J* = 8.4 Hz, 1H), 6.66 – 6.45 (m, 2H), 6.44 – 6.40 (m, 1H), 5.90 – 5.68 (m, 1H), 5.67 – 5.42 (m, 1H), 4.32 – 4.12 (m, 13H), 3.95 (dd, *J* = 9.2, 4.0 Hz, 1H), 2.06 – 1.75 (m, 5H), 1.71 – 1.64 (m, 1H), 1.60 – 1.45 (m, 1H), 1.31 – 1.16 (m, 10H).

^{13}C{^{1}H} NMR (101 MHz, $CDCl_3$) δ = 169.3, 168.7, 155.6, 150.0, 145.0, 143.6, 142.3, 141.5, 127.2, 117.7, 117.1, 117.0, 113.5, 112.5, 109.3, 64.5, 64.3, 64.2, 61.8, 61.6, 53.0, 52.9, 40.2, 30.9, 29.5, 27.0, 26.9, 26.4, 14.2, 14.1.

HRMS (ESI-FT) calcd for $C_{32}H_{38}N_5O_8^+$ ([M+H$^+$]) = 620.2715, Found 620.2713.

Chiral HPLC spectrum **5j**:

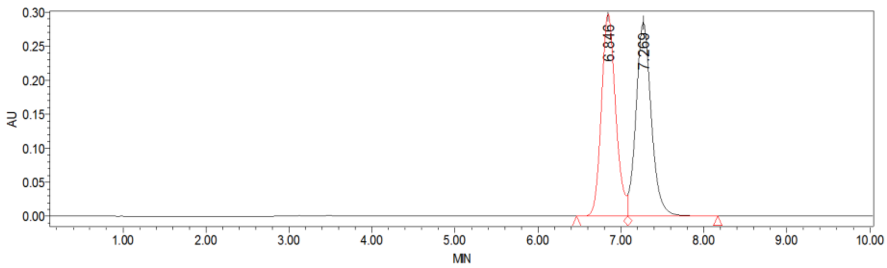

	Retention Time	Area	% Area
1	6.846	3531594	49.30
2	7.269	3631243	50.70

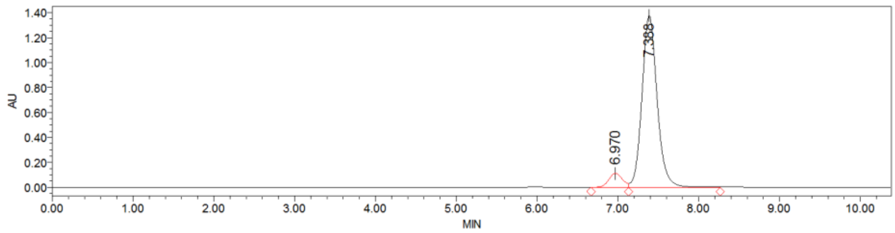

	Retention Time	Area	% Area
1	6.970	1320513	6.86
2	7.388	17930363	93.14

Diisopropyl 2-{1-cyclohexyl-2-[1-(2,3-dihydrobenzo[b][1,4]dioxin-6-yl)-1H-tetrazol-5-yl]-2-[(2,3-dihydrobenzo[b][1,4]dioxin-6-yl)imino]ethyl}malonate (5k)

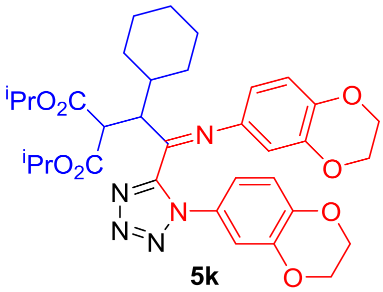

5k

Yellow solid; m.p. 52-54 °C; 88% yield, 87.5:12.5 e.r.; $[\alpha]^{21}_{D}$ = –140.3 (*c* = 0.80 in CH_2Cl_2).

HPLC DAICEL CHIRALCEL IA, *n*-hexane/2-propanol = 70/30, flow rate = 1.0 mL/min, λ = 254 nm, t_R (minor) = 5.95 min, t_R (major) = 6.34 min.

IR (neat): 2982, 2932, 1728, 1587, 1504, 1458, 1306, 1103, 1065, 895, 812 and 739 cm^{-1}.

^{1}H NMR (400 MHz, $CDCl_3$) δ = 6.83 – 6.66 (m, 1H), 6.59 – 6.50 (m, 2H), 6.49 – 6.32 (m, 1H), 5.76 – 5.66 (m, 1H), 5.63 – 5.49 (m, 1H), 5.22 – 4.75 (m, 2H), 4.40 – 4.01 (m, 8H), 3.93 (dd, *J* = 10.0, 4.0 Hz, 1H), 2.06 – 1.73 (m, 7H), 1.32 – 1.05 (m, 15H).

^{13}C{^{1}H} NMR (101 MHz, $CDCl_3$) δ = 168.7, 168.4, 155.6, 150.0, 145.0, 143.6, 142.3, 141.5, 127.2, 119.5, 117.7, 117.1, 117.0, 113.6, 112.9, 112.6, 109.3, 109.0, 69.2, 64.5, 64.3, 64.2, 53.6, 52.6, 40.4, 30.8, 29.5, 27.1, 27.0, 26.4, 21.8, 21.7, 21.6, 21.5.

HRMS (ESI-FT) calcd for $C_{34}H_{42}N_5O_8^+$ ($[M+H^+]$) = 648.3028, Found 648.3033.

Chiral HPLC spectrum **5k**:

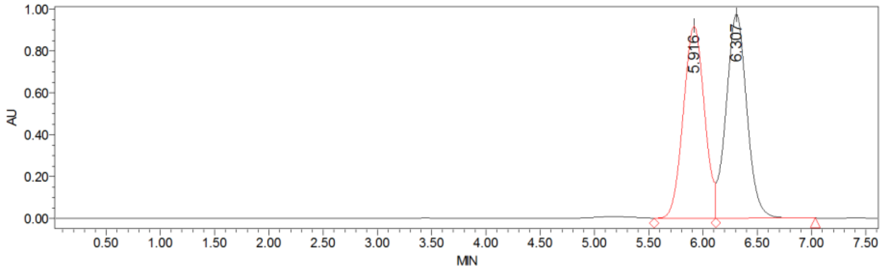

	Retention Time	Area	% Area
1	5.916	12385768	49.14
2	6.307	12819936	50.86

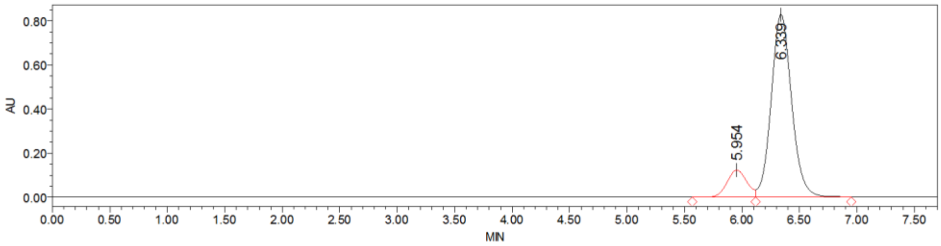

	Retention Time	Area	% Area
1	5.954	1459786	12.48
2	6.339	10241701	87.52

Dibenzyl 2-{1-cyclohexyl-2-[1-(2,3-dihydrobenzo[b][1,4]dioxin-6-yl)-1H-tetrazol-5-yl] -2-[(2,3-dihydrobenzo[b][1,4]dioxin-6-yl)imino]ethyl}malonate (5l)

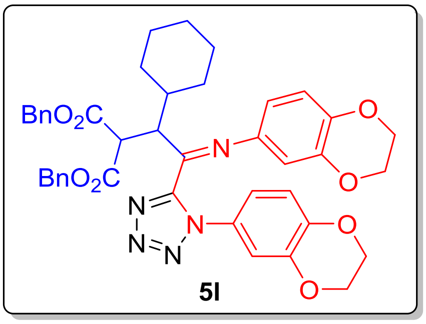

Yellow solid; m.p. 48-52 °C; 92% yield, 95.5:4.5 e.r.; $[\alpha]^{21}_{D}$ = –149.5 (*c* = 0.94 in CH_2Cl_2).

HPLC DAICEL CHIRALCEL IC, *n*-hexane/2-propanol = 70/30, flow rate = 1.0 mL/min, λ = 254 nm, t_R (minor) = 20.56 min, t_R (major) = 22.74 min.

IR (neat): 2928, 1732, 1503, 1375, 1063, 893, 810, 746 and 696 cm^{-1}.

^{1}H NMR (400 MHz, $CDCl_3$) δ = 7.32 (s, 5H), 7.24 (s, 5H), 6.71 – 6.62 (m, 1H), 6.60

– 6.43 (m, 2H), 6.41 – 6.32 (m, 1H), 5.70 (s, 1H), 5.52 (d, J = 8.4 Hz, 1H), 5.23 – 5.10 (m, 3H), 5.06 (d, J = 13.2 Hz, 1H), 4.34 (d, J = 9.2 Hz, 1H), 4.30 – 4.12 (m, 8H), 4.02 – 3.94 (m, 1H), 1.92 (s, 1H), 1.83 – 1.56 (m, 5H), 1.27 – 1.02 (m, 5H).

^{13}C{^{1}H} NMR (101 MHz, $CDCl_3$) δ = 168.8, 168.4, 155.3, 149.9, 145.0, 143.6, 142.2, 141.5, 135.5, 135.1, 128.7, 128.6, 128.5, 128.4, 128.3, 128.2, 128.0 127.0, 117.7, 117.2, 117.0, 113.5, 112.6, 109.3, 67.5, 67.2, 64.4, 64.3, 64.2, 53.0, 40.1, 30.7, 29.5, 26.9, 26.7, 26.2.

HRMS (ESI-FT) calcd for $C_{42}H_{42}N_5O_8^+$ ([M+H$^+$]) = 744.3028, Found 744.3020.

Chiral HPLC spectrum **5l**:

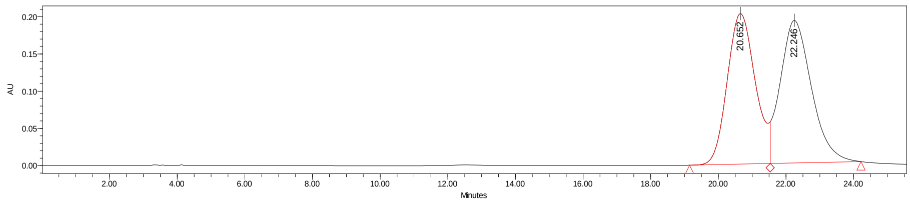

	Retention Time	Area	% Area
1	20.652	11671290	48.83
2	22.246	12228496	51.17

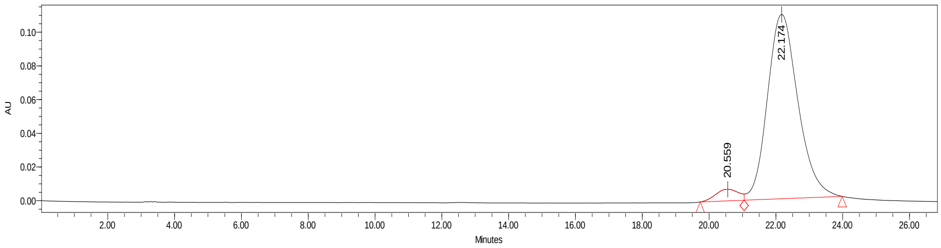

	Retention Time	Area	% Area
1	20.559	329808	4.53
2	22.174	6955873	95.47

Dibenzyl 2-{1-cyclohexyl-2-[1-(naphthalen-2-yl)-1H-tetrazol-5-yl]-2-(naphthalen-2-ylimino) ethyl}malonate (5m)

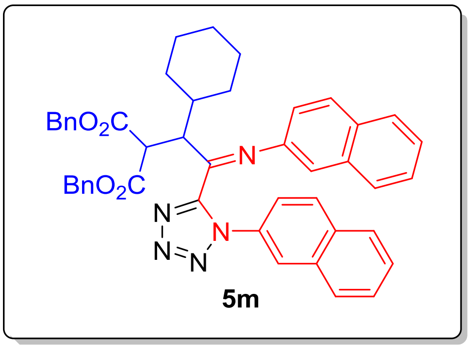

Yellow solid; m.p. 46-50 °C; 88% yield, 92:8 e.r.; $[\alpha]^{21}_{D}$ = −173.9 (c = 0.84 in CH_2Cl_2).

HPLC DAICEL CHIRALCEL IA, *n*-hexane/2-propanol = 70/30, flow rate = 1.0 mL/min, λ = 254 nm, t_R (minor) = 6.90 min, t_R (major) = 9.60 min.

IR (neat): 2930, 2855, 1734, 1504, 1452, 1267, 1022, 856, 814, 745, 698 and 476 cm^{-1}.

^{1}H NMR (400 MHz, $CDCl_3$) δ = 7.98 (s, 0.1H), 7.93 – 7.82 (m, 1H), 7.71 – 7.67 (m, 1.1H), 7.56 – 7.112 (m, 19.1H), 7.00 (dd, J = 22.4, 8.4 Hz, 2H), 6.87 (d, J = 8.0 Hz, 0.1H), 6.73 (d, J = 8.8 Hz, 1H), 6.21 (s, 1H), 5.99 (d, J = 8.8 Hz, 1H), 5.33 – 4.98 (m, 4.4H), 4.78 (d, J = 9.6 Hz, 0.1H), 4.44 (d, J = 9.2 Hz, 1H), 4.22 – 4.08 (m, 1H), 3.97 – 3.94 (m, 0.1H), 2.17 – 2.04 (m, 1.1H), 1.98 – 1.96 (m, 2.2H), 1.84 – 1.72 (m, 2.2H), 1.70 – 1.6 (m, 1.1H), 1.37 – 1.13 (m, 5.5H).

^{13}C{^{1}H} NMR (101 MHz, $CDCl_3$) δ = 169.0, 168.5, 156.6, 150.1, 145.7, 135.4, 135.1, 133.3, 133.1, 132.6, 131.0, 130.9, 129.1, 128.8, 128.7, 128.5, 128.4, 128.3, 128.1, 127.6, 127.5, 127.4, 127.2, 126.3, 125.5, 123.0, 121.0, 119.4, 116.0, 67.6, 67.4, 53.2, 52.9, 40.1, 31.1, 29.5, 27.0, 26.8, 26.3.

HRMS (ESI-FT) calcd for $C_{46}H_{42}N_5O_4^+$ ([M+H$^+$]) = 728.3231, Found 728.3233.

Chiral HPLC spectrum **5m**:

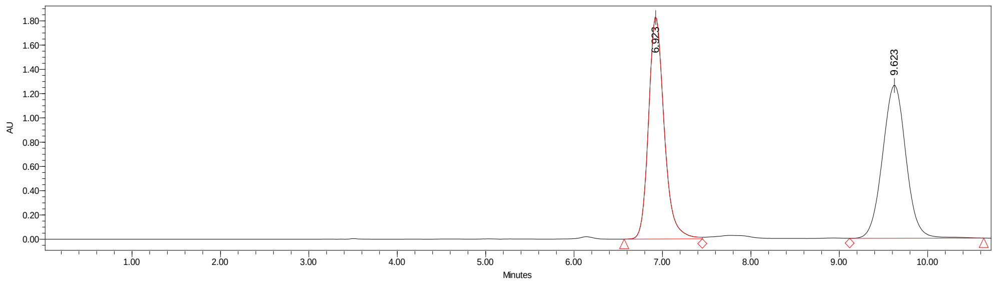

	Retention Time	Area	% Area
1	6.923	22450730	49.12
2	9.623	23252280	50.88

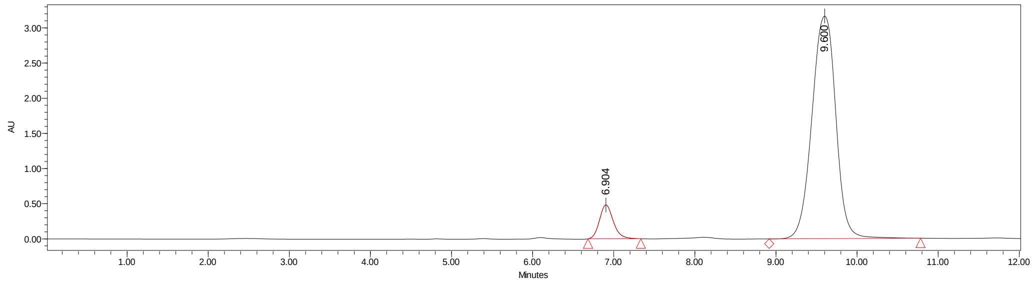

	Retention Time	Area	% Area
1	6.904	5697486	8.08
2	9.600	64793579	91.92

Dibenzyl 2-{1-cyclohexyl-2-[1-(4-methoxyphenyl)-1H-tetrazol-5-yl]-2-[(4-methoxyphenyl) imino]ethyl}malonate (5n)

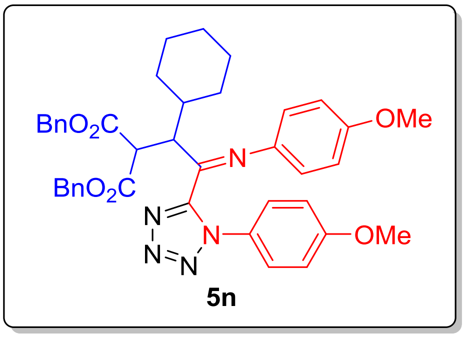

Yellow solid; m.p. 46-50 °C; 80% yield, 92.5:7.5 e.r.; $[\alpha]^{21}_{D}$ = –220.9 (*c* = 0.62 in CH_2Cl_2).

HPLC DAICEL CHIRALCEL IA, *n*-hexane/2-propanol = 70/30, flow rate = 1.0 mL/min, λ = 254 nm, t_R (minor) = 7.05 min, t_R (major) = 12.46 min.

IR (neat): 2928, 1738, 1605, 1508, 1452, 1252, 1028, 833, 746 and 698 cm^{-1}.

^{1}**H NMR** (400 MHz, $CDCl_3$) δ = 7.36 – 7.20 (m, 13.3H), 6.96 – 6.86 (m, 2.9H), 6.81 – 6.74 (m, 1.3H), 6.72 – 6.65 (m, 2H), 6.43 (d, *J* = 8.8 Hz, 2H), 5.99 (d, *J* = 8.8 Hz, 2H), 5.25 – 4.93 (m, 5.2H), 4.73 (d, *J* = 10.2 Hz, 0.3H), 4.34 (d, *J* = 9.4 Hz, 1H), 4.01 – 3.87 (m, 1.3H), 3.85 – 3.60 (m, 7.8H), 2.05 – 1.89 (m, 1.3H), 1.80 – 1.68 (m, 3.9H), 1.63 – 1.45 (m, 2.6H), 1.26 – 0.96 (m, 6.5H).

^{13}**C{**^{1}**H} NMR** (101 MHz, $CDCl_3$) δ = 168.9, 168.8, 168.6, 168.4, 160.7, 160.5, 157.4, 156.9, 155.5, 155.3, 151.7, 149.9, 141.6, 135.5, 135.2, 135.1, 128.7, 128.6, 128.5, 128.4, 128.3, 128.2, 128.0, 127.8, 126.7, 125.5, 121.1, 120.9, 114.2, 114.1, 113.9,

67.6, 67.5, 67.2, 55.8, 55.7, 53.9, 53.0, 52.8, 45.9, 41.1, 39.9, 31.2, 30.9, 30.6, 29.4, 26.9, 26.7, 26.3, 26.2, 25.9.

HRMS (ESI-FT) calcd for $C_{40}H_{42}N_5O_6^+$ ($[M+H^+]$) = 688.3130, Found 688.3132.

Chiral HPLC spectrum **5n**:

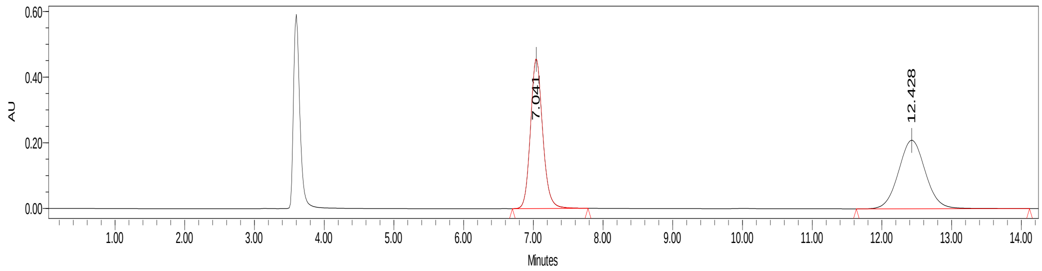

	Retention Time	Area	% Area
1	7.041	5527798	49.69
2	12.428	5596898	50.31

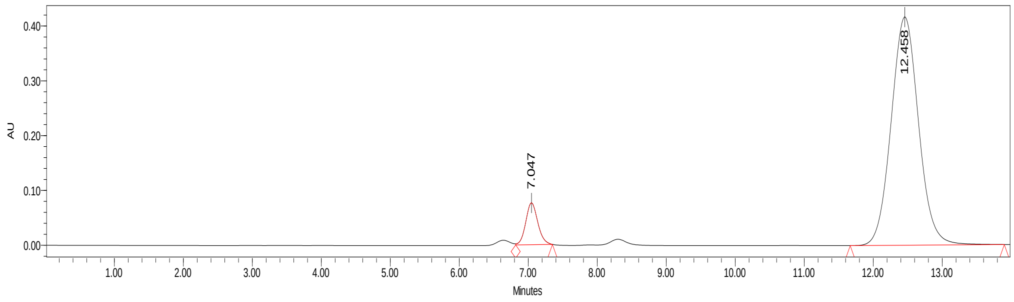

	Retention Time	Area	% Area
1	7.047	926386	7.46
2	12.458	11494782	92.54

Methyl 4,4-dimethyl-3-{[1-(naphthalen-2-yl)-1H-tetrazol-5-yl](naphthalen-2-ylimino) Methyl}pentanoate (6e)

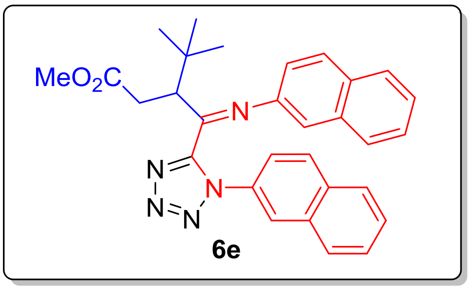

Yellow oil; 70% yield, 85:15 e.r.; $[\alpha]^{21}_{D}$ = –106.2 (c = 0.26 in CH_2Cl_2).

UPC^2 Phenomenex CHIRALCEL IC-3, CO_2/CH_3OH = 90/10, flow rate = 1.5

mL/min, λ = 254 nm, t_R (minor) = 11.99 min, t_R (major) = 12.23 min.

IR (neat): 2961, 1732, 1628, 1207, 1107, 856, 814, 746 and 474 cm^{-1}.

^{1}H NMR (400 MHz, $CDCl_3$) δ = 7.77 – 7.68 (m, 1H), 7.56 – 7.28 (m, 6H), 7.24 – 7.18 (m, 1H), 7.14 – 7.05 (m, 2H), 7.01 (d, *J* = 8.6 Hz, 1H), 6.82 (d, *J* = 8.4 Hz, 1H), 6.37 (s, 1H), 6.17 (d, *J* = 8.4 Hz, 1H), 3.84 (d, *J* = 10.4 Hz, 1H), 3.68 (s, 3H), 3.31 – 3.18 (m, 1H), 2.87 (d, *J* = 17.2 Hz, 1H), 1.22 (s, 9H).

^{13}C{^{1}H} NMR (101 MHz, $CDCl_3$) δ = 173.9, 146.0, 133.4, 132.6, 130.9, 129.1, 128.5, 128.2, 127.7, 127.6, 127.4, 127.2, 126.3, 125.4, 123.1, 121.0, 119.5, 116.0, 53.8, 52.0, 35.5, 35.4, 28.6.

HRMS (ESI-FT) calcd for $C_{30}H_{30}N_5O_2^+$ ($[M+H^+]$) = 492.2394, Found 492.2392.

Chiral HPLC spectrum **6e**:

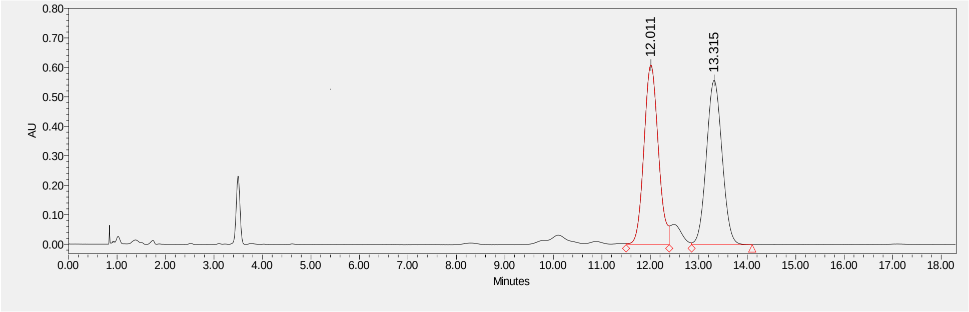

	Retention Time	% Area
1	12.011	50.12
2	13.315	49.88

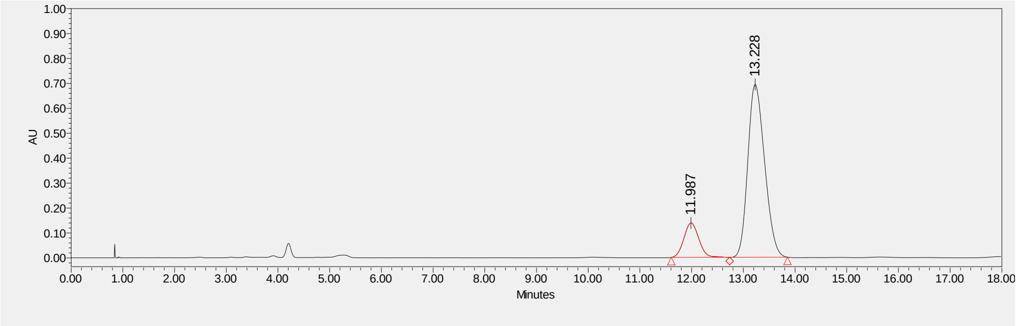

	Retention Time	% Area
1	11.987	14.97
2	13.228	85.03

Dimethyl 2-{2-[1-(naphthalen-2-yl)-1H-tetrazol-5-yl]-2-(naphthalen-2-ylimino)-1-phenylethyl}malonate (5o)

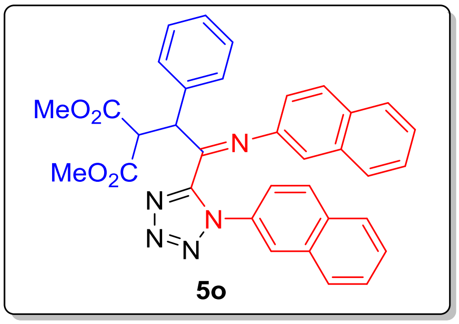

5o

Yellow solid; m.p. 50-52 °C; 99% yield, 94.5:5.5 e.r.; $[\alpha]^{26}_{D}$ = –272.2 (*c* = 0.90 in CH_2Cl_2).

HPLC DAICEL CHIRALCEL IA, *n*-hexane/2-propanol = 70/30, flow rate = 1.0 mL/min, λ = 330 nm, t_R (minor) = 6.39 min, t_R (major) = 11.26 min.

IR (neat): 1744, 1502, 1437, 1302, 1265, 1149, 1030, 860, 816,750 and 702 cm^{-1}.

^{1}H NMR (400 MHz, $CDCl_3$) δ = 7.89 – 6.92 (m, 15H), 6.48 – 6.19 (m, 2H), 6.16 – 6.01(m, 1H), 6.00 – 5.76 (m, 1H), 5.41 (d, *J* = 12.0 Hz, 1H), 4.85 (d, *J* = 12.4 Hz, 1H), 3.78 (s, 3H), 3.53 (s, 3H).

^{13}C{^{1}H} NMR (101 MHz, $CDCl_3$) δ = 168.2, 167.9, 167.2, 155.9, 155.8, 149.6, 145.1, 134.7, 133.8, 133.3, 133.1, 132.8, 132.3, 131.1, 130.7, 130.2, 129.5, 129.1, 128.9, 128.7, 128.7, 128.6, 128.5, 128.1, 127.7, 127.6, 127.5, 127.4, 127.2, 126.5, 125.7, 125.2, 122.8, 122.6, 120.7, 119.3, 116.6, 57.0, 55.2, 54.36, 53.1, 52.7, 40.8.

HRMS (ESI-FT) calcd for $C_{34}H_{28}N_5O_4^+$ ($[M+H^+]$) = 570.2136, Found 570.2133.

Chiral HPLC spectrum **5o**:

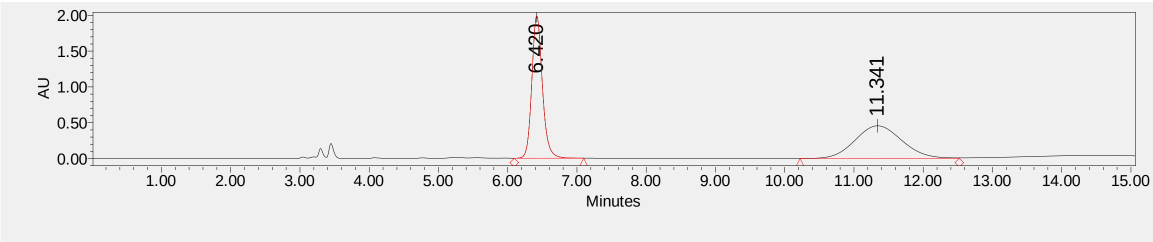

	Retention Time	Area	% Area
1	6.420	21082548	49.59
2	11.341	21430829	50.41

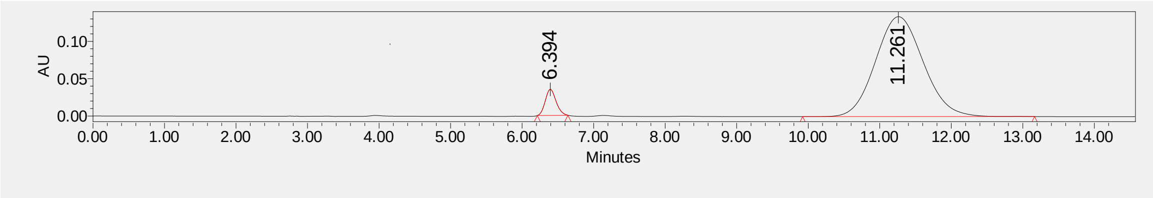

	Retention Time	Area	% Area
1	6.394	367458	5.59
2	11.261	6208176	94.41

Dimethyl 2-{1-(4-methoxyphenyl)-2-[1-(naphthalen-2-yl)-1H-tetrazol-5-yl]-2-(naphthalen-2-ylimino)ethyl}malonate (5p)

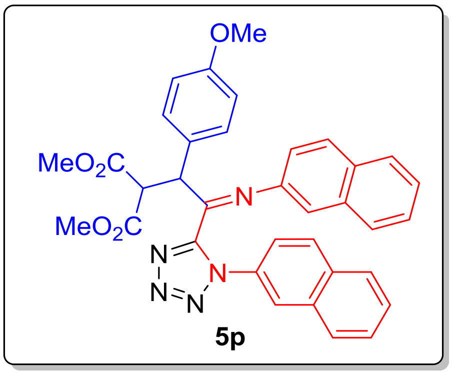

Yellow solid; m.p. 50-52 °C; 93% yield, 84.5:15.5 e.r.; $[\alpha]^{26}_{D}$ = –233.4 (c = 0.99 in CH_2Cl_2).

HPLC DAICEL CHIRALCEL IA, *n*-hexane/2-propanol = 70/30, flow rate = 1.0 mL/min, λ = 330 nm, t_R (minor) = 8.14 min, t_R (major) = 14.18 min.

IR (neat): 1744, 1605, 1510, 1437, 1302, 1258, 1182, 1032, 858, 816 and 748 cm^{-1}.

^{1}H NMR (400 MHz, $CDCl_3$) δ = 7.70 – 7.66 (m, 1H), 7.60 – 7.30 (m, 7H), 7.29 – 7.26 (m, 1H), 7.21– 7.16 (m, 1H), 7.09 (t, J = 8.8 Hz, 2H), 7.00 – 6.91 (m, 2H), 6.44 (s, 1H), 6.36 –6.28 (m, 1H), 6.17 – 6.07 (m, 1H), 6.02 –5.90 (m, 1H), 5.34 (d, J = 12.0 Hz, 1H), 4.79 (d, J = 11.6 Hz, 1H), 3.82 (s, 3H), 3.77 (s, 3H), 3.55 (s, 3H).

^{13}C{^{1}H} NMR (101 MHz, $CDCl_3$) δ = 168.3, 168.0, 160.0, 156.0, 149.7, 145.2, 133.4, 133.2, 132.4, 131.0, 130.7, 130.2, 129.1, 128.6, 128.1, 127.7, 127.5, 127.4, 127.2, 126.5, 126.2, 125.7, 122.7, 120.8, 119.4, 116.6, 114.9, 55.4, 55.3, 53.6, 53.0, 52.7.

HRMS (ESI-FT) calcd for $C_{35}H_{30}N_5O_5^+$ ([M+H$^+$]) = 600.2241, Found 600.2238.

Chiral HPLC spectrum **5p**:

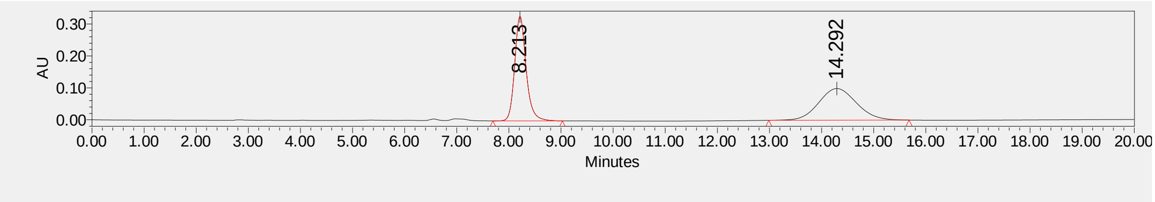

	Retention Time	Area	% Area
1	8.213	4977243	49.88
2	14.292	5000261	50.12

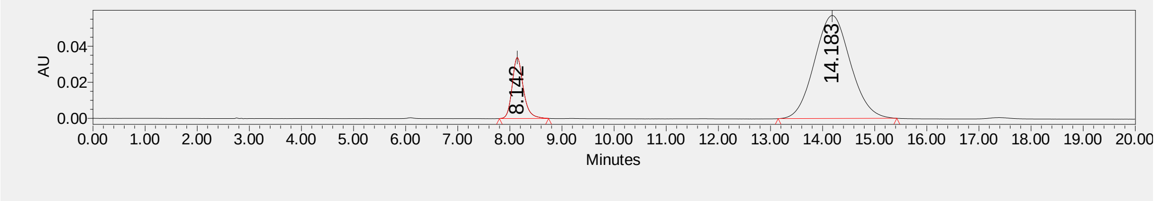

	Retention Time	Area	% Area
1	8.142	503006	15.54
2	14.183	2734798	84.46

Dimethyl 2-{1-(4-cyanophenyl)-2-[1-(naphthalen-2-yl)-1H-tetrazol-5-yl]-2-(naphthalen-2-ylimino)ethyl}malonate (5q)

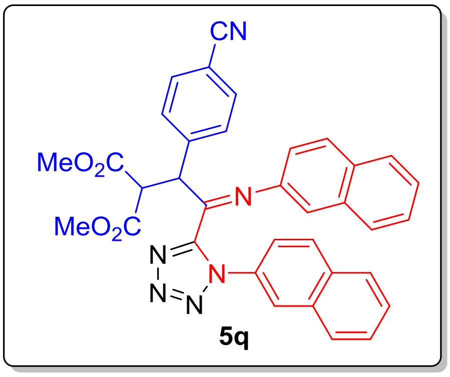

Yellow solid; m.p. 68-72 °C; 97% yield, 95:5 e.r.; $[\alpha]^{26}_{D}$ = –199.4 (*c* = 1.07 in CH_2Cl_2).

HPLC DAICEL CHIRALCEL IA, *n*-hexane/2-propanol = 70/30, flow rate = 1.0 mL/min, λ = 330 nm, t_R (minor) = 10.87 min, t_R (major) = 24.29 min.

IR (neat): 1744, 1508, 1437, 1302, 1267, 1150, 1030, 856, 816 and 746 cm^{-1}.

^{1}H NMR (400 MHz, $CDCl_3$) δ = 7.77 – 7.42 (m, 9H), 7.39 – 7.32 (m, 2H), 7.30 – 7.23 (m, 1H), 7.09 (t, *J* = 9.6 Hz, 2H), 6.83 (s, 1H), 6.30 (s, 1H), 6.28 – 6.18 (m, 1H), 6.14 – 6.96 (m, 1H), 5.35 (d, *J* = 11.6 Hz, 1H), 4.76 (d, *J* = 11.6 Hz, 1H), 3.84 (s, 3H), 3.54 (s, 3H).

^{13}C{^{1}H} NMR (101 MHz, $CDCl_3$) δ = 168.1, 167.4, 154.4, 149.2, 144.7, 140.6, 133.2, 132.9, 132.4, 131.2, 130.4, 130.1, 129.2, 128.8, 128.1, 127.8, 127.7, 127.5, 127.4, 126.7, 126.0, 122.8, 120.6, 119.0, 118.3, 116.7, 112.8, 55.7, 54.1, 53.3, 53.0.

HRMS (ESI-FT) calcd for $C_{35}H_{27}N_6O_4^+$ ([M+H$^+$]) = 595.2088, Found 595.2082.

Chiral HPLC spectrum **5q**:

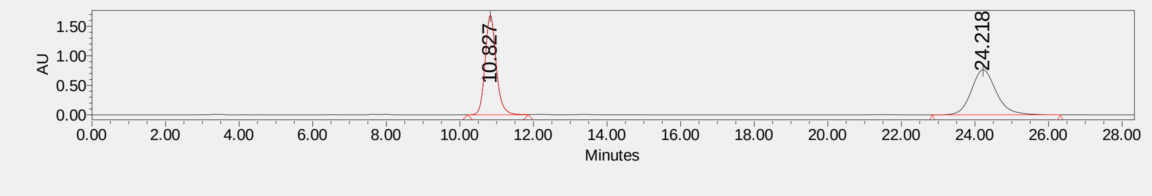

	Retention Time	Area	% Area
1	10.827	35244053	49.59
2	24.218	35825609	50.41

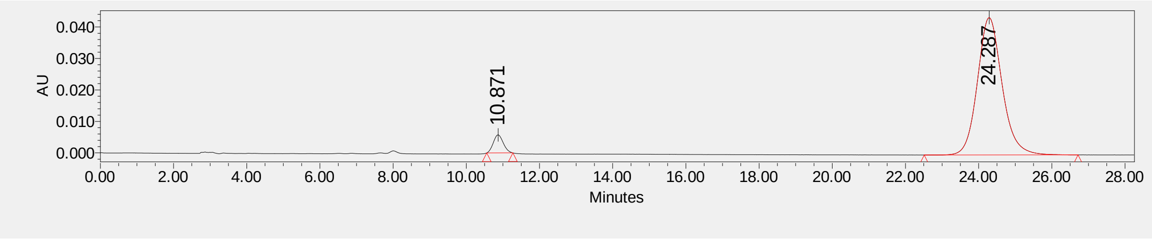

	Retention Time	Area	% Area
1	10.871	108113	5.03
2	24.287	2039561	94.97

Dimethyl 2-{1-(4-chlorophenyl)-2-[1-(naphthalen-2-yl)-1H-tetrazol-5-yl]-2-(naphthalen-2-ylimino)ethyl}malonate (5r)

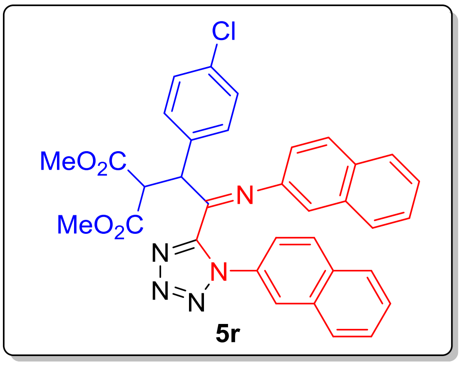

Yellow solid; m.p. 56-60 °C; 98% yield, 94:6 e.r.; $[\alpha]^{26}_D$ = –266.4 (*c* = 0.93 in CH_2Cl_2).

HPLC DAICEL CHIRALCEL IA, *n*-hexane/2-propanol = 70/30, flow rate = 1.0 mL/min, λ = 330 nm, t_R (minor) = 7.76 min, t_R (major) = 13.78 min.

IR (neat): 1744, 1649, 1495, 1437, 1302, 1265, 1096, 1024, 858, 814 and 745 cm^{-1}.

^{1}H NMR (400 MHz, $CDCl_3$) δ = 7.73 – 7.67 (m, 1H), 7.60 – 7.50 (m, 2H), 7.49 – 7.33 (m, 6H), 7.31 – 7.19 (m, 3H), 7.11 (t, *J* = 9.2 Hz, 2H), 6.56 (s, 1H), 6.38 – 6.25

(m, 1H), 6.22 – 5.94 (m, 2H), 5.34 (d, *J* = 11.6 Hz, 1H), 4.77 (d, *J* = 11.6 Hz, 1H), 3.79 (s, 3H), 3.56 (s, 3H).

$^{13}C\{^1H\}$ NMR (101 MHz, $CDCl_3$) δ = 168.2, 167.7, 155.3, 149.5, 144.9, 135.0, 133.4, 133.3, 133.2, 132.4, 131.2, 130.9, 130.2, 129.7, 129.2, 128.7, 128.1, 127.8, 127.7, 127.6, 127.5, 127.3, 126.6, 125.9, 122.8, 120.8, 119.3, 116.7, 55.5, 53.7, 53.2, 52.9.

HRMS (ESI-FT) calcd for $C_{34}H_{27}{}^{35}ClN_5O_4^+$ ([M+H$^+$]) = 604.1746, Found 604.1741.

HRMS (ESI-FT) calcd for $C_{34}H_{27}{}^{37}ClN_5O_4^+$ ([M+H$^+$]) = 606.1717, Found 606.1721.

Chiral HPLC spectrum **5r**:

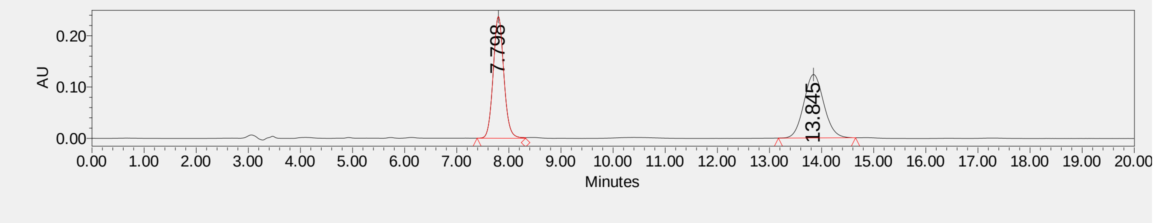

	Retention Time	Area	% Area
1	7.798	3354782	50.41
2	13.845	3300398	49.59

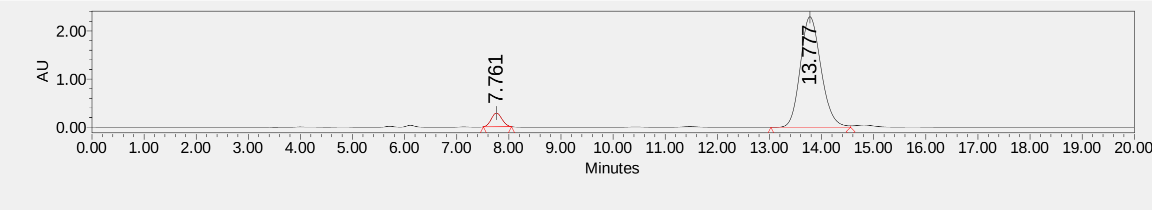

	Retention Time	Area	% Area
1	7.761	4020600	5.95
2	13.777	63495850	94.05

Dimethyl 2-{1-(3-chlorophenyl)-2-[1-(naphthalen-2-yl)-1H-tetrazol-5-yl]-2-(naphthalen-2-ylimino)ethyl}malonate (5s)

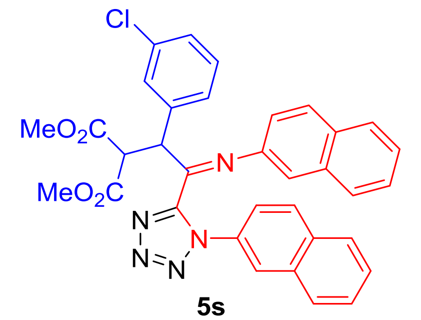

5s

Yellow solid; m.p. 46-48 °C; 95% yield, 92:8 e.r.; $[\alpha]^{26}_{D}$ = –238.3 (*c* = 1.00 in CH_2Cl_2).

HPLC DAICEL CHIRALCEL IA, *n*-hexane/2-propanol = 70/30, flow rate = 1.0 mL/min, λ = 330 nm, t_R (minor) = 5.96 min, t_R (major) = 10.32 min.

IR (neat): 1744, 1649, 1495, 1435, 1298, 1265, 1150, 1030, 815, 748 and 696 cm^{-1}.

^{1}H NMR (400 MHz, $CDCl_3$) δ = 7.72 – 7.67 (m, 1H), 7.60 – 7.50 (m, 3H), 7.48 – 7.30 (m, 6H), 7.28 – 7.19 (m, 2H), 7.09 (t, *J* = 8.8 Hz, 2H), 6.60 – 6.50 (m, 1H), 6.39 – 6.24 (m, 1H), 6.21 – 5.95 (m, 2H), 5.39 (d, *J* = 11.6 Hz, 1H), 4.76 (d, *J* = 11.6 Hz, 1H), 3.78 (s, 3H), 3.58 (s, 3H).

^{13}C{^{1}H} NMR (101 MHz, $CDCl_3$) δ = 168.1, 167.7, 155.1, 149.3, 144.9, 136.9, 135.3, 133.3, 133.2, 132.4, 131.2, 130.7, 130.2, 129.2, 128.7, 128.2, 127.8, 127.7, 127.4, 127.3, 126.6, 125.9, 122.7, 120.7, 119.2, 116.7, 55.4, 53.9, 53.2, 52.9.

HRMS (ESI-FT) calcd for $C_{34}H_{27}{}^{35}ClN_5O_4^+$ ($[M+H^+]$) = 604.1746, Found 604.1744.

HRMS (ESI-FT) calcd for $C_{34}H_{27}{}^{37}ClN_5O_4^+$ ($[M+H^+]$) = 606.1717, Found 606.1724.

Chiral HPLC spectrum **5s**:

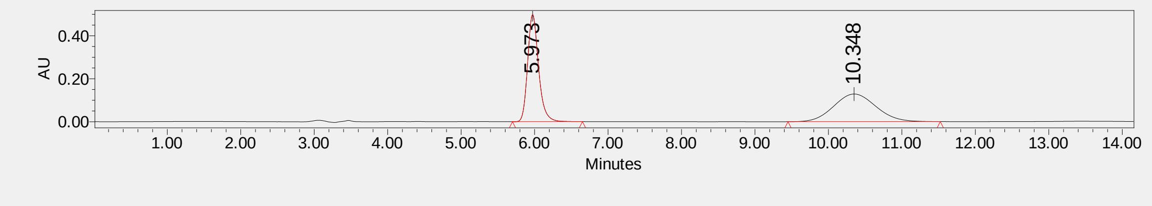

	Retention Time	Area	% Area
1	5.973	5154938	50.05
2	10.348	5145665	49.95

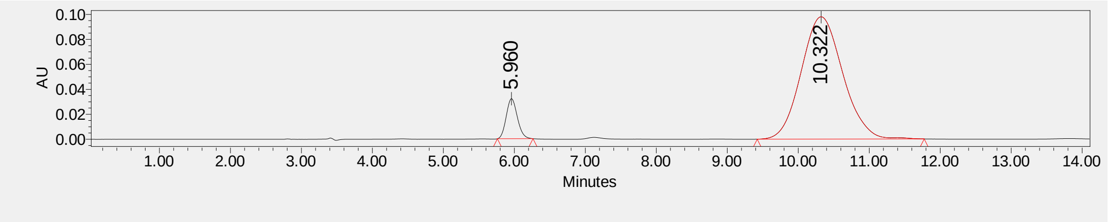

	Retention Time	Area	% Area
1	5.960	348494	8.05
2	10.322	3983263	91.95

Dimethyl 2-{1-(2-chlorophenyl)-2-[1-(naphthalen-2-yl)-1H-tetrazol-5-yl]-2-(naphthalen-2-ylimino)ethyl}malonate (5t)

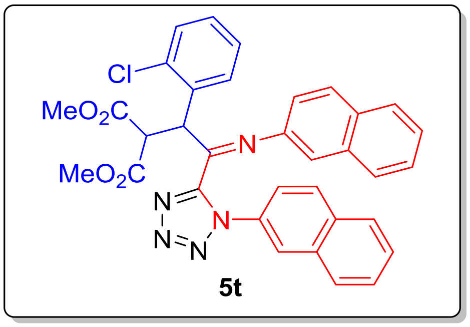

5t

Yellow solid; m.p. 46-48 °C; 62% yield, 76.5:23.5 e.r.; $[\alpha]^{26}_{D}$ = –133.30 (c = 0.87 in CH_2Cl_2).

HPLC DAICEL CHIRALCEL IA, *n*-hexane/2-propanol = 70/30, flow rate = 1.0 mL/min, λ = 330 nm, t_R (minor) = 6.28 min, t_R (major) = 8.84 min.

IR (neat): 1742, 1649, 1512, 1437, 1300, 1265, 1148, 1036, 860, 815 and 750 cm^{-1}.

^{1}H NMR (400 MHz, $CDCl_3$) δ = 7.75 – 7.30 (m, 11H), 7.29 – 7.21 (m, 1H), 7.16 – 7.07 (m, 1H), 7.00 – 6.12 (m, 1H), 6.40 – 5.89 (m, 4H), 4.97 – 4.66 (m, 1H), 3.82 (s, 3H), 3.56 (s, 3H).

^{13}C{^{1}H} NMR (101 MHz, $CDCl_3$) δ = 168.3, 167.5, 149.1, 144.9, 135.9, 133.3, 133.2, 132.5, 131.2, 130.7, 130.3, 130.0, 129.7, 129.2, 128.7, 128.3, 127.7, 127.6, 127.5, 127.3, 126.6, 125.8, 122.7, 120.9, 119.3, 116.8, 100.1, 55.7, 53.1, 52.8, 49.2.

HRMS (ESI-FT) calcd for $C_{34}H_{27}{}^{35}ClN_5O_4^+$ ([M+H$^+$]) = 604.1746, Found 604.1742.

HRMS (ESI-FT) calcd for $C_{34}H_{27}{}^{37}ClN_5O_4^+$ ([M+H$^+$]) = 606.1717, Found 606.1722.

Chiral HPLC spectrum **5t**:

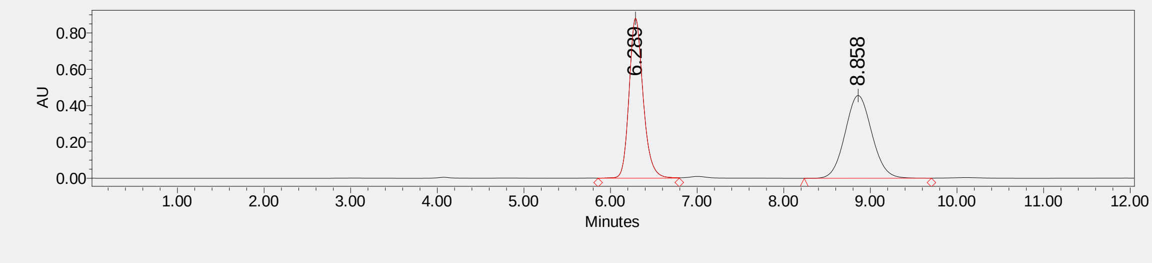

	Retention Time	Area	% Area
1	5.960	338051	8.03
2	10.321	3870204	91.97

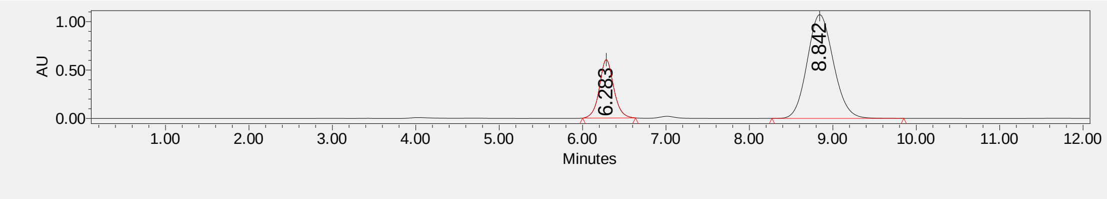

	Retention Time	Area	% Area
1	6.283	7197018	23.55
2	8.842	23357130	76.45

Dimethyl 2-{1-(3,4-dichlorophenyl)-2-[1-(naphthalen-2-yl)-1H-tetrazol-5-yl]-2-(naphthalene-2-ylimino)ethyl}malonate (5u)

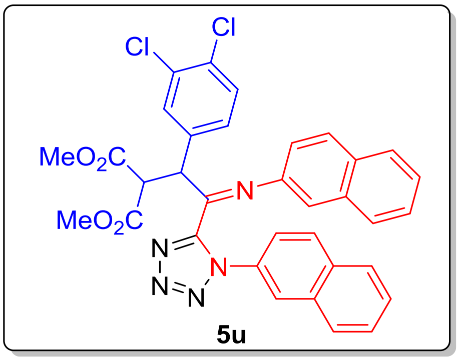

Yellow solid; m.p. 48-52 °C; 92% yield, 93:7 e.r.; $[\alpha]^{26}_{D}$ = –221.0 (c = 0.91 in CH_2Cl_2).

HPLC DAICEL CHIRALCEL IA, *n*-hexane/2-propanol = 70/30, flow rate = 1.0 mL/min, λ = 330 nm, t_R (minor) = 6.92 min, t_R (major) = 12.08 min.

IR (neat): 1744, 1649, 1510, 1467, 1296, 1265, 1143, 1031, 860, 816 and 745 cm^{-1}.

^{1}H NMR (400 MHz, $CDCl_3$) δ = 7.75 – 7.68 (m, 1H), 7.66 – 7.61 (m, 1H), 7.60 – 7.43 (m, 4H), 7.40 – 7.29 (m, 3H), 7.29 – 7.22 (m, 2H), 7.11 (t, *J* = 8.4 Hz, 2H), 6.71 (s, 1H), 6.42 – 6.28 (m, 1H), 6.26 – 6.00 (m, 2H), 5.31 (d, *J* = 11.6 Hz, 1H), 4.71 (d, *J* = 11.6 Hz, 1H), 3.80 (s, 3H), 3.59 (s, 3H).

^{13}C{^{1}H} NMR (101 MHz, $CDCl_3$) δ = 168.0, 167.5, 154.7, 149.3, 144.8, 135.3, 133.5, 133.3, 133.2, 132.4, 131.3, 131.1, 130.2, 129.3, 128.8, 128.2, 127.8, 127.7, 127.6, 127.5, 127.4, 126.7, 126.0, 122.8, 120.7, 119.2, 116.8, 55.7, 53.3, 53.2, 53.0.

HRMS (ESI-FT) calcd for $C_{34}H_{26}{}^{35}Cl_2N_5O_4^+$ ([M+H$^+$]) = 638.1356, Found 638.1351.

HRMS (ESI-FT) calcd for $C_{34}H_{26}{}^{35}Cl^{37}ClN_5O_4^+$ ([M+H$^+$]) = 640.1327, Found 640.1328.

HRMS (ESI-FT) calcd for $C_{34}H_{26}{}^{37}Cl_2N_5O_4^+$ ([M+H$^+$]) = 642.1297, Found 642.1313.

Chiral HPLC spectrum **5u**:

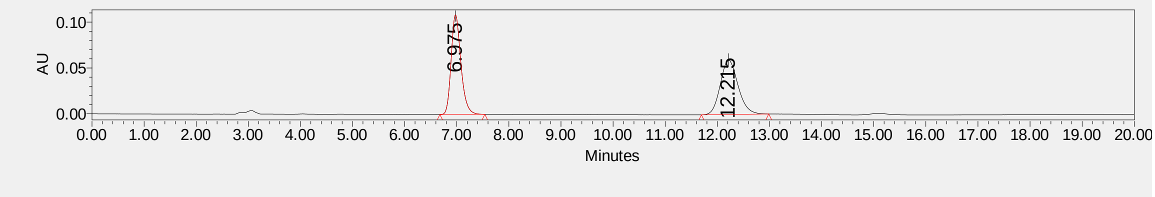

	Retention Time	Area	% Area
1	6.975	1416101	50.29
2	12.215	1400017	49.71

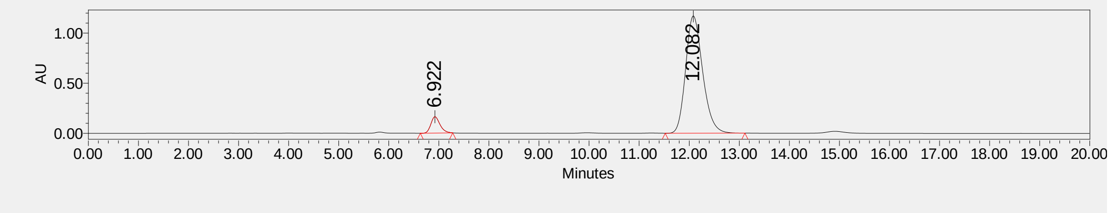

	Retention Time	Area	% Area
1	6.922	2069486	6.96
2	12.082	27659934	93.04

Dimethyl 2-{2-[1-(naphthalen-2-yl)-1H-tetrazol-5-yl]-2-(naphthalen-2-ylimino)-1-(*m*-tolyl) ethyl}malonate (5v)

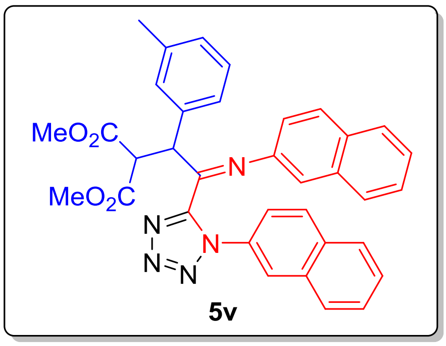

5v

Yellow solid; m.p. 44-46 ^{o}C; 84% yield, 89:11 e.r.; $[\alpha]^{26}_{D}$ = -227.1 (c = 0.83 in CH_2Cl_2).

HPLC DAICEL CHIRALCEL IA, n-hexane/2-propanol = 70/30, flow rate = 1.0 mL/min, λ = 330 nm, t_R (minor) = 5.63 min, t_R (major) = 10.29 min.

IR (neat): 1744, 1649, 1512, 1437, 1300, 1267, 1150, 1034, 860, 815, 750 and 705 cm^{-1}.

^{1}H NMR (400 MHz, $CDCl_3$) δ = 7.71 – 7.65 (m, 1H), 7.59 – 7.49 (m, 2H), 7.46 – 7.28 (m, 5H), 7.26 – 7.14 (m, 4H), 7.09 (t, *J* = 7.6 Hz, 2H), 6.47 – 6.18 (m, 2H), 6.18 – 6.04 (m, 1H), 6.01 – 5.85 (m, 1H), 5.37 (d, *J* = 12.0 Hz, 1H), 4.83 (d, *J* = 12.0 Hz, 1H), 3.77 (s, 3H), 3.55 (s, 3H), 2.38 (s, 3H).

^{13}C{^{1}H} NMR (101 MHz, $CDCl_3$) δ = 168.3, 167.9, 156.0, 149.6, 145.2, 139.3, 134.5, 133.4, 133.1, 132.4, 131.0, 130.2, 129.7, 129.4, 129.0, 128.6, 128.2, 127.7, 127.6, 127.5, 127.4, 127.2, 126.5, 125.7, 122.7, 120.8, 119.3, 116.6, 55.2, 54.3, 53.1, 52.7, 21.6.

HRMS (ESI-FT) calcd for $C_{35}H_{30}N_5O_4^+$ ([M+H$^+$]) = 584.2292, Found 584.2291.

Chiral HPLC spectrum **5v**:

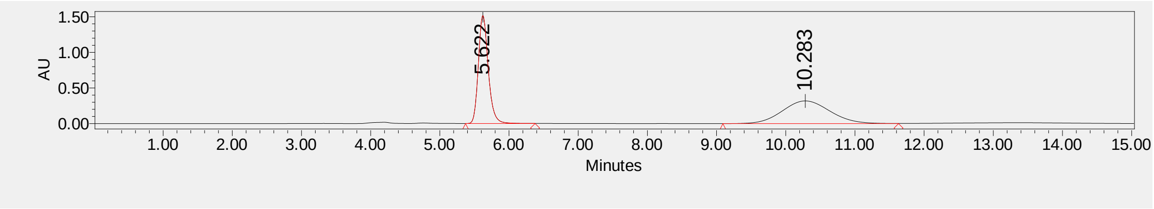

	Retention Time	Area	% Area
1	5.622	15053800	49.64
2	10.283	15271756	50.36

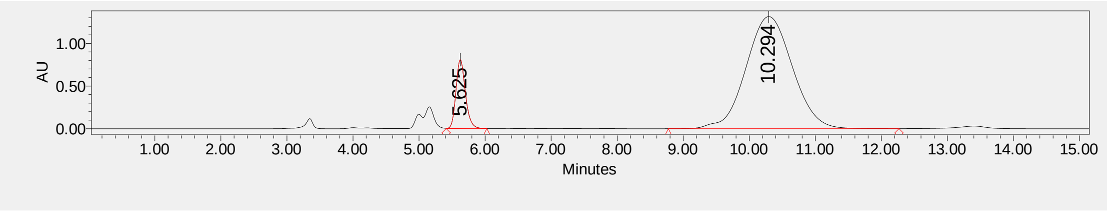

	Retention Time	Area	% Area
1	5.625	7991841	11.06
2	10.294	64293561	88.94

Dimethyl 2-{1-(naphthalen-2-yl)-2-[1-(naphthalen-2-yl)-1H-tetrazol-5-yl]-2-(naphthalen-2-ylimino)ethyl}malonate (5w)

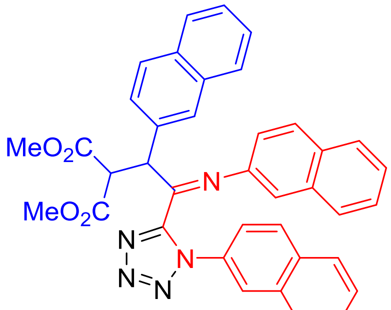

5w

Yellow solid; m.p. 56-60 °C; 97% yield, 92.5:7.5 e.r.; $[\alpha]^{26}_{D}$ = –218.3 (*c* = 0.84 in CH_2Cl_2).

HPLC DAICEL CHIRALCEL IA, *n*-hexane/2-propanol = 70/30, flow rate = 1.0 mL/min, λ = 330 nm, t_R (minor) = 8.51 min, t_R (major) = 14.47 min.

IR (neat): 1744, 1597, 1510, 1437, 1300, 1267, 1153, 1028, 893, 860, 815 and 750 cm^{-1}.

^{1}H NMR (400 MHz, $CDCl_3$) δ = 8.00 – 7.19 (m, 26H), 7.16 – 7.01 (m, 2H), 7.01 – 6.86 (m, 2H), 6.41 – 6.32 (m, 1H), 6.25 (s, 1H), 6.20 – 6.12 (m, 1H), 5.92 – 5.80 (m, 1H), 5.58 (d, *J* = 11.6 Hz, 1H), 5.05 – 4.93 (m, 1.62H), 4.90 – 4.85 (m, 0.62H), 3.80 (s, 3H), 3.72 (s, 2H), 3.48 (s, 3H), 3.35 (s, 2H).

^{13}C{^{1}H} NMR (101 MHz, $CDCl_3$) δ = 168.3, 167.9, 167.2, 155.9, 155.8, 149.7, 145.2, 133.8, 133.7, 133.4, 133.3, 133.1, 132.9, 132.2, 132.0, 131.9, 131.1, 130.7, 130.2, 130.1, 129.3, 129.2, 129.1, 129.0, 128.7, 128.5, 128.4, 128.3, 128.2, 128.1, 128.0, 127.8, 127.7, 127.6, 127.6, 127.4, 127.1, 127.0, 126.9, 126.8, 126.5, 125.8, 125.3, 122.9, 122.6, 120.8, 119.4, 116.7, 57.0, 55.4, 54.5, 53.3, 53.1, 52.8, 40.9.

HRMS (ESI-FT) calcd for $C_{38}H_{30}N_5O_4^+$ ([M+H$^+$]) = 620.2292, Found 620.2289.

Chiral HPLC spectrum **5w**:

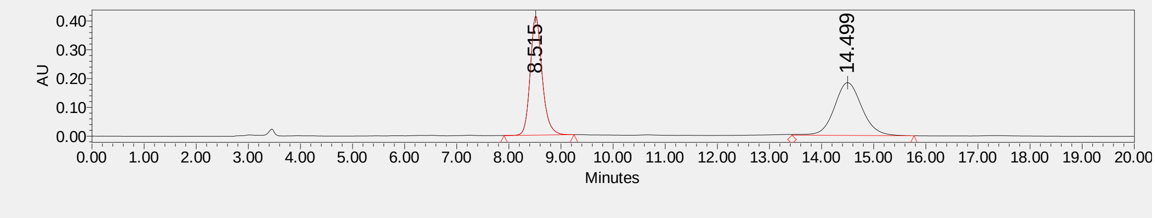

	Retention Time	Area	% Area
1	8.515	6567129	49.27
2	14.499	6762011	50.73

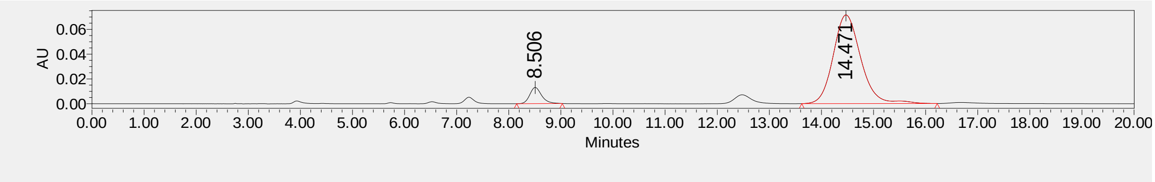

	Retention Time	Area	% Area
1	8.506	208683	7.35
2	14.471	2628949	92.65

Dimethyl 2-{1-(4-cyanophenyl)-2-[1-(4-methoxyphenyl)-1H-tetrazol-5-yl]-2-[(4-methoxyphenyl)imino]ethyl}malonate (5x)

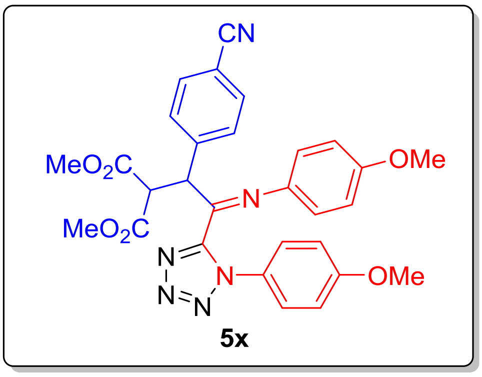

Yellow solid; m.p. 66-70 °C; 92% yield, 93:7 e.r.; $[\alpha]^{21}_{D}$ = –237.05 (*c* = 0.89 in CH_2Cl_2).

HPLC DAICEL CHIRALCEL IA, *n*-hexane/2-propanol = 70/30, flow rate = 1.0 mL/min, λ = 254 nm, t_R (minor) = 10.32 min, t_R (major) = 25.23 min.

IR (neat): 2956, 1738, 1606, 1517, 1437, 1302, 1275, 1251, 1181, 1029, 833 and 750 cm^{-1}.

^{1}H NMR (400 MHz, $CDCl_3$) δ = 7.68 – 7.58 (m, 2H), 7.52 – 7.46 (m, 2H), 6.64 – 6.58 (m, 1H), 6.54 – 6.47 (m, 2H), 6.41 – 6.30 (m, 2H), 6.14 – 6.04 (m, 2H), 5.14 (d, J = 12.0 Hz, 1H), 4.66 (d, J = 11.6 Hz, 1H), 3.79 (s, 3H), 3.77 (s, 3H), 3.70 (s, 3H), 3.50 (s, 3H).

^{13}C{^{1}H} NMR (101 MHz, $CDCl_3$) δ = 167.9, 167.5, 160.9, 158.1, 152.7, 149.1, 140.6, 132.8, 130.3, 125.8, 125.1, 121.5, 118.4, 114.3, 114.2, 112.6, 55.8, 55.6, 55.5, 54.1, 53.2, 52.9.

HRMS (ESI-FT) calcd for $C_{29}H_{27}N_6O_6^+$ ([M+H$^+$]) = 555.1987, Found 555.1989.

Chiral HPLC spectrum **5x**:

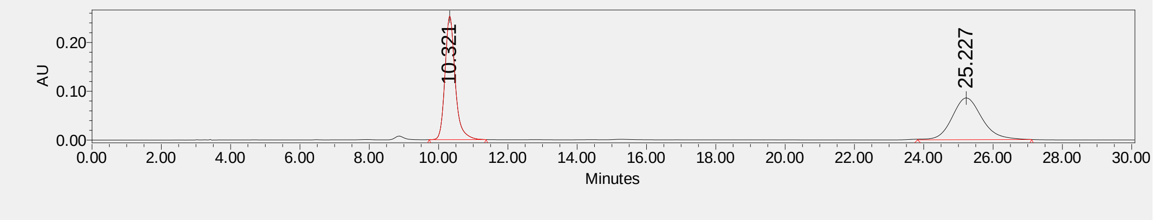

	Retention Time	% Area	Height
1	10.321	50.12	252751
2	25.227	49.88	85576

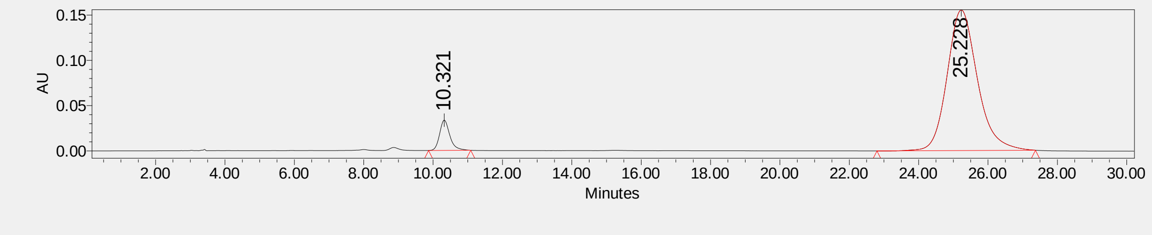

	Retention Time	% Area	Height
1	10.321	6.80	33482
2	25.228	93.20	155299

Dimethyl 2-{1-[1-(4-methoxyphenyl)-1H-tetrazol-5-yl]-1-[(4-methoxyphenyl)imino]-3,3-dimethylbutan-2-yl}malonate (5z)

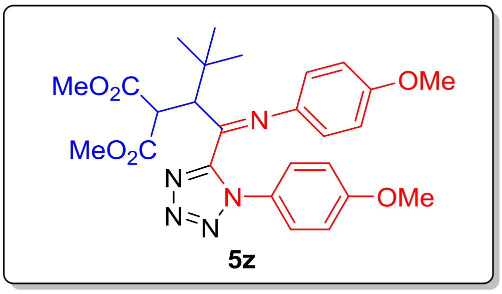

Yellow solid; m.p. 38-42 ^{o}C; 91% yield, 85:15 e.r.; $[\alpha]^{21}_{D}$ = –352.99 (*c* = 0.34 in CH_2Cl_2).

HPLC DAICEL CHIRALCEL IC, *n*-hexane/2-propanol = 70/30, flow rate = 1.0 mL/min, λ = 254 nm, t_R (minor) = 15.53 min, t_R (major) = 19.39 min.

IR (neat): 2956, 1754, 1734, 1606, 1516, 1504, 1466, 1437, 1276, 1251, 1168, 1030, 832 and 749 cm^{-1}.

^{1}H NMR (400 MHz, $CDCl_3$) δ = 7.49 – 7.43 (m, 1.36H), 7.04 – 6.94 (m, 3.55H), 6.88 (s, 2.58H), 6.77 – 6.72 (m, 2H), 6.53 – 6.44 (m, 2H), 6.22 – 6.13 (m, 2H), 4.65 (d, *J* =

10.4 Hz, 0.68H), 4.23 – 4.06 (m, 2.75H), 3.90 (s, 2H), 3.83 (s, 3H), 3.80 (s, 2H), 3.77 (s, 4H), 3.72 (s, 3H), 3.71 (s, 3H), 3.61 (s, 3H), 1.10 (s, 8H), 0.60 (s, 6H).

^{13}C{^{1}H} NMR (101 MHz, $CDCl_3$) δ = 170.5, 169.9, 169.5, 160.8, 160.6, 157.4, 157.0, 155.5, 155.1, 152.9, 150.5, 142.0, 141.4, 128.7, 128.2, 127.6, 127.2, 125.8, 121.3, 120.8, 114.2, 113.8, 56.4, 55.8, 55.56, 55.5, 54.0, 53.3, 53.0, 52.8, 52.7, 52.5, 48.6, 35.9, 35.4, 28.6, 28.5, 27.7.

HRMS (ESI-FT) calcd for $C_{26}H_{32}N_5O_6^+$ ([M+H$^+$]) = 510.2347, Found 510.2343.

Chiral HPLC spectrum **5z**:

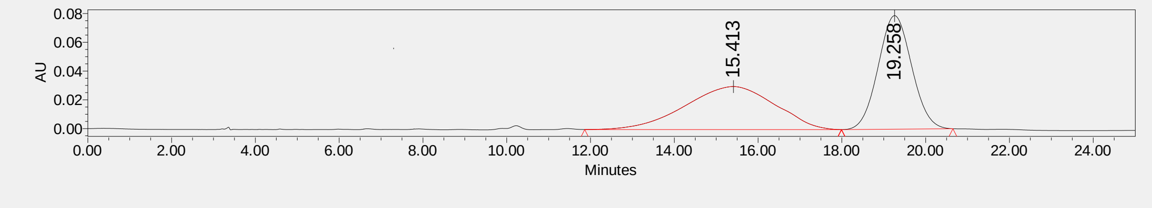

	Retention Time	% Area	Height
1	15.413	50.29	29894
2	19.258	49.71	78921

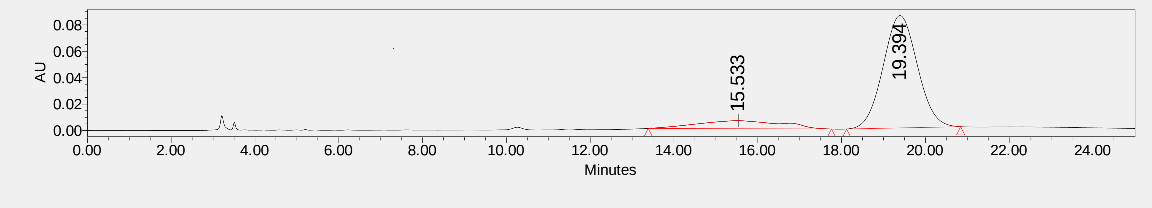

	Retention Time	% Area	Height
1	15.533	15.10	6126
2	19.394	84.90	85055

Dimethyl 2-{(2*S*,4*S*)-4,8-dimethyl-1-[1-(naphthalen-2-yl)-1H-tetrazol-5-yl]-1-(naphthalen-2-ylimino)non-7-en-2-yl)malonate (5y)

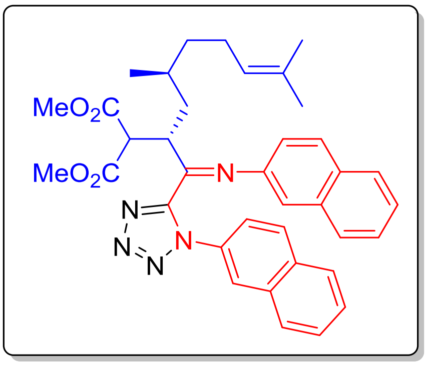

5y: 96% yield, 2.0:1 d.r.
ent-**L-RaPr$_2$**: 95% yield, 1:4.0 d.r.

Yellow oil; 96% (95%) yield, 2.0:1 (1:4) d.r.; $[\alpha]^{21}_{D}$ = –66.59 (+135.66) (*c* = 0.34 in CH_2Cl_2).

IR (neat): 2988, 1734, 1625, 1596, 1505, 1437, 1411, 1275, 1258, 764 and 750 cm^{-1}.

^{1}H NMR (400 MHz, $CDCl_3$) δ = 8.14 – 8.06 (m, 0.46H), 7.95 – 7.83 (m, 1.45H), 7.77 – 7.63 (m, 3H), 7.58 – 7.43 (m, 5.47H), 7.41 – 7.28 (m, 3.75H), 7.24 – 7.18 (m, 1H), 7.14 – 7.09 (m, 0.50H), 7.06 – 6.98 (m, 2H), 6.94 – 6.87 (m, 0.47H), 6.78 – 6.70 (m, 1H), 6.30 – 6.23 (m, 1H), 6.11 – 6.03 (m, 1H), 5.14 – 5.07 (m, 0.70H), 5.05 – 5.00 (m 0.35H), 4.91 – 4.84 (m, 0.15H), 4.69 – 4.56 (m, 0.5H), 4.42 – 4.35 (m, 0.32H), 4.32 – 4.22 (m, 1H), 4.21 – 4.12 (m, 1H), 3.81 – 3.68 (m, 9H), 2.33 – 2.21 (m, 0.5H), 2.09 – 1.91 (m, 4H), 1.82 – 1.72 (m, 1H), 1.70 – 1.41 (m, 12H), 1.09 (d, *J* = 6.4 Hz, 1.26H), 1.02 (d, *J* = 6.4 Hz, 2.70H), 0.62 (d, *J* = 6.4 Hz, 0.8H), 0.17 (d, *J* = 5.2 Hz, 0.35H)

^{13}C{^{1}H} NMR (101 MHz, $CDCl_3$) δ = 169.2, 169.1, 169.0, 157.4, 157.3, 149.7, 149.5, 145.3, 133.2, 133.2, 132.6, 131.7, 131.6, 131.0, 130.9, 130.7, 129.2, 129.1, 128.9, 128.4, 128.0, 127.8, 127.7, 127.6, 127.5, 127.4, 127.3, 127.2, 126.6, 126.4, 125.6, 125.2, 124.8, 124.6, 124.4, 123.4, 122.9, 120.8, 119.3, 116.3, 115.3, 55.0, 54.4, 52.8, 46.2, 46.2, 38.9, 38.8, 37.7, 37.0, 30.6, 30.4, 30.3, 25.9, 25.7, 25.5, 25.4, 25.2, 19.9, 19.8, 19.5, 17.8, 17.7.

HRMS (ESI-FT) calcd for $C_{37}H_{40}N_5O_4^+$ ([M+H$^+$]) = 618.3075, Found 618.3070.

Dimethyl (2*S*,10b*S*,*E*)-3-(tert-butylimino)-2-phenyl-2,3-dihydropyrrolo[2,1-a]isoquinoline-1,1(10bH)-dicarboxylate (8a)

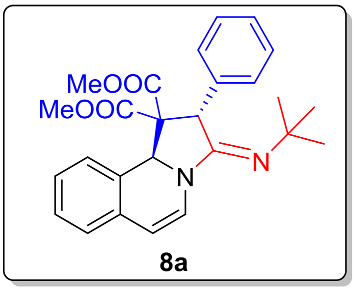

White solid; m.p. 134-138 °C; 85% yield, 96.5:3.5 e.r.; $[\alpha]^{25}_{D}$ = +213.6 (*c* = 0.55 in CH_2Cl_2).

HPLC DAICEL CHIRALCEL IA, *n*-hexane/2-propanol = 90/10, flow rate = 1.0

mL/min, λ = 350 nm, t_R (major) = 4.14 min, t_R (minor) = 4.76 min.

IR (neat): 2963, 1738, 1672, 1630, 1450, 1352, 1304, 1267, 1211, 1072, 775 and 709 cm^{-1}.

^{1}H NMR (400 MHz, $CDCl_3$) δ = 7.33 – 7.27 (m, 3H), 7.23 – 7.22 (m, 1), 7.20 – 7.13 (m, 3H), 7.12 – 7.06 (m, 1H), 7.01 – 6.95 (m, 1H), 6.94 – 6.89 (m, 1H), 5.96 (s, 1H), 5.63 (d, *J* = 7.6 Hz, 1H), 4.68 (s, 1H), 3.53 (d, *J* = 5.2 Hz, 6H), 1.08 (s, 9H).

^{13}C{^{1}H} NMR (101 MHz, $CDCl_3$) δ = 169.3, 168.3, 151.3, 135.0, 133.6, 128.8, 128.4, 128.0, 127.2, 126.8, 125.6, 124.8, 124.6, 105.7, 67.1, 59.0, 53.7, 53.2, 52.7, 52.5, 31.7.

HRMS (ESI-FT) calcd for $C_{26}H_{29}N_2O_4^+$ ([M+H$^+$]) = 433.2122, Found 433.2120.

Chiral HPLC spectrum **8a**:

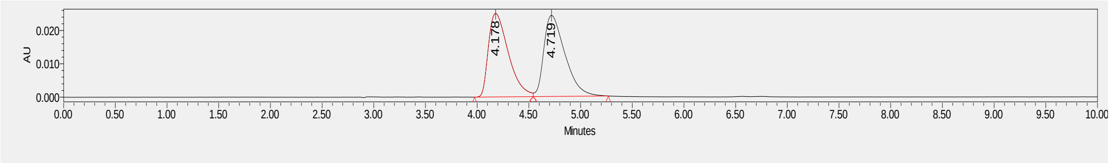

	Retention Time	Area	% Area
1	4.178	336377	49.25
2	4.719	346669	50.75

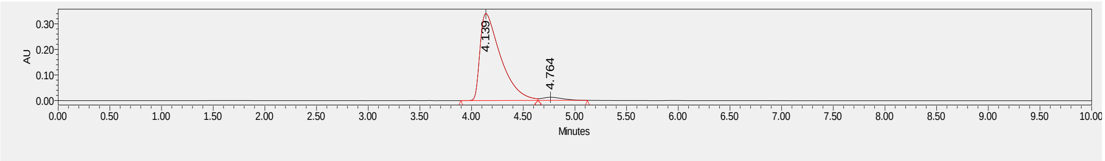

	Retention Time	Area	% Area
1	4.139	4743021	96.49
2	4.764	172367	3.51

Diethyl (2*S*,10b*S*,*E*)-3-(tert-butylimino)-2-phenyl-2,3-dihydropyrrolo[2,1-a]isoquinoline-1,1(10bH)-dicarboxylate (8b)

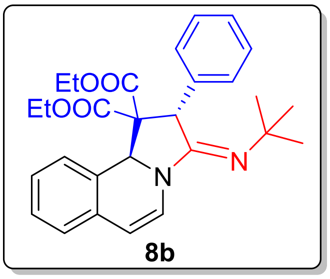

Pale yellow oil; 86% yield, 94.5:5.5 e.r.; $[\alpha]^{25}_D$ = +115.16 (*c* = 0.49 in CH_2Cl_2).

HPLC DAICEL CHIRALCEL ODH, *n*-hexane/2-propanol = 90/10, flow rate = 1.0 mL/min, λ = 350 nm, t_R (major) = 3.58 min, t_R (minor) = 3.93 min.

IR (neat): 2972, 2930, 1736, 1672, 1630, 1458, 1406, 1304, 1263, 1204, 1070, 773 and 708 cm^{-1}.

^{1}H NMR (400 MHz, $CDCl_3$) δ = 7.31 – 7.25 (m, 3H), 7.24 – 7.21 (m, 2H), 7.20 – 7.16 (m, 2H), 7.11 – 7.05 (m, 1H), 7.00 – 6.94 (m, 1H), 6.92 – 6.87 (m, 1H), 6.00 (s, 1H), 5.60 (d, *J* = 7.8 Hz, 1H), 4.68 (s, 1H), 4.10 – 3.83 (m, 4H), 1.15 (t, *J* = 7.2 Hz, 3H), 1.08 (s, 9H), 0.95 (t, *J* = 7.2 Hz, 3H).

^{13}C{^{1}H} NMR (101 MHz, $CDCl_3$) δ = 168.9, 167.9, 151.4, 135.1, 133.6, 128.8, 128.3, 128.0, 127.4, 127.3, 125.4, 124.9, 124.5, 105.2, 66.9, 62.0, 61.7, 59.0, 53.7, 53.0, 31.8, 13.9, 13.8.

HRMS (ESI-FT) calcd for $C_{28}H_{33}N_2O_4^+$ ([M+H$^+$]) = 461.2435, Found 461.2433.

Chiral HPLC spectrum **8b**:

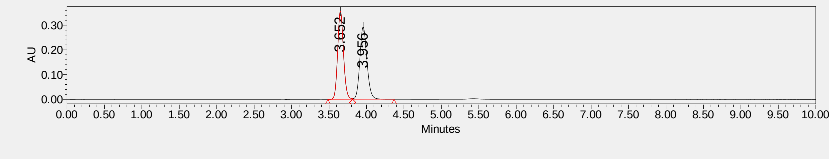

	Retention Time	Area	% Area
1	3.652	1931974	49.95
2	3.956	1936019	50.05

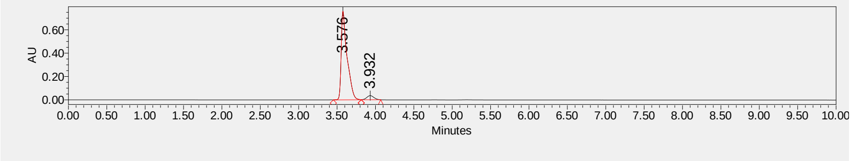

	Retention Time	Area	% Area
1	3.576	4040615	94.56
2	3.932	232508	5.44

Dimethyl (2*S*,10b*S*,*E*)-2-(4-bromophenyl)-3-(tert-butylimino)-2,3-dihydropyrrolo[2,1-a] isoquinoline-1,1(10bH)-dicarboxylate (8c)

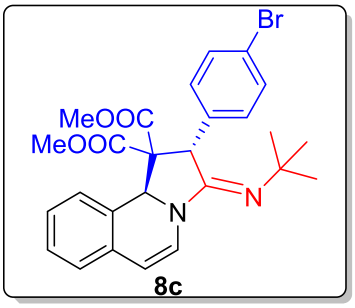

Pale yellow oil; 79% yield, 95:5 e.r.; $[\alpha]^{25}_{D}$ = +98.28 (c = 0.35 in CH_2Cl_2).

HPLC DAICEL CHIRALCEL IA, *n*-hexane/2-propanol = 90/10, flow rate = 1.0 mL/min, λ = 350 nm, t_R (major) = 4.19 min, t_R (minor) = 4.86 min.

IR (neat): 2965, 2858, 1738, 1672, 1630, 1449, 1406, 1435, 1271, 1211, 1072, 943, 773 and 735 cm^{-1}.

^{1}H NMR (400 MHz, $CDCl_3$) δ = 7.47 – 7.41 (m, 2H), 7.23 – 7.17 (m, 2H), 7.13 – 7.08 (m, 1H), 7.06 – 7.03 (m, 2H), 7.02 – 6.97 (m, 1H), 6.95 – 6.90 (m, 1H), 5.90 (s, 1H), 5.64 (d, J = 7.8 Hz, 1H), 4.63 (s, 1H), 3.57 (s, 3H), 3.53 (s, 3H), 1.08 (s, 9H).

^{13}C{^{1}H} NMR (101 MHz, $CDCl_3$) δ = 169.1, 168.1, 150.7, 134.2, 133.5, 132.1, 128.2, 127.0, 126.8, 125.7, 124.7, 124.6, 122.5, 105.9, 66.9, 58.9, 53.8, 52.8, 52.6, 52.5, 31.8.

HRMS (ESI-FT) calcd for $C_{26}H_{28}{}^{79}BrN_2O_4^+$ ($[M+H^+]$) = 511.1227, Found 511.1228, $C_{26}H_{28}{}^{81}BrN_2O_4^+$ ($[M+H^+]$) = 513.1206, Found 513.1208.

Chiral HPLC spectrum **8c**:

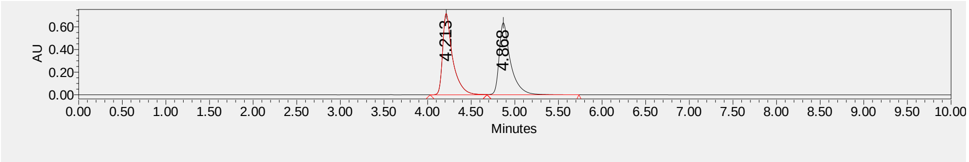

	Retention Time	Area	% Area
1	4.213	5760293	49.84
2	4.868	5797686	50.16

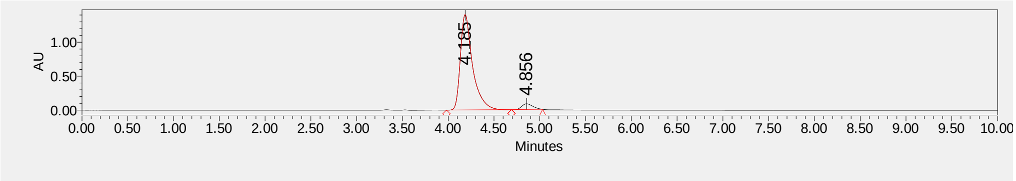

	Retention Time	Area	% Area
1	4.185	12992239	94.90
2	4.856	697892	5.10

Dmethyl (2*S*,10b*S*,*E*)-3-(tert-butylimino)-2-(4-chlorophenyl)-2,3-dihydropyrrolo[2,1-a] isoquinoline-1,1(10bH)-dicarboxylate (8d)

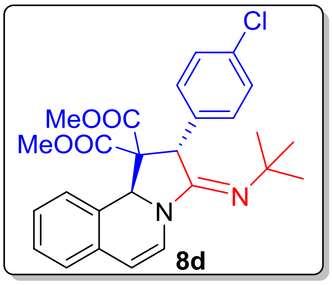

Pale yellow oil; 90% yield, 95:5 e.r.; $[\alpha]^{25}_D$ = +154.03 (c = 0.37 in CH_2Cl_2).

HPLC DAICEL CHIRALCEL IA, *n*-hexane/2-propanol = 90/10, flow rate = 1.0 mL/min, λ = 350 nm, t_R (major) = 4.16 min, t_R (minor) = 4.90 min.

IR (neat): 2965, 2864, 1738, 1672, 1630, 1489, 1449, 1406, 1354, 1306, 1269, 1211, 1084, 947, 773 and 731 cm^{-1}.

^{1}H NMR (400 MHz, $CDCl_3$) δ = 7.32 – 7.27 (m, 2H), 7.22 – 7.17 (m, 2H), 7.14 – 7.08 (m, 3H), 7.02 – 6.96 (m, 1H), 6.94 – 6.90 (m, 1H), 5.90 (s, 1H), 5.64 (d, J = 7.8 Hz, 1H), 4.65 (s, 1H), 3.57 (s, 3H), 3.54 (s, 3H), 1.08 (s, 9H).

^{13}C{^{1}H} NMR (101 MHz, $CDCl_3$) δ = 169.1, 168.1, 150.7, 134.4, 133.7, 133.5, 130.6, 129.1, 128.2, 127.0, 126.8, 125.7, 124.7, 124.6, 105.9, 66.9, 58.9, 53.7, 52.8, 52.6, 52.4, 31.8.

HRMS (ESI-FT) calcd for $C_{26}H_{28}{}^{35}ClN_2O_4^+$ ([M+H$^+$]) = 467.1732, Found 467.1729, $C_{26}H_{28}{}^{37}ClN_2O_4^+$ ([M+H$^+$]) = 469.1703, Found 469.1724.

Chiral HPLC spectrum **8d**:

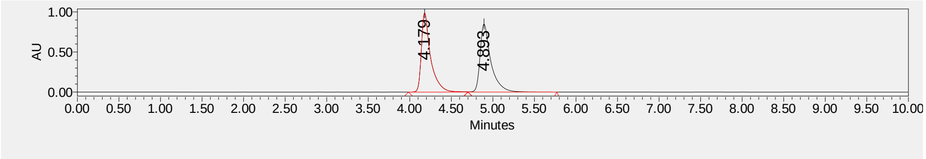

	Retention Time	Area	% Area
1	4.179	7631806	49.84
2	4.893	7679352	50.16

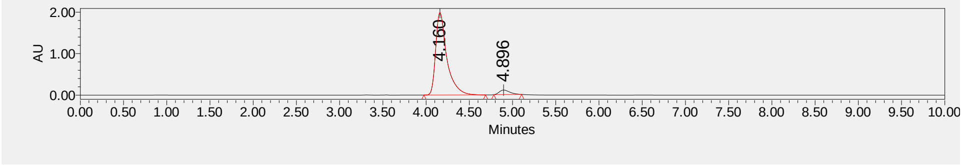

	Retention Time	Area	% Area
1	4.160	17832631	95.08

2	4.896	923498	4.92

Dimethyl (2*S*,10b*S*,*E*)-3-(tert-butylimino)-2-(3-chlorophenyl)-2,3-dihydropyrrolo[2,1-a] isoquinoline-1,1(10bH)-dicarboxylate (8e)

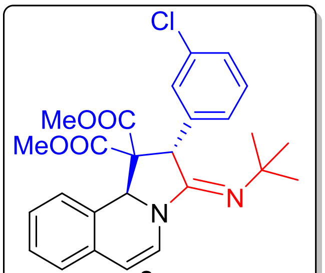

8e

Pale yellow oil; 88% yield, 92:8 e.r.; $[\alpha]^{25}_{D}$ = +159.44 (*c* = 0.60 in CH_2Cl_2).

HPLC DAICEL CHIRALCEL IA, *n*-hexane/2-propanol = 90/10, flow rate = 1.0 mL/min, λ = 350 nm, t_R (major) = 3.87 min, t_R (minor) = 4.94 min.

IR (neat): 2965, 1740, 1672, 1630, 1406, 1354, 1306, 1271, 1209, 1072, 951, 891, 775 and 706 cm^{-1}.

^{1}H NMR (400 MHz, $CDCl_3$) δ = 7.28 – 7.25 (m, 2H), 7.24 – 7.16 (m, 3H), 7.13 – 7.04 (m, 2H), 7.02 – 7.96 (m, 1H), 6.95 – 6.90 (m, 1H), 5.91 (s, 1H), 5.64 (d, *J* = 7.8Hz, 1H), 4.64 (s, 1H), 3.60 (s, 3H), 3.54 (s, 3H), 1.09 (s, 9H).

^{13}C{^{1}H} NMR (101 MHz, $CDCl_3$) δ = 169.0, 168.1, 150.5, 137.2, 134.7, 133.5, 130.2, 128.6, 128.2, 126.9, 126.8, 125.7, 124.7, 124.6, 105.9, 58.9, 53.8, 52.8, 52.6, 31.8.

HRMS (ESI-FT) calcd for $C_{26}H_{28}{}^{35}ClN_2O_4^+$ ([M+H$^+$]) = 467.1732, Found 467.1738, $C_{26}H_{28}{}^{37}ClN_2O_4^+$ ([M+H$^+$]) = 469.1703, Found 469.1712.

Chiral HPLC spectrum **8e**:

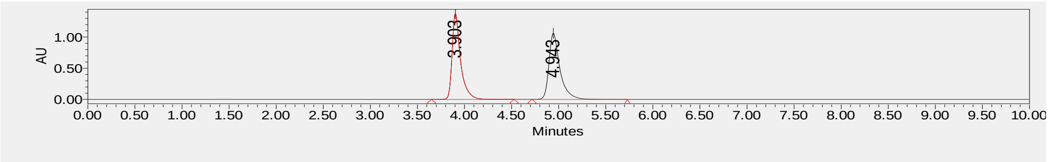

	Retention Time	Area	% Area
1	3.903	8636808	49.96
2	4.943	8650836	50.04

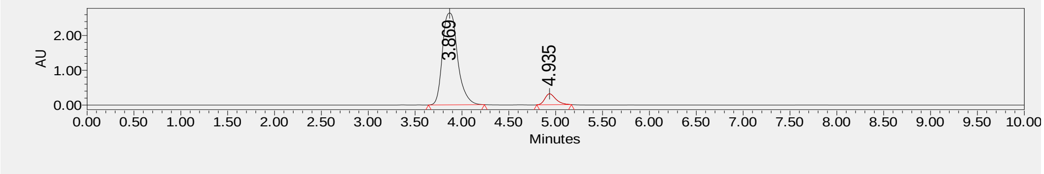

	Retention Time	Area	% Area
1	3.869	29714217	92.03
2	4.935	2574573	7.97

Dimethyl (2*S*,10b*S*,*E*)-3-(tert-butylimino)-2-(p-tolyl)-2,3-dihydropyrrolo[2,1-a]isoquinoline-1,1(10bH)-dicarboxylate (8f)

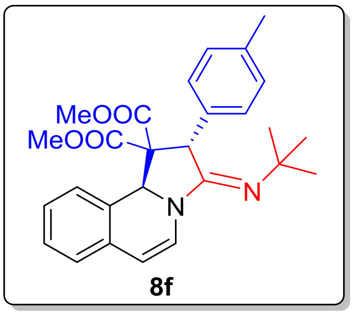

Pale yellow oil; 83% yield, 95.5:4.5 e.r.; $[\alpha]^{25}_{D}$ = +183.11 (*c* = 0.60 in CH_2Cl_2). **HPLC** DAICEL CHIRALCEL IA, *n*-hexane/2-propanol = 90/10, flow rate = 1.0 mL/min, λ = 350 nm, t_R (major) = 4.21 min, t_R (minor) = 4.89 min.

IR (neat): 2963, 1738, 1672, 1627, 1449, 1406, 1352, 1304, 1265, 1207, 1072, 943, 773 and 727 cm^{-1}.

^{1}**H NMR** (400 MHz, $CDCl_3$) δ = 7.25 – 7.18 (m, 2H), 7.12 – 7.07 (m, 3H), 7.06 – 7.02 (m, 2H), 7.00 – 7.96 (m, 1H), 6.94 – 7.89 (m, 1H), 5.94 (s, 1H), 5.63 (d, *J* = 7.8 Hz, 1H), 4.64 (s, 1H), 3.54 (d, *J* = 7.6 Hz, 6H), 2.31 (s, 3H), 1.08 (s, 9H).

^{13}**C{^{1}H} NMR** (101 MHz, $CDCl_3$) δ = 169.4, 168.3, 151.5, 138.1, 133.6, 131.8, 129.5, 128.0, 127.3, 126.8, 125.6, 124.8, 124.6, 105.6, 67.1, 59.0, 53.6, 52.8, 52.7, 52.4, 31.7, 21.3.

HRMS (ESI-FT) calcd for $C_{27}H_{31}N_2O_4^+$ ($[M+H^+]$) = 447.2278, Found 47.2282.

Chiral HPLC spectrum **8f**:

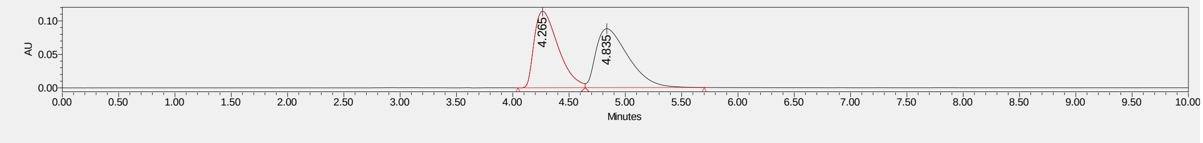

	Retention Time	Area	% Area
1	4.265	1698273	49.16
2	4.835	1756495	50.84

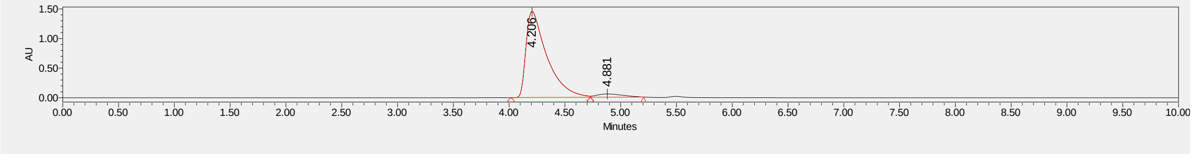

	Retention Time	Area	% Area
1	4.206	18951347	95.53
2	4.881	886515	4.47

Dimethyl (2*S*,10b*S*,*E*)-3-(tert-butylimino)-2-(m-tolyl)-2,3-dihydropyrrolo[2,1-a]isoquinoline-1,1(10bH)-dicarboxylate (8g)

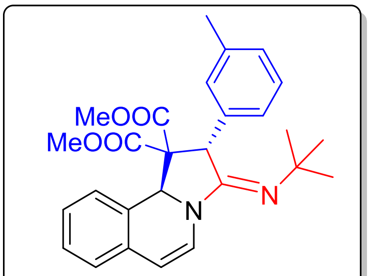

8g

Pale yellow oil; 91% yield, 95:5 e.r.; $[\alpha]^{25}_{D}$ = +175.36 (*c* = 0.82 in CH_2Cl_2).

HPLC DAICEL CHIRALCEL IA, *n*-hexane/2-propanol = 90/10, flow rate = 1.0 mL/min, λ = 350 nm, t_R (major) = 3.77 min, t_R (minor) = 4.21 min.

IR (neat): 2963, 1740, 1672, 1630, 1450, 1408, 1352, 1306, 1256, 1207, 1072, 957, 775 and 729 cm^{-1}.

^{1}H NMR (400 MHz, $CDCl_3$) δ = 7.26 – 7.23 (m, 1H), 7.21 – 7.16 (m, 2H), 7.12 – 7.06 (m, 2H), 7.01 – 6.89 (m, 4H), 5.96 (s, 1H), 5.63 (d, *J* = 7.8 Hz, 1H), 4.65 (s, 1H), 3.54 (d, *J* = 2.4 Hz, 6H), 2.32 (s, 3H), 1.09 (s, 9H).

^{13}C{^{1}H} NMR (101 MHz, $CDCl_3$) δ = 169.3, 168.3, 151.5, 138.4, 134.8, 133.6, 129.1, 128.6, 128.0, 127.2, 126.9, 125.6, 124.8, 124.6, 105.5, 67.1, 59.0, 53.6, 53.1, 52.7, 52.4, 31.7, 21.6.

HRMS (ESI-FT) calcd for $C_{27}H_{31}N_2O_4^+$ ([M+H$^+$]) = 447.2278, Found 447.2282.

Chiral HPLC spectrum **8g**:

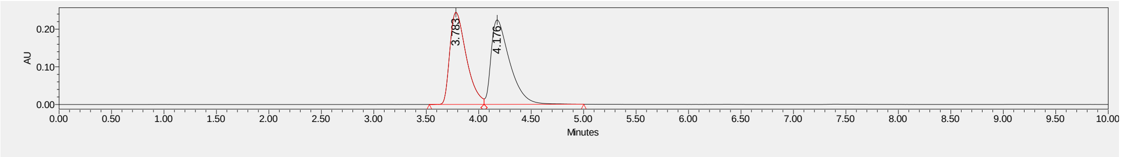

	Retention Time	Area	% Area
1	3.783	2661242	48.98
2	4.176	2772307	51.02

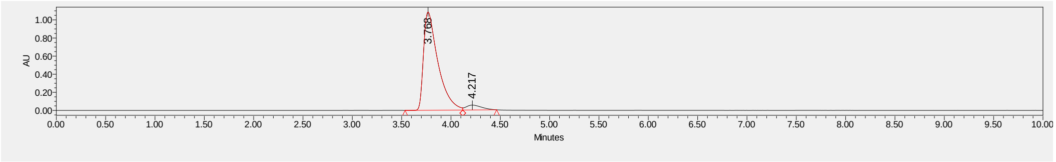

	Retention Time	Area	% Area
1	3.768	10993248	94.93
2	4.217	586895	5.07

Dimethyl (2*S*,10b*S*,*E*)-3-(tert-butylimino)-2-(naphthalen-2-yl)-2,3-dihydropyrrolo[2,1-a] isoquinoline-1,1(10bH)-dicarboxylate (8h)

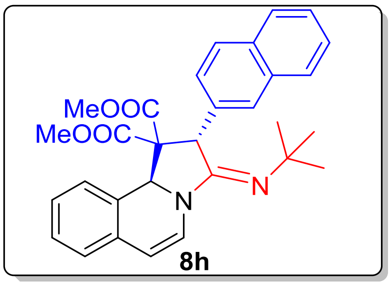

White solid; m.p. 76-78 °C; 93% yield, 97:3 e.r.; $[\alpha]^{25}_{D}$ = +88.95 (*c* = 0.38 in CH_2Cl_2).

HPLC DAICEL CHIRALCEL ODH, *n*-hexane/2-propanol = 90/10, flow rate = 1.0 mL/min, λ = 350 nm, t_R (major) = 4.18 min, t_R (minor) = 5.23 min.

IR (neat): 2965, 1738, 1672, 1630, 1449, 1406, 1354, 1306, 1263, 1209, 1072, 901, 775 and 743 cm^{-1}.

^{1}H NMR (400 MHz, $CDCl_3$) δ = 7.85 – 7.78 (m, 3H), 7.68 – 7.63 (m, 1H), 7.52 – 7.46 (m, 2H), 7.34 – 7.27 (m, 2H), 7.23 – 7.18 (m, 1H), 7.14 – 7.09 (m, 1H), 7.02 – 6.92 (m, 2H), 6.08 (s, 1H), 5.69 (d, *J* = 7.8 Hz, 1H), 4.87 (s, 1H), 3.57 (s, 3H), 3.49 (s, 3H), 1.09 (s, 9H).

^{13}C{^{1}H} NMR (101 MHz, $CDCl_3$) δ = 169.3, 168.2, 151.3, 133.6, 133.4, 133.0, 132.5, 128.6, 128.1, 127.8, 127.2, 126.9, 126.6, 125.6, 124.8, 124.6, 105.7, 67.3, 59.1, 53.7, 53.2, 52.8, 52.5, 31.8.

HRMS (ESI-FT) calcd for $C_{30}H_{31}N_2O_4^+$ ($[M+H^+]$) = 483.2278, Found 483.2277.

Chiral HPLC spectrum **8h**:

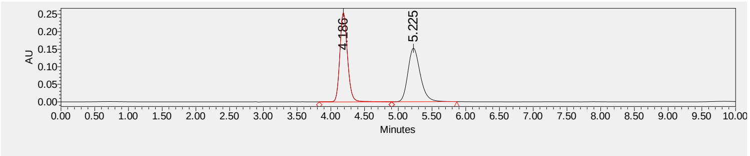

	Retention Time	Area	% Area
1	4.186	1987417	50.65
2	5.225	1936199	49.35

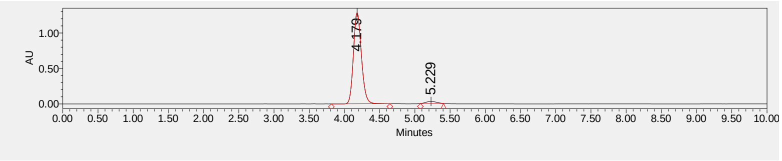

	Retention Time	Area	% Area
1	4.179	10091286	97.08
2	5.229	303935	2.92

Dimethyl (2*S*,10b*S*,*E*)-3-(tert-butylimino)-7-nitro-2-phenyl-2,3-dihydropyrrolo[2,1-a] isoquinoline-1,1(10bH)-dicarboxylate (8i)

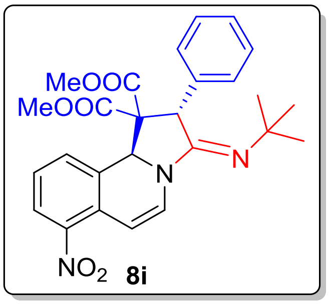

Orange red oil; 53% yield, 97:3 e.r.; $[\alpha]^{25}_{D}$ = +86.19 (*c* = 0.42 in CH_2Cl_2).

HPLC DAICEL CHIRALCEL IA, *n*-hexane/2-propanol = 90/10, flow rate = 1.0 mL/min, λ = 350 nm, t_R (major) = 4.63 min, t_R (minor) = 6.64 min.

IR (neat): 2963, 2926, 2858, 1740, 1682, 1612, 1524, 1462, 1402, 1350, 1269, 1213, 1072 and 763 cm^{-1}.

^{1}H NMR (400 MHz, $CDCl_3$) δ = 7.87 – 7.81 (m, 1H), 7.60 – 7.52 (m, 2H), 7.48 – 7.41 (m, 3H), 7.28 – 7.23 (m, 2H), 7.16 (t, *J* = 8.0 Hz, 1H), 6.44 (d, *J* = 8.0, 1H), 6.07 (s, 1H), 4.82 (s, 1H), 3.73 (s, 3H), 3.68 (s, 3H), 1.20 (s, 9H).

^{13}C{^{1}H} NMR (101 MHz, $CDCl_3$) δ = 168.7, 168.0, 149.8, 144.6, 134.5, 131.3, 129.5, 129.3, 129.1, 129.0, 128.7, 124.9, 124.6, 98.5, 67.0, 58.6, 54.1, 53.1, 53.0, 52.7, 31.5.

HRMS (ESI-FT) calcd for $C_{26}H_{28}N_3O_6^+$ ($[M+H^+]$) = 478.1973, Found 478.1964.

Chiral HPLC spectrum **8i**:

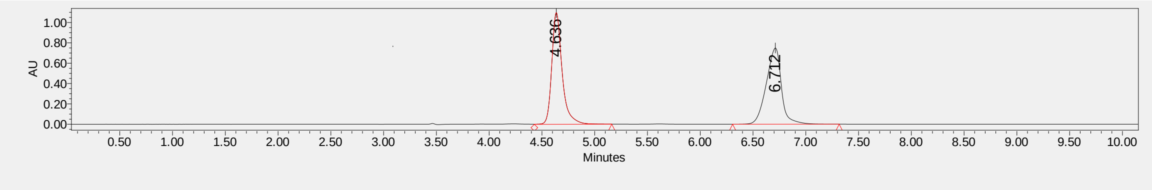

	Retention Time	Area	% Area
1	4.636	7473300	50.08
2	6.712	7448875	49.92

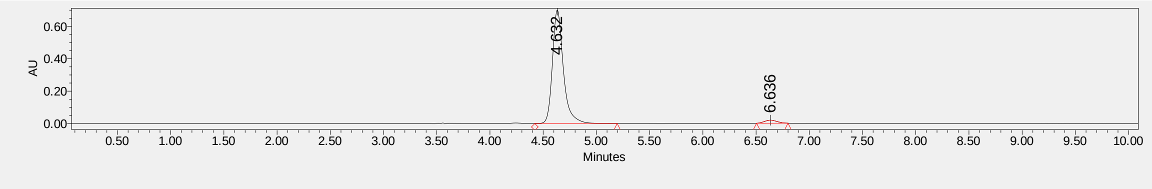

	Retention Time	Area	% Area
1	4.632	5016304	97.04
2	6.636	153249	2.96

Dimethyl (2*S*,10b*S*,*E*)-3-(tert-butylimino)-2-(4-methoxyphenyl)-7-nitro-2,3-dihydropyrrolo [2,1-a]isoquinoline-1,1(10bH)-dicarboxylate (8j)

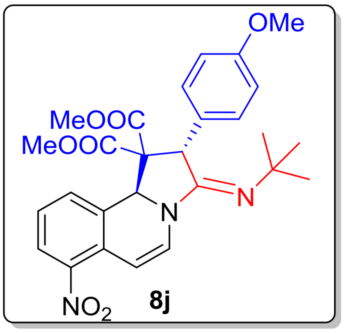

Orange red oil; 40% yield, 98:2 e.r.; $[\alpha]^{25}_{D}$ = +32.24 (*c* = 0.37 in CH_2Cl_2).

HPLC DAICEL CHIRALCEL IA, *n*-hexane/2-propanol = 90/10, flow rate = 1.0 mL/min, λ = 350 nm, t_R (major) = 5.26 min, t_R (minor) = 10.72 min.

IR (neat): 2965, 1738, 1682, 1612, 1518, 1463, 1350, 1263, 1070, and 770 cm^{-1}.

^{1}H NMR (400 MHz, $CDCl_3$) δ = 7.75 – 7.68 (m, 1H), 7.47 – 7.38 (m, 2H), 7.07 – 6.98 (m, 3H), 6.87 – 6.80 (m, 2H), 6.30 (d, *J* = 8.0 Hz, 1H), 5.91 (s, 1H), 4.64 (s, 1H), 3.79 (s, 3H), 3.59 (d, *J* = 1.2 Hz, 6H), 1.08 (s, 9H).

^{13}C{^{1}H} NMR (101 MHz, $CDCl_3$) δ = 168.8, 168.2, 159.7, 150.0, 144.6, 131.3, 130.2, 129.6, 129.3, 129.2, 126.1, 124.9, 124.6, 114.4, 98.5, 67.1, 58.6, 55.3, 54.0, 53.1, 52.7, 52.3, 31.5.

HRMS (ESI-FT) calcd for $C_{27}H_{30}N_3O_7^+$ ([M+H$^+$]) = 508.2078, Found 508.2081.

Chiral HPLC spectrum **8j**:

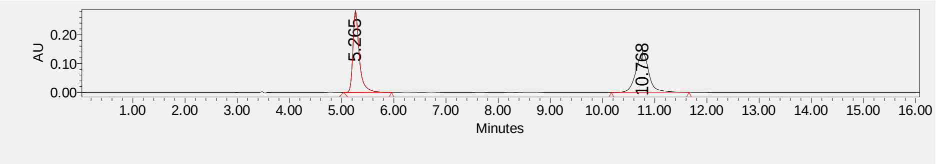

	Retention Time	Area	% Area
1	5.265	2405177	50.14
2	10.768	2391489	49.86

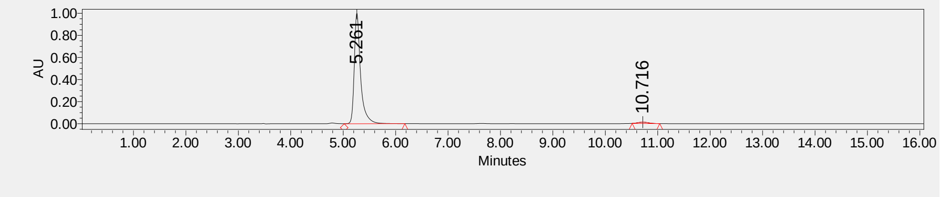

	Retention Time	Area	% Area
1	5.261	8365420	98.00
2	10.716	170581	2.00

Dimethyl (2*S*,10b*S*,*E*)-3-(tert-butylimino)-2-(4-cyanophenyl)-7-nitro-2,3-dihydropyrrolo [2,1-a]isoquinoline-1,1(10bH)-dicarboxylate (8k)

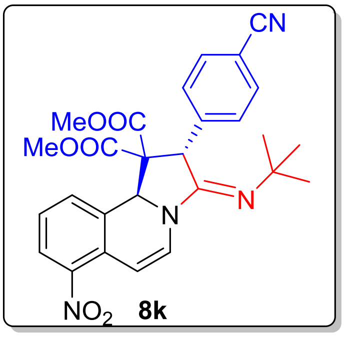

Orange red oil; 60% yield, 96:4 e.r.; $[\alpha]^{25}_D$ = +13.54 (c = 0.58 in CH_2Cl_2).

HPLC DAICEL CHIRALCEL IA, *n*-hexane/2-propanol = 70/30, flow rate = 1.0 mL/min, λ = 350 nm, t_R (major) = 4.35 min, t_R (minor) = 10.10 min.

IR (neat): 2967, 2232, 1740, 1682, 1612, 1522, 1462, 1404, 1350, 1271, 1213, 1070, 768 and 733 cm^{-1}.

^{1}H NMR (400 MHz, $CDCl_3$) δ = 7.75 – 7.70 (m, 1H), 7.67 – 7.61 (m, 2H), 7.44 – 7.37 (m, 2H), 7.29 (d, J = 8.2 Hz, 2H), 7.06 (t, J = 8.0 Hz, 1H), 6.32 (d, J = 8.0 Hz, 1H), 5.88 (s, 1H), 4.74 (s, 1H), 3.60 (d, J = 5.8 Hz, 6H), 1.06 (s, 9H).

^{13}C{^{1}H} NMR (101 MHz, $CDCl_3$) δ = 168.2, 167.6, 148.4, 144.8, 140.3, 132.7, 131.3,

129.9, 129.1, 128.9, 128.8, 125.2, 124.8, 118.1, 112.8, 99.1, 66.8, 58.5, 58.5, 54.2, 53.3, 53.0, 52.6, 31.5.

HRMS (ESI-FT) calcd for $C_{27}H_{27}N_4O_6^+$ ($[M+H^+]$) = 503.1925, Found 503.1921.

Chiral HPLC spectrum **8**:

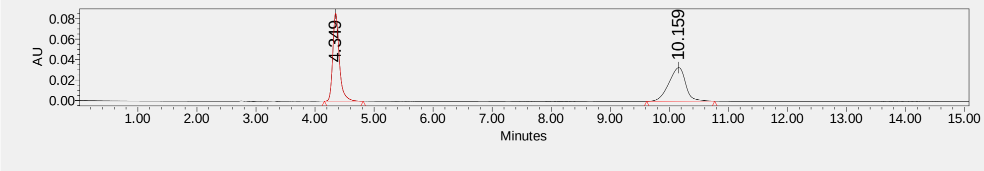

	Retention Time	Area	% Area
1	4.349	658122	50.49
2	10.159	645383	49.51

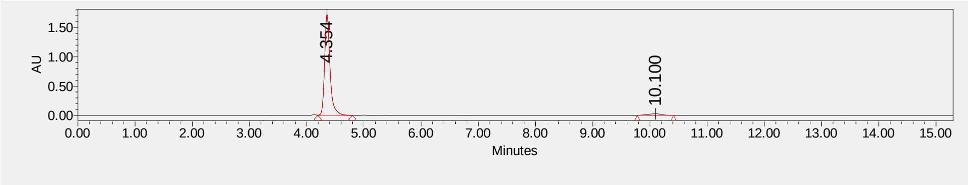

	Retention Time	Area	% Area
1	4.354	11361458	96.23
2	10.100	444742	3.77

Dimethyl (2*S*,10b*S*,*E*)-2-(4-bromophenyl)-3-(tert-butylimino)-7-nitro-2,3-dihydropyrrolo [2,1-a]isoquinoline-1,1(10bH)-dicarboxylate (8l)

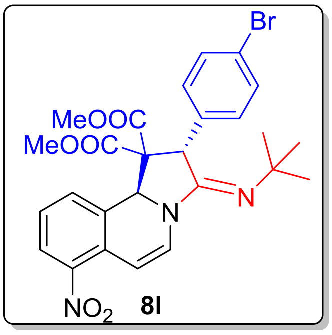

Orange red oil; 72% yield, 97.5:2.5 e.r.; $[\alpha]^{25}_{D}$ = +17.01 (*c* = 0.78 in CH_2Cl_2).

HPLC DAICEL CHIRALCEL IA, *n*-hexane/2-propanol = 90/10, flow rate = 1.0 mL/min, λ = 350 nm, t_R (major) = 4.91 min, t_R (minor) = 7.66 min.

IR (neat): 2965, 1738, 1682, 1612, 1523, 1350, 1273, 1213, 1072 and 766 cm^{-1}.

^{1}H NMR (400 MHz, $CDCl_3$) δ = 7.74 – 7.70 (m, 1H), 7.49 – 7.38 (m, 4H), 7.08 – 6.98 (m, 3H), 6.31 (d, *J* = 8.4, 1H), 5.88 (s, 1H), 4.65 (s, 1H), 3.60 (d, *J* = 2.0 Hz, 6H),

1.07 (s, 9H).

^{13}C{^{1}H} NMR (101 MHz, $CDCl_3$) δ = 168.5, 167.9, 149.1, 144.7, 133.7, 132.3, 131.3, 130.7, 129.3, 129.1, 129.0, 125.0, 124.7, 122.9, 98.8, 66.8, 58.5, 54.1, 53.2, 52.9, 52.3, 31.5.

HRMS (ESI-FT) calcd for $C_{26}H_{27}{}^{79}BrN_3O_6^+$ ([M+H$^+$]) = 556.1078, Found 556.1083, $C_{26}H_{27}{}^{81}BrN_3O_6^+$ ([M+H$^+$]) = 558.1057, Found 558.1062.

Chiral HPLC spectrum **8l**:

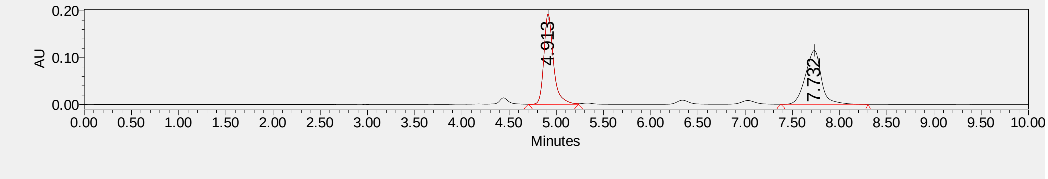

	Retention Time	Area	% Area
1	4.913	1363259	50.26
2	7.732	1348935	49.74

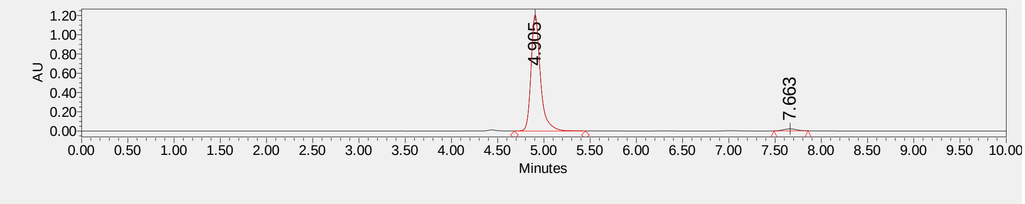

	Retention Time	Area	% Area
1	4.905	8482327	97.58
2	7.663	210429	2.42

Dimethyl (2*S*,10b*S*,*E*)-3-(tert-butylimino)-2-(4-chlorophenyl)-7-nitro-2,3-dihydropyrrolo [2,1-a]isoquinoline-1,1(10bH)-dicarboxylate (8m)

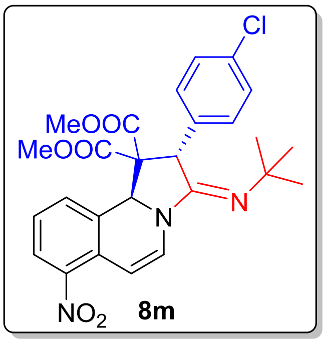

Orange red oil; 65% yield, 98:2 e.r.; $[\alpha]^{25}_{D}$ = +29.03 (*c* = 0.62 in CH_2Cl_2).

HPLC DAICEL CHIRALCEL IA, *n*-hexane/2-propanol = 90/10, flow rate = 1.0 mL/min, λ = 350 nm, t_R (major) = 4.79 min, t_R (minor) = 7.77 min.

IR (neat): 2965, 1740, 1680, 1612, 1524, 1462, 1350, 1273, 1093, 766 and 733 cm^{-1}.

^{1}H NMR (400 MHz, $CDCl_3$) δ = 7.75 – 7.68 (m, 1H), 7.45 – 7.38 (m, 2H), 7.33 – 7.29 (m, 2H), 7.12 – 7.00 (m, 3H), 6.31 (d, *J* = 8.0 Hz, 1H), 5.89 (s, 1H), 4.67 (s, 1H), 3.59 (s, 3H), 1.07 (s, 9H).

^{13}C{^{1}H} NMR (101 MHz, $CDCl_3$) δ = 168.5, 167.9, 149.2, 144.7, 134.7, 133.1, 131.3, 130.4, 129.3, 129.1, 129.0, 125.0, 124.7, 98.8, 66.9, 58.5, 54.1, 53.2, 52.9, 52.2, 31.5.

HRMS (ESI-FT) calcd for $C_{26}H_{27}{}^{35}ClN_3O_6^+$ ([M+H$^+$]) = 512.1583, Found 512.1285, $C_{26}H_{27}{}^{37}ClN_3O_6^+$ ([M+H$^+$]) = 514.1553, Found 514.1563.

Chiral HPLC spectrum **8m**:

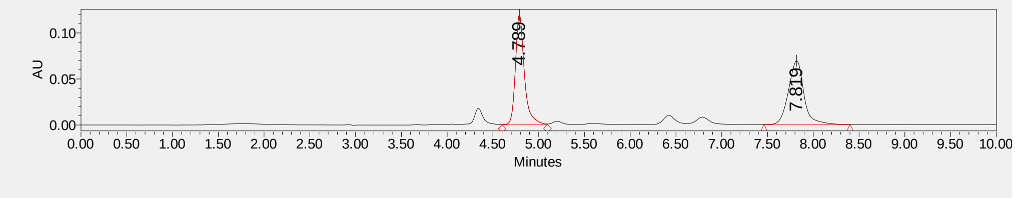

	Retention Time	Area	% Area
1	4.789	817491	50.17
2	7.819	811950	49.83

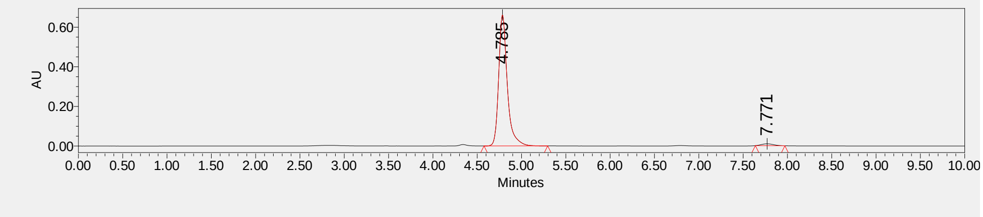

	Retention Time	Area	% Area
1	4.785	4478089	98.05
2	7.771	89286	1.95

Dimethyl (2*S*,10b*S*,*E*)-3-(tert-butylimino)-2-(3-chlorophenyl)-7-nitro-2,3-dihydropyrrolo [2,1-a]isoquinoline-1,1(10bH)-dicarboxylate (8n)

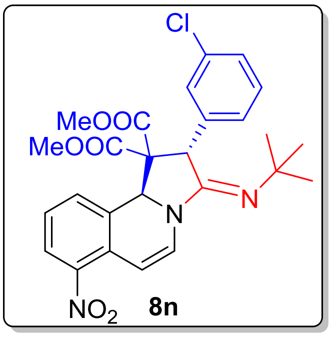

Orange red solid; m.p. 78-82 °C; 63% yield, 97:3 e.r.; $[\alpha]^{25}_{D}$ = +89.41 (c = 0.34 in CH_2Cl_2).

HPLC DAICEL CHIRALCEL IA, *n*-hexane/2-propanol = 90/10, flow rate = 1.0 mL/min, λ = 350 nm, t_R (major) = 4.53 min, t_R (minor) = 7.56 min.

IR (neat): 2965, 1740, 1680, 1612, 1526, 1466, 1402, 1350, 1273, 1213, 770 and 735 cm^{-1}.

^{1}H NMR (400 MHz, $CDCl_3$) δ = 7.75 – 7.68 (m, 1H), 7.46 – 7.39 (m, 2H), 7.33 – 7.27 (m, 2H), 7.16 – 7.13 (m, 1H), 7.08 – 6.99 (m, 2H), 6.31 (d, *J* = 8.0 Hz, 1H), 5.89 (s, 1H), 4.67 (s, 1H), 3.63 (s, 3H), 3.60 (s, 3H), 1.09 (s, 9H).

^{13}C{^{1}H} NMR (101 MHz, $CDCl_3$) δ = 168.4, 167.8, 149.0, 144.7, 136.6, 134.9, 131.4, 130.4, 129.3, 129.1, 129.0, 128.9, 125.0, 124.7, 98.7, 66.9, 58.6, 54.2, 53.2, 52.9, 52.4, 31.5.

HRMS (ESI-FT) calcd for $C_{26}H_{27}{}^{35}ClN_3O_6^+$ ([M+H$^+$]) = 512.1583, Found 512.1589, $C_{26}H_{27}{}^{37}ClN_3O_6^+$ ([M+H$^+$]) = 514.1553, Found 514.1561.

Chiral HPLC spectrum **8n**:

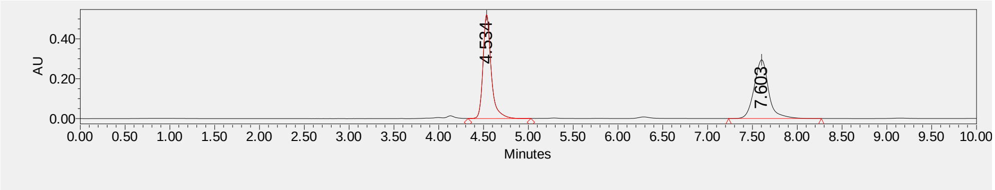

	Retention Time	Area	% Area
1	4.534	3329168	50.24
2	7.603	3296936	49.76

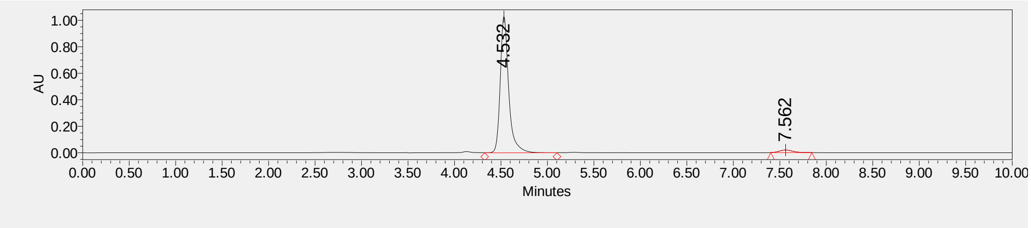

	Retention Time	Area	% Area
1	4.532	6515916	97.09
2	7.562	195584	2.91

Dimethyl (2*S*,10b*S*,*E*)-3-(tert-butylimino)-7-methoxy-2-phenyl-2,3-dihydropyrrolo [2,1-a]isoquinoline-1,1(10bH)-dicarboxylate (8o)

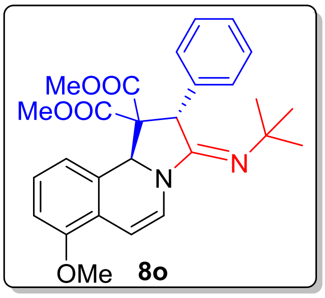

White solid; m.p. 64-68 °C; 38% yield, 97.5:2.5 e.r.; $[\alpha]^{25}_{D}$ = +188.33 (c = 0.24 in CH_2Cl_2).

HPLC DAICEL CHIRALCEL IA, *n*-hexane/2-propanol = 90/10, flow rate = 1.0 mL/min, λ = 350 nm, t_R (major) = 4.95 min, t_R (minor) = 8.13 min.

IR (neat): 2963, 1738, 1672, 1570, 1468, 1308, 1261, 1211, 1069 and 754 cm^{-1}.

^{1}H NMR (400 MHz, $CDCl_3$) δ = 7.33 – 7.26 (m, 3H), 7.23 – 7.13 (m, 3H), 6.95 (t, J = 8.0 Hz, 1H), 6.83 – 7.77 (m, 1H), 6.72 – 6.45 (m, 1H), 6.05 (d, J = 8.0 Hz, 1H), 5.93 (s, 1H), 4.67 (s, 1H), 3.79 (s, 3H), 3.55 (s, 3H), 3.51 (s, 3H), 1.07 (s, 9H).

^{13}C{^{1}H} NMR (101 MHz, $CDCl_3$) δ = 169.3, 168.3, 153.5, 135.1, 128.8, 128.4, 128.3, 126.1, 124.0, 123.0, 119.2, 110.1, 99.5, 67.1, 59.0, 55.8, 53.7, 53.3, 52.8, 52.4, 31.7.

HRMS (ESI-FT) calcd for $C_{27}H_{31}N_2O_5^+$ ([M+H$^+$]) = 463.2227, Found 463.2227.

Chiral HPLC spectrum **8o**:

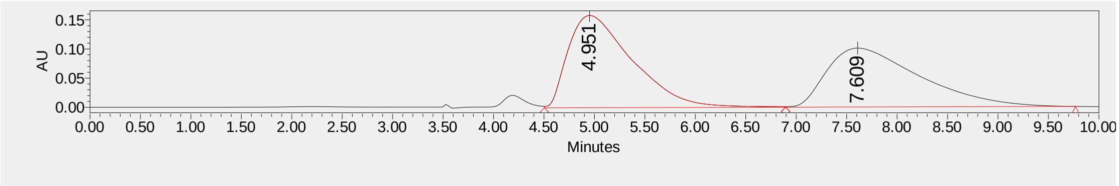

	Retention Time	Area	% Area
1	4.951	7017921	51.98
2	7.609	6483581	48.02

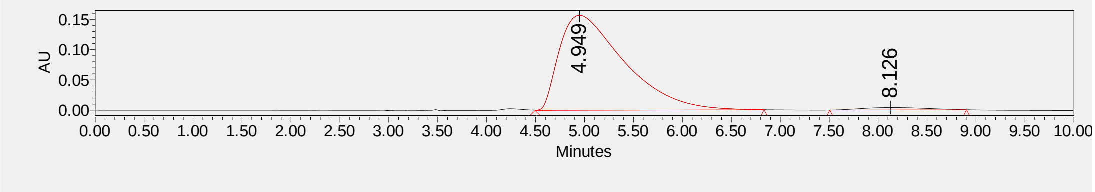

	Retention Time	Area	% Area
1	4.949	7239923	97.59
2	8.126	179097	2.41

Dimethyl (2*S*,10b*S*,*E*)-3-(tert-butylimino)-8-cyano-2-phenyl-2,3-dihydropyrrolo [2,1-a]isoquinoline-1,1(10bH)-dicarboxylate (8p)

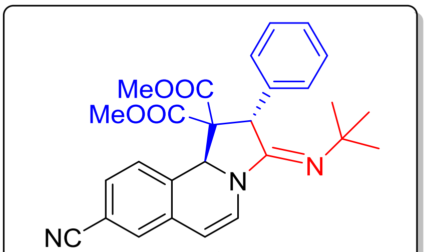

8p

White solid; m.p. 72-76 °C; 59% yield, 99:1 e.r.; $[\alpha]^{25}_{D}$ = +269.15 (c = 0.47 in CH_2Cl_2).

HPLC DAICEL CHIRALCEL IC, *n*-hexane/2-propanol = 90/10, flow rate = 1.0 mL/min, λ = 350 nm, t_R (major) = 10.73 min, t_R (minor) = 13.44 min.

IR (neat): 2965, 2232, 1740, 1678, 1624, 1491, 1443, 1273, 1209, 1072, 937, 733 and 706 cm^{-1}.

^{1}H NMR (400 MHz, $CDCl_3$) δ = 7.33 – 7.26 (m, 3H), 7.23 – 7.13 (m, 3H), 6.95 (t, *J* = 8.0 Hz, 1H), 6.83 – 7.77 (m, 1H), 6.72 – 6.45 (m, 1H), 6.05 (d, *J* = 8.0 Hz, 1H), 5.93 (s, 1H), 4.67 (s, 1H), 3.79 (s, 3H), 3.55 (s, 3H), 3.51 (s, 3H), 1.07 (s, 9H).

^{13}C{^{1}H} NMR (101 MHz, $CDCl_3$) δ = 168.8, 168.0, 150.3, 135.2, 134.5, 131.8, 129.0, 128.7, 128.6, 127.8, 127.3, 126.9, 118.9, 112.1, 103.4, 66.9, 58.9, 53.9, 53.0, 52.9, 52.7, 31.6.

HRMS (ESI-FT) calcd for $C_{27}H_{28}N_3O_4^+$ ([M+H$^+$]) = 458.2074, Found 458.2072.

Chiral HPLC spectrum **8p**:

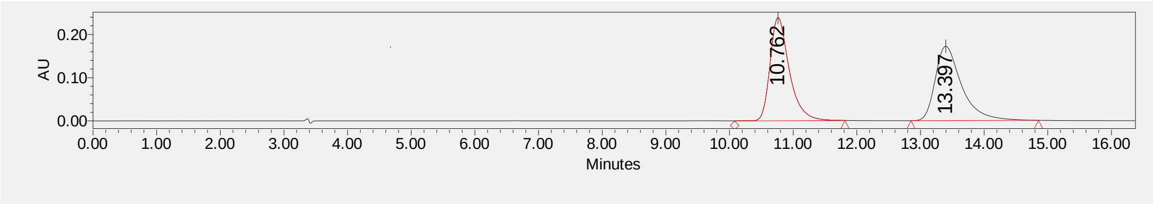

	Retention Time	Area	% Area
1	10.762	5016888	50.35
2	13.397	4947944	49.65

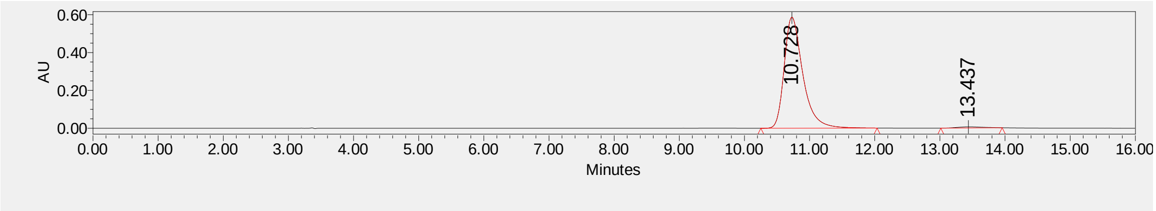

	Retention Time	Area	% Area
1	10.728	11661955	98.68
2	13.437	156288	1.32

Dimethyl (2*S*,10b*S*,*E*)-3-(adamantan-1-ylimino)-7-nitro-2-phenyl-2,3-dihydropyrrolo[2,1-a] isoquinoline-1,1(10bH)-dicarboxylate (8q)

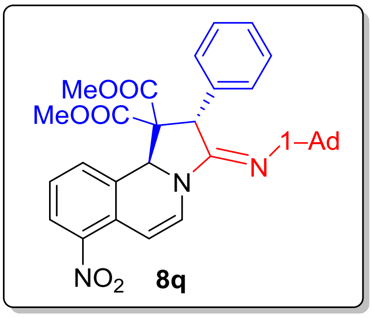

Orange red oil; 73% yield, 98:2 e.r.; $[\alpha]^{25}_{D}$ = +140.97 (*c* = 0.62 in CH_2Cl_2).

HPLC DAICEL CHIRALCEL IA, *n*-hexane/2-propanol = 90/10, flow rate = 1.0 mL/min, λ = 350 nm, t_R (major) = 4.78 min, t_R (minor) = 6.82 min.

IR (neat): 2909, 2853, 1740, 1682, 1612, 1524, 1404, 1346, 1265, 1219, 1090, 766, 735 and 708 cm^{-1}.

^{1}H NMR (400 MHz, $CDCl_3$) δ = 7.76 – 7.67 (m, 1H), 7.47 (d, *J* = 8.0 Hz, 1H), 7.44 – 7.70 (m, 1H), 7.36 – 7.28 (m, 3H), 7.16 – 7.02 (m, 2H), 7.03 (t, *J* = 8.0 Hz, 1H), 6.31 (d, *J* = 8.0 Hz, 1H), 5.92 (s, 1H), 4.71 (s, 1H), 3.60 (s, 3H), 3.56 (s, 3H), 1.98 – 1.90 (m, 3H), 1.68 – 1.63 (m, 3H), 1.59 – 1.47 (m, 9H).

^{13}C{^{1}H} NMR (101 MHz, $CDCl_3$) δ = 168.8, 168.0, 149.2, 144.6, 134.8, 131.3, 129.6, 129.3, 129.2, 129.0, 128.6, 124.8, 124.6, 98.5, 67.0, 58.5, 54.7, 53.5, 53.1, 52.7, 44.2, 36.5, 29.8.

HRMS (ESI-FT) calcd for $C_{32}H_{34}N_3O_6^+$ ([M+H$^+$]) = 556.2442, Found 556.2444.

Chiral HPLC spectrum **8q**:

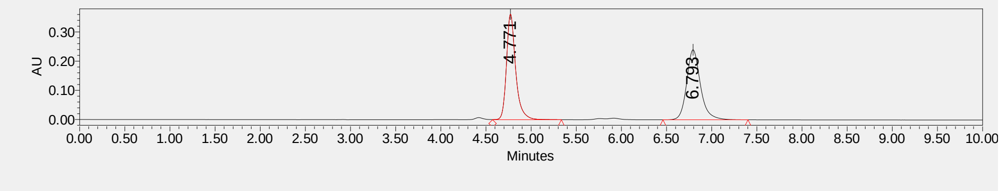

	Retention Time	Area	% Area
1	4.771	2555003	50.08
2	6.793	2547028	49.92

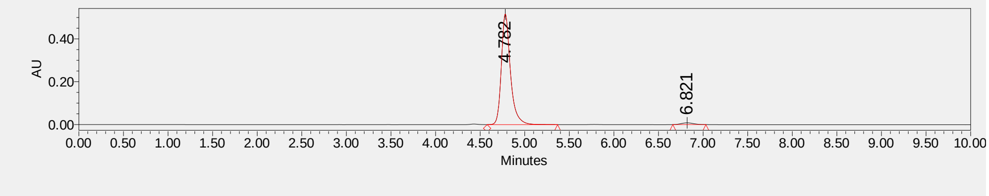

	Retention Time	Area	% Area
1	4.782	3673779	97.91
2	6.821	78396	2.09

Dimethyl (2*S*,10b*S*,*E*)-3-(isopropylimino)-7-nitro-2-phenyl-2,3-dihydropyrrolo[2,1-a] isoquinoline-1,1(10bH)-dicarboxylate (8r)

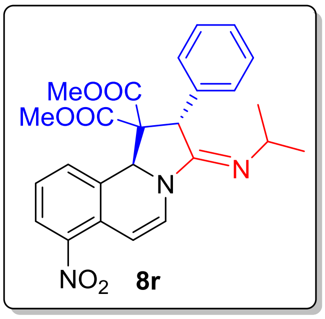

Orange red oil; 75% yield, 89:11 e.r.; $[\alpha]^{25}_{D}$ = +51.62 (*c* = 0.37 in CH_2Cl_2).

HPLC DAICEL CHIRALCEL IA, *n*-hexane/2-propanol = 90/10, flow rate = 1.0 mL/min, λ = 350 nm, t_R (major) = 5.19 min, t_R (minor) = 8.24 min.

IR (neat): 2965, 2874, 1740, 1678, 1614, 1524, 1462, 1406, 1271, 1177, 1074, 768, 735 and 704 cm^{-1}.

^{1}H NMR (400 MHz, $CDCl_3$) δ = 7.75 – 7.70 (m, 1H), 7.45 – 7.37 (m, 2H), 7.35 – 7.29 (m, 3H), 7.19 – 7.14 (m, 2H), 7.07 (t, *J* = 8.0 Hz, 1H), 6.32 (d, *J* = 8.0, 1H), 6.08 (s, 1H), 4.67 (s, 1H), 3.59 (s, 3H), 3.50 (s, 3H), 3.42 (p, *J* = 6.0 Hz, 1H), 1.12 (d, *J* = 6.0 Hz, 3H), 0.66 (d, *J* = 6.0 Hz, 3H).

^{13}C{^{1}H} NMR (101 MHz, $CDCl_3$) δ = 168.7, 168.0, 153.9, 144.8, 134.2, 131.2, 129.5,

129.1, 129.0, 128.8, 128.7, 125.2, 124.7, 99.0, 66.7, 60.5, 53.2, 52.7, 51.6, 51.0, 24.6, 24.0.

HRMS (ESI-FT) calcd for $C_{25}H_{26}N_3O_6^+$ ([M+H$^+$]) = 464.1816, Found 464.1809.

Chiral HPLC spectrum **8r**:

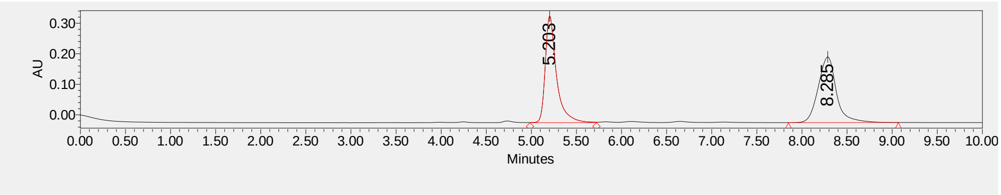

	Retention Time	Area	% Area
1	5.203	3009974	50.08
2	8.285	3000898	49.92

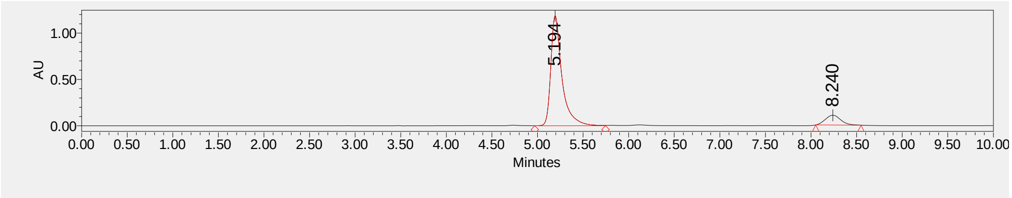

	Retention Time	Area	% Area
1	5.194	10094442	89.10
2	8.240	1235233	10.90

Dimethyl (*E*)-3-((2-methoxy-2-oxoethyl)imino)-7-nitro-2-phenyl-2,3-dihydropyrrolo[2,1-a] isoquinoline-1,1(10bH)-dicarboxylate (8s)

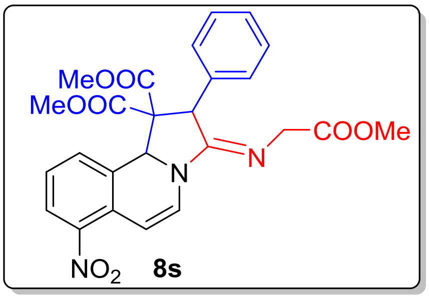

Orange red oil; 81% yield, 50:50 e.r.;

IR (neat): 2955, 2853, 1738, 1676, 1620, 1526, 1462, 1445, 1406, 1350, 1275, 1211, 1184, 1088, 843, 770, 737 and 702 cm^{-1}.

^{1}**H NMR** (400 MHz, $CDCl_3$) δ = 7.78 – 7.70 (m, 1H), 7.42 (d, *J* = 8.0 Hz, 2H), 7.36 – 7.29 (m, 3H), 7.20 – 7.14 (m, 2H), 7.10 (t, *J* = 8.0 Hz, 1H), 6.39 (d, *J* = 8.0 Hz, 1H), 6.15 (s, 1H), 4.63 (s, 1H), 4.04 – 4.00 (m, 1H), 3.85 – 3.78 (m, 1H), 3.60 (s, 6H), 3.47 (s, 3H).

$^{13}C\{^1H\}$ **NMR** (101 MHz, $CDCl_3$) δ = 171.1, 168.4, 167.8, 159.0, 145.1, 132.4, 131.1, 129.6, 129.2, 129.1, 128.0, 125.8, 124.8, 100.5, 66.6, 61.1, 53.4, 52.8, 52.2, 52.1, 52.0.

HRMS (ESI-FT) calcd for $C_{25}H_{24}N_3O_8^+$ ([M+H$^+$]) = 494.1558, Found 494.1562.

Dimethyl (2*S*,10b*S*,*E*)-3-(tert-butylimino)-2-cycloheptyl-7-nitro-2,3-dihydropyrrolo[2,1-a] isoquinoline-1,1(10bH)-dicarboxylate (8t)

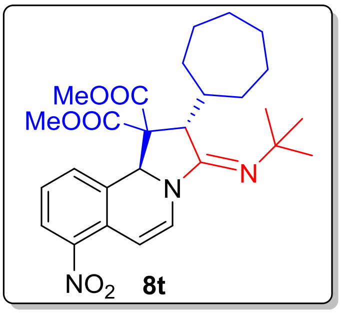

Orange red oil; 21% yield, 77:23 e.r.; $[\alpha]^{21}_D$ = +48.89 (*c* = 0.12 in CH_2Cl_2).

UPC^2 Phenomenex CHIRALCEL IA, CO_2/CH_3CH_2OH = 90/10, λ = 254 nm, flow rate = 1.5 mL/min, t_R (major) = 3.57 min, t_R (minor) = 4.20 min.

IR (neat): 2360, 1733, 1616, 1522, 1458, 1276, 1259, 764 and 750 cm^{-1}.

^{1}**H NMR** (400 MHz, $CDCl_3$) δ = 7.70 (d, *J* = 8.0 Hz, 1H), 7.51 (d, *J* = 8.0 Hz, 1H), 7.29 – 7.22 (m, 2H), 7.04 (t, *J* = 8.0 Hz, 1H), 6.20 (d, *J* = 8.0 Hz, 1H), 5.71 (s, 1H), 3.89 (s, 3H), 3.59 – 3.48 (m, 4H), 1.78 – 1.63 (m, 9H), 1.63 – 1.50 (m, 3H), 1.48 – 1.41 (m, 1H), 1.32 (s, 9H).

$^{13}C\{^1H\}$ **NMR** (101 MHz, $CDCl_3$) δ = 169.8, 169.0, 148.9, 144.4, 131.4, 129.9, 129.6, 129.4, 124.6, 124.5, 97.6, 67.1, 58.9, 54.2, 53.6, 52.9, 40.6, 34.3, 33.1, 31.9, 28.0, 27.5, 27.1.

HRMS (ESI-FT) calcd for $C_{27}H_{36}N_3O_6^+$ ([M+H$^+$]) = 498.2599, Found 498.1600.

Chiral HPLC spectrum **8t**:

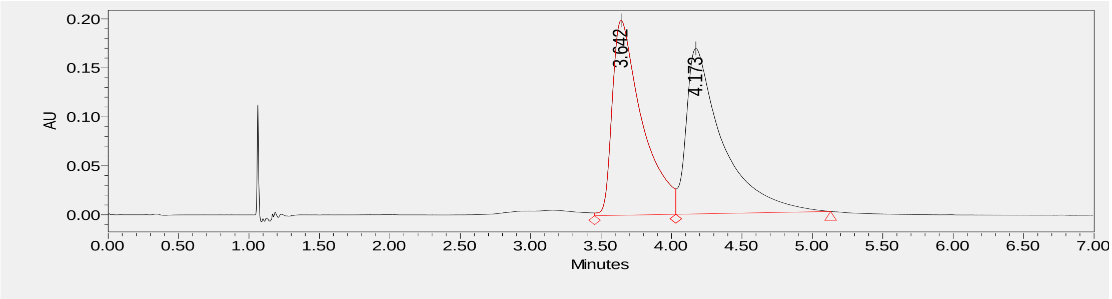

	Retention Time	Area	% Area
1	3.642	2938978	49.07
2	4.173	3050489	50.93

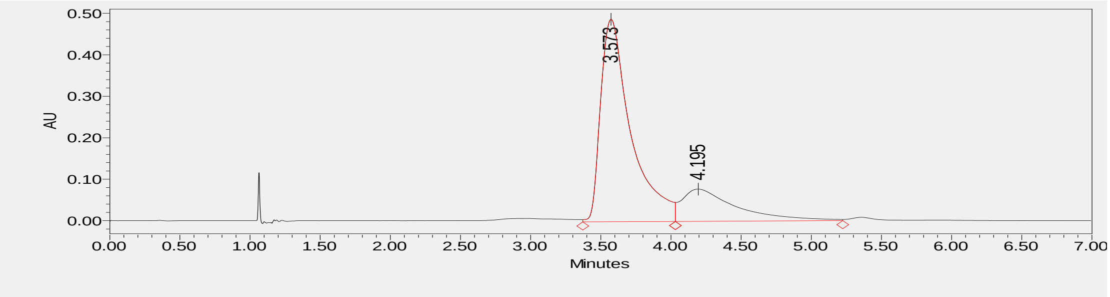

	Retention Time	Area	% Area
1	3.573	7304373	77.69
2	4.195	2097491	22.31

Dimethyl (2*S*,10b*S*,*E*)-3-(naphthalen-2-ylimino)-7-nitro-2-phenyl-2,3-dihydropyrrolo[2,1-a] isoquinoline-1,1(10bH)-dicarboxylate (8u)

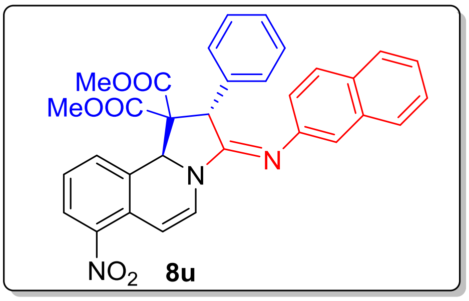

Orange red oil; 68% yield, 73:27 e.r.; $[\alpha]^{21}_D$ = +89.19 (*c* = 0.67 in CH_2Cl_2).

HPLC DAICEL CHIRALCEL IA, *n*-hexane/2-propanol = 90/10, flow rate = 1.0 mL/min, λ = 254 nm, t_R (major) = 23.87 min, t_R (minor) = 37.11 min.

IR (neat): 2361, 1733, 1672, 1623, 1526, 1276, 1259, 764 and 750 cm^{-1}.

^{1}H NMR (400 MHz, $CDCl_3$) δ = 7.82 – 7.77 (m, 1H), 7.74 – 7.68 (m, 1H), 7.58 – 7.43 (m, 4H), 7.39 – 7.28 (m, 3H), 7.24 – 7.13 (m, 3H), 6.93 – 6.85 (m, 2H), 6.79 – 6.76 (m, 1H), 6.72 (dd, *J* = 8.6, 2.1 Hz, 1H), 6.53 (dd, *J* = 8.0, 0.7 Hz, 1H), 6.28 (s, 1H), 4.54 (s, 1H), 3.67 (s, 3H), 3.39 (s, 3H).

^{13}C{^{1}H} NMR (101 MHz, $CDCl_3$) δ = 168.6, 167.9, 157.1, 147.1, 145.2, 134.1, 133.9, 131.2, 130.2, 129.7, 128.9, 128.7, 128.4, 127.7, 127.6, 127.6, 127.2, 126.0, 126.0, 124.9, 124.3, 122.5, 117.4, 101.5, 66.7, 61.2, 53.4, 52.7, 52.4.

HRMS (ESI-FT) calcd for $C_{32}H_{26}N_3O_6^+$ ([M+H$^+$]) = 548.1816, Found 548.1816.

Chiral HPLC spectrum **8u**:

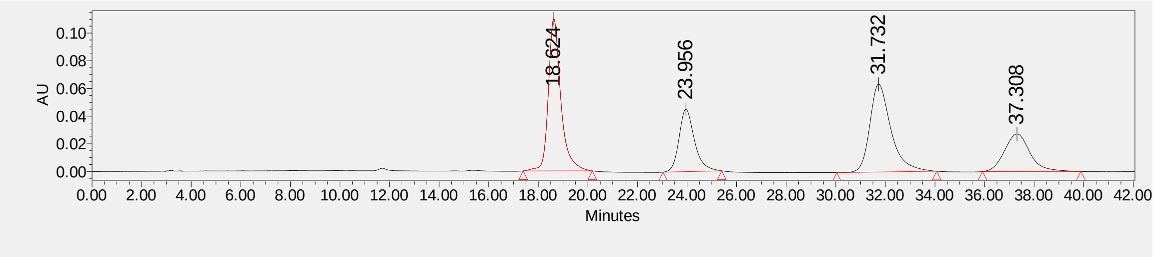

	Retention Time	Area	% Area
1	18.624	3850991	33.51
2	23.956	1950565	16.97
3	31.732	3755358	32.67
4	37.308	1936777	16.85

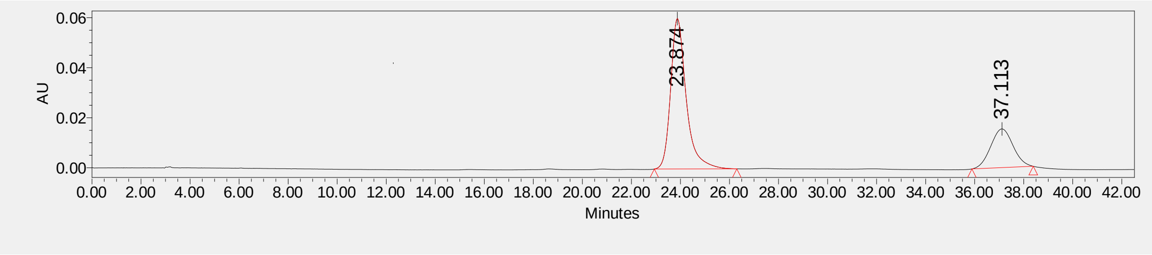

	Retention Time	Area	% Area
1	23.874	2652510	73.03
2	37.113	979436	26.97

Dimethyl (2*S*,10b*S*,*E*)-7-amino-3-(tert-butylimino)-2-phenyl-2,3-dihydropyrrolo[2,1-a] isoquinoline-1,1(10bH)-dicarboxylate (8v)

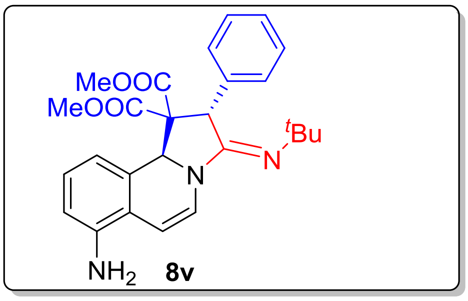

Coreless oil; 32% yield, 98:2 e.r.; $[\alpha]^{21}_{D}$ = +114.13 (*c* = 0.18 in CH_2Cl_2).

HPLC DAICEL CHIRALCEL IA, *n*-hexane/2-propanol = 70/30, flow rate = 1.0 mL/min, λ = 254 nm, t_R (major) = 4.96 min, t_R (minor) = 6.39 min.

IR (neat): 2965, 1740, 1682, 1612, 1526, 1466, 1350, 1263, 770 and 735 cm^{-1}.

^{1}H NMR (400 MHz, $CDCl_3$) δ = 7.34 – 7.27 (m, 3H), 7.24 – 7.20 (m, 1H), 7.18 –

7.12 (m, 2H), 6.81 (t, J = 7.8 Hz, 1H), 6.67 – 6.62 (m, 1H), 6.50 (d, J = 7.8 Hz, 1H), 5.90 (s, 1H), 5.65 (d, J = 7.8 Hz, 1H), 4.65 (s, 1H), 3.66 – 3.52 (m, 5H), 3.50 (s, 3H), 1.07 (s, 9H).

^{13}C{^{1}H} NMR (101 MHz, $CDCl_3$) δ = 169.4, 168.3, 151.4, 140.2, 135.2, 129.3, 128.8, 128.6, 128.3, 126.3, 124.0, 119.6, 117.8, 115.7, 99.8, 67.0, 59.1, 53.7, 53.4, 52.7, 52.4, 31.7.

HRMS (ESI-FT) calcd for $C_{26}H_{30}N_3O_4^+$ ([M+H$^+$]) = 448.2231, Found 448.2237.

Chiral HPLC spectrum **8v**:

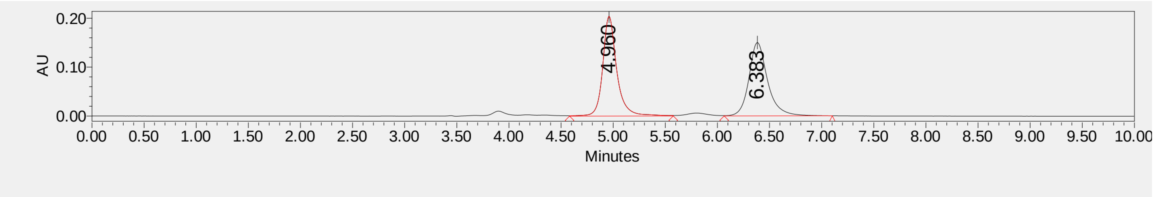

	Retention Time	Area	% Area
1	4.960	1911210	50.58
2	6.383	1867712	49.42

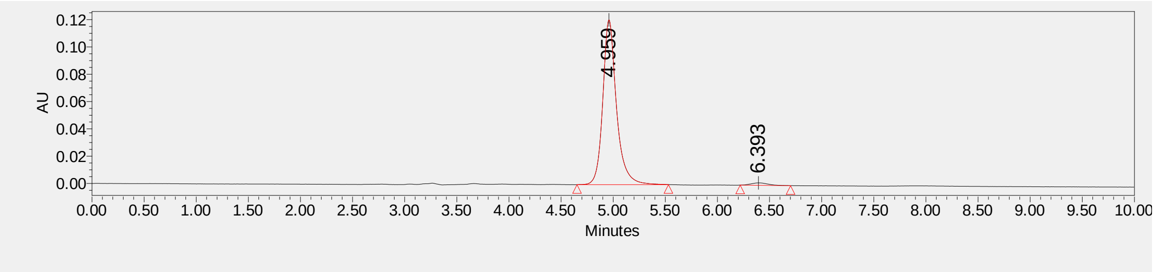

	Retention Time	Area	% Area
1	4.959	1096958	98.17
2	6.393	20398	1.83

Supplementary Table 10. Control experiment 1

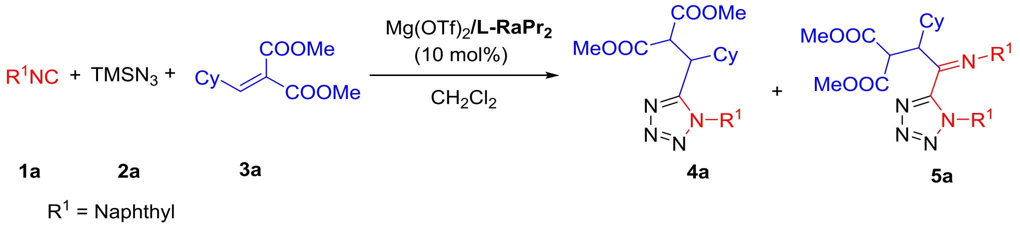

Entry	Conditions	Results
1	No **2a,** 30 °C, 3h	mess
2	No **3a,** 30 °C, 3h	nr
3	No **1a,** 30 °C, 3h	nr

Reactions were carried out with [**1a** (0.20 mmol), **2a** (0.24 mmol), **3a** (0.20 mmol)] and $Mg(OTf)_2$/**L-RaPr₂** (1.4:1.0, 10 mol%) in CH_2Cl_2 (1.0 mL) at 30 °C for 3 h.

Supplementary Discussion 3. It was proved that in the presence of chiral Lewis acid catalyst, no reaction occurred after mixing **1a** and **2a** or mixing **2a** and **3a**. Isocyanide **1a** could reacted with **3a** smoothly, however, only messy mixture was obtained at the end of reaction. These results implied that it is the addition of isocyanide **1a** to **3a** catalyzed by chiral Lewis acid which triggered this type of MCRs.

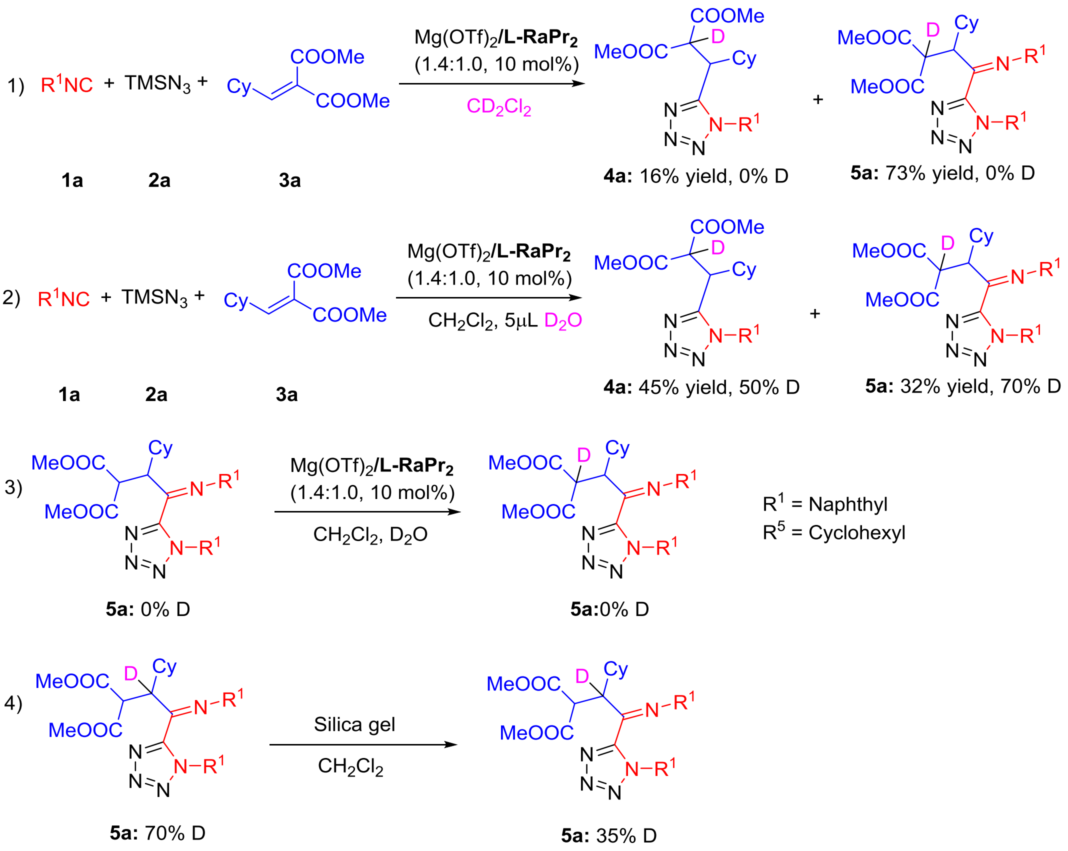

Supplementary Figure 8. Control experiment 2

Supplementary Discussion 4.

1) Reactions were carried out with **1a** (0.10 mmol), **2a** (0.12mmol), **3a** (0.10 mmol) and $Mg(OTf)_2$/**L-RaPr₂** (1.4:1.0, 10 mol%) in CD_2Cl_2 (1.0 mL) at 30 °C for 3 h.
   It proved that the hydrogen source of the product does not come from CD_2Cl_2.
2) Reactions were carried out with **1a** (0.10 mmol), **2a** (0.12mmol), **3a** (0.10 mmol), 5μL D_2O and $Mg(OTf)_2$/**L-RaPr$_2$** (1.4:1.0, 10 mol%) in CH_2Cl_2 (1.0 mL) at 30 °C for 3 h. The products were separated by silica gel column chromatography.
   This observation not only indicated that the proton transfer was facilitated by a trace amount of water but also suggested water has a remarkable influence on the pathway.
3) Reactions were carried out with **5a** (0.10 mmol), 5μL D_2O and $Mg(OTf)_2$/**L-RaPr$_2$** (1.4:1.0, 10 mol%) in CH_2Cl_2 (1.0 mL) at 30 °C for 3 h.
4) Reactions were carried out with **5a** (70% deuterium) and silica gel in CD_2Cl_2 (1.0 mL) at 30 °C for 3 h.
   It explained the reason of different deuterium of **4a** and **5a** in the reaction 2 and the deuterium is not stable.

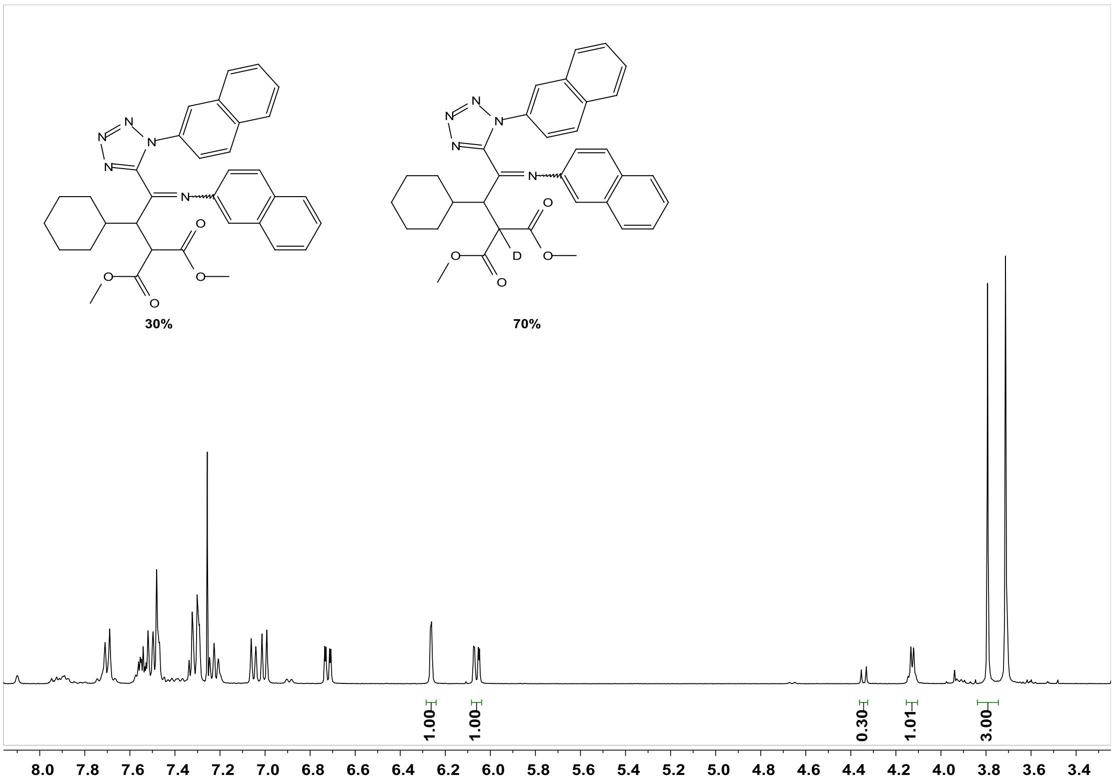

Supplementary Figure 9. ^{1}H spectra for deuterated **5a**

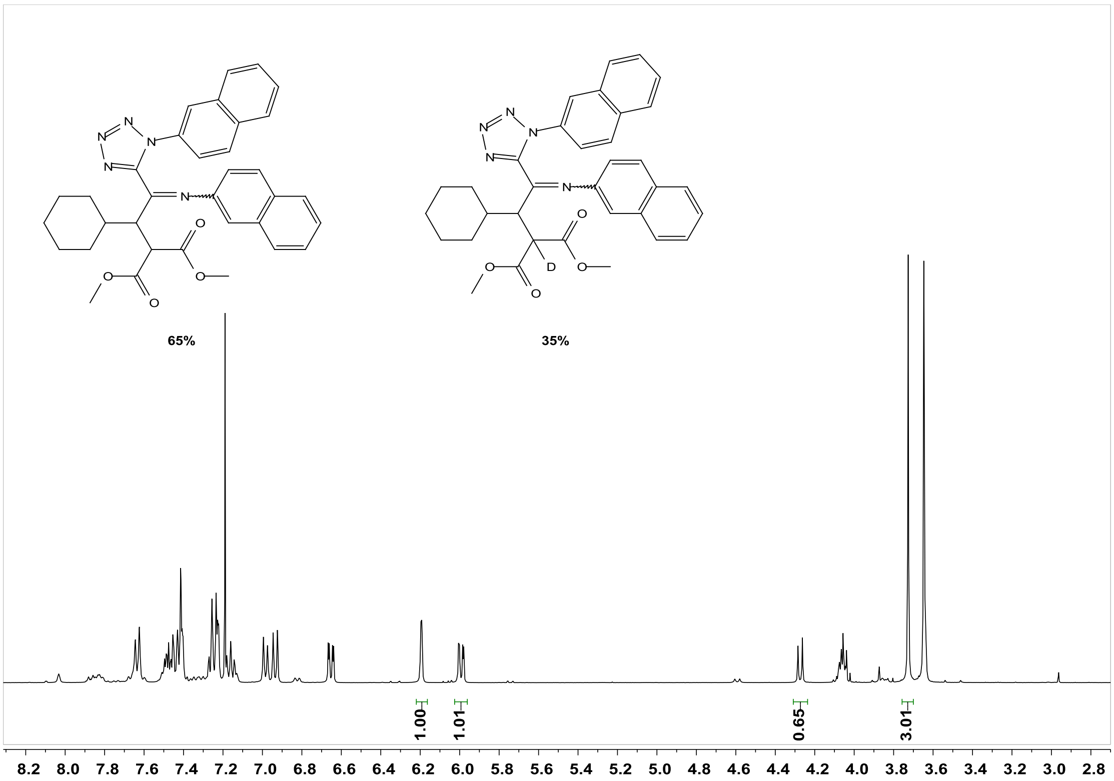

Supplementary Figure 10. ^{1}H spectra for deuterated **5a**

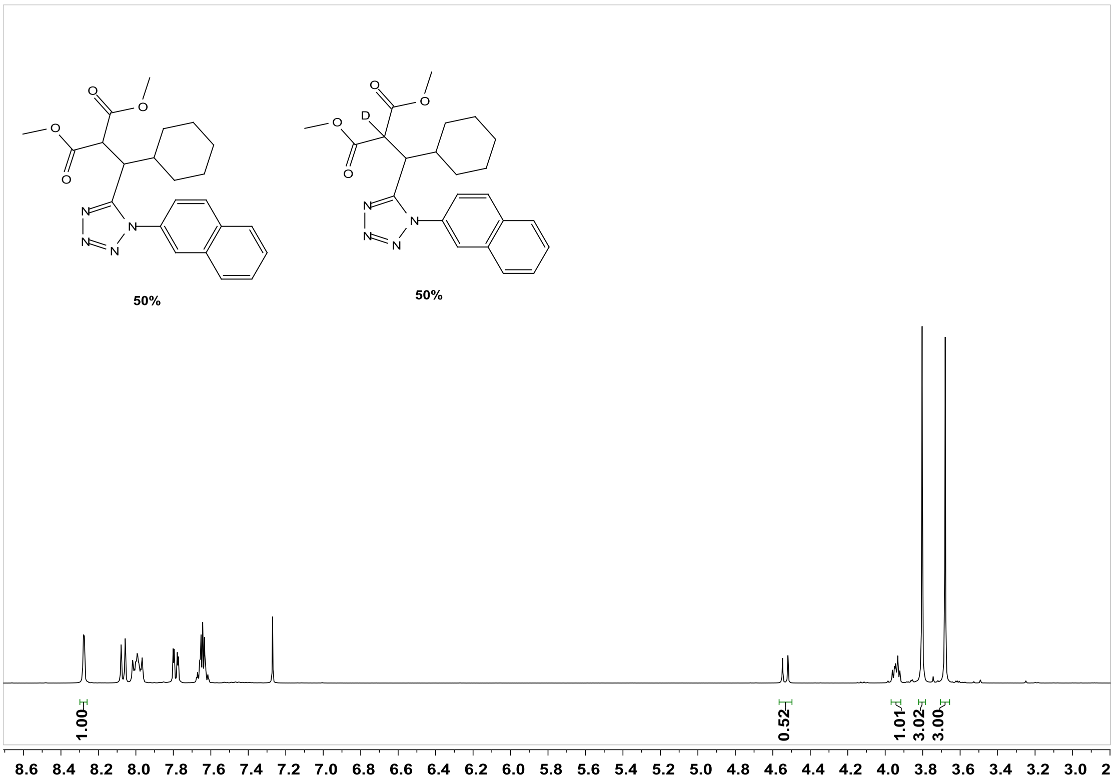

Supplementary Figure 11. ^{1}H spectra for deuterated **4a**

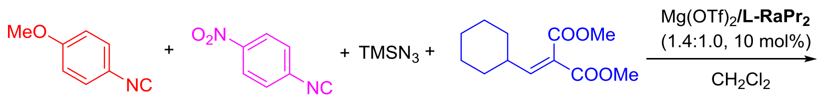

1c **1b** **2a** **3a**

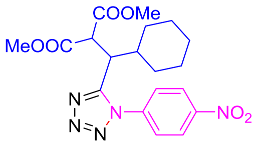

4B

HRMS (ESI-FT) calcd for $C_{19}H_{24}N_5O_6^+$ ([M+H$^+$]) = 418.1722, Found 418.1716.

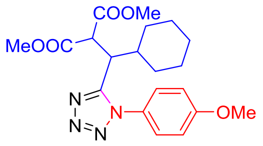

4A

HRMS (ESI-FT) calcd for $C_{20}H_{27}N_4O_5^+$ ([M+H$^+$]) = 403.1976, Found 403.1978.

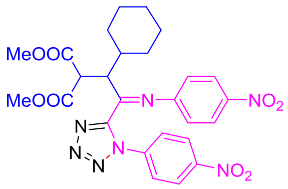

5B

HRMS (ESI-FT) calcd for$C_{26}H_{28}N_7O_8^+$ ([M+H$^+$]) = 566.1994, Found 566.1996.

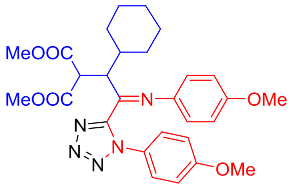

5A: main product

HRMS (ESI-FT) calcd for $C_{28}H_{34}N_5O_6^+$ ([M+H$^+$]) = 536.2504, Found 536.2512.

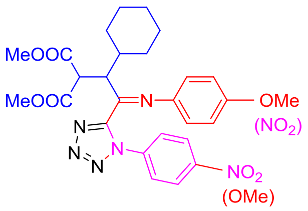

5C, 5D

HRMS (ESI-FT) calcd for $C_{27}H_{31}N_6O_7^+$ ([M+H$^+$]) = 551.2249, Found 551.2252.

Supplementary Figure 12. Control experiment 3

Reactions were carried out with **1b** (0.10 mmol), 1c (0.10 mmol) **2a** (0.24mmol), **3a** (0.20 mmol) and Mg(OTf)$_2$/**L-RaPr$_2$** (1.4:1.0, 10 mol%) in CH_2Cl_2 (1.0 mL) at 30 °C for 3 h.

Supplementary Discussion 5.

It proved the reaction both three-molecule reaction pathway and four-molecule reaction pathway shared with the same intermediate. Moreover, the results mentioned above also illustrated that electron-rich isocyanides were more reactive than electron-poor isocyanides in current system.

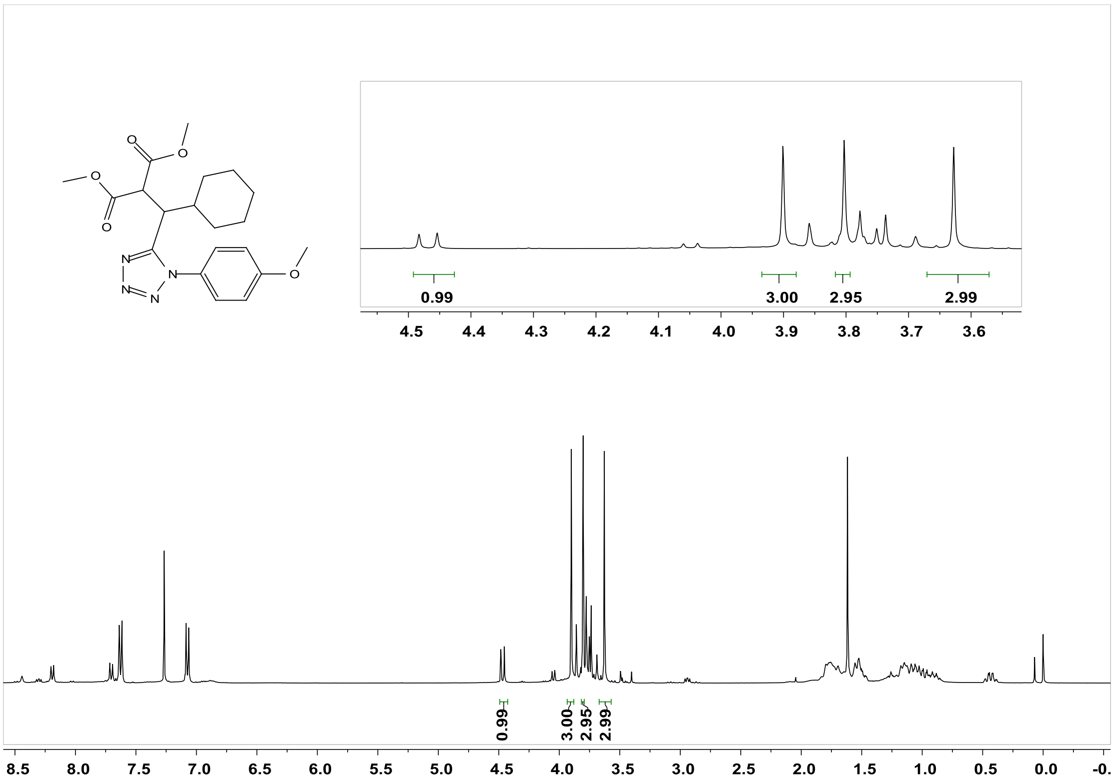

Supplementary Figure 13. ^{1}H spectra for product mixture 1

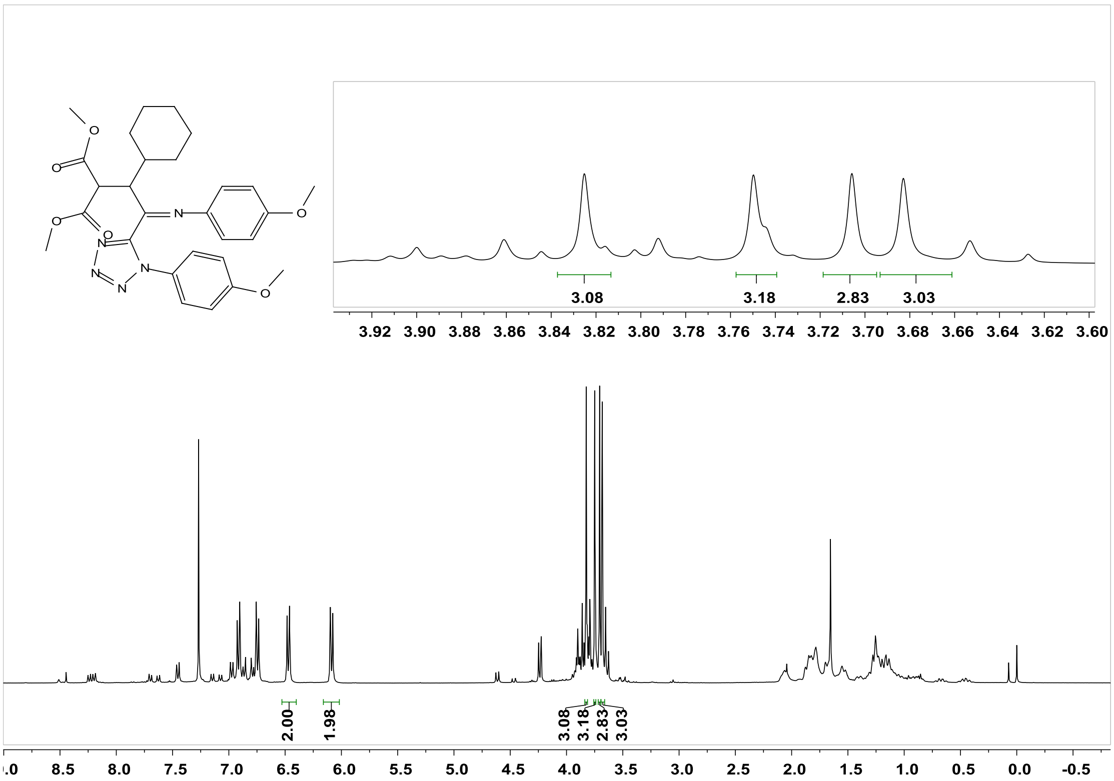

Supplementary Figure 14. ^{1}H spectra for product mixture 2

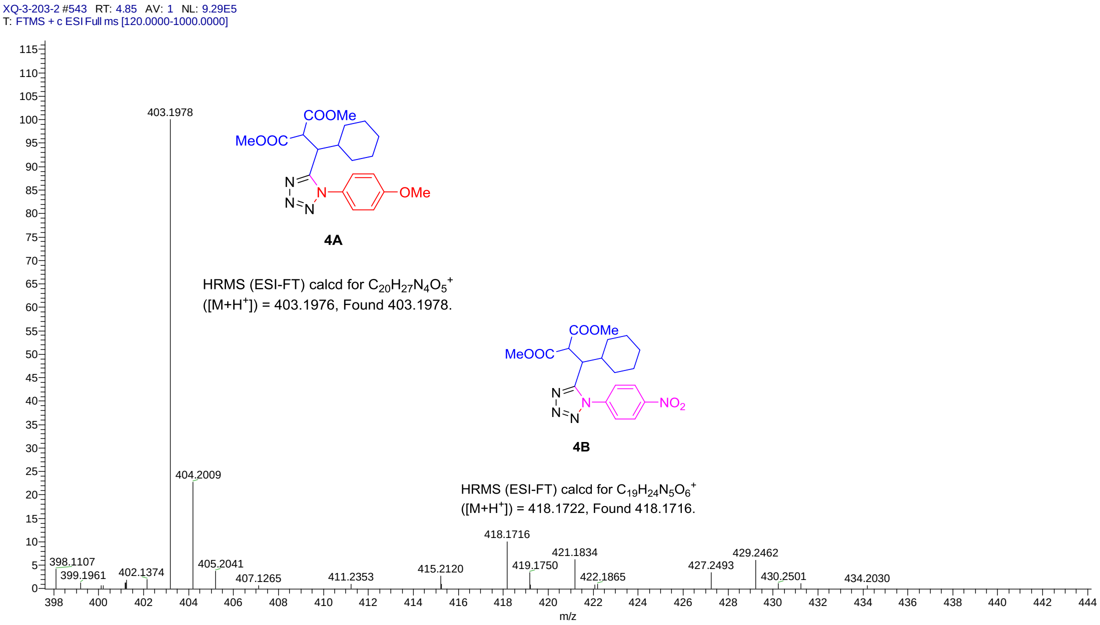

Supplementary Figure 15. HRMS for **4A**

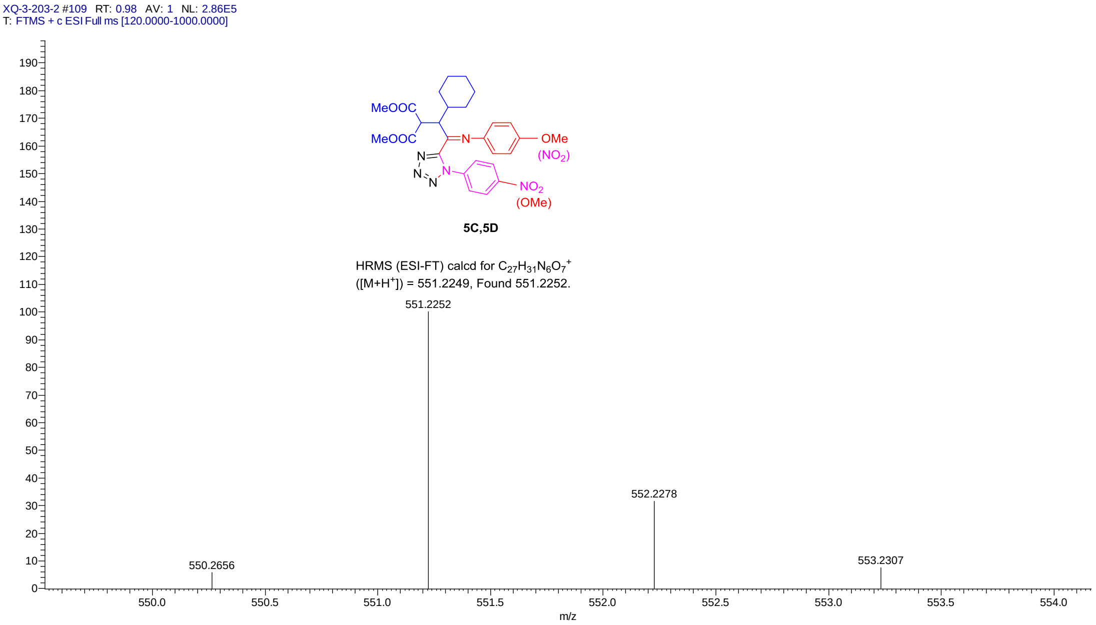

Supplementary Figure 16. HRMS for **5C** and **5D**

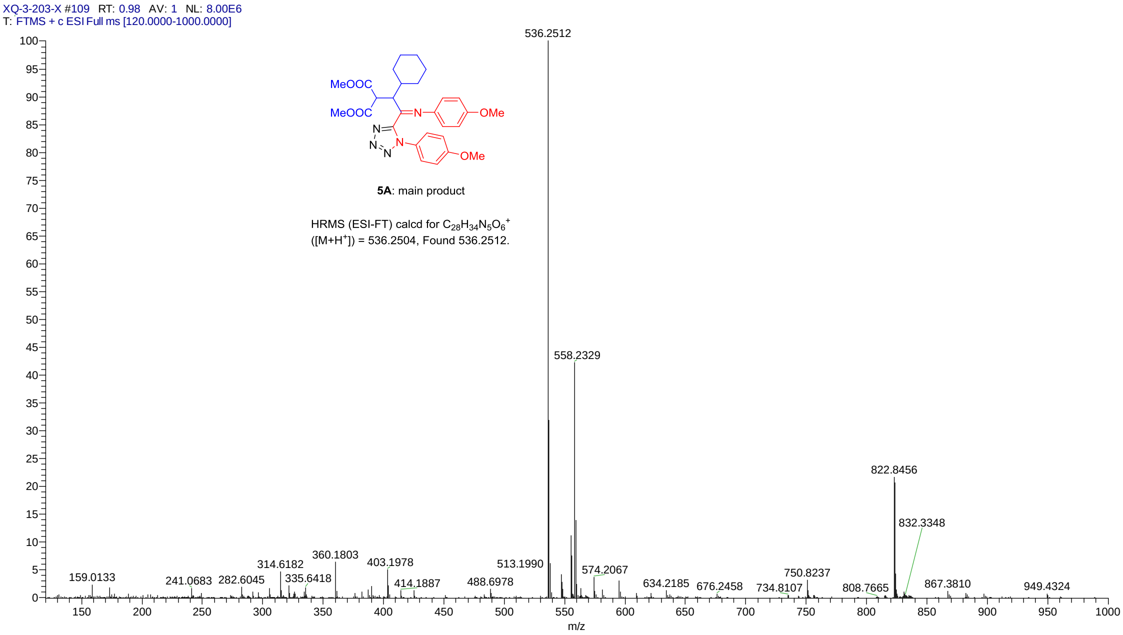

Supplementary Figure 17. HRMS for **5A**

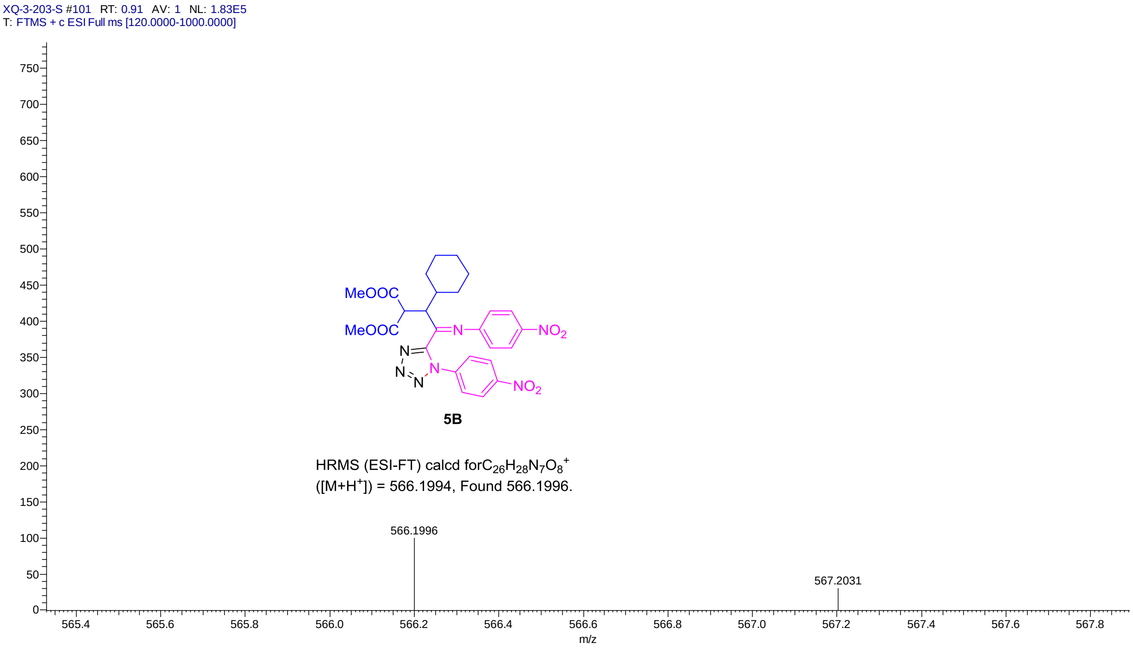

Supplementary Figure 18. HRMS for **5B**

Supplementary Table 11. Influence of metal saults and base

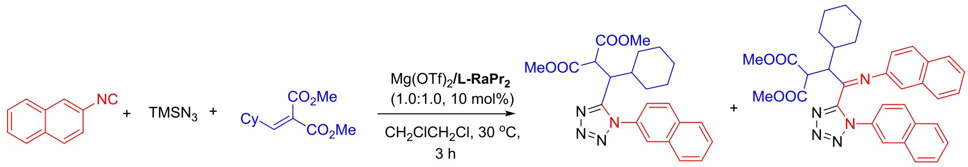

Entry[a]	Additive	Yield of **4a** (%)[b]	Yield of **5a** (%)[b]	e.r. of **4a** [c]	e.r. of **5a** [c]
1	-	38	51	83.5:16.5	96:4
2	$Ca(OTf)_2$	-	81	-	94:6
3[b]	LiOTf	-	88	-	94:6
4[b]	NaOTf	-	86	-	94:6
5	$Mg(ClO_4)_2$	-	75	-	92.5:7.5
6	$Mg(NTf)_2$	no	92	-	90.5:9.5
7	Et_3N	53	no	86:14	-
8	$^iPr_2NEt$	45	32	82:16	91.5:8.5
9	DMAP	44	35	82:16	96:4
10	DBACO	76	-	86:14	-
11[c]	Et_3N	52	-	70:30	-

[a] Reaction conditions: Unless otherwise noted, all reactions were carried out with **1a** (0.10 mmol), **2a** (0.15mmol), **3a** (0.15 mmol), additive (0.005 mmol) and $Mg(OTf)_2$/**L-RaPr$_2$** (1.0:1.0, 10 mol %) in CH_2ClCH_2Cl (1.0 mL) at 30 ℃ for 3 h. [b] addtitive is 0.01 mmol. [c] The reaction was performed in CH_2Cl_2 at -40 °C for 2days and -20 °C for 3 days. no= not detect

Supplementary Discussion 6. Excess metal salts play a role in accelerating the production of products. Excess **L-RaPr$_2$** plays similar roles to base, but the enantioselectivity decreases when the temperature decreases to Et_3N.

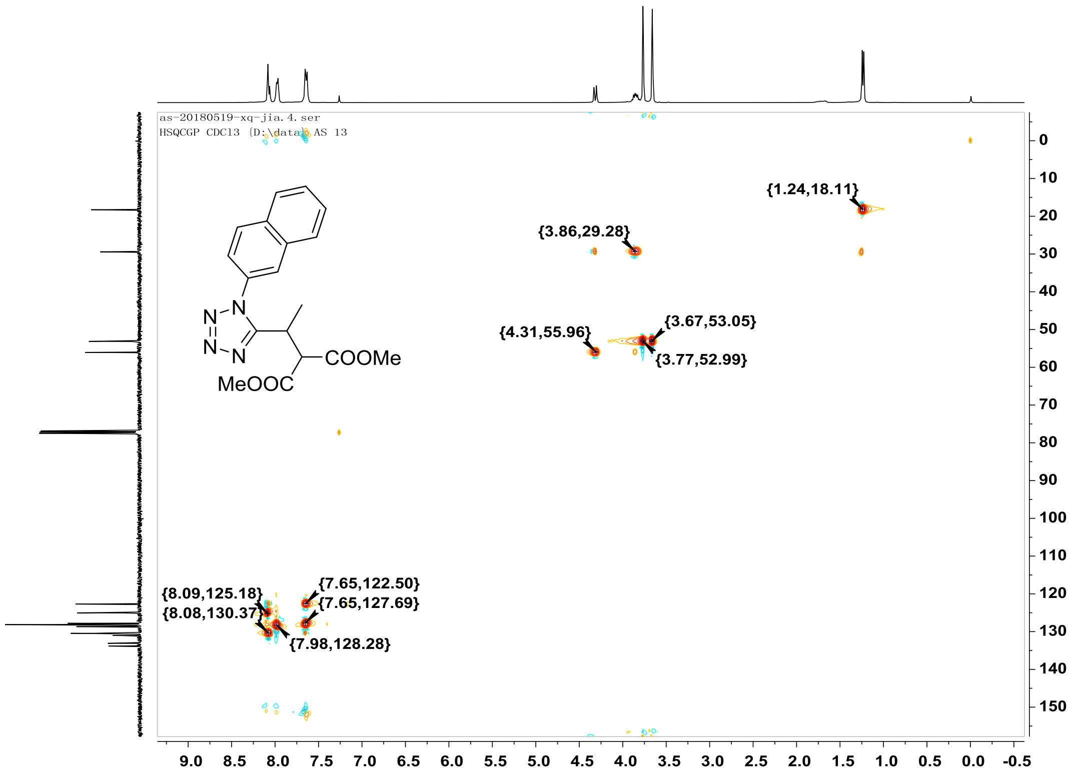

Supplementary Figure 19. HSQCGP of **4k**

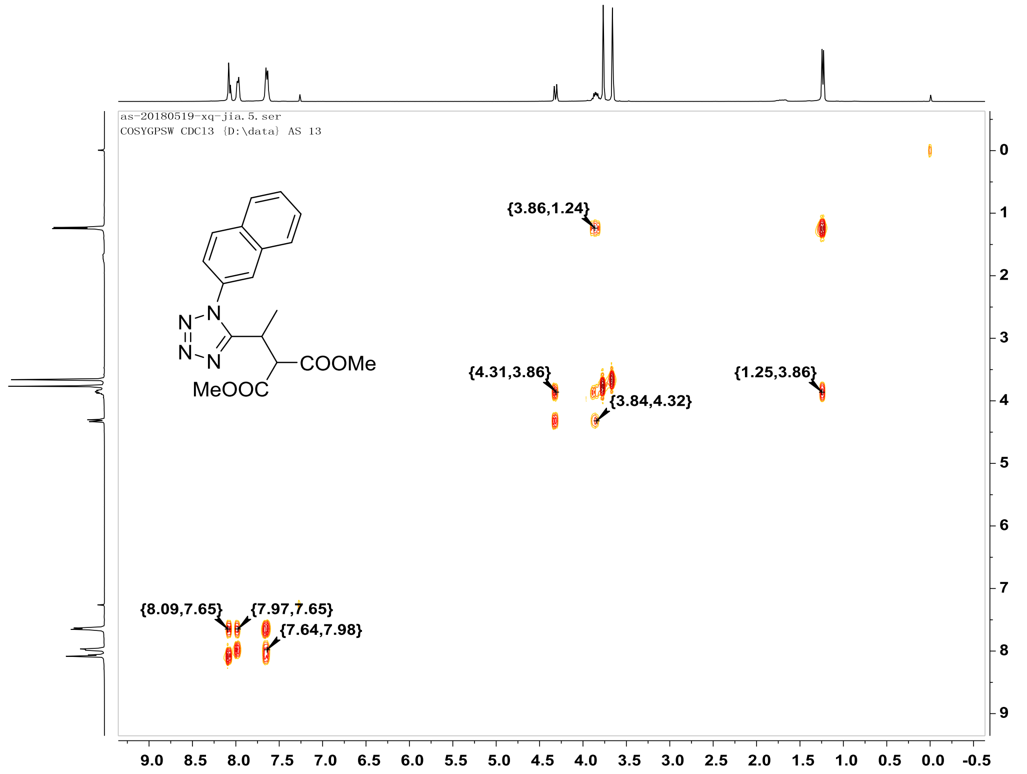

Supplementary Figure 20. COSYGPSW of **4k**

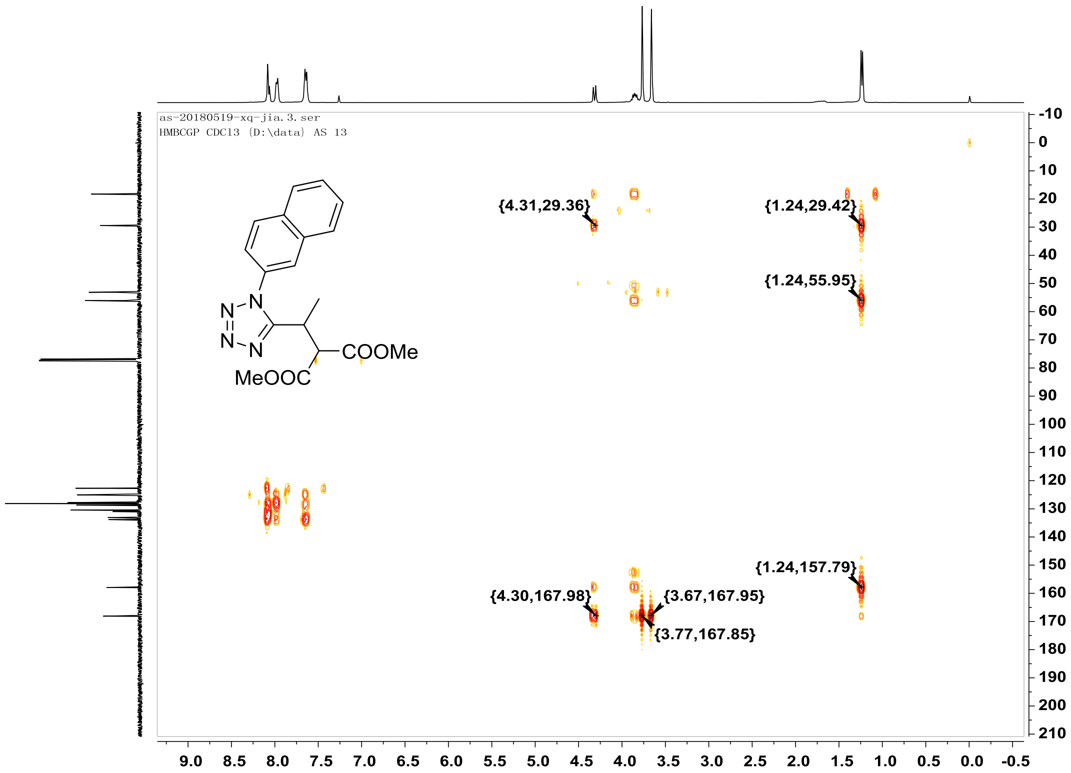

Supplementary Figure 21. HMBCGP of **4k**

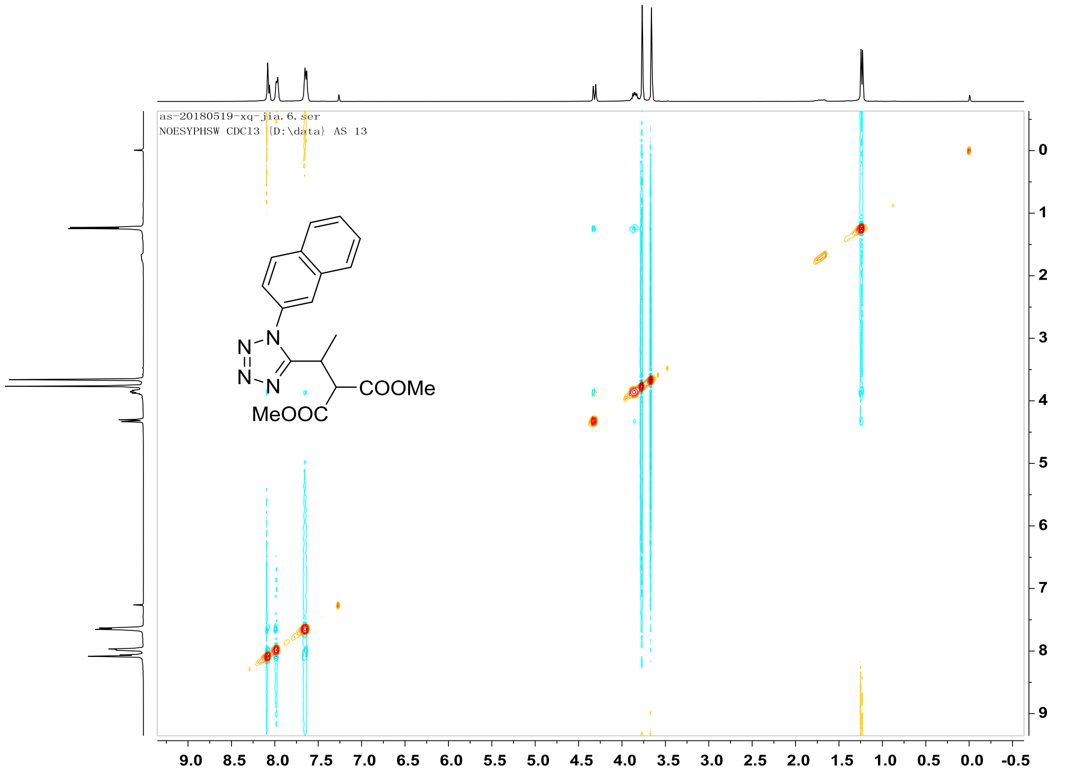

Supplementary Figure 22. NOESYPHSW of **4k**

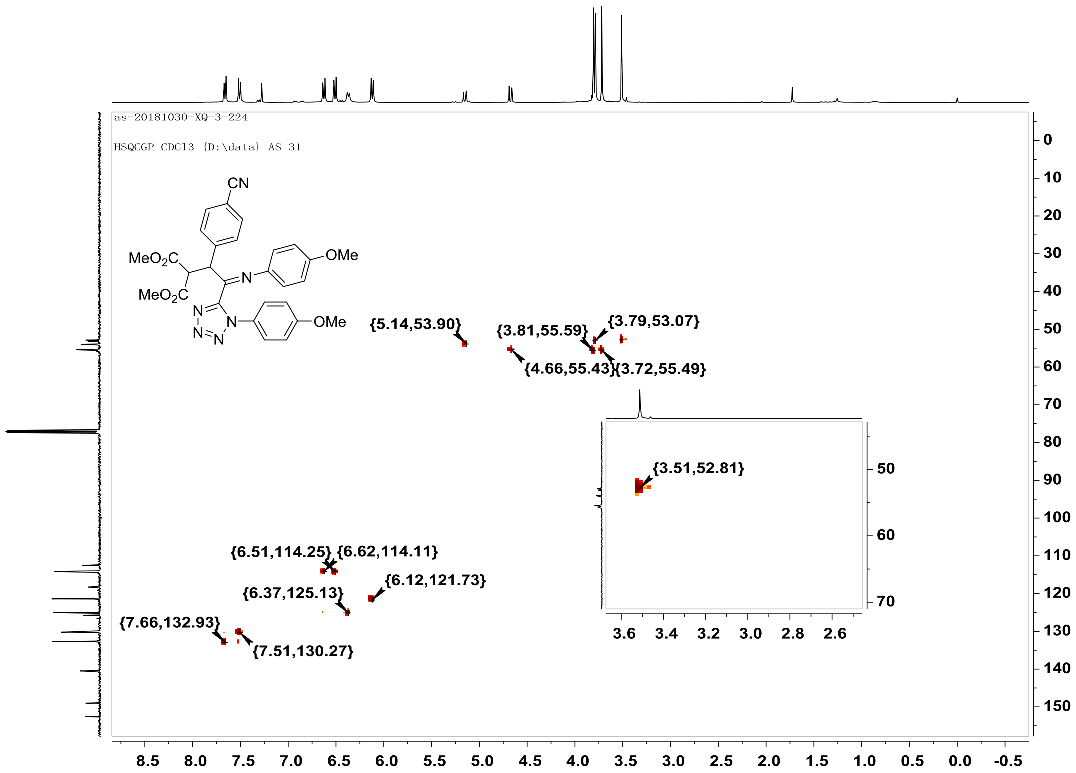

Supplementary Figure 23. HSQCGP of **5x**

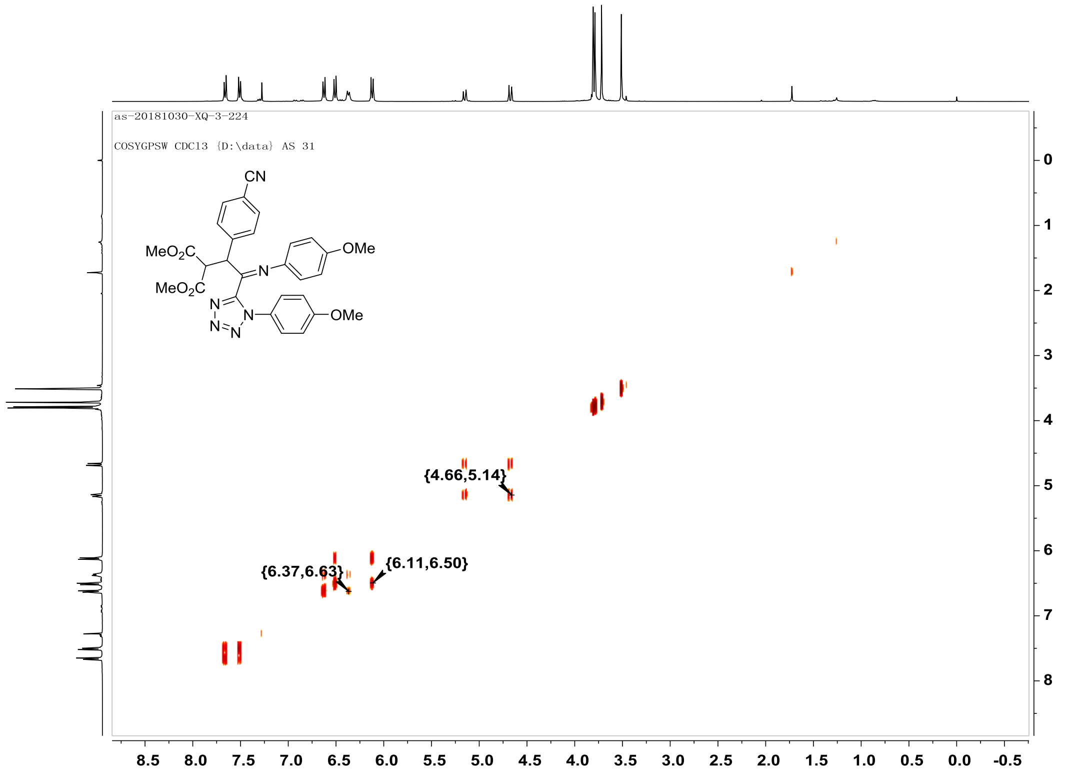

Supplementary Figure 24. COSYGPSW of **5x**

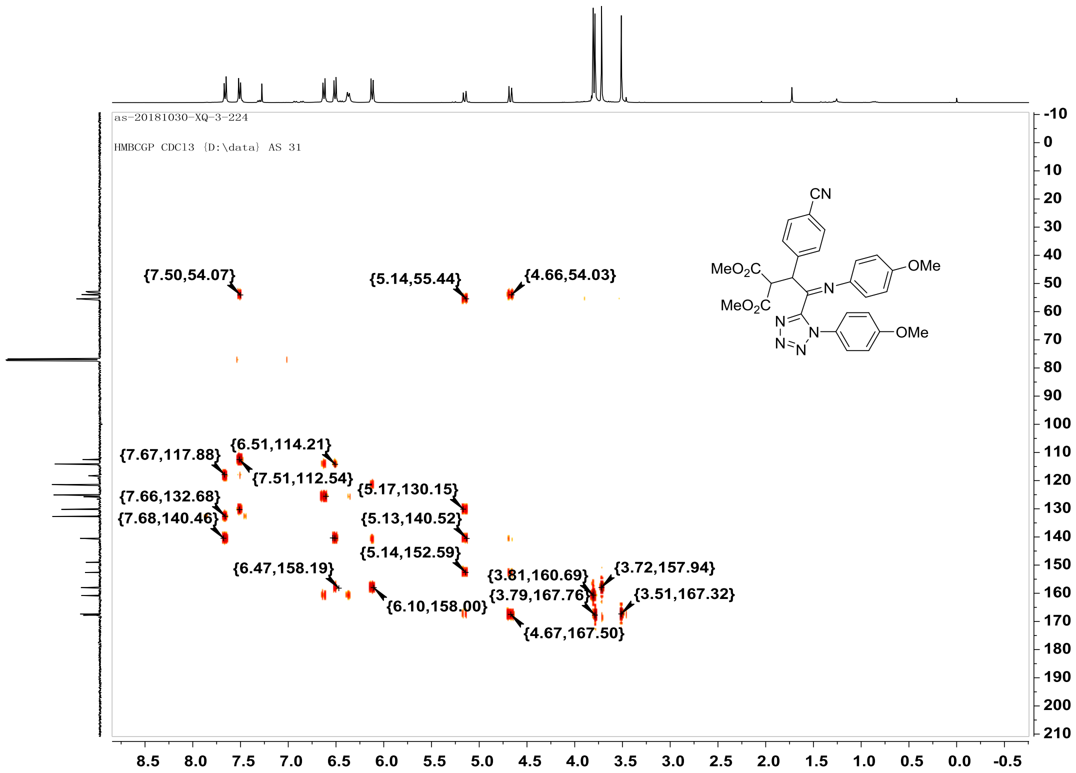

Supplementary Figure 25. HMBCGP of **5x**

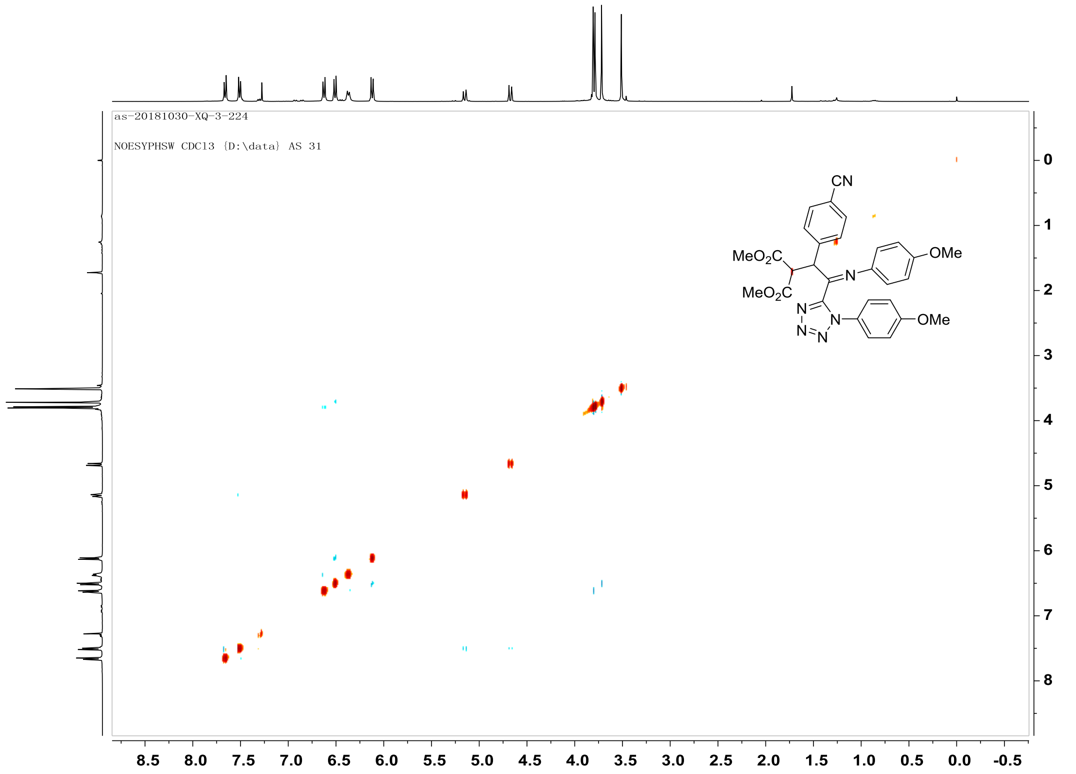

Supplementary Figure 26. NOESYPHSW of **5x**

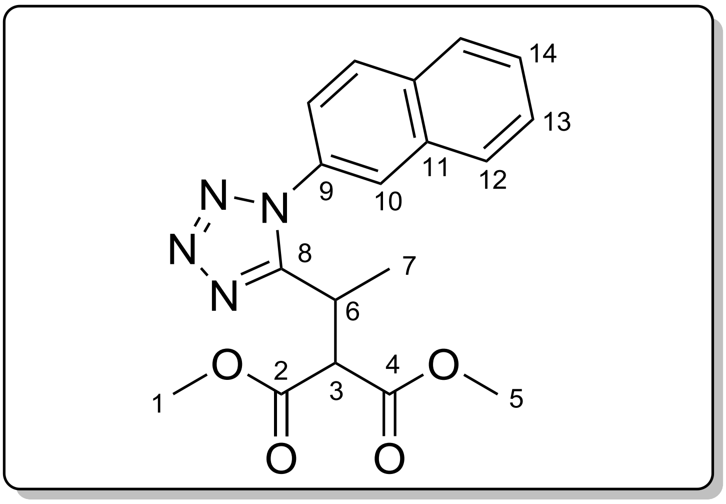

1: 3.77, 53.0; 2: 168.0;
3: 4.31, 56.0; 4: 167.9;
5: 3.67, 53.1; 6: 3.86, 29.3;
7: 1.24, 18.1; 8: 157.8;

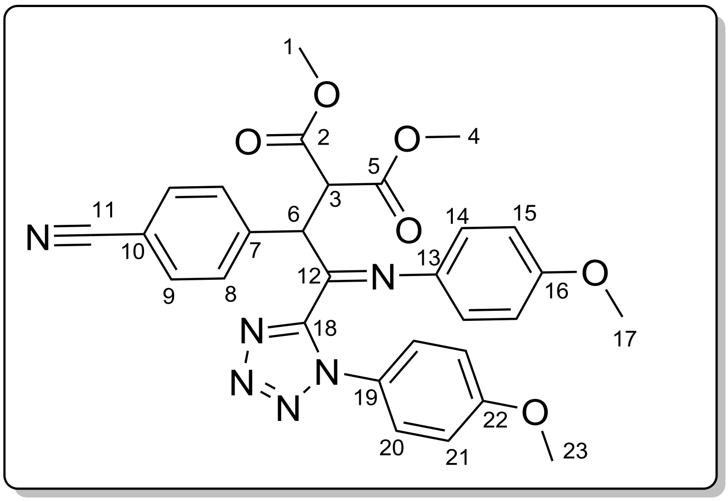

1: 3.77, 53.0; 2: 167.7;
3: 4.66, 55.4; 4: 3.50, 52.8;
5: 167.3; 6: 5.14, 53.9;
7: 140.3; 8: 7.49, 130.1;
9: 7.64, 132.6; 10: 112.5;
11: 118.4; 12: 148.9;
13: 140.4; 14: 6.10, 121.3;
15: 6.50, 114.1; 16: 157.9;
17: 3.70, 55.4; 18: 152.6;
19: 125.7; 20: 6.35, 125.0;
21: 6.61, 114.0; 22: 160.7;
23: 3.79, 55.6;

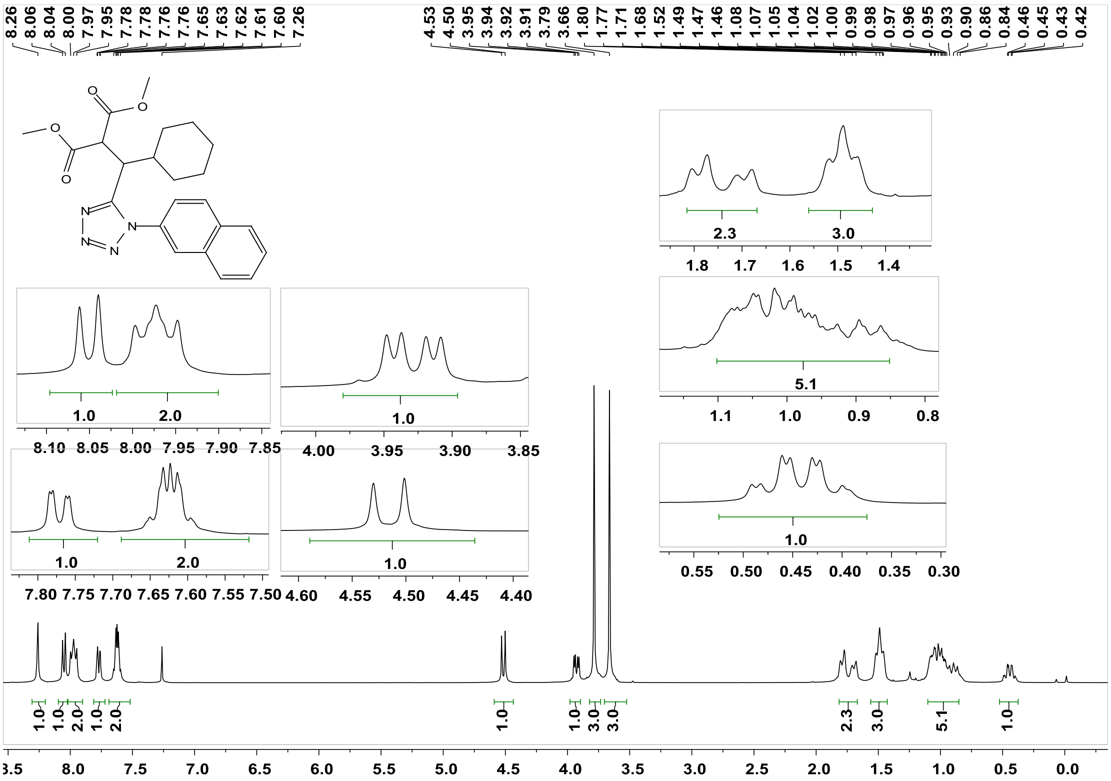

Supplementary Figure 27. ^{1}H NMR spectra for product **4a**

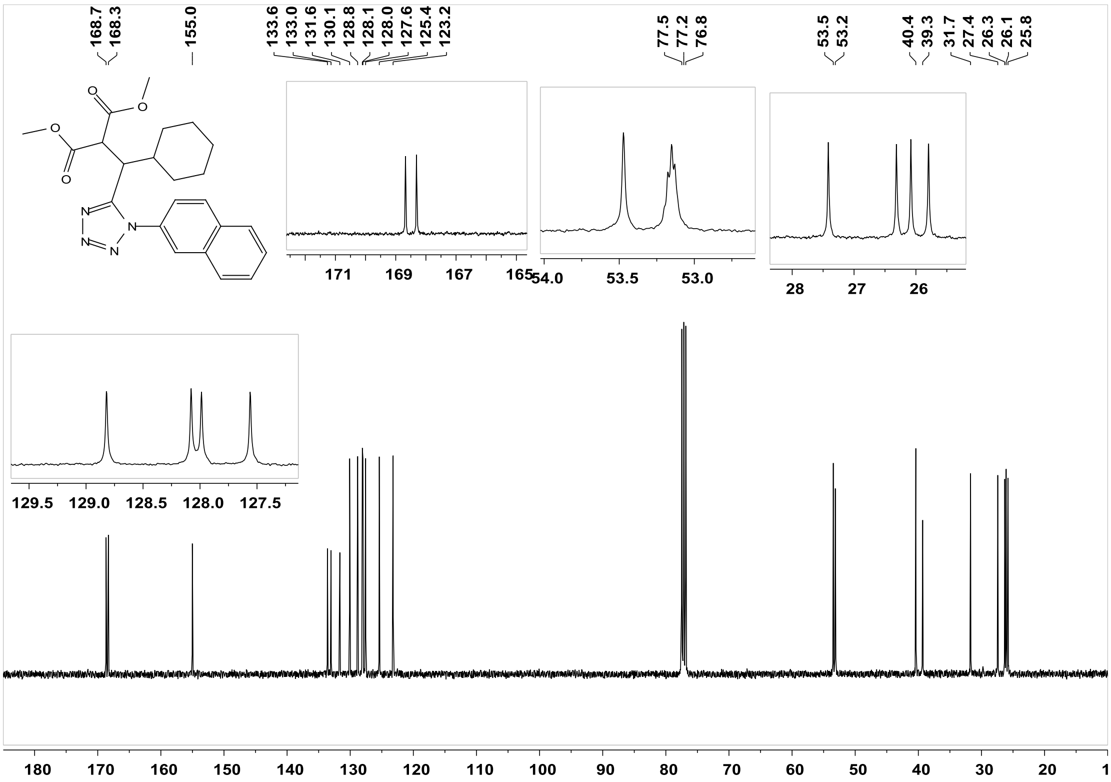

Supplementary Figure 28. ^{13}C NMR spectra for product **4a**

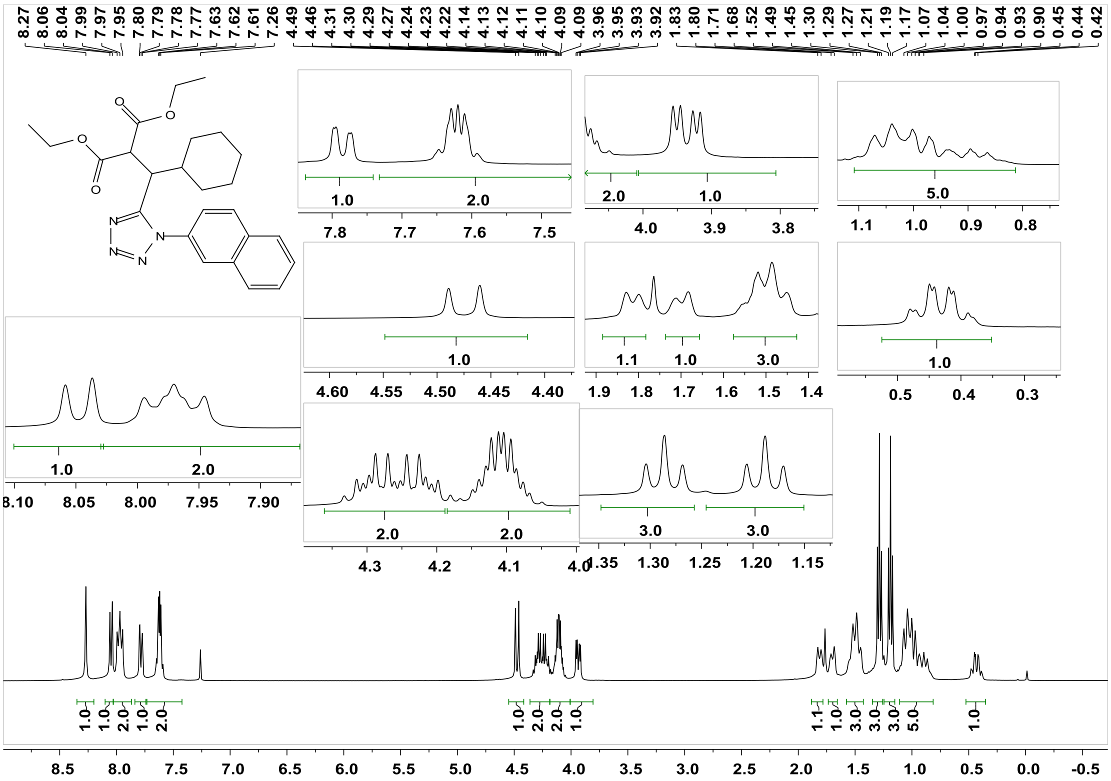

Supplementary Figure 29. ^{1}H NMR spectra for product **4b**

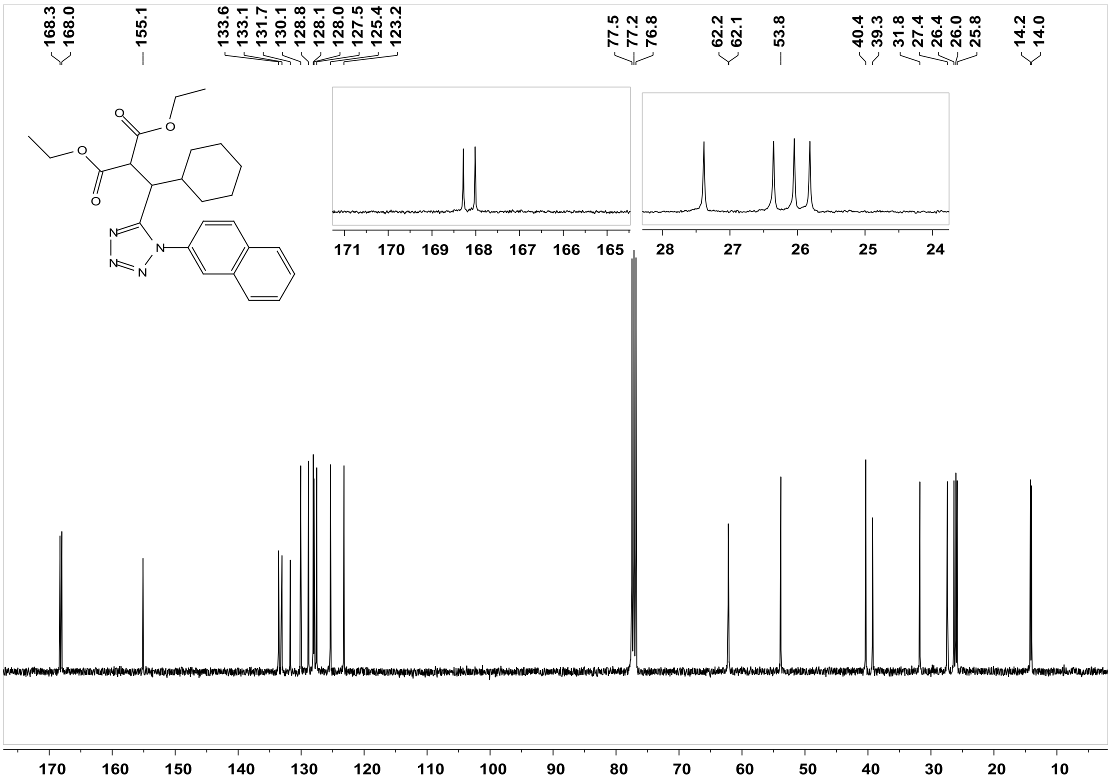

Supplementary Figure 30. ^{13}C NMR spectra for product **4b**

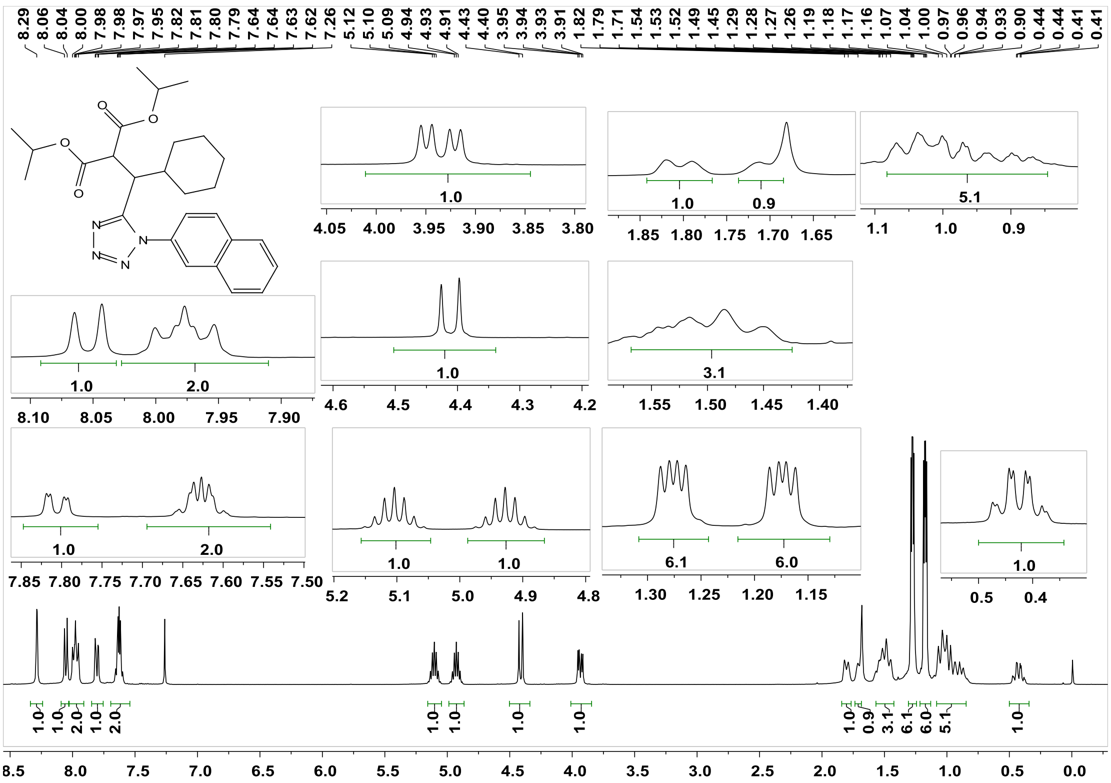

Supplementary Figure 31. ^{1}H NMR spectra for product **4c**

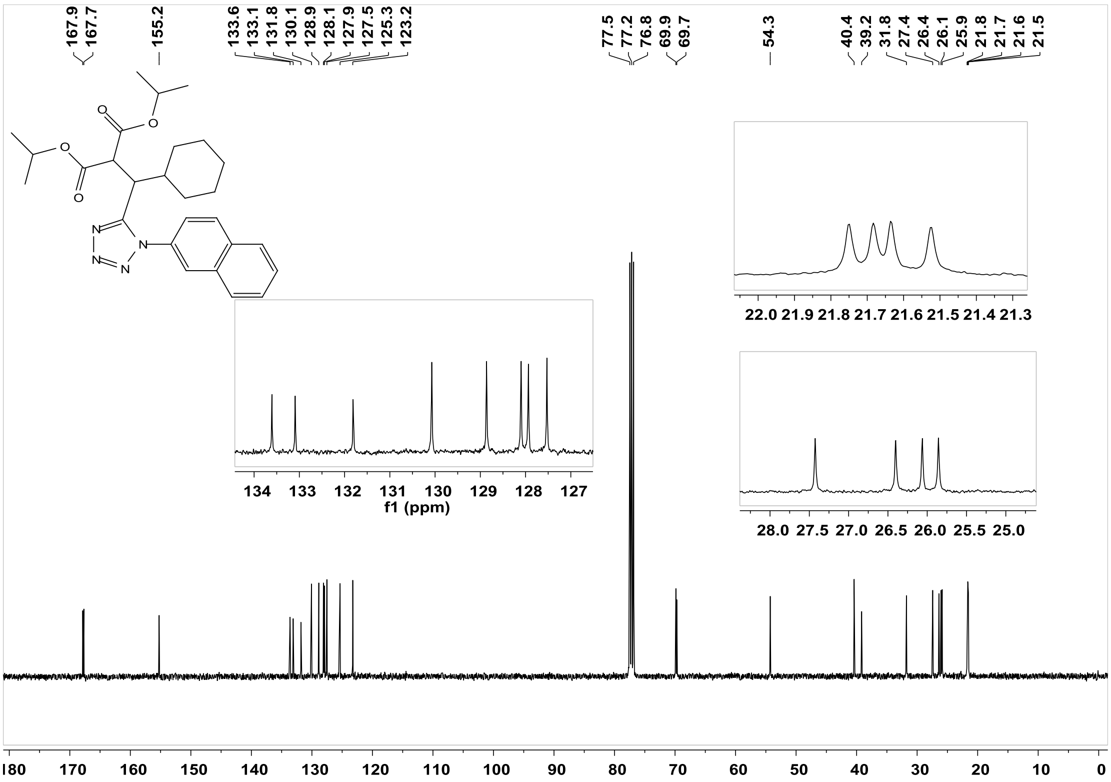

Supplementary Figure 32. ^{13}C NMR spectra for product **4c**

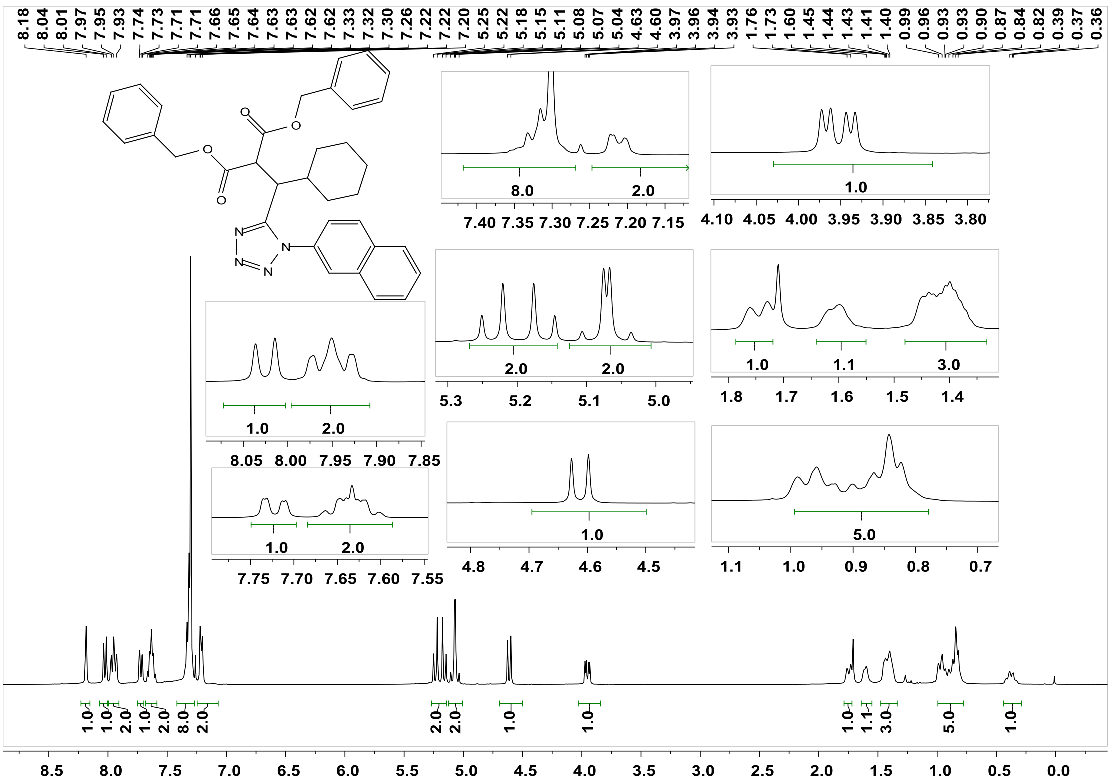

Supplementary Figure 33. ^{1}H NMR spectra for product **4d**

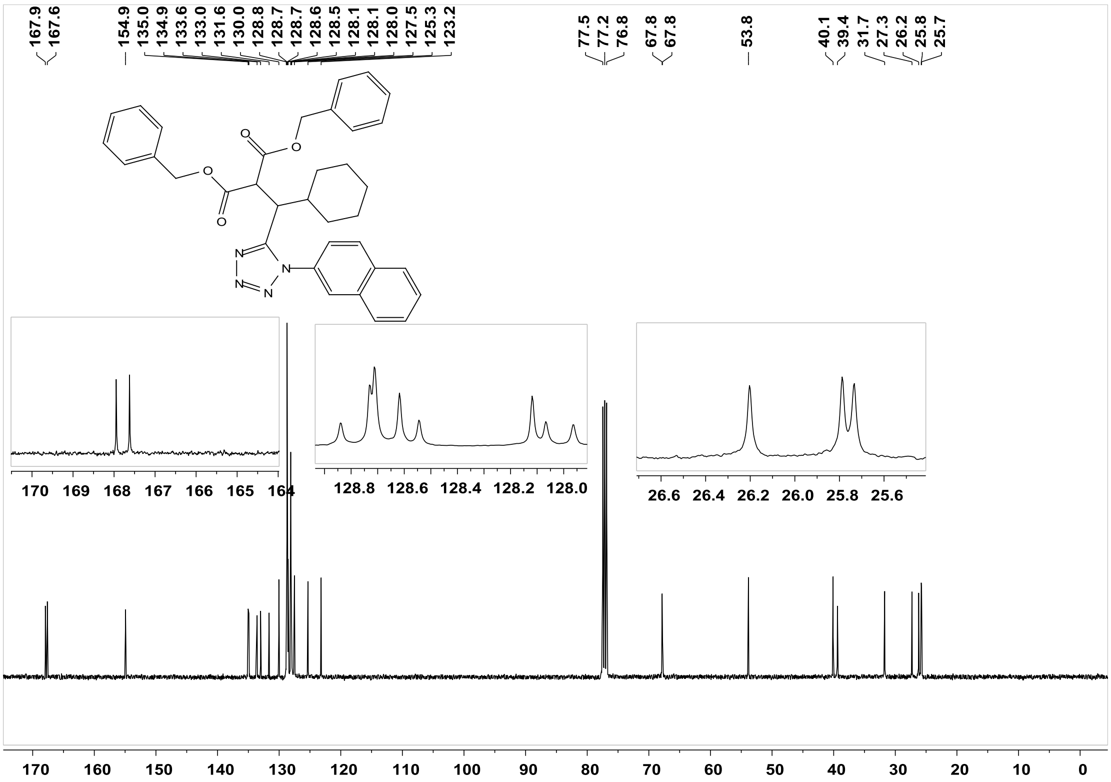

Supplementary Figure 34. ^{13}C NMR spectra for product **4d**

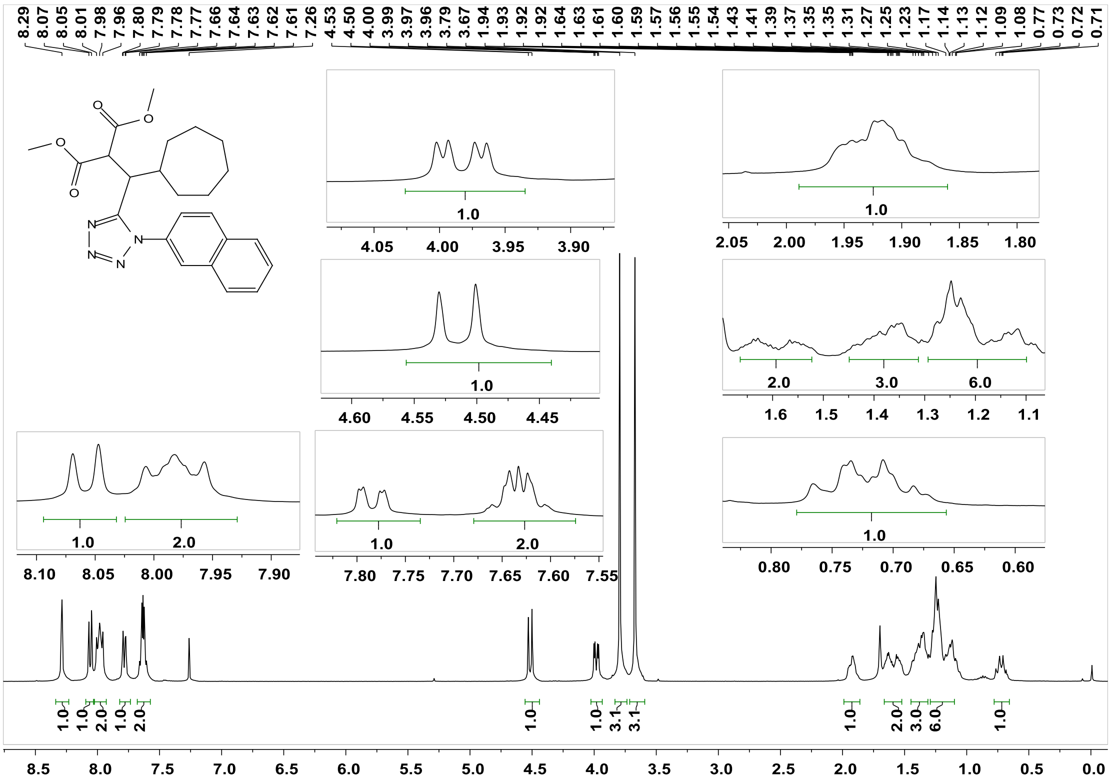

Supplementary Figure 35. ^{1}H NMR spectra for product **4e**

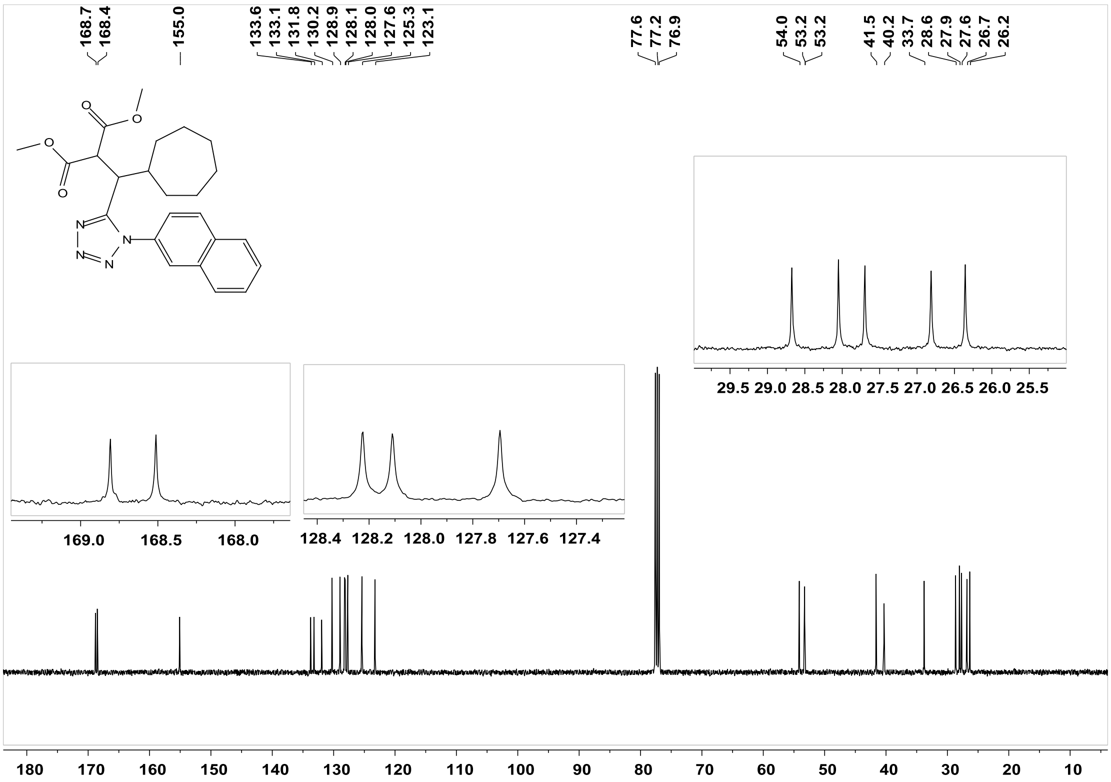

Supplementary Figure 36. ^{13}C NMR spectra for product **4e**

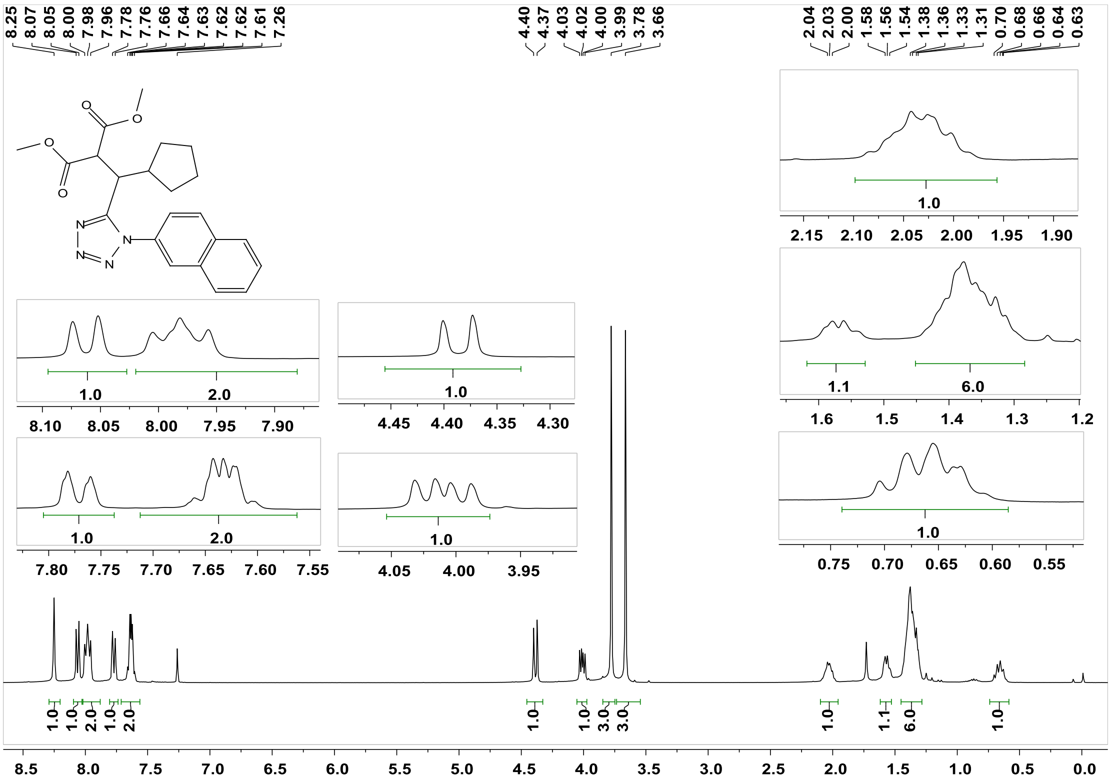

Supplementary Figure 37. ^{1}H NMR spectra for product **4f**

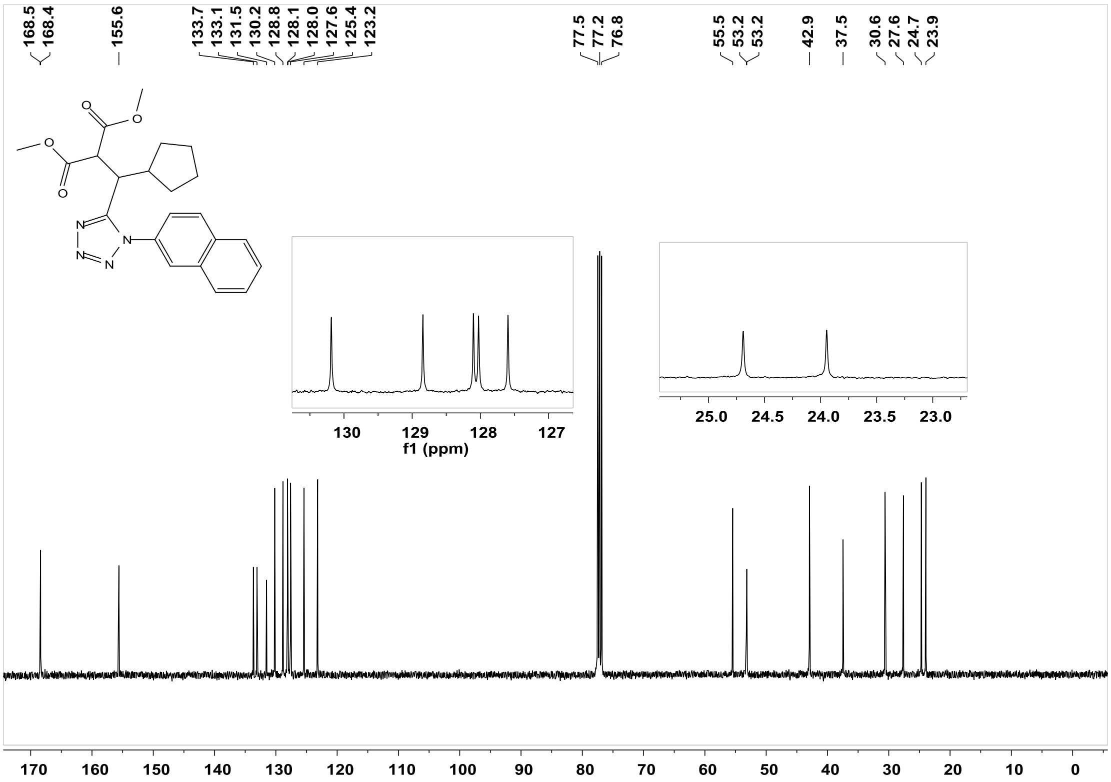

Supplementary Figure 38. ^{13}C NMR spectra for product **4f**

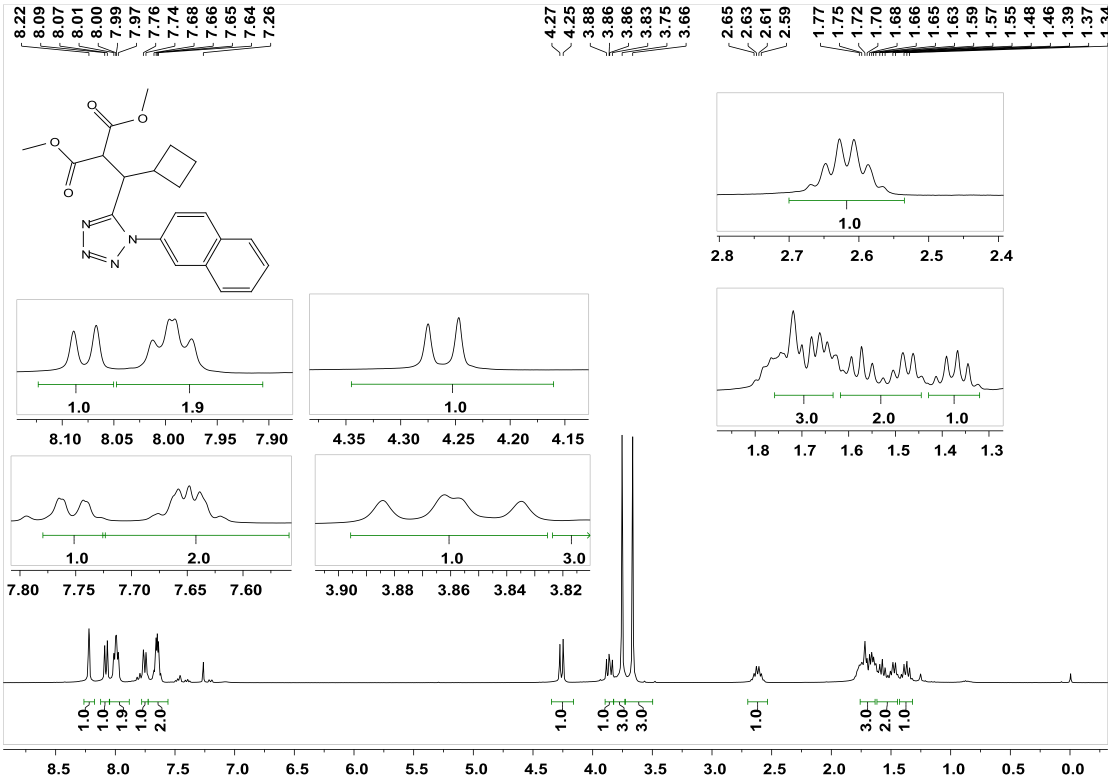

Supplementary Figure 39. ^{1}H NMR spectra for product **4g**

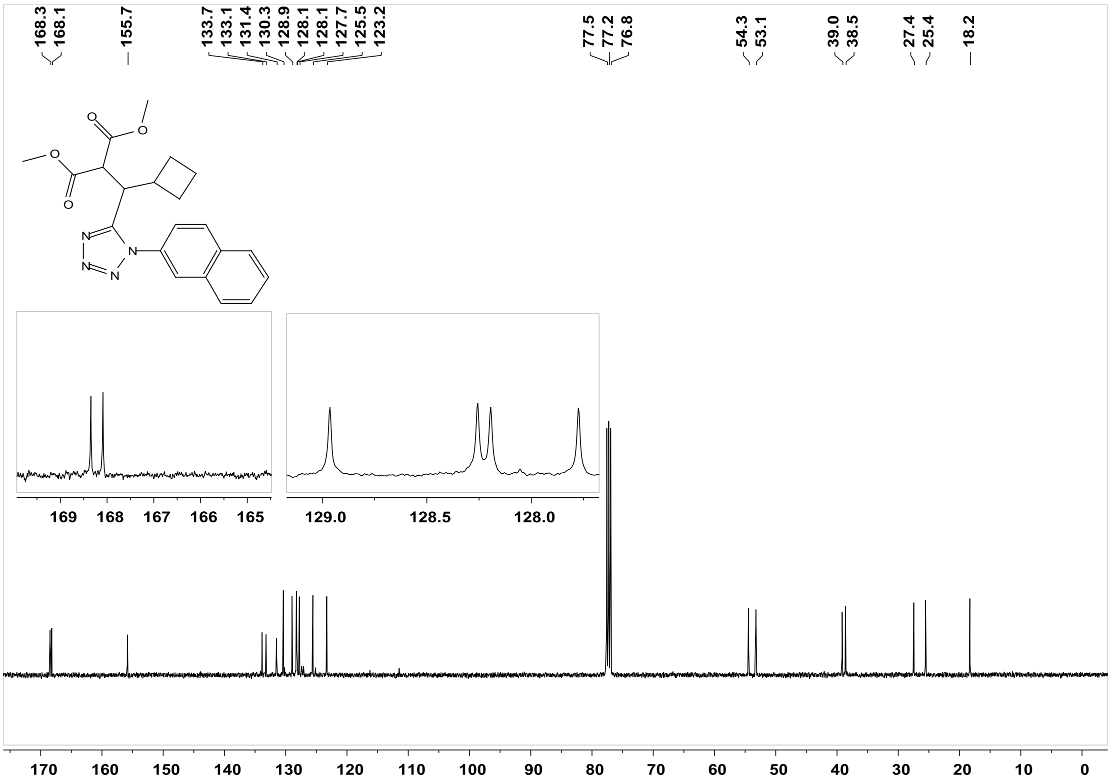

Supplementary Figure 40. ^{13}C NMR spectra for product **4g**

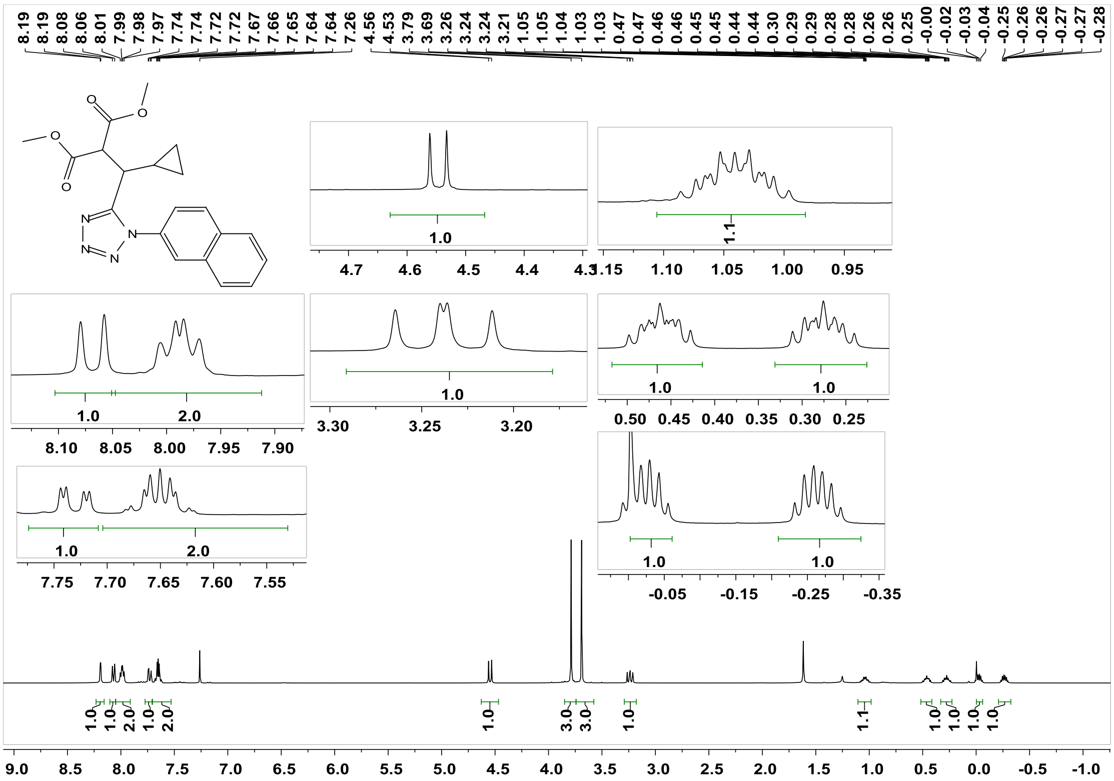

Supplementary Figure 41. ^{1}H NMR spectra for product **4h**

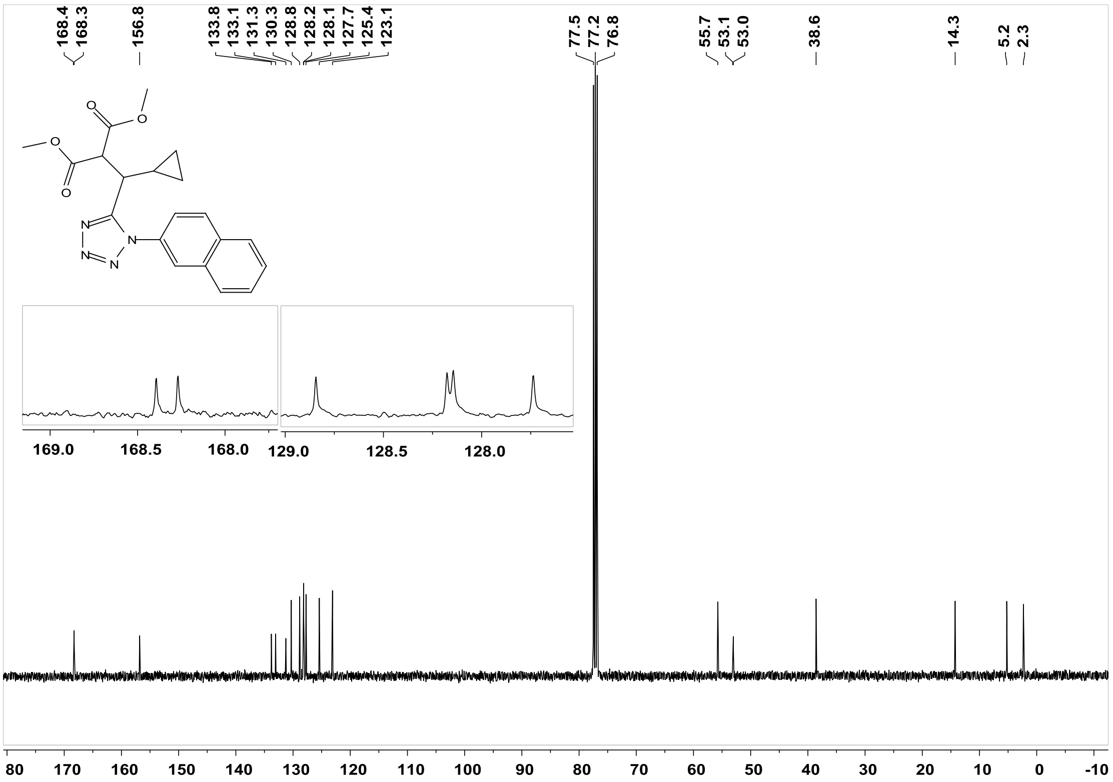

Supplementary Figure 42. ^{13}C NMR spectra for product **4h**

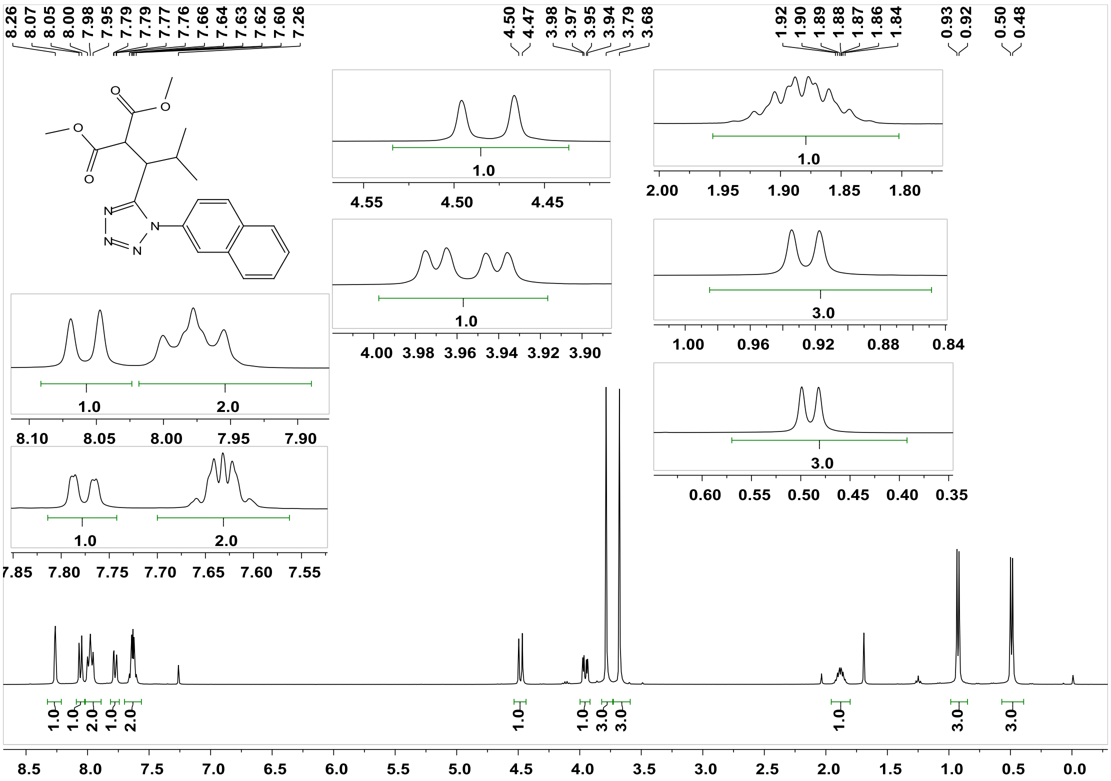

Supplementary Figure 43. ^{1}H NMR spectra for product **4i**

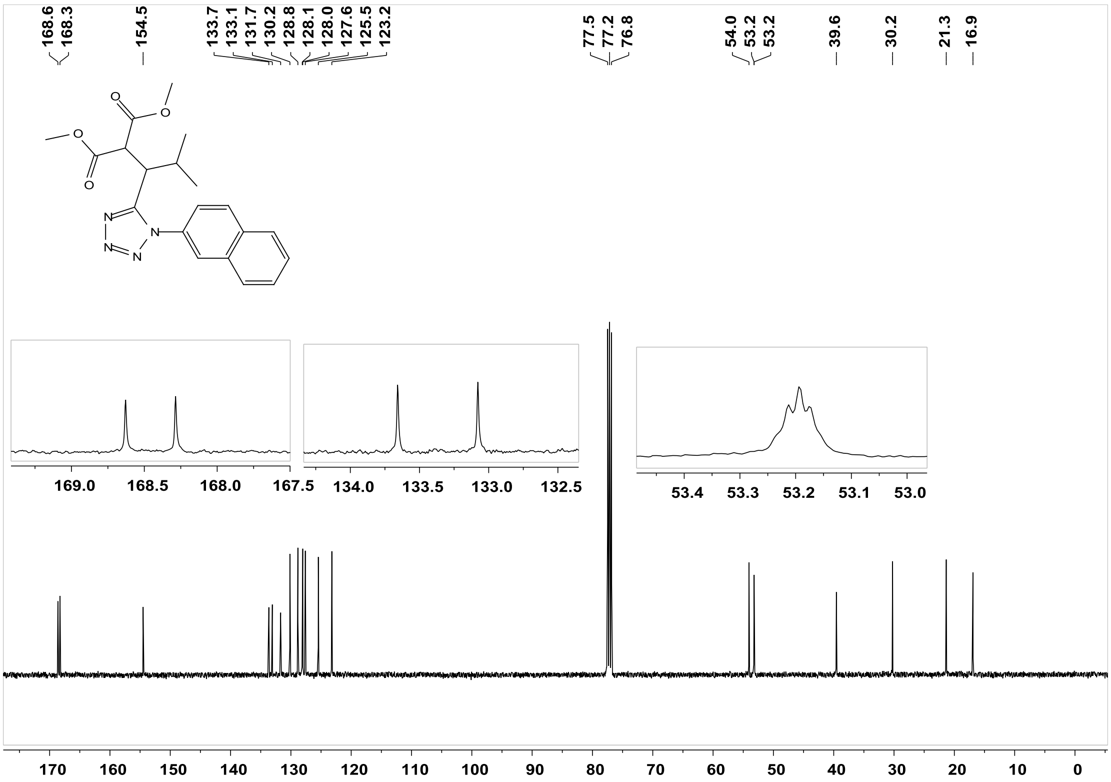

Supplementary Figure 44. ^{13}C NMR spectra for product **4i**

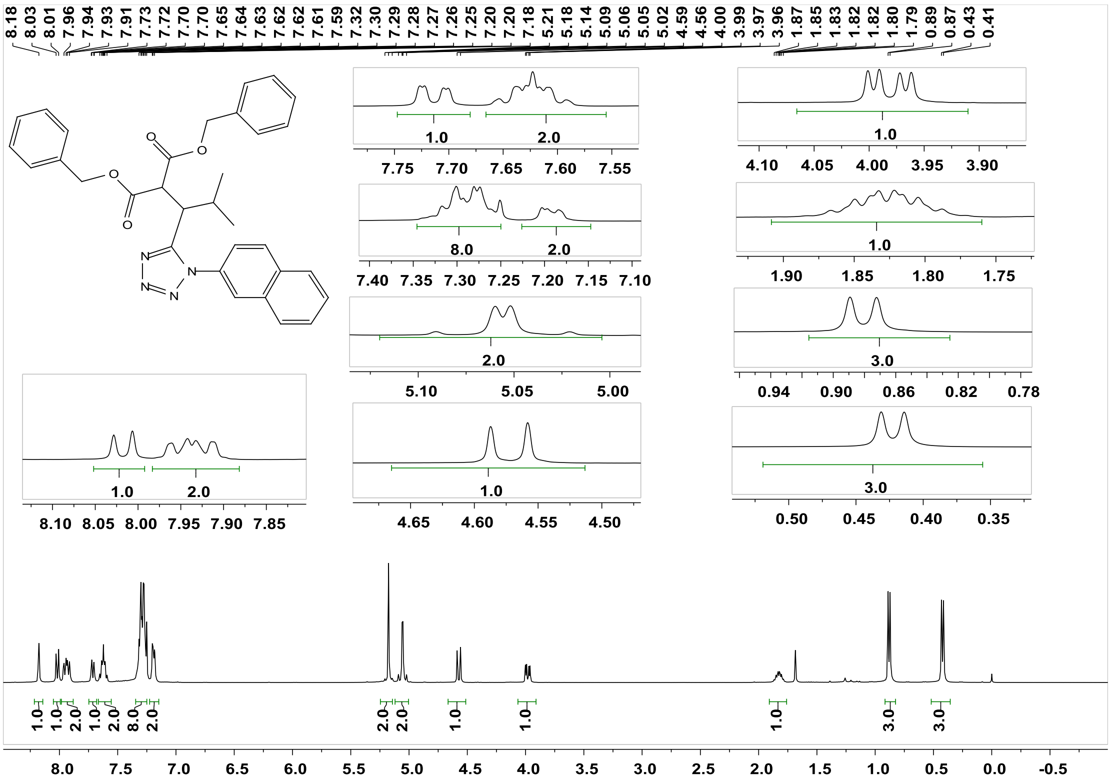

Supplementary Figure 45. ^{1}H NMR spectra for product **4j**

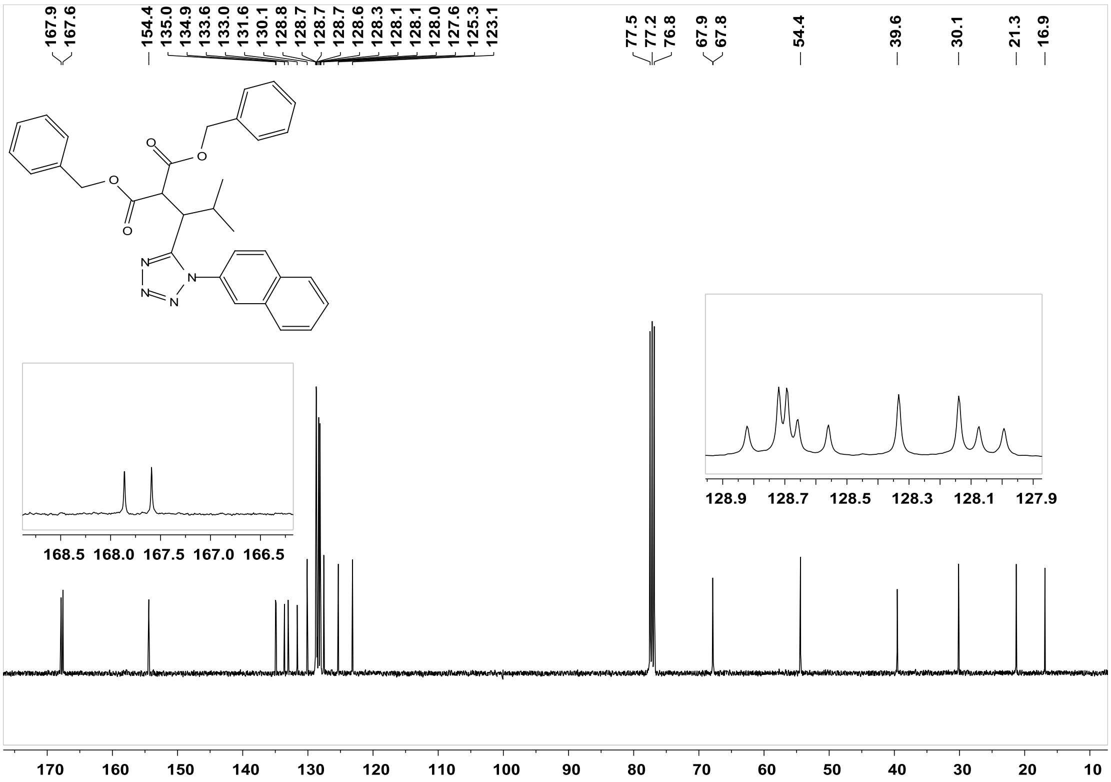

Supplementary Figure 46. ^{13}C NMR spectra for product **4j**

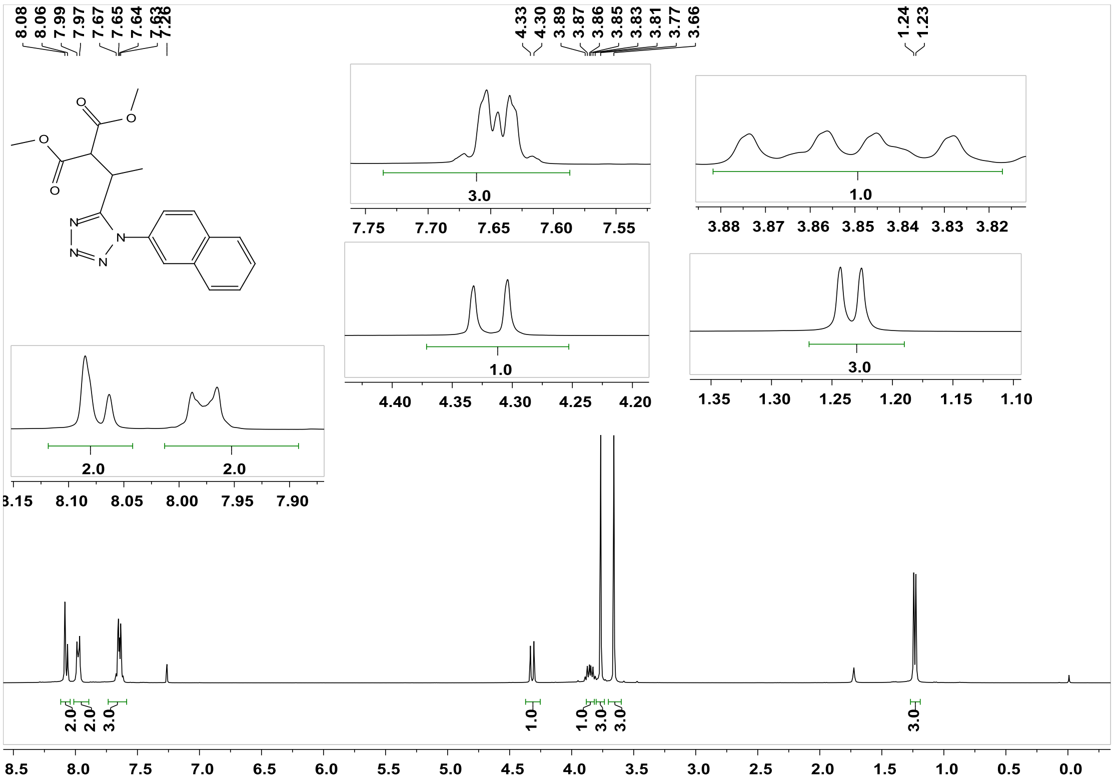

Supplementary Figure 47. ^{1}H NMR spectra for product **4k**

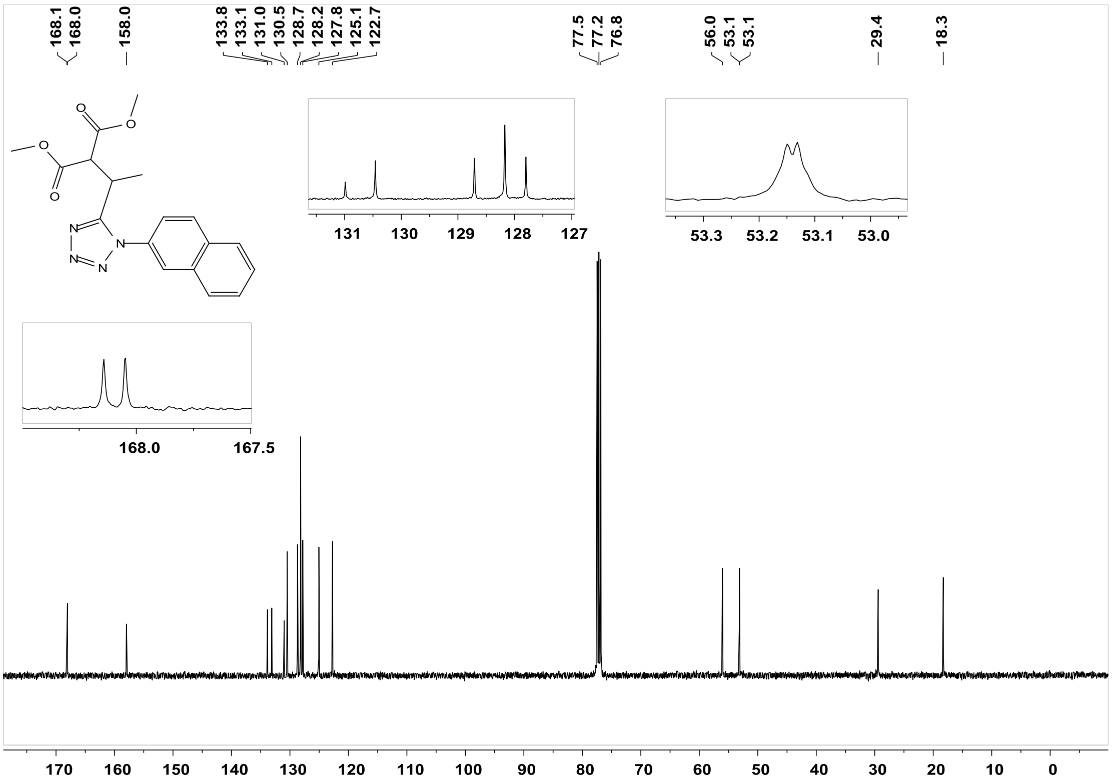

Supplementary Figure 48. ^{13}C NMR spectra for product **4k**

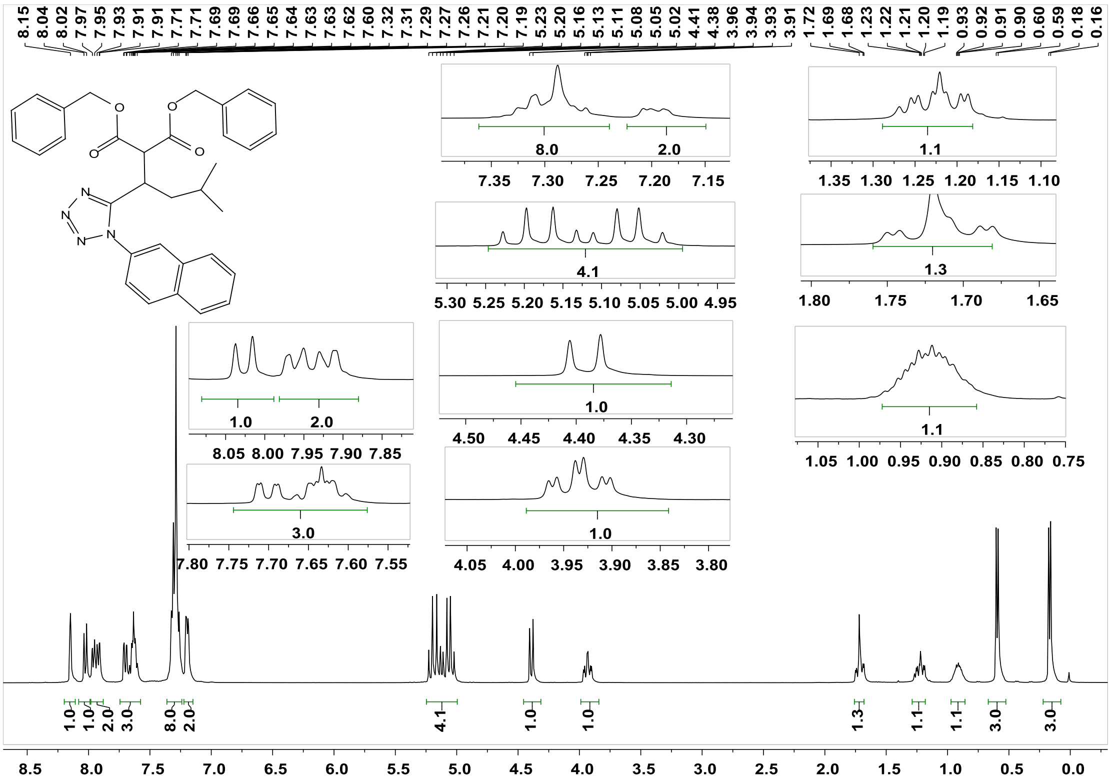

Supplementary Figure 49. ^{1}H NMR spectra for product **4l**

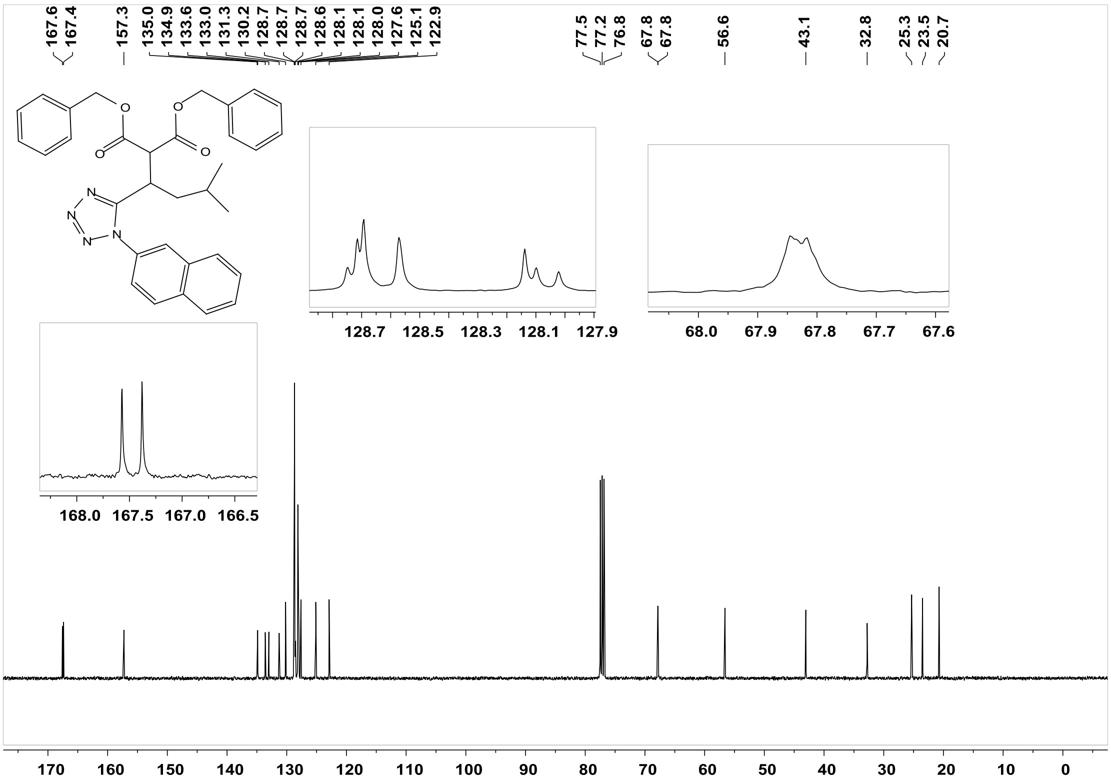

Supplementary Figure 50. ^{13}C NMR spectra for product **4l**

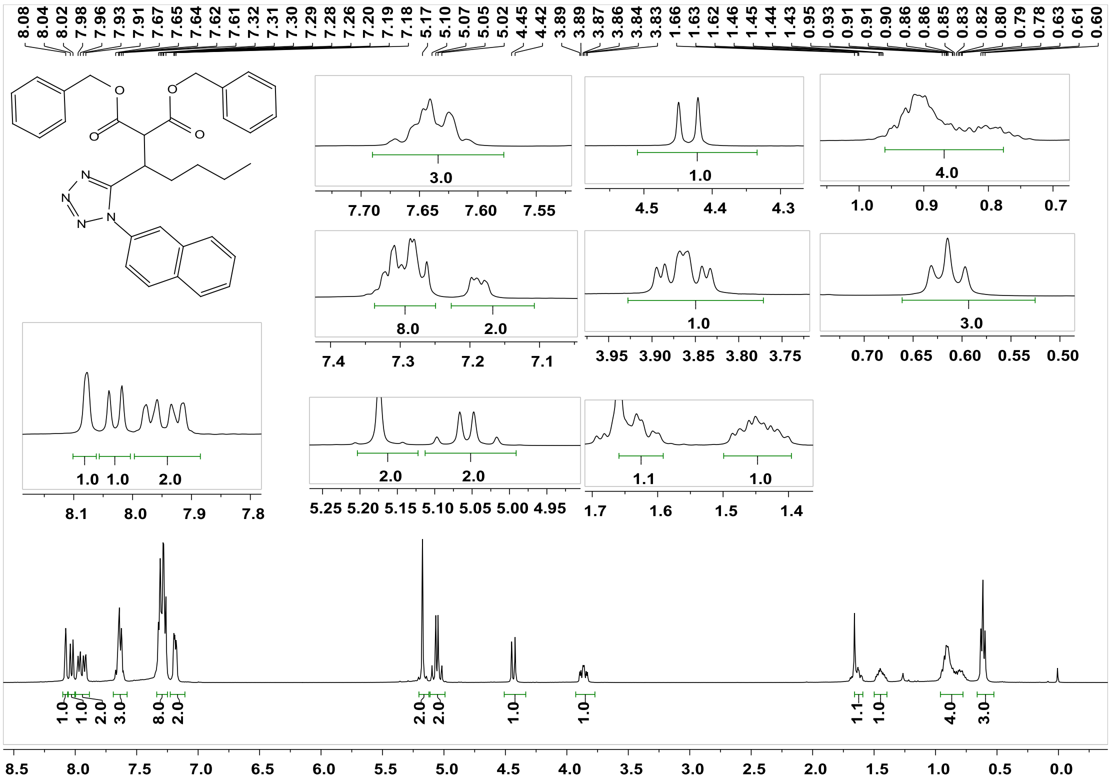

Supplementary Figure 51. ^{1}H NMR spectra for product **4m**

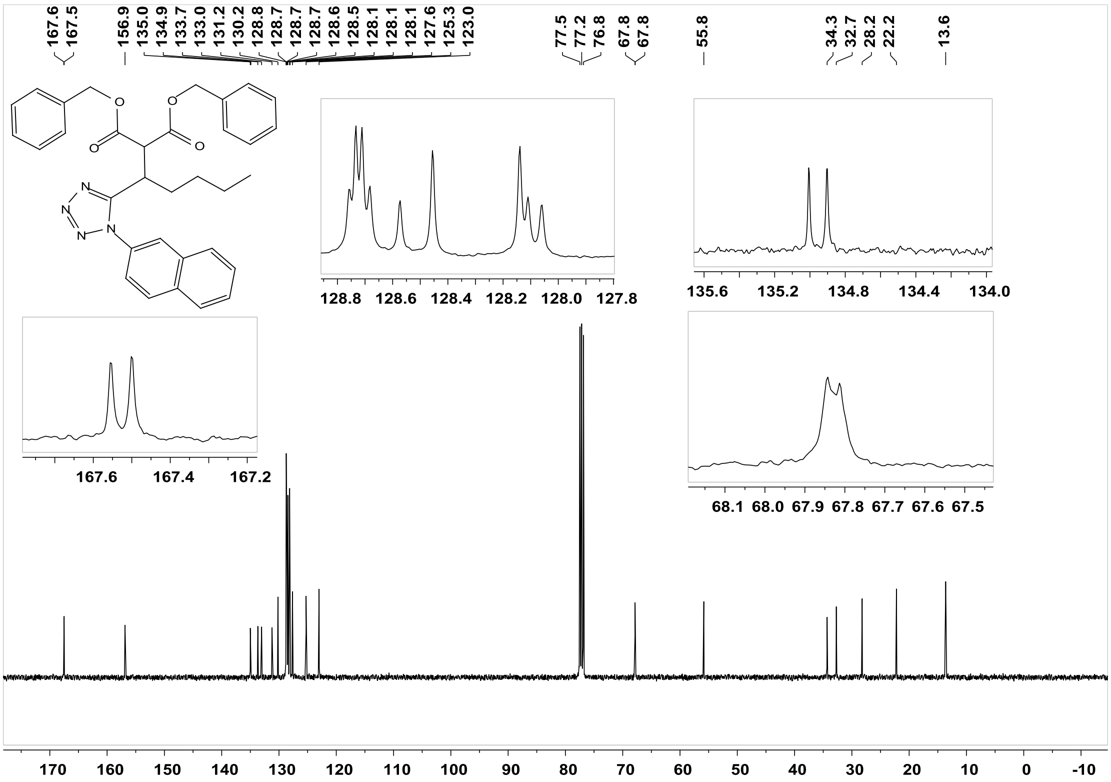

Supplementary Figure 52. ^{13}C NMR spectra for product **4m**

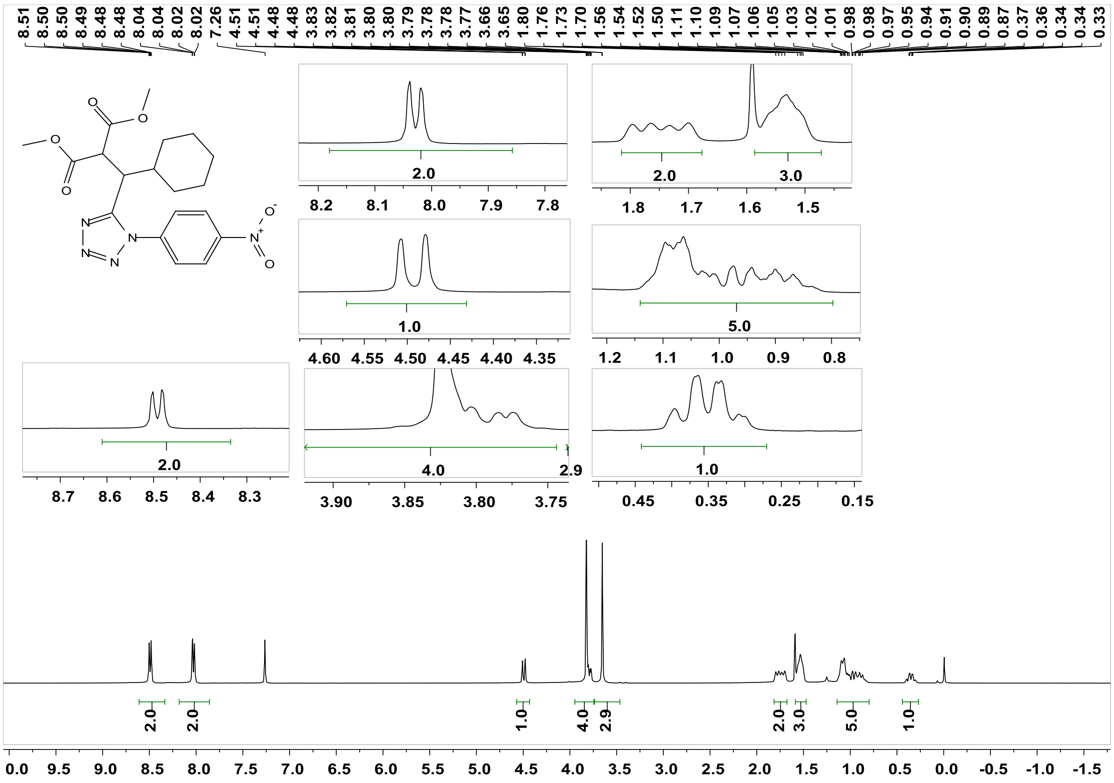

Supplementary Figure 53. ^{1}H NMR spectra for product **4n**

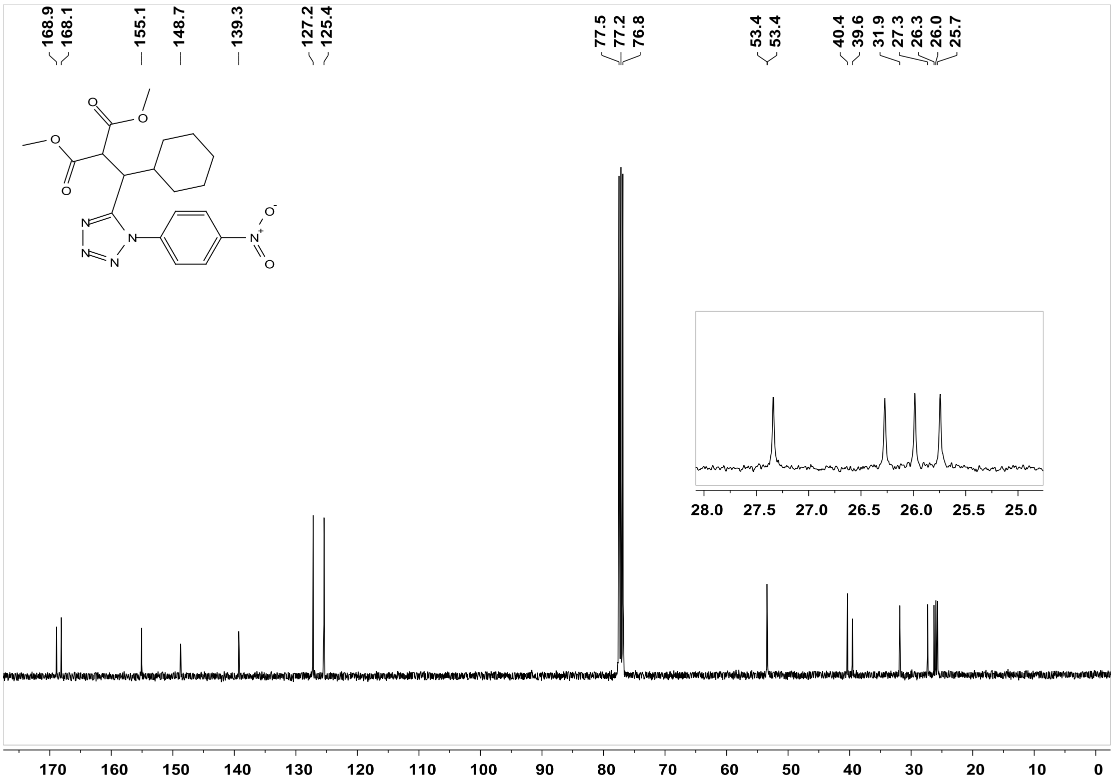

Supplementary Figure 54. ^{13}C NMR spectra for product **4n**

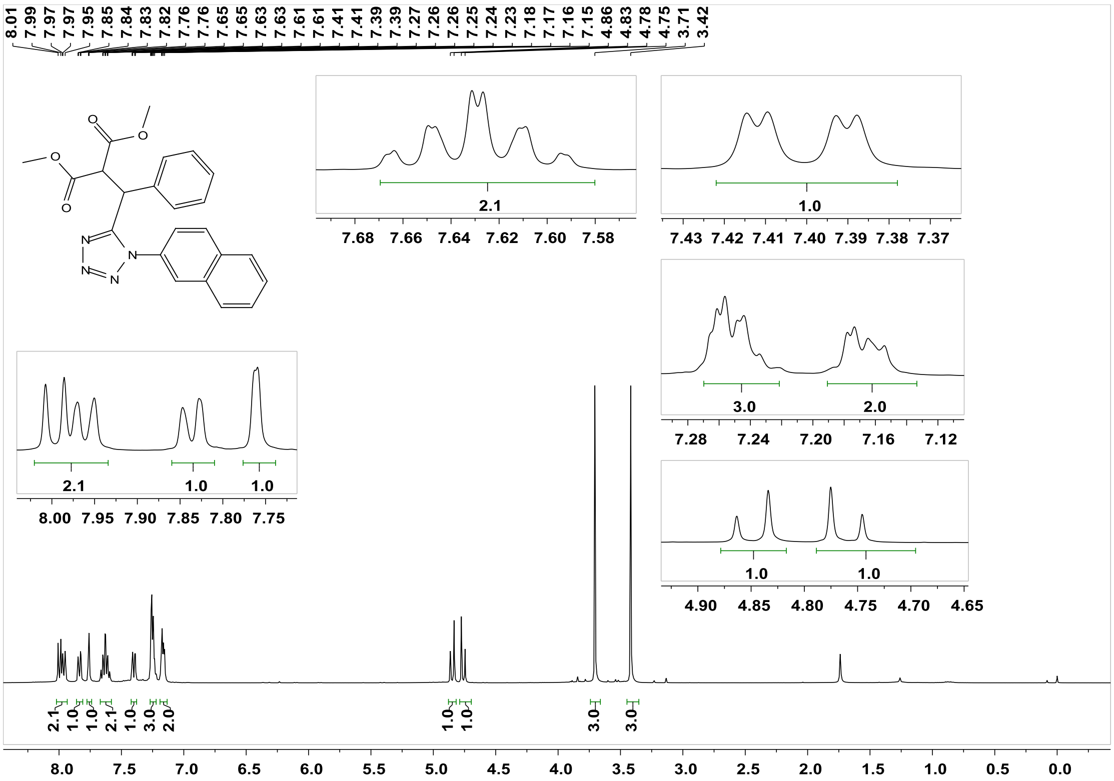

Supplementary Figure 55. ^{1}H NMR spectra for product **4o**

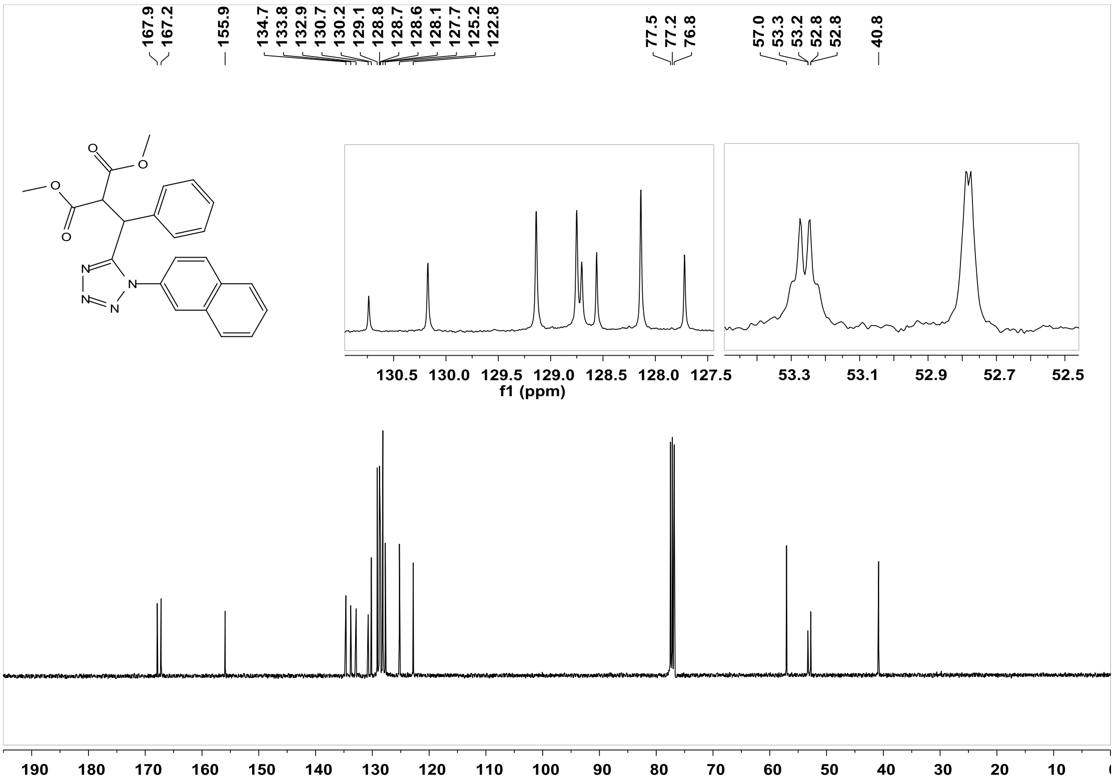

Supplementary Figure 56. ^{13}C NMR spectra for product **4o**

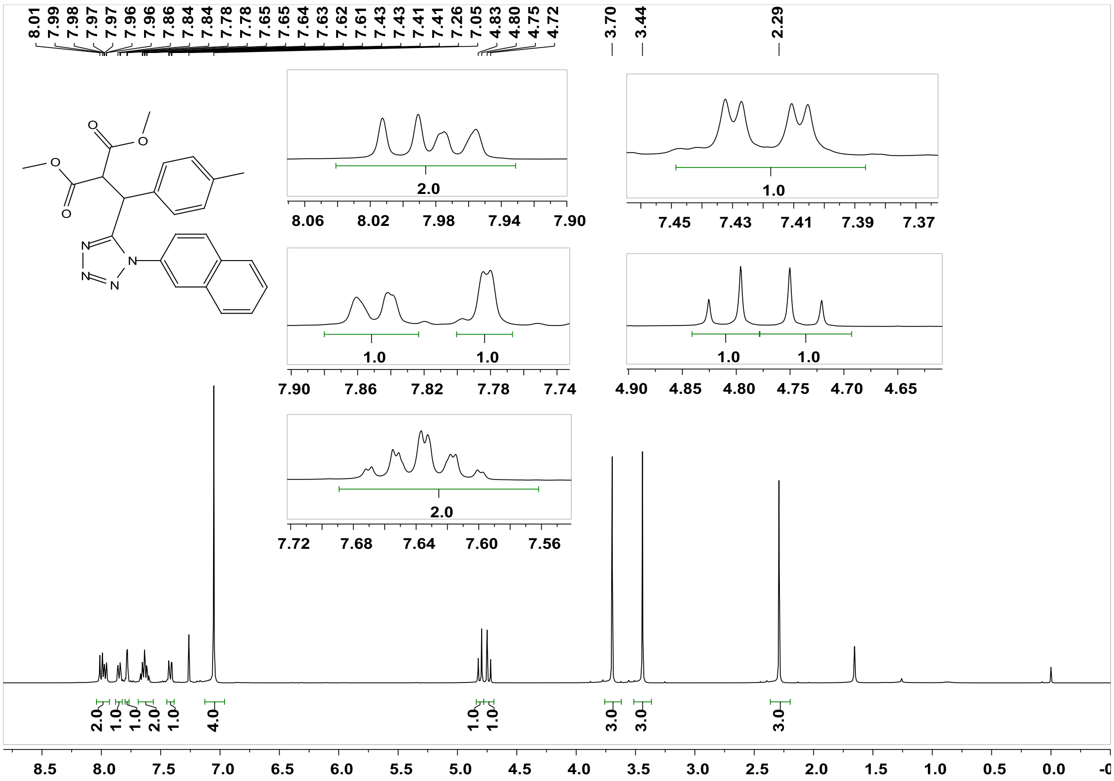

Supplementary Figure 57. ^{1}H NMR spectra for product **4p**

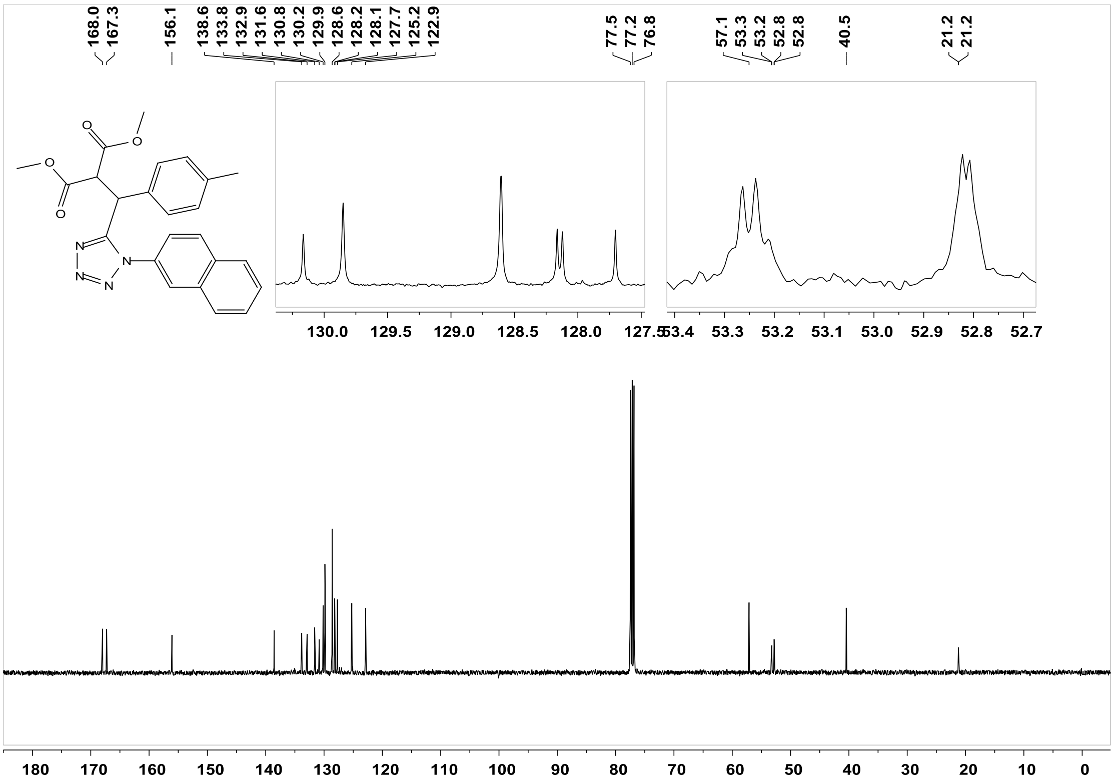

Supplementary Figure 58. ^{13}C NMR spectra for product **4p**

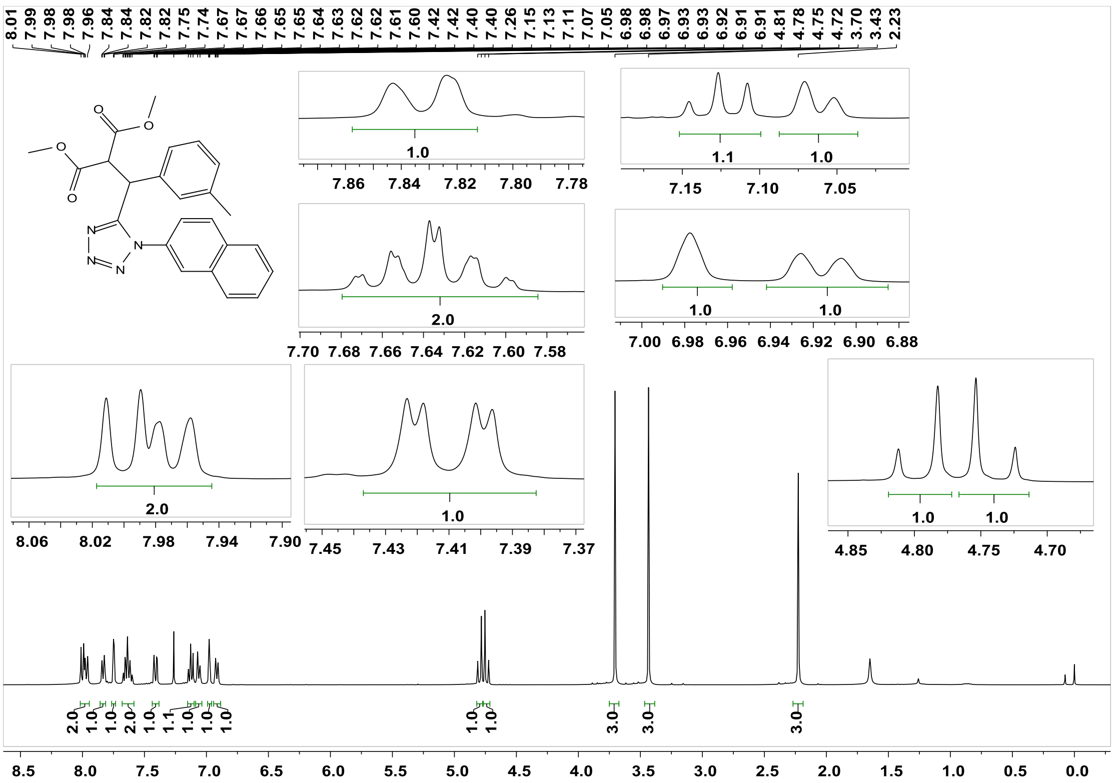

Supplementary Figure 59. ^{1}H NMR spectra for product **4q**

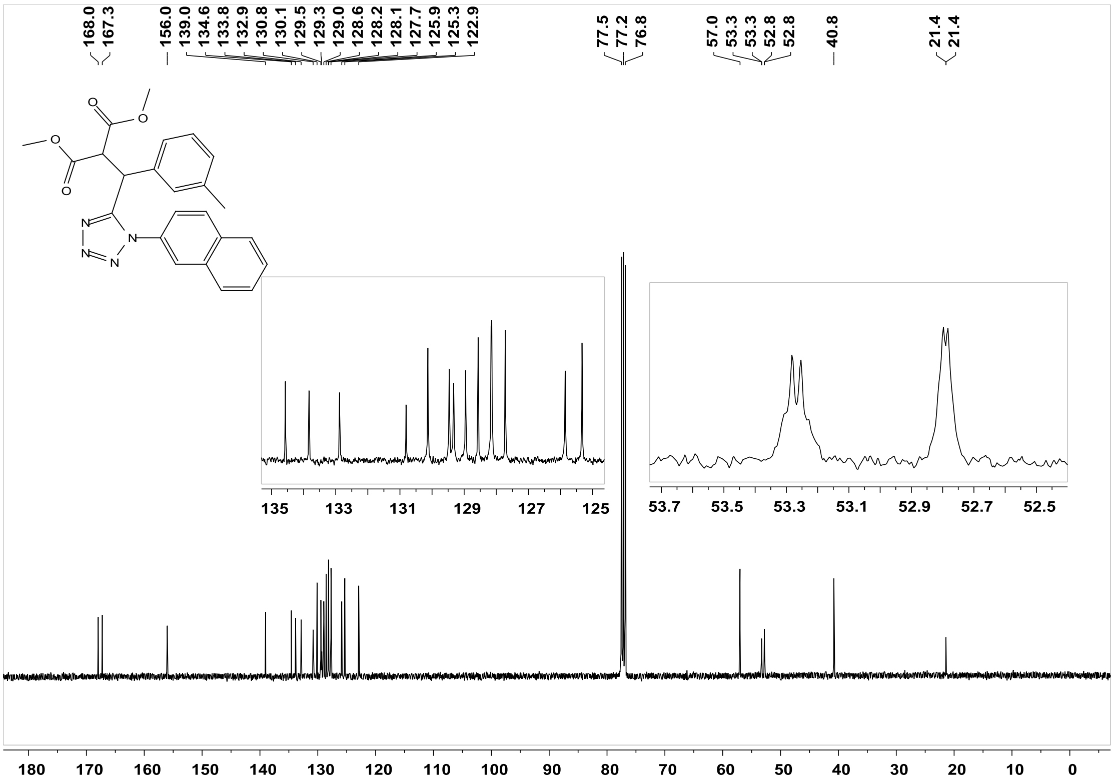

Supplementary Figure 60. ^{13}C NMR spectra for product **4q**

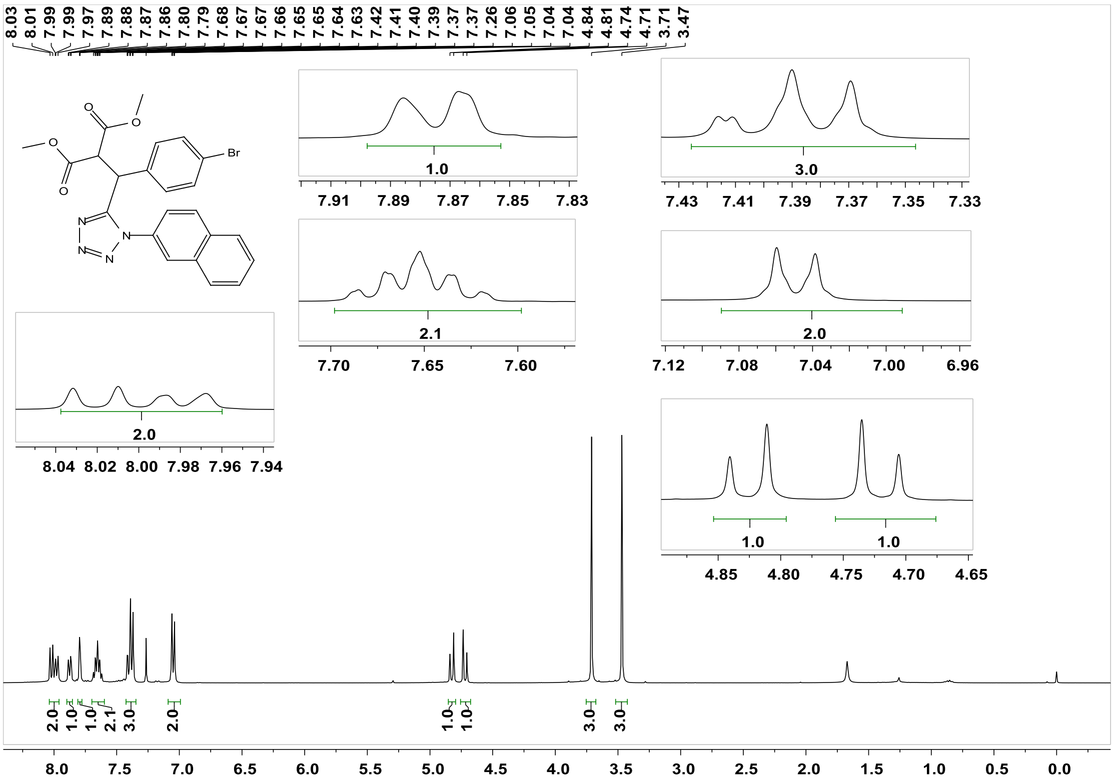

Supplementary Figure 61. ^{1}H NMR spectra for product **4r**

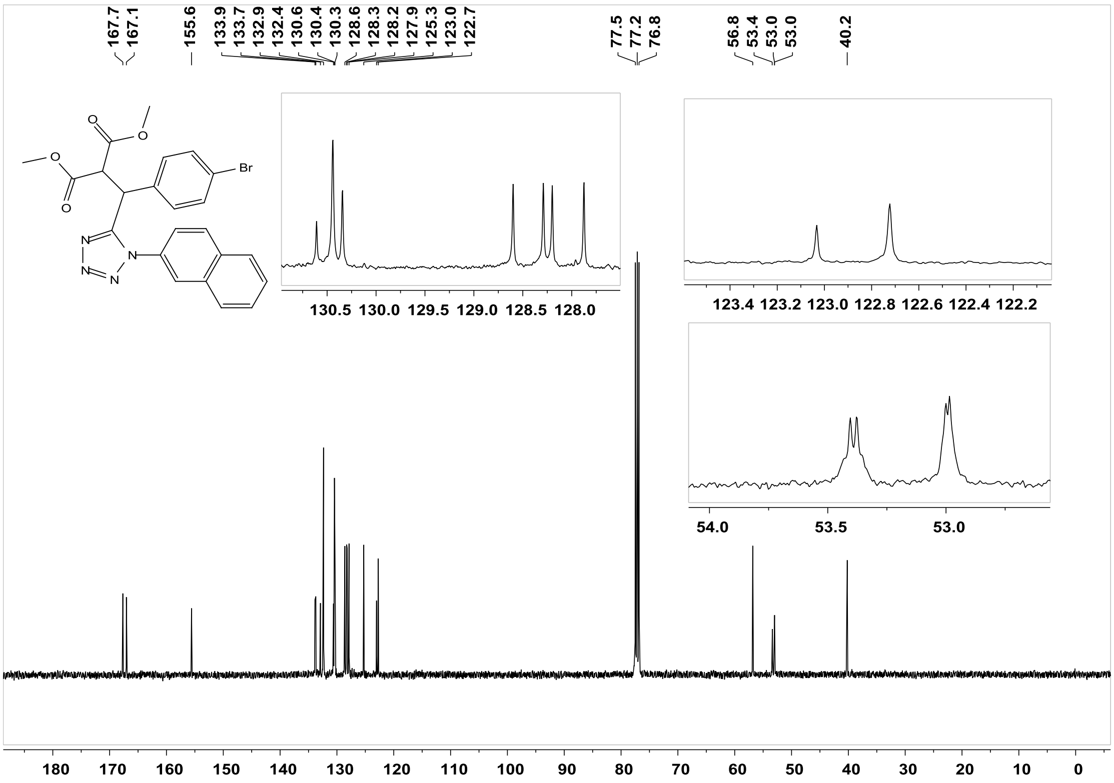

Supplementary Figure 62. ^{13}C NMR spectra for product **4r**

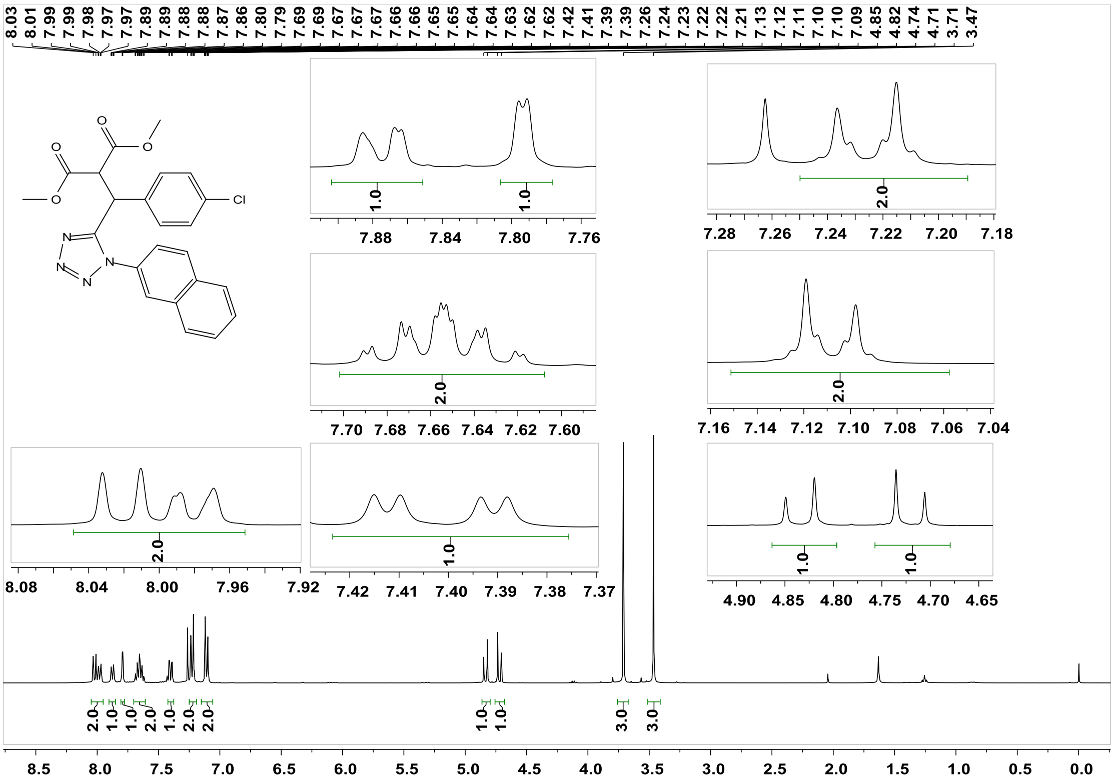

Supplementary Figure 63. ^{1}H NMR spectra for product **4s**

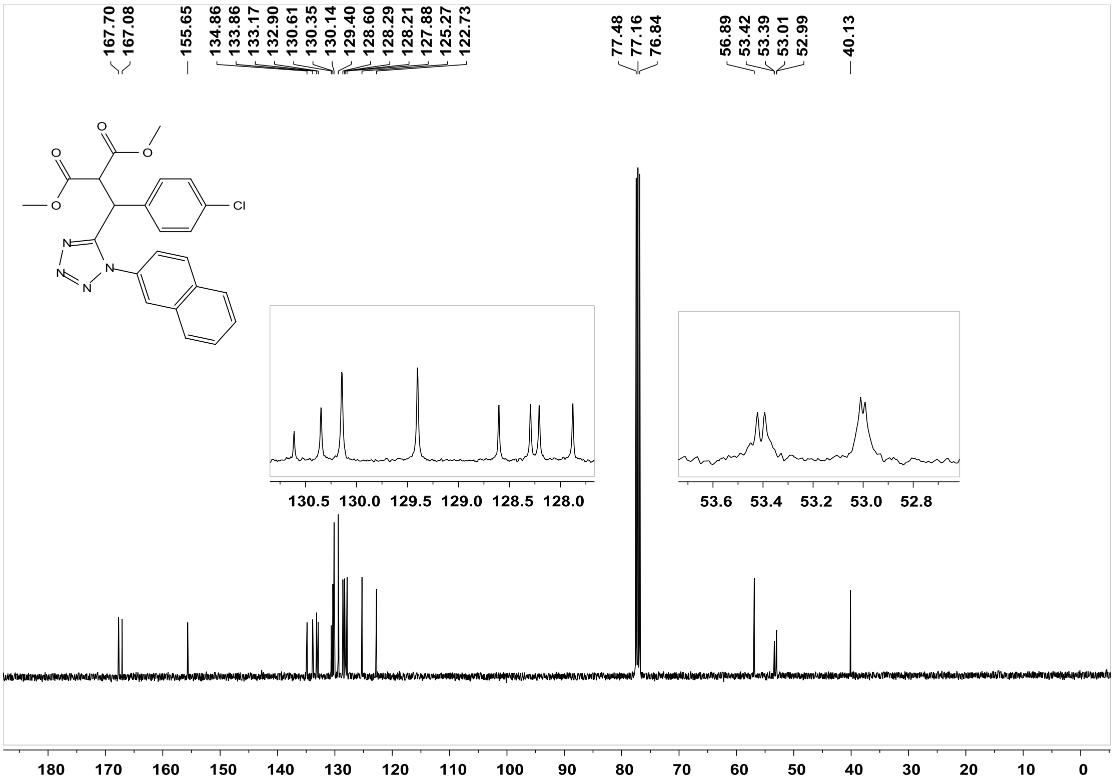

Supplementary Figure 64. ^{13}CNMR spectra for product **4s**

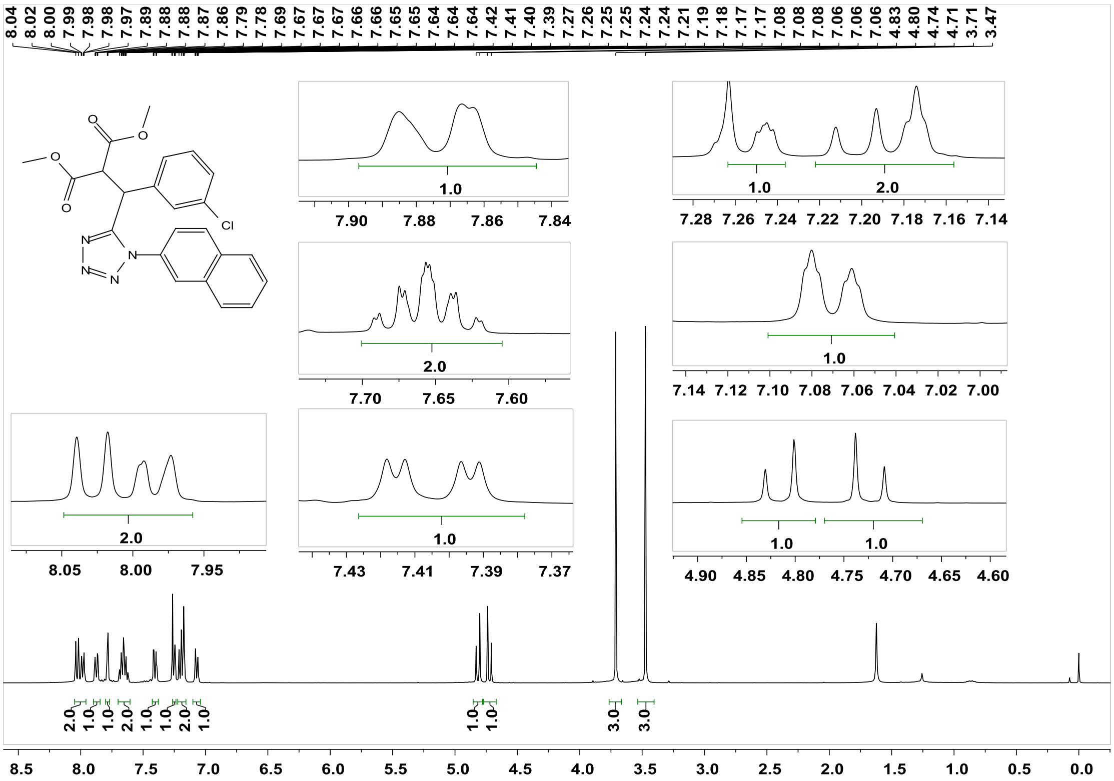

Supplementary Figure 65. ^{1}H NMR spectra for product **4t**

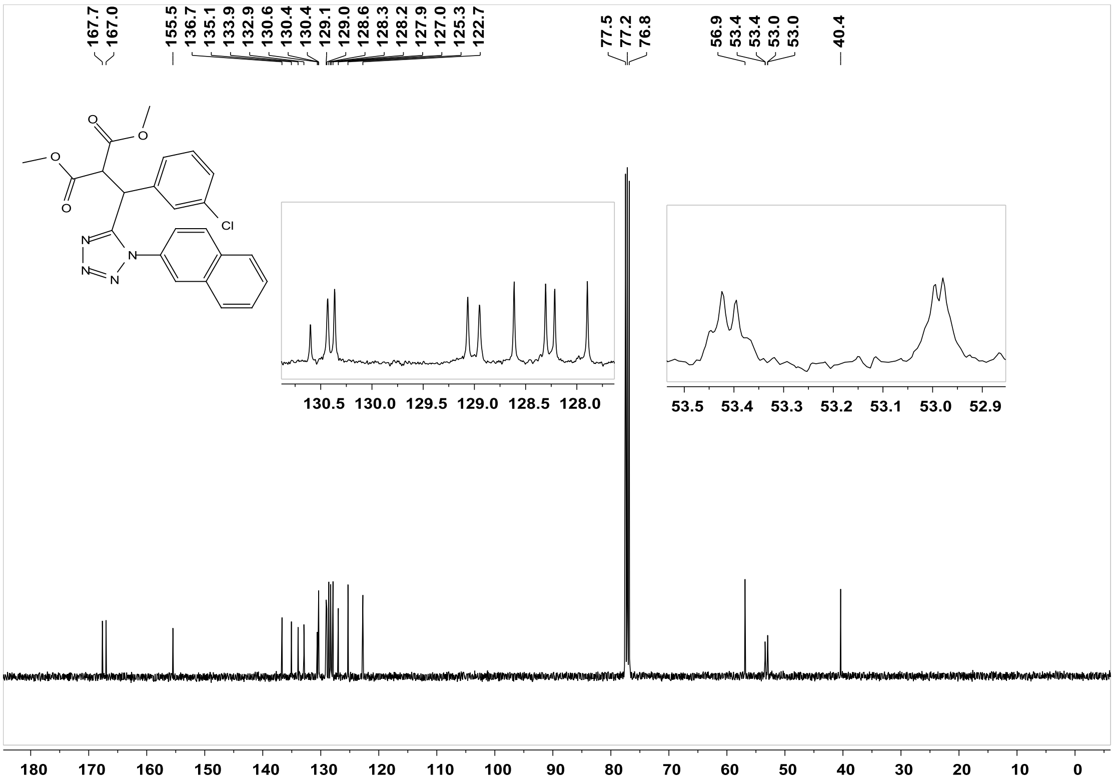

Supplementary Figure 66. ^{13}C NMR spectra for product **4t**

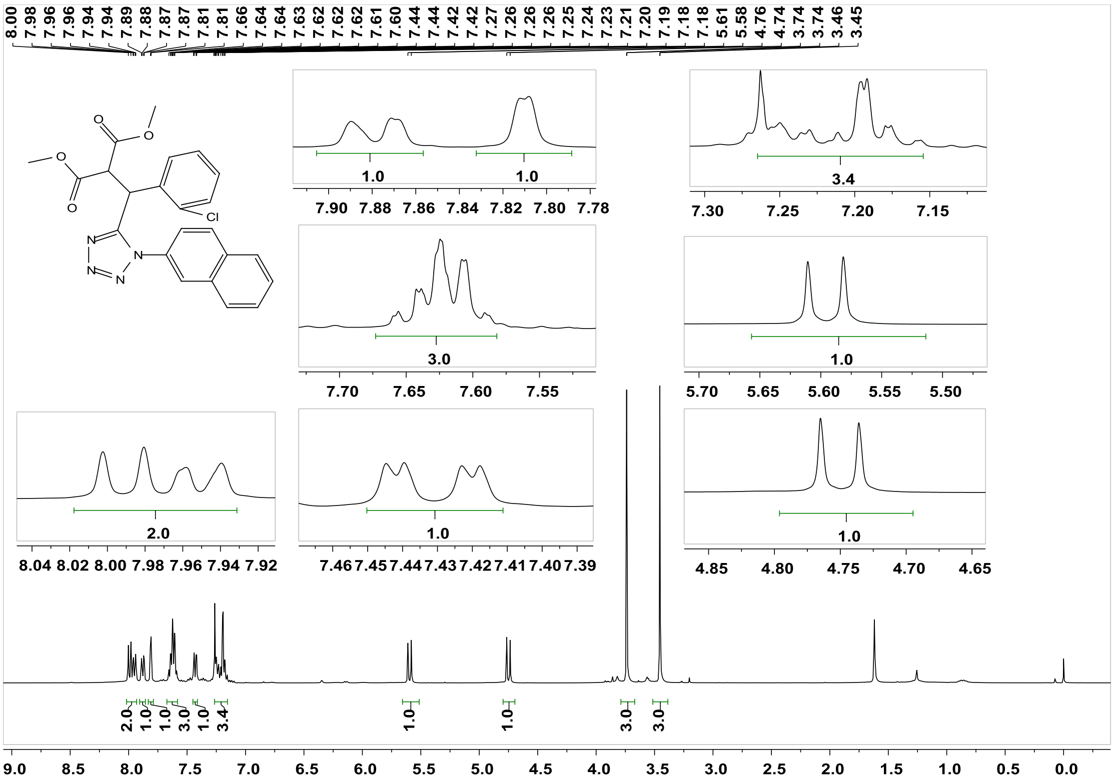

Supplementary Figure 67. ^{1}H NMR spectra for product **4u**

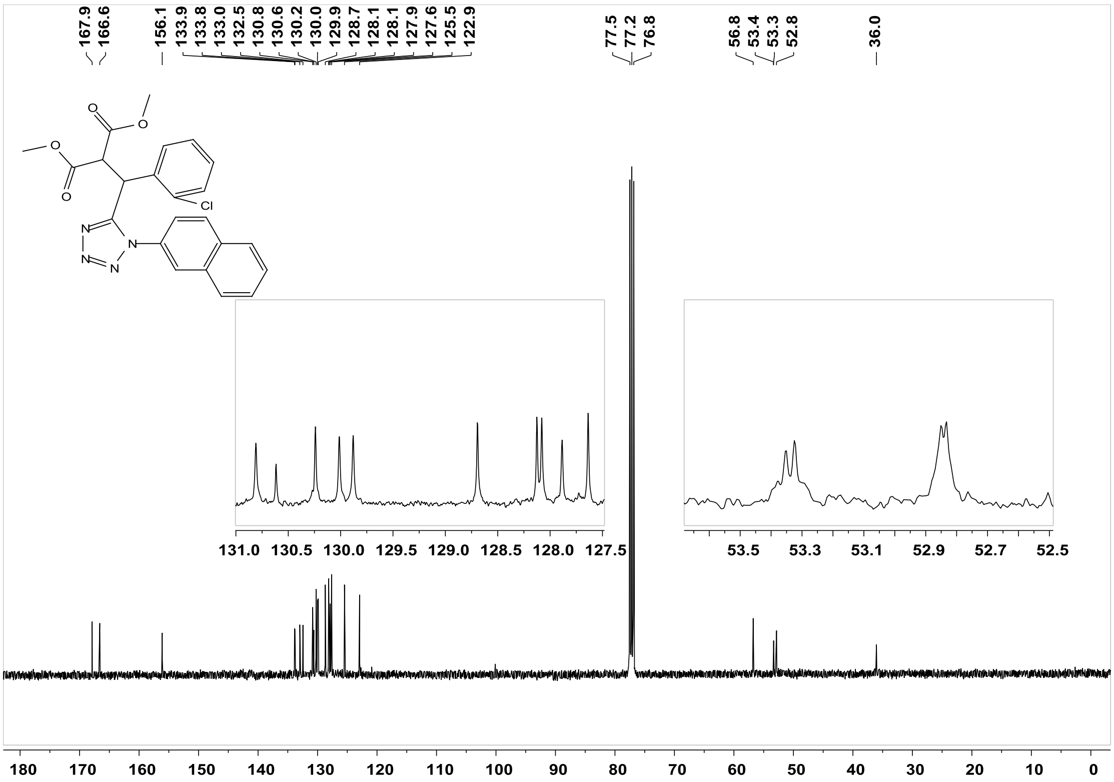

Supplementary Figure 68. ^{13}C NMR spectra for product **4u**

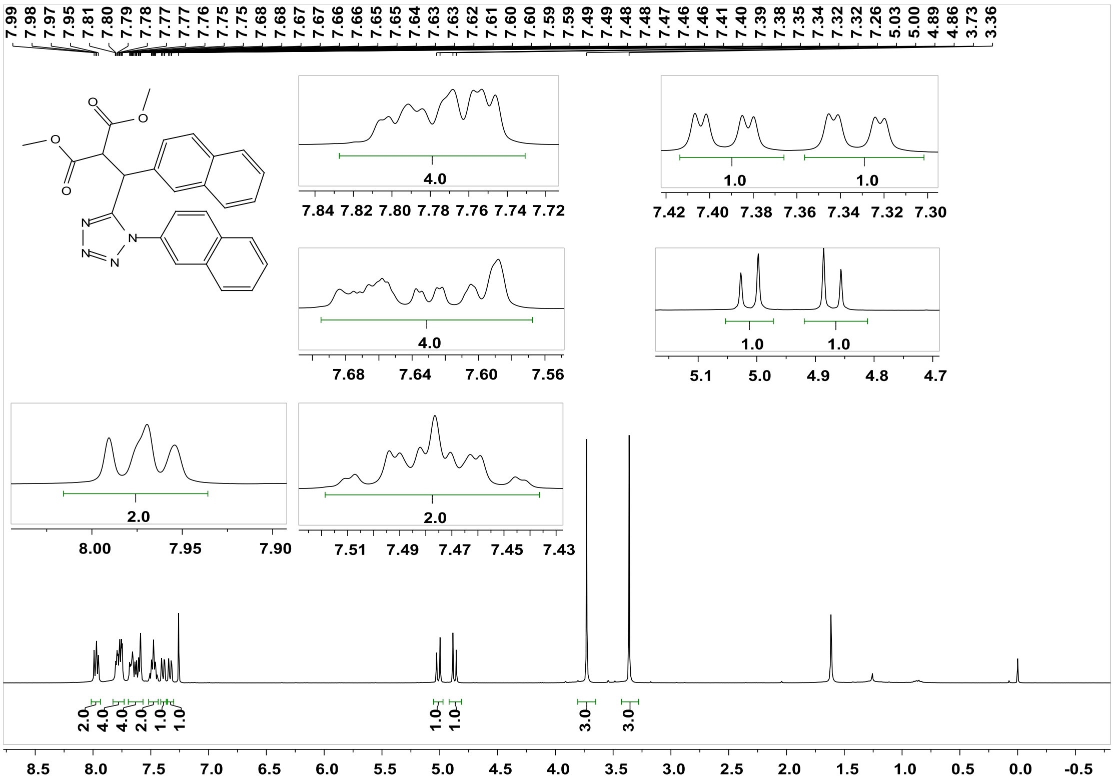

Supplementary Figure 69. ^{1}H NMR spectra for product **4v**

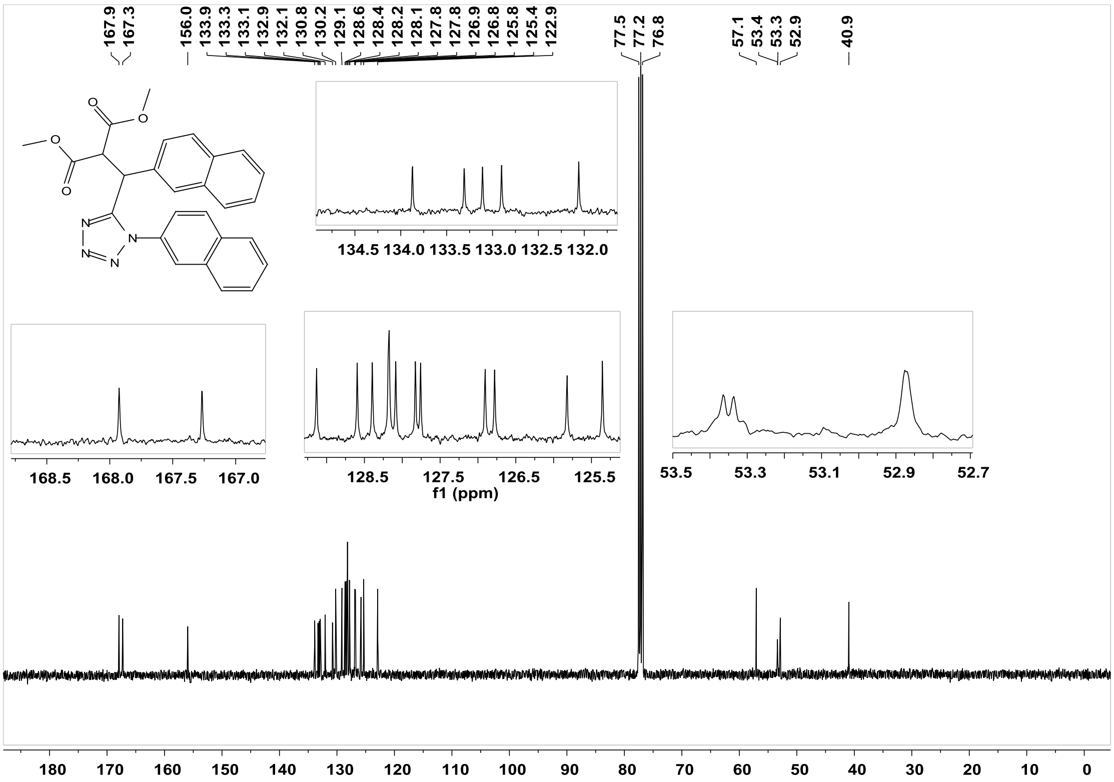

Supplementary Figure 70. ^{13}C NMR spectra for product **4v**

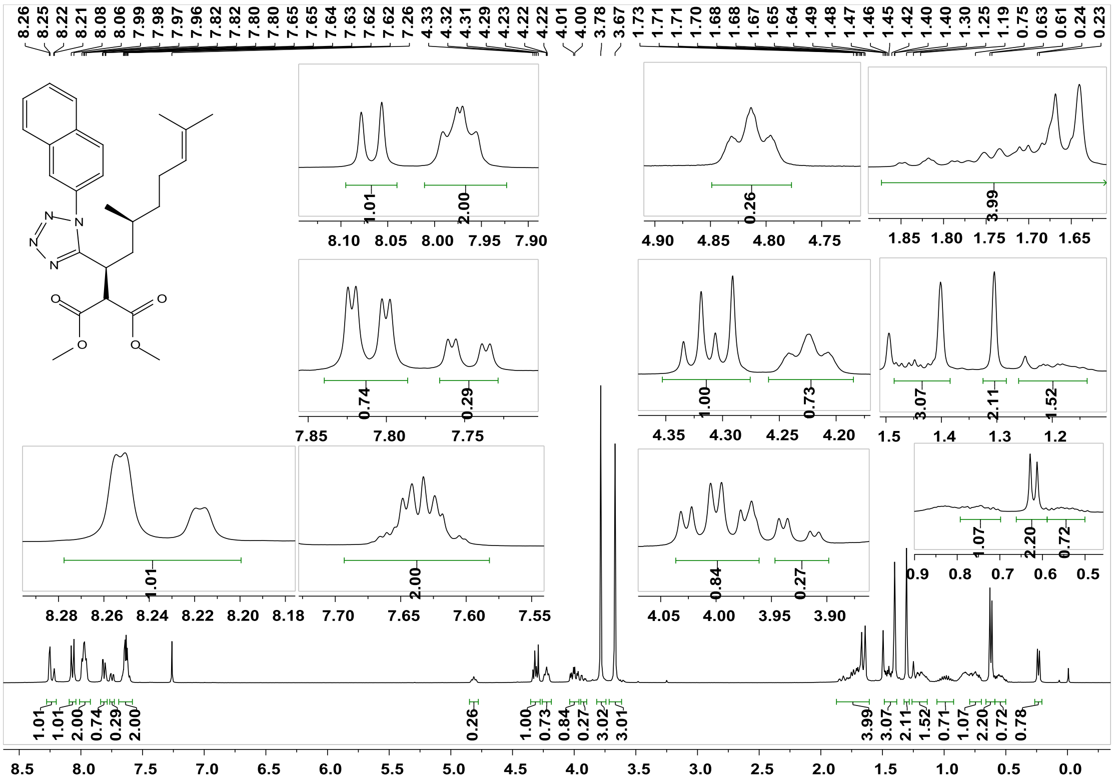

Supplementary Figure 71. ^{1}H NMR spectra for product **4w**

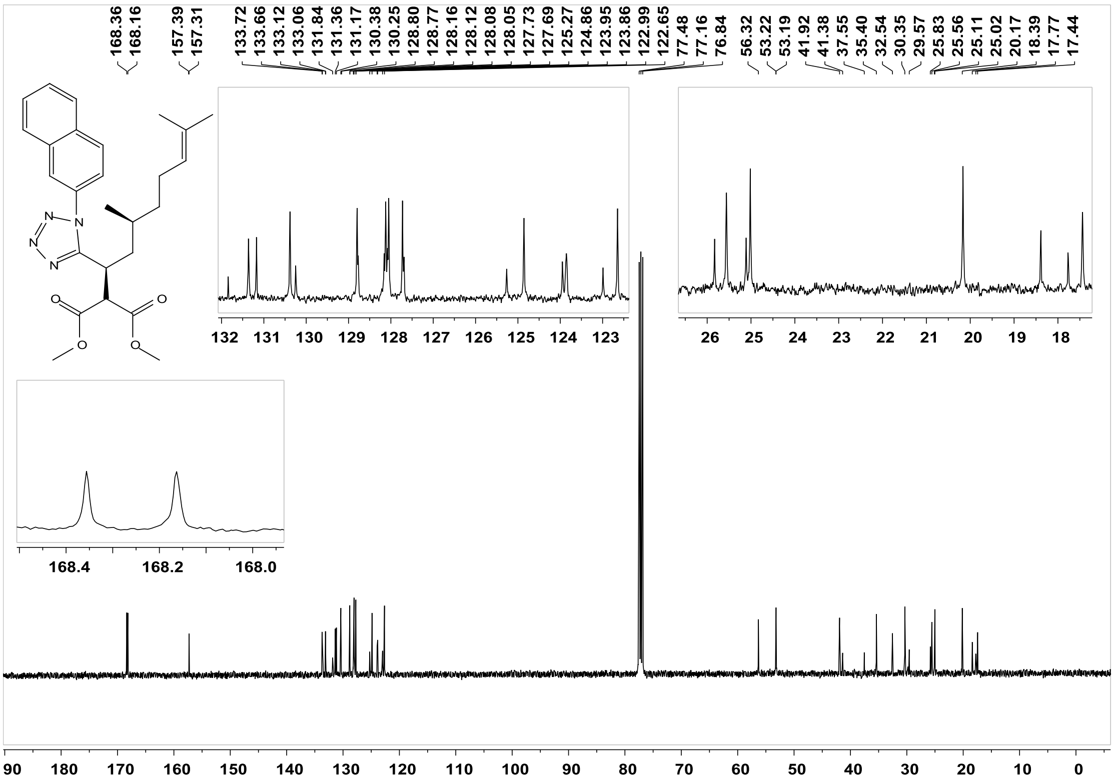

Supplementary Figure 72. ^{13}C NMR spectra for product **4w**

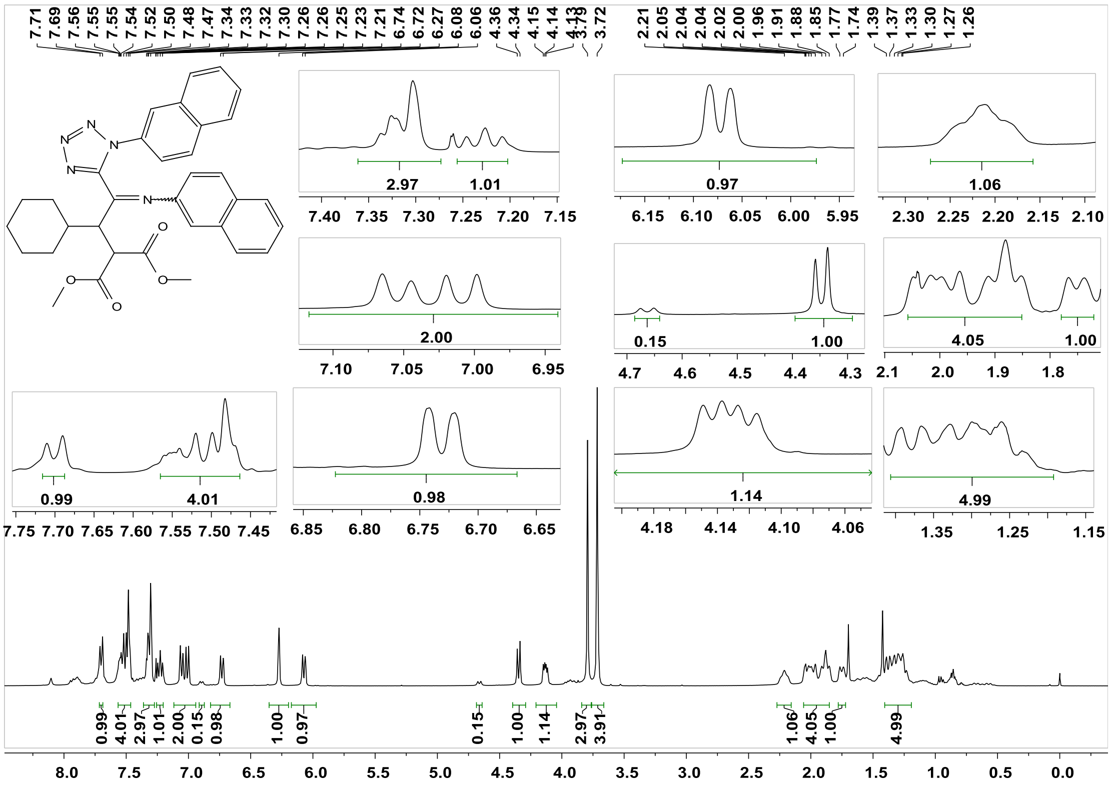

Supplementary Figure 73. ^{1}H NMR spectra for product **5a**

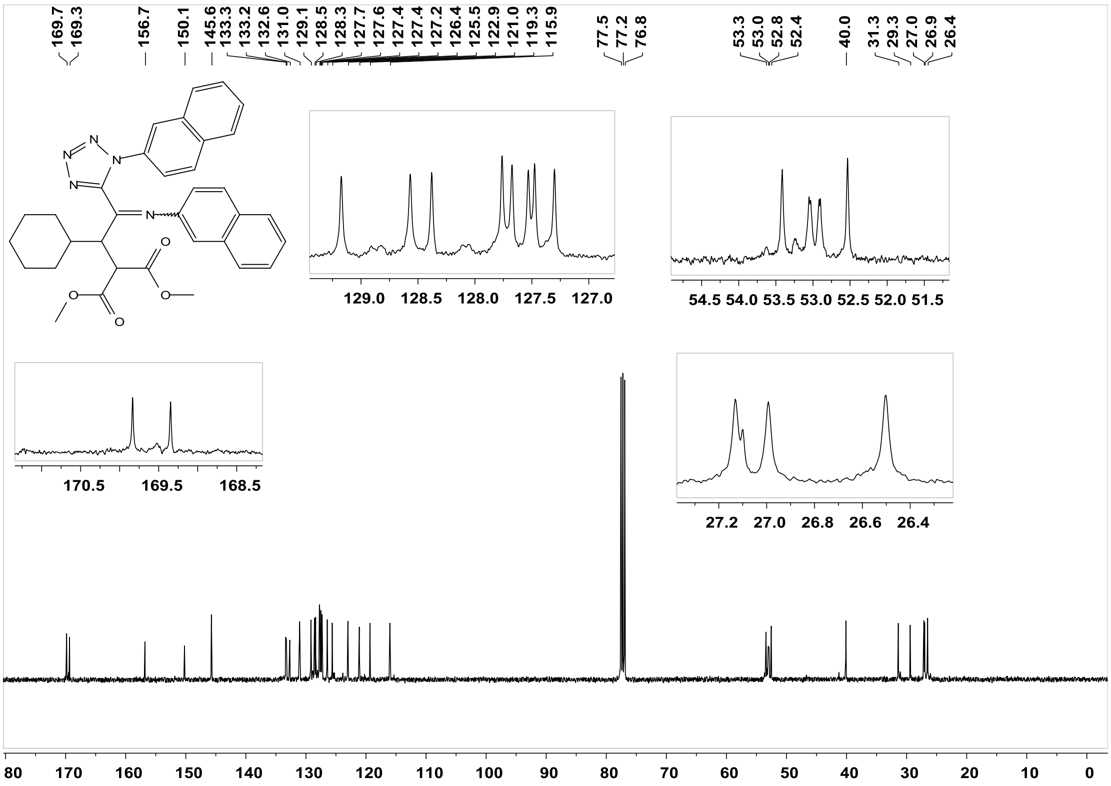

Supplementary Figure 74. ^{13}C NMR spectra for product **5a**

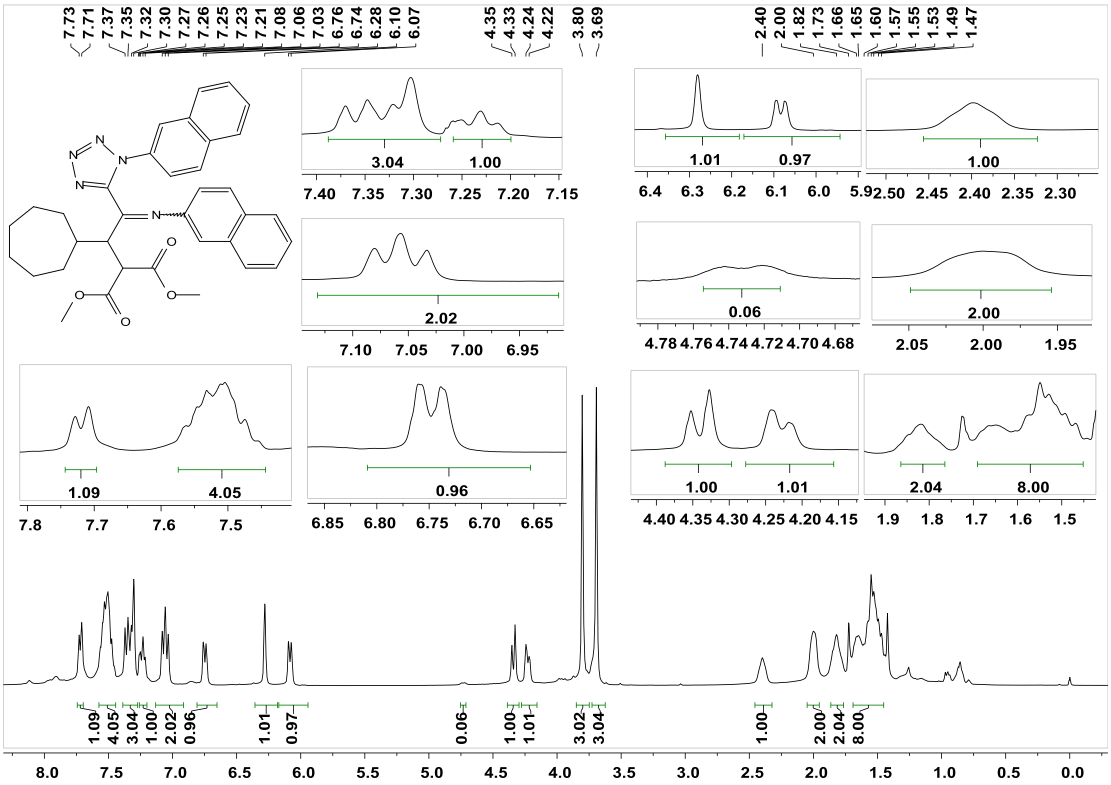

Supplementary Figure 75. ^{1}H NMR spectra for product **5b**

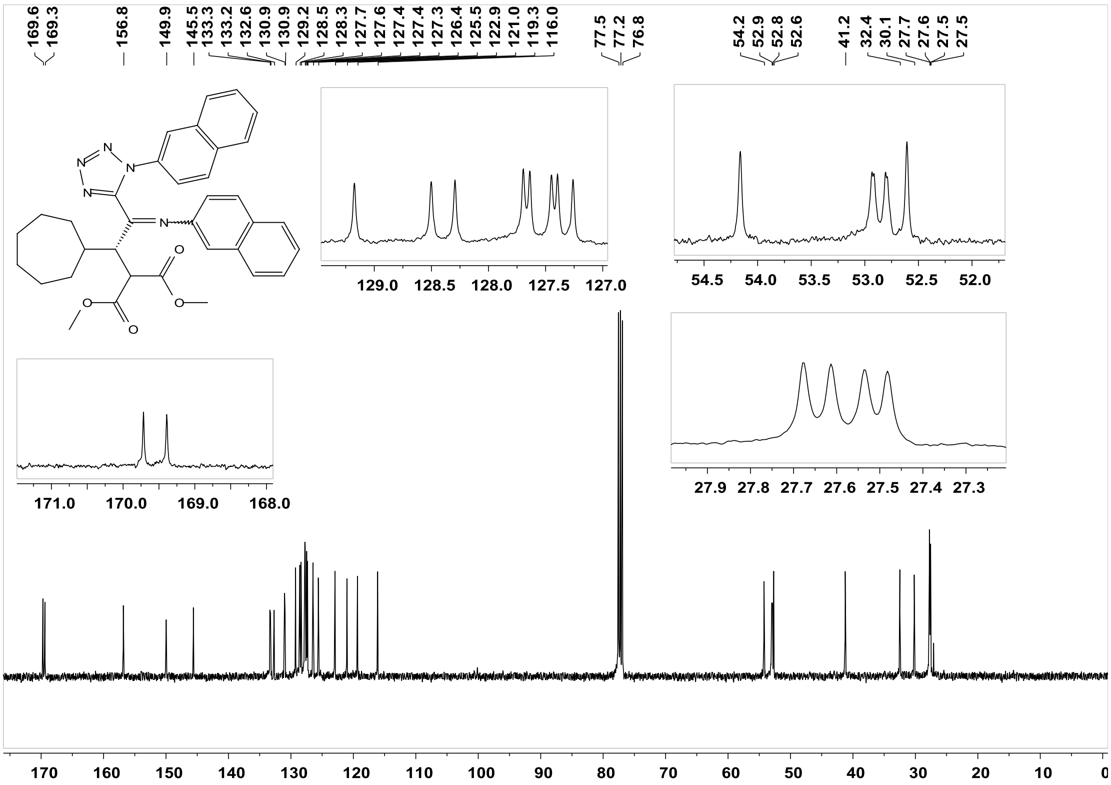

Supplementary Figure 76. ^{13}C NMR spectra for product **5b**

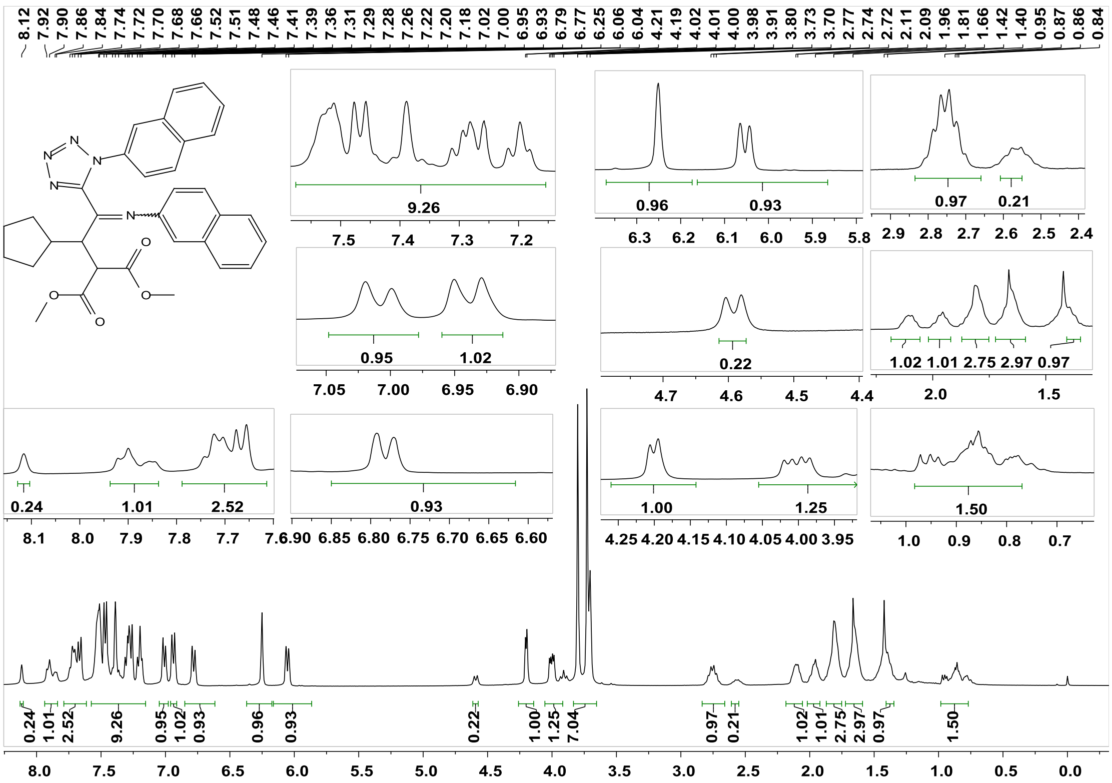

Supplementary Figure 77. ^{1}H NMR spectra for product **5c**

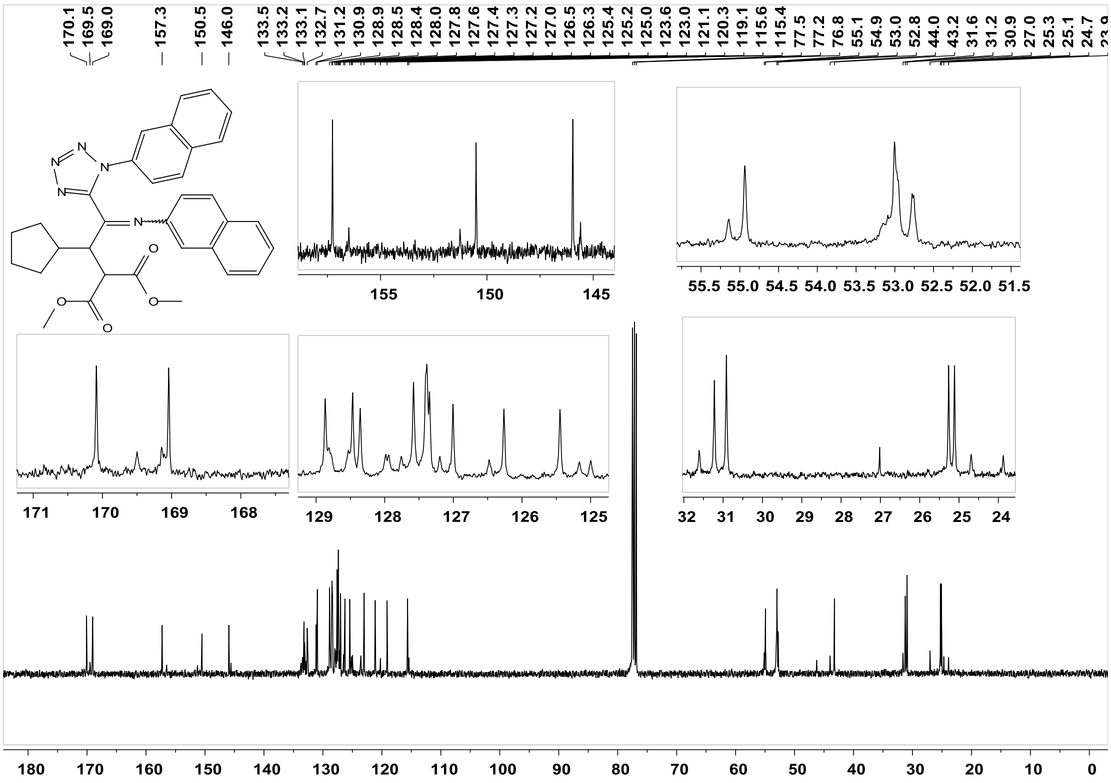

Supplementary Figure 78. ^{13}C NMR spectra for product **5c**

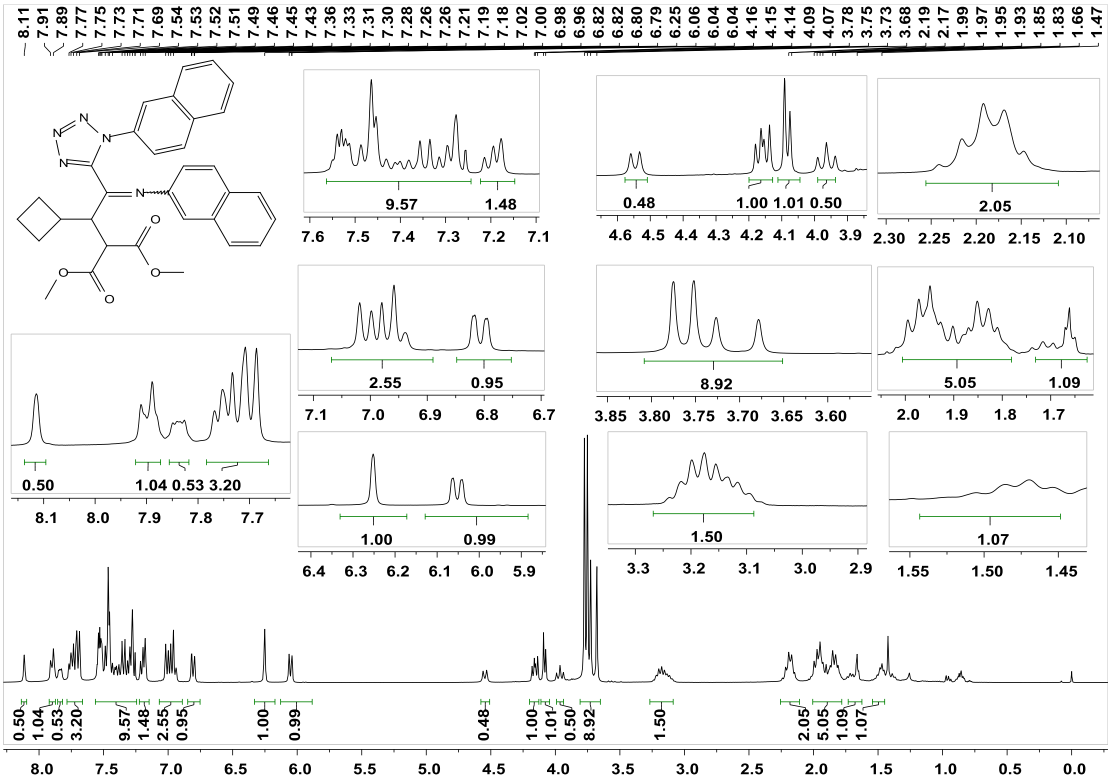

Supplementary Figure 79. ^{1}H NMR spectra for product **5d**

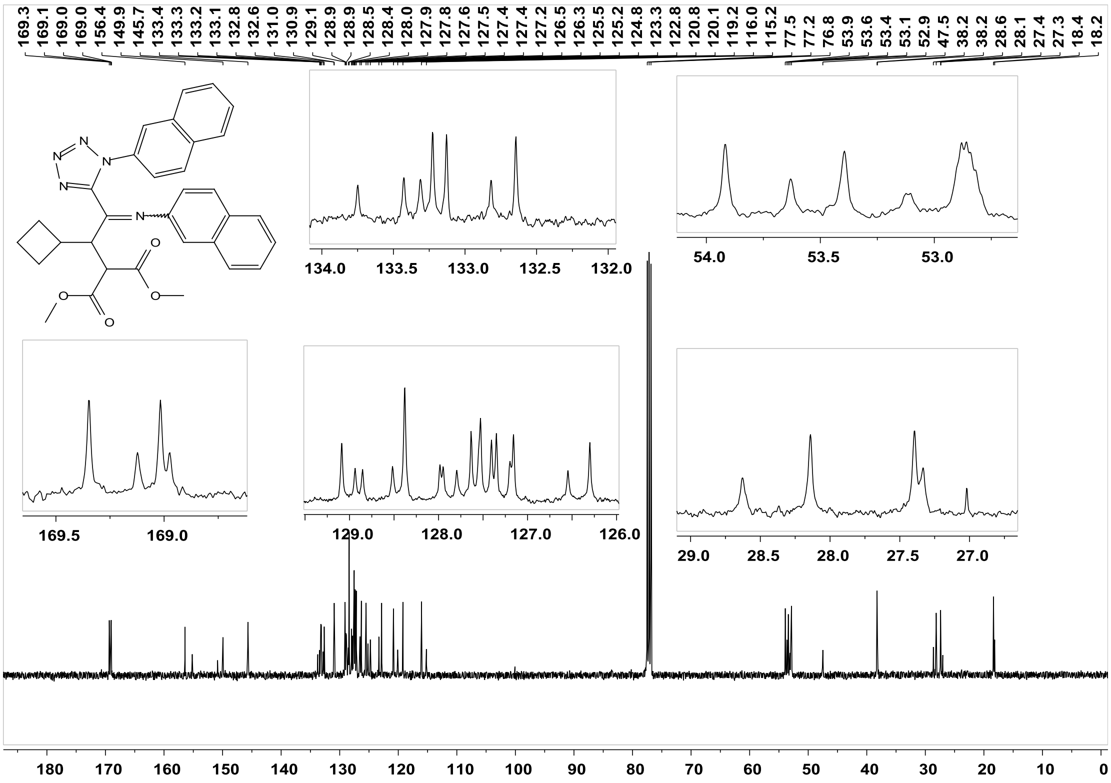

Supplementary Figure 80. ^{13}C NMR spectra for product **5d**

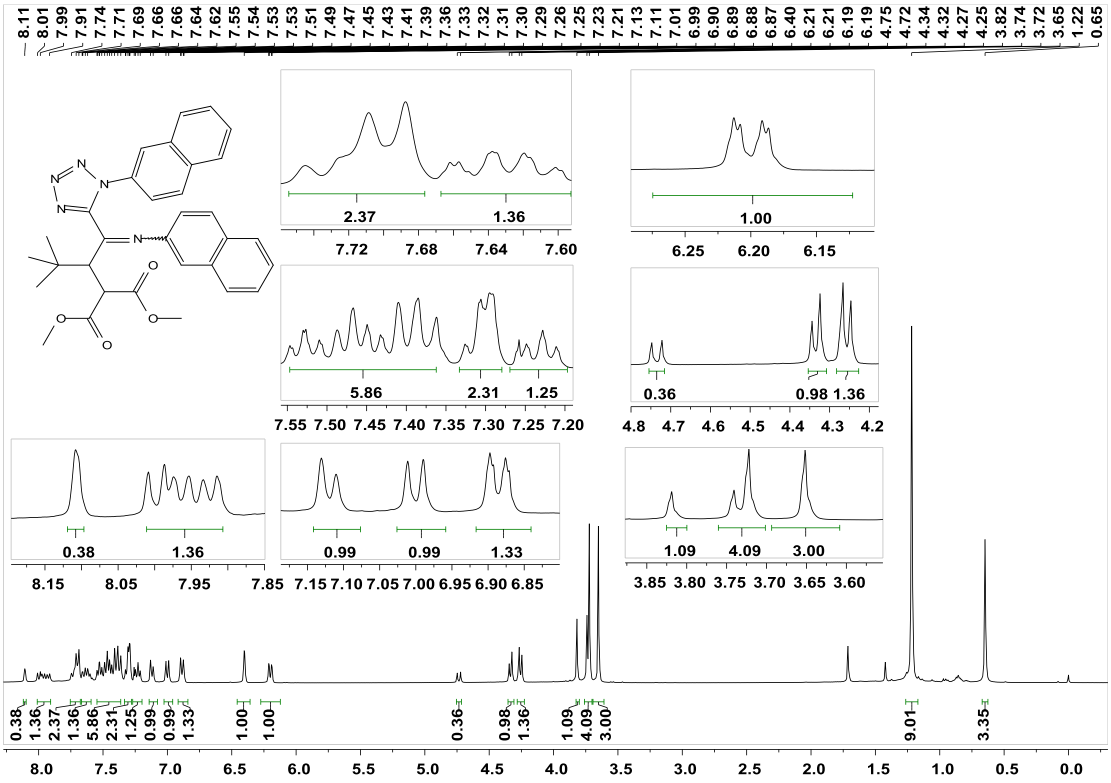

Supplementary Figure 81. ^{1}H NMR spectra for product **5e**

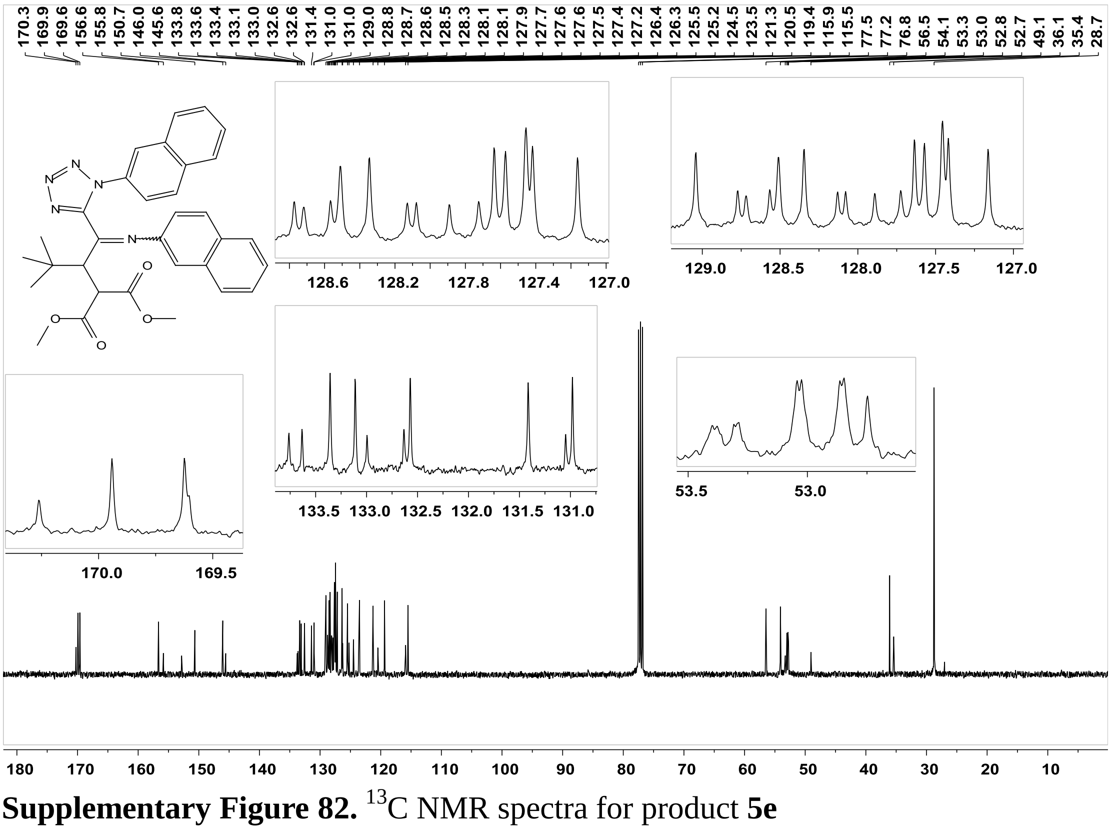

Supplementary Figure 82. ^{13}C NMR spectra for product **5e**

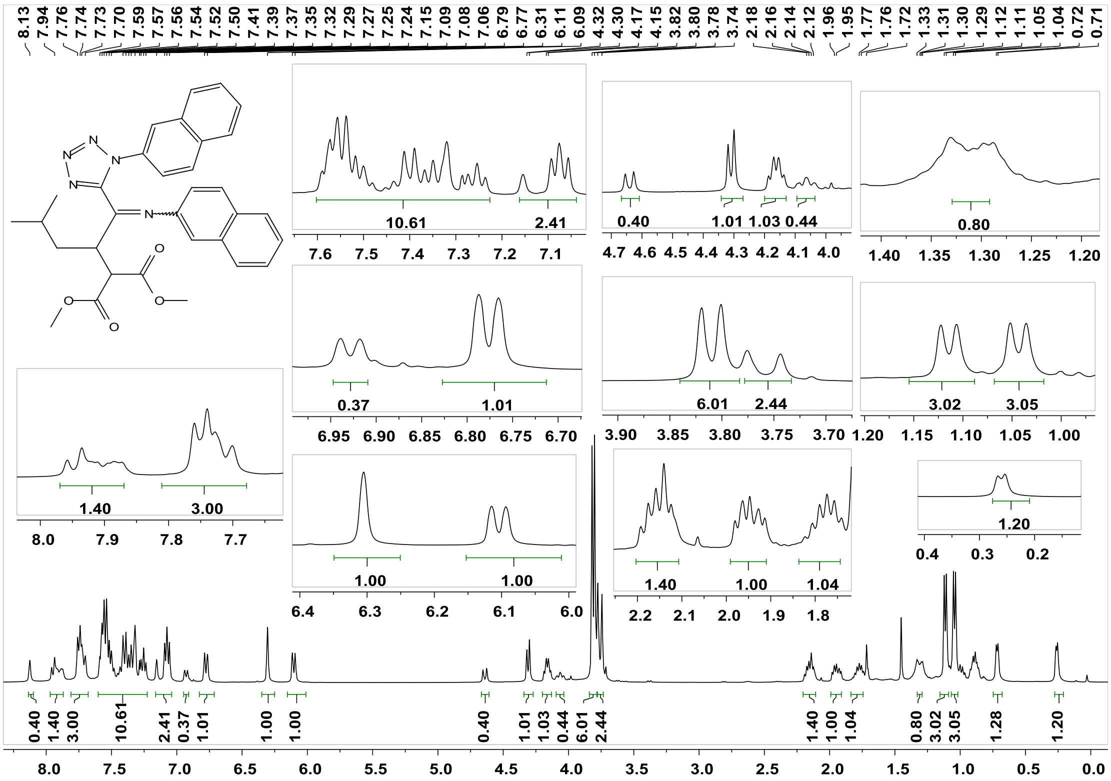

Supplementary Figure 83. ^{1}H NMR spectra for product **5f**

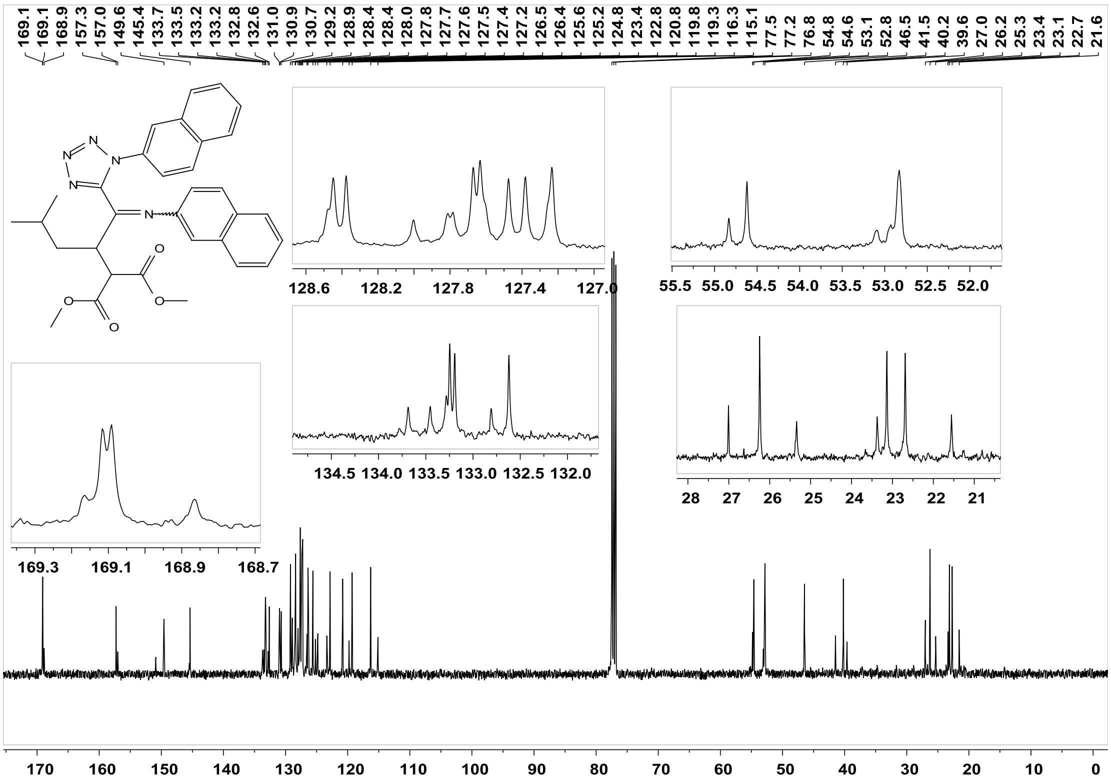

Supplementary Figure 84. ^{13}C NMR spectra for product **5f**

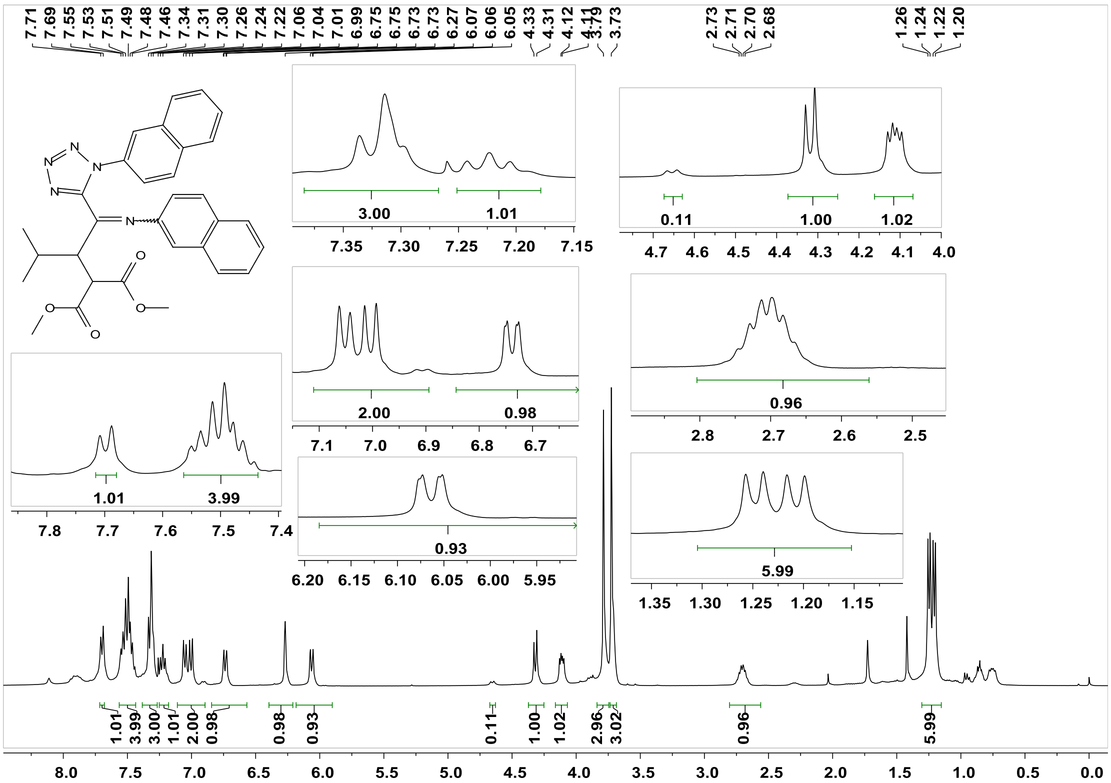

Supplementary Figure 85. ^{1}H NMR spectra for product **5g**

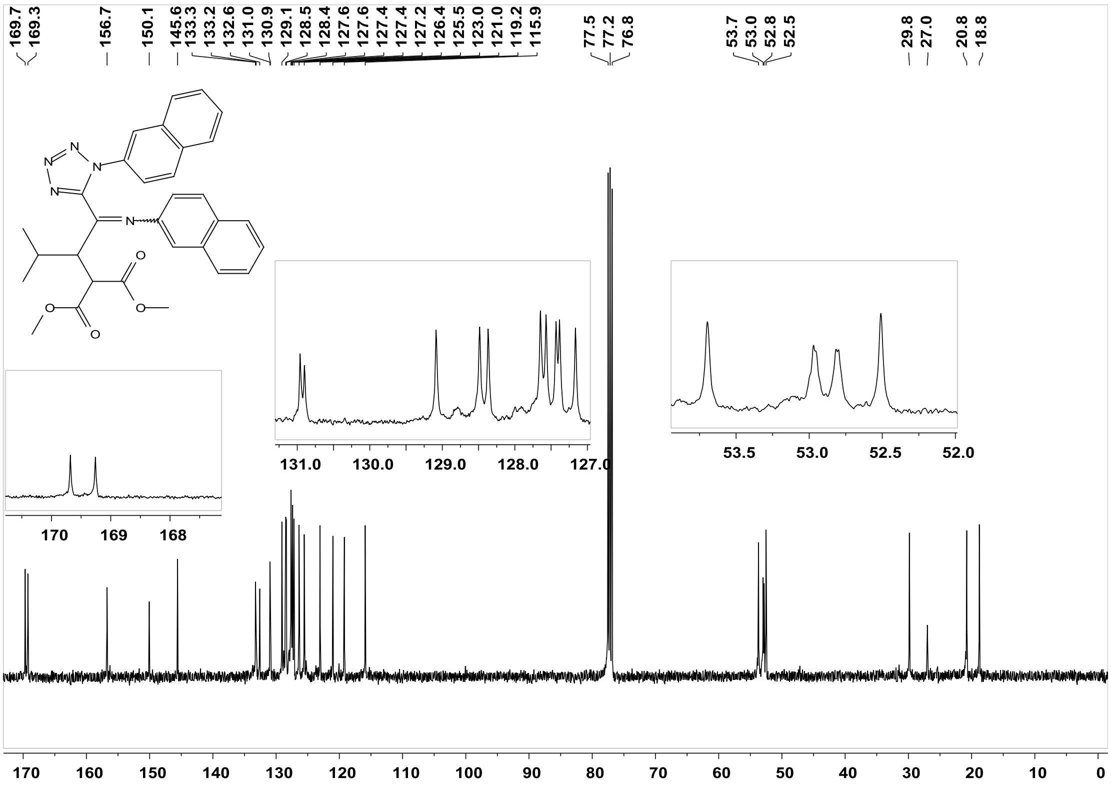

Supplementary Figure 86. ^{13}C NMR spectra for product **5g**

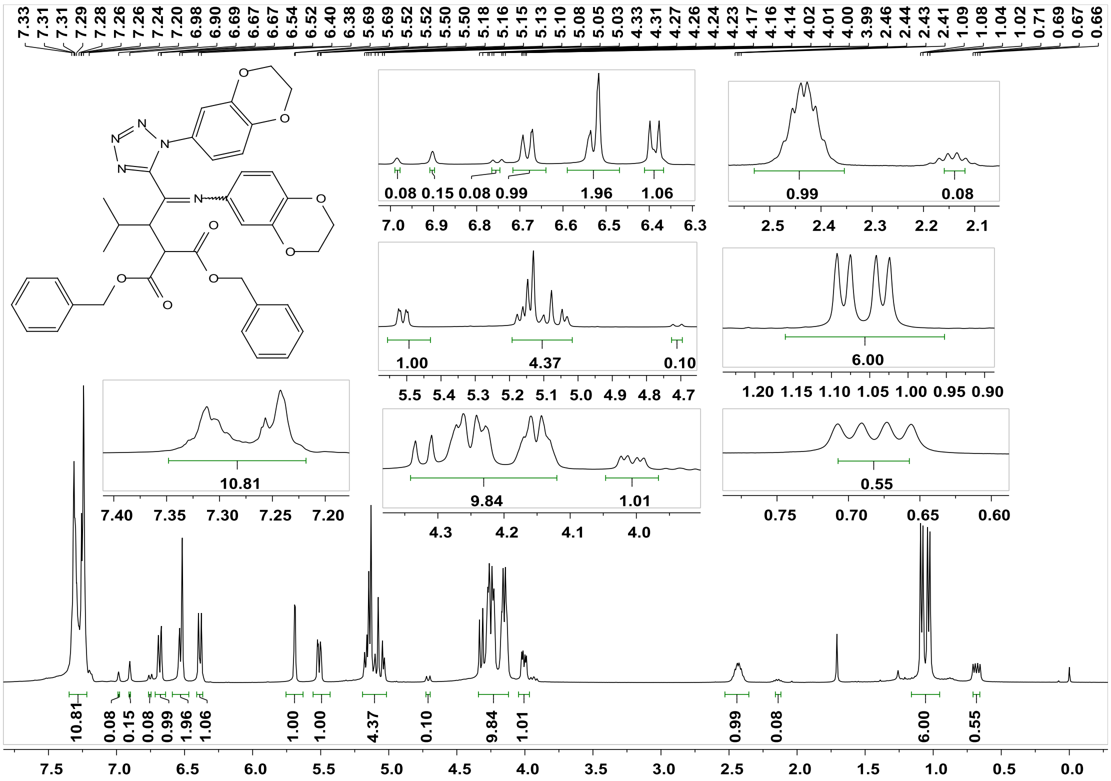

Supplementary Figure 87. ^{1}H NMR spectra for product **5h**

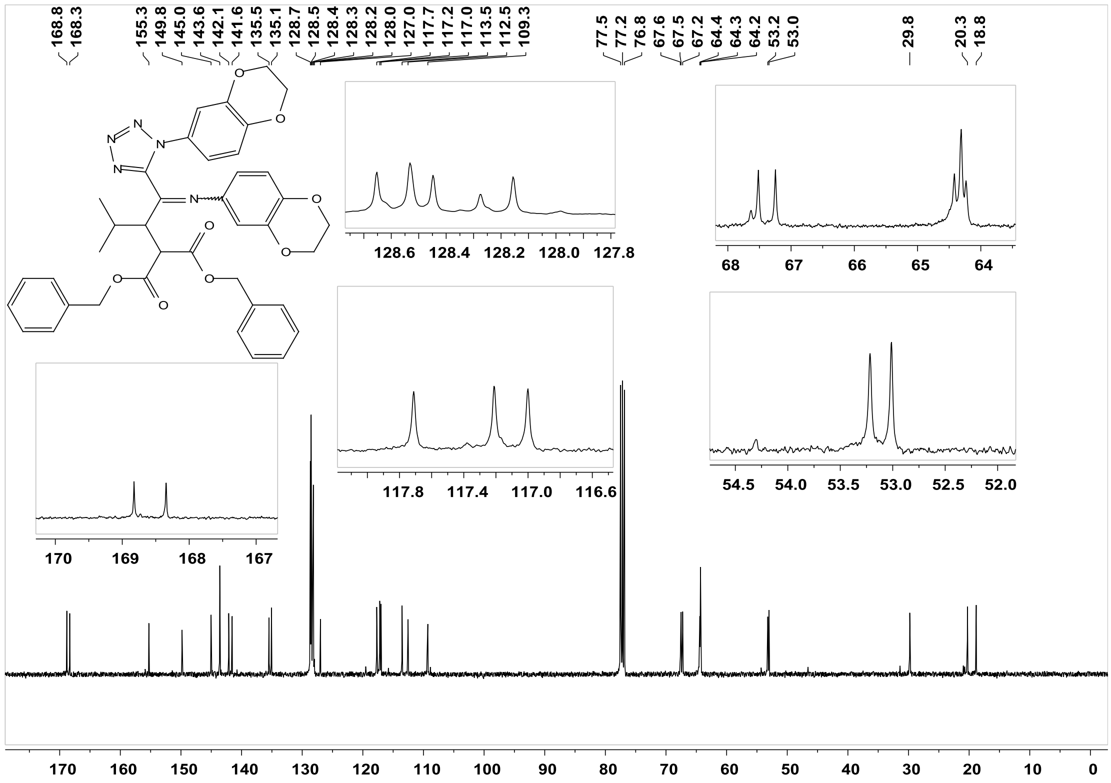

Supplementary Figure 88. ^{13}C NMR spectra for product **5h**

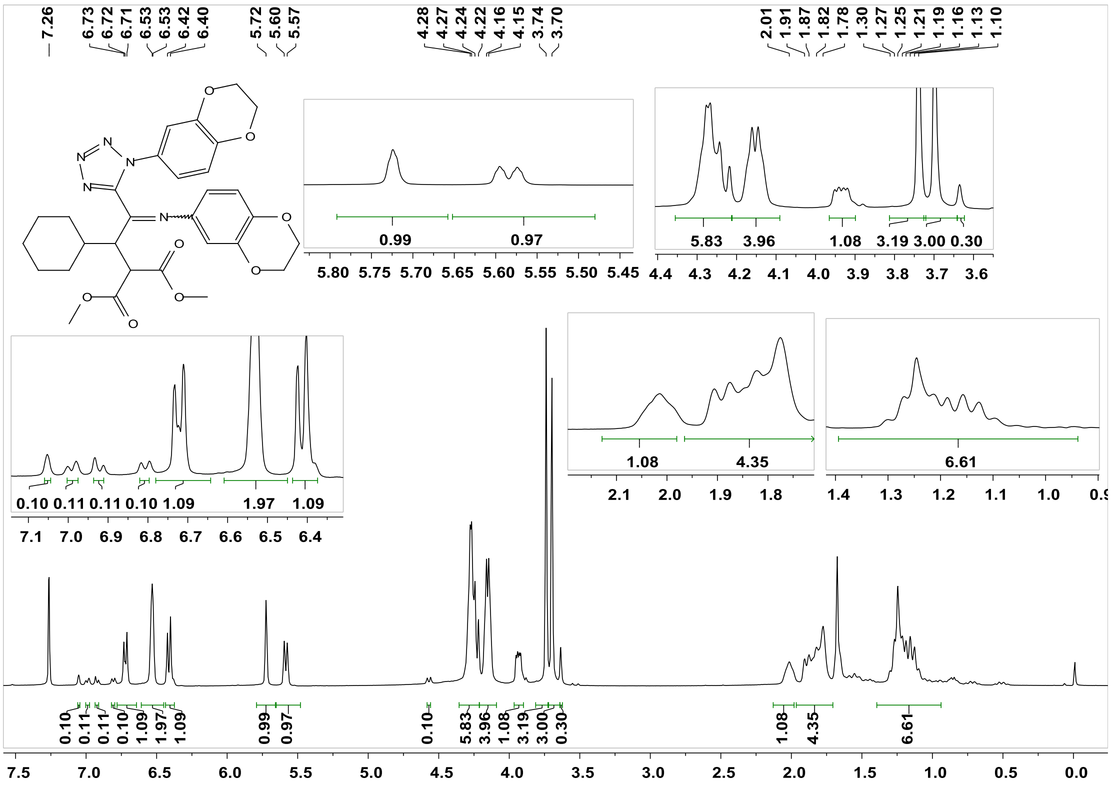

Supplementary Figure 89. ^{1}H NMR spectra for product **5i**

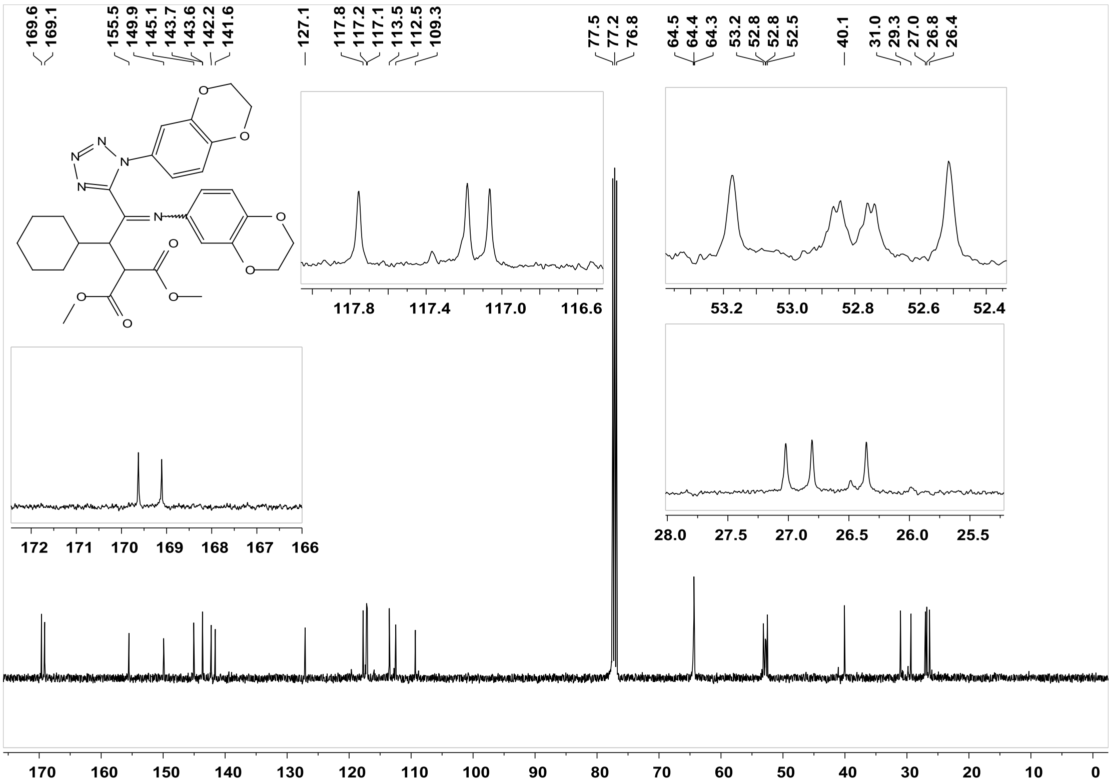

Supplementary Figure 90. ^{13}C NMR spectra for product **5i**

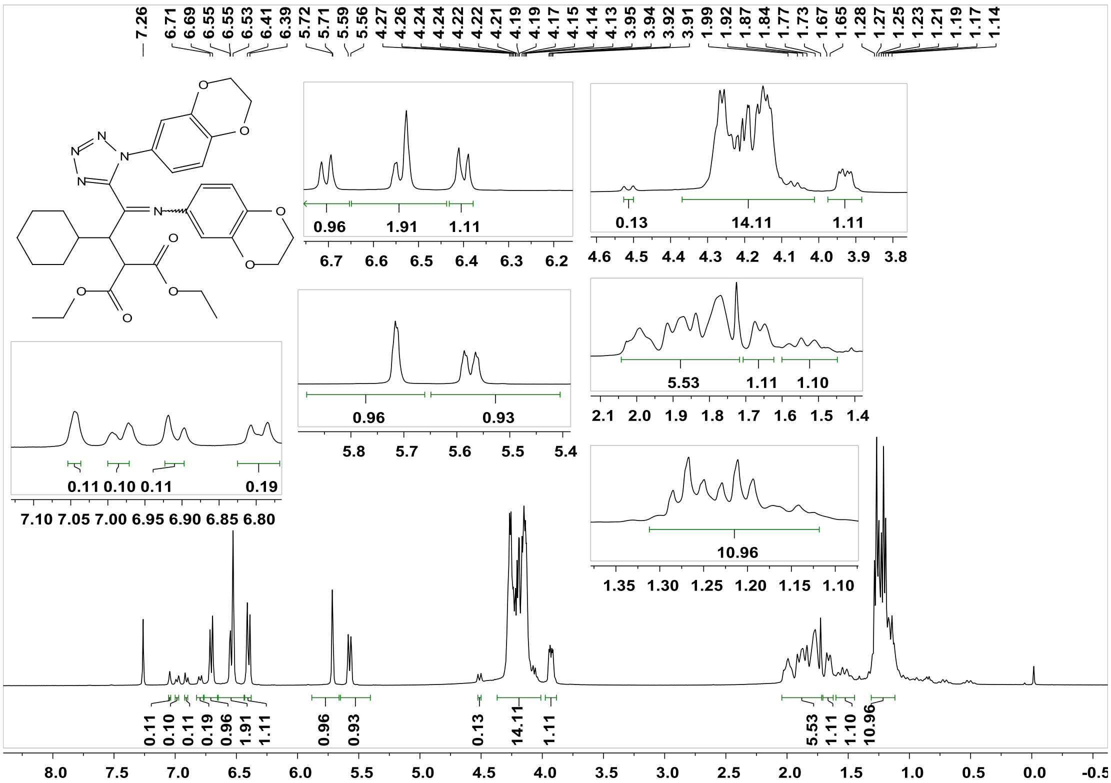

Supplementary Figure 91. ^{1}H NMR spectra for product **5j**

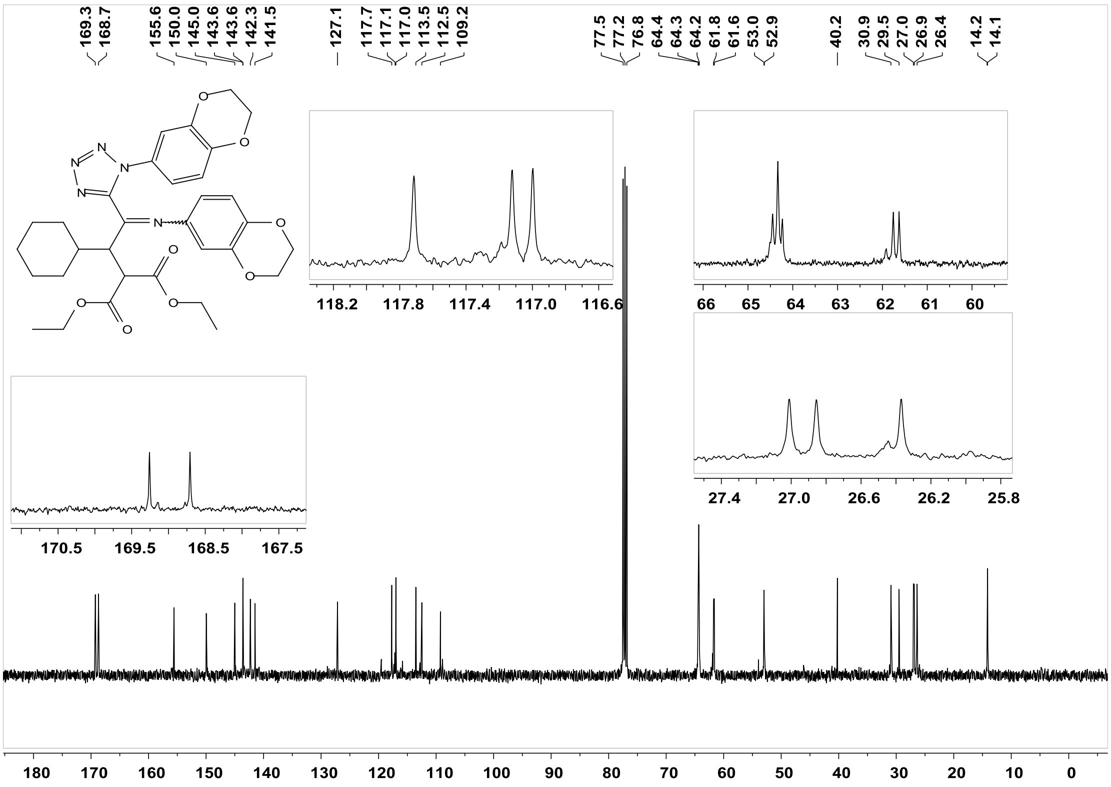

Supplementary Figure 92. ^{13}C NMR spectra for product **5j**

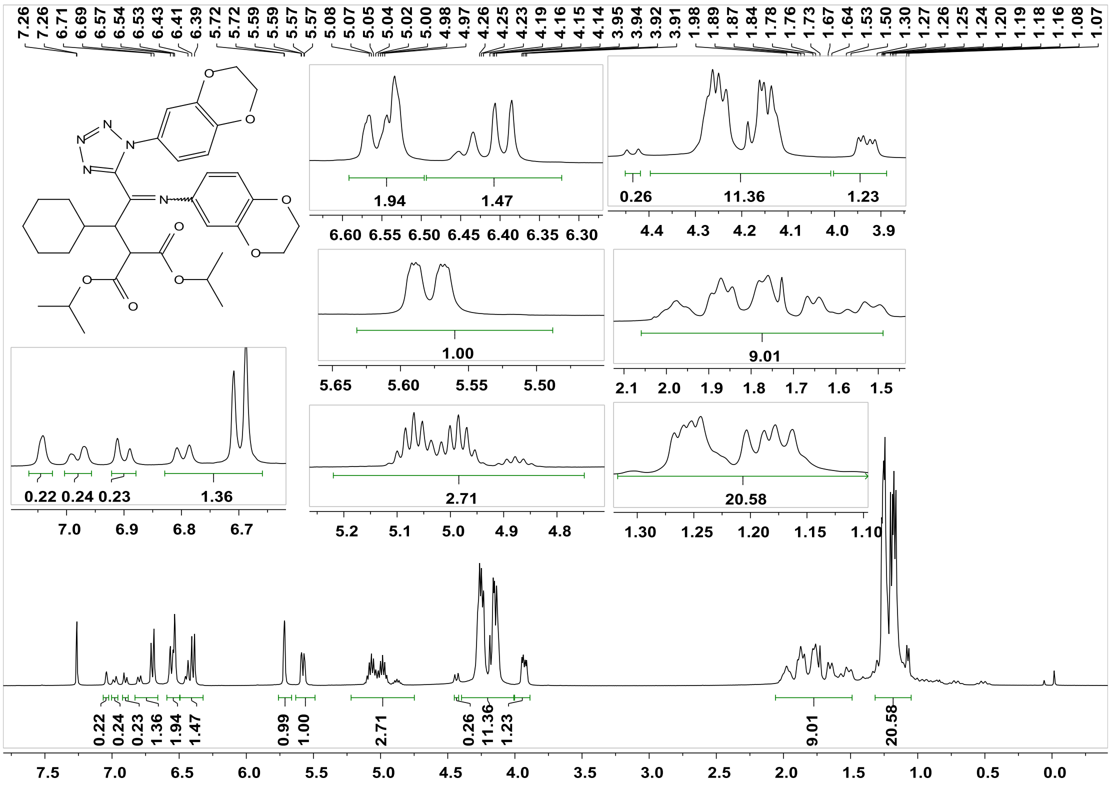

Supplementary Figure 93. ^{1}H NMR spectra for product **5k**

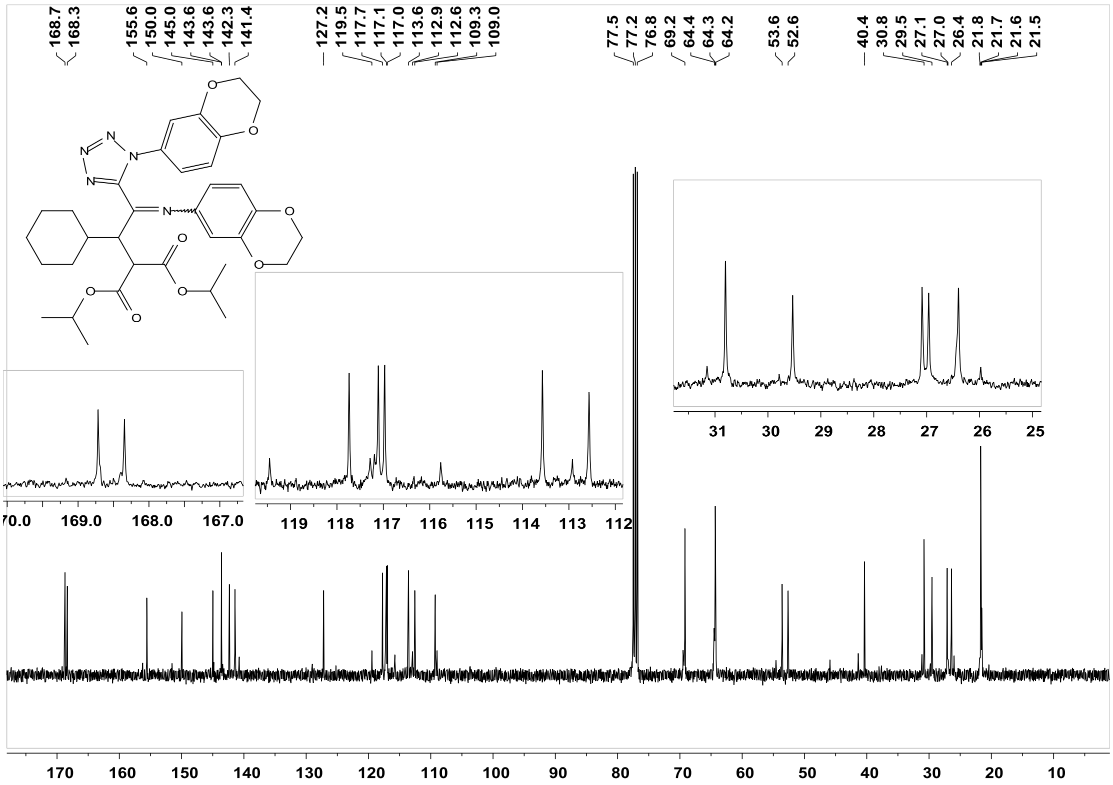

Supplementary Figure 94. ^{13}C NMR spectra for product **5k**

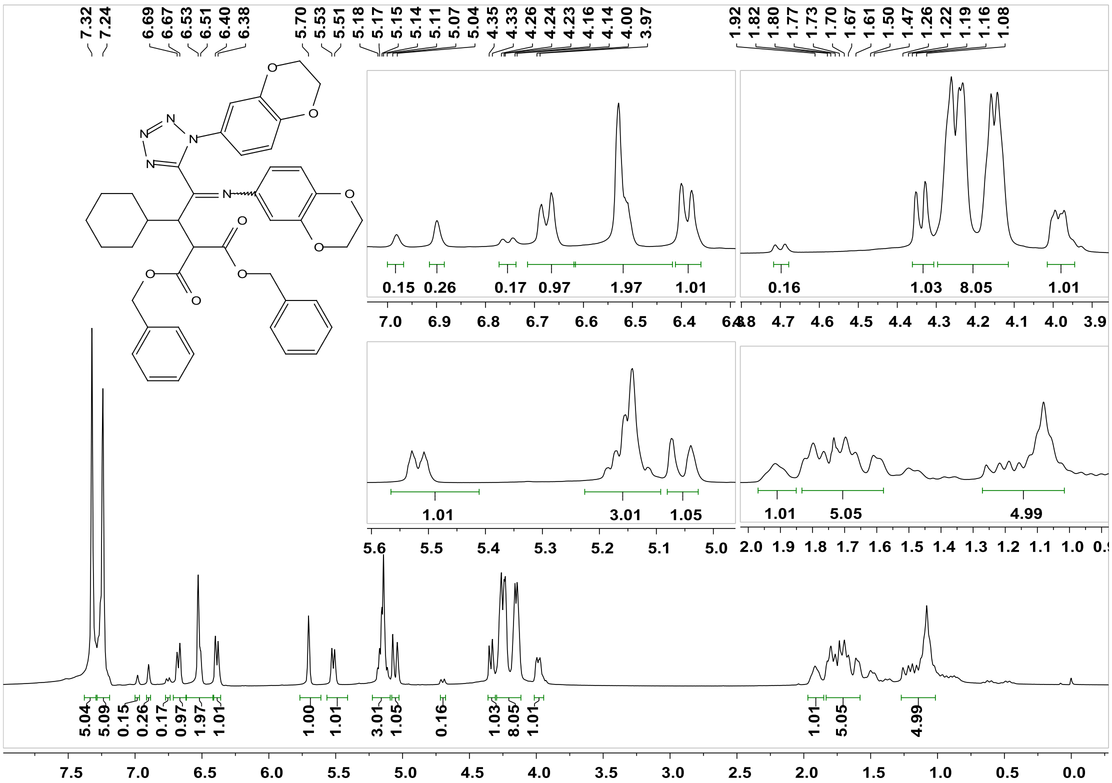

Supplementary Figure 95. ^{1}H NMR spectra for product **5l**

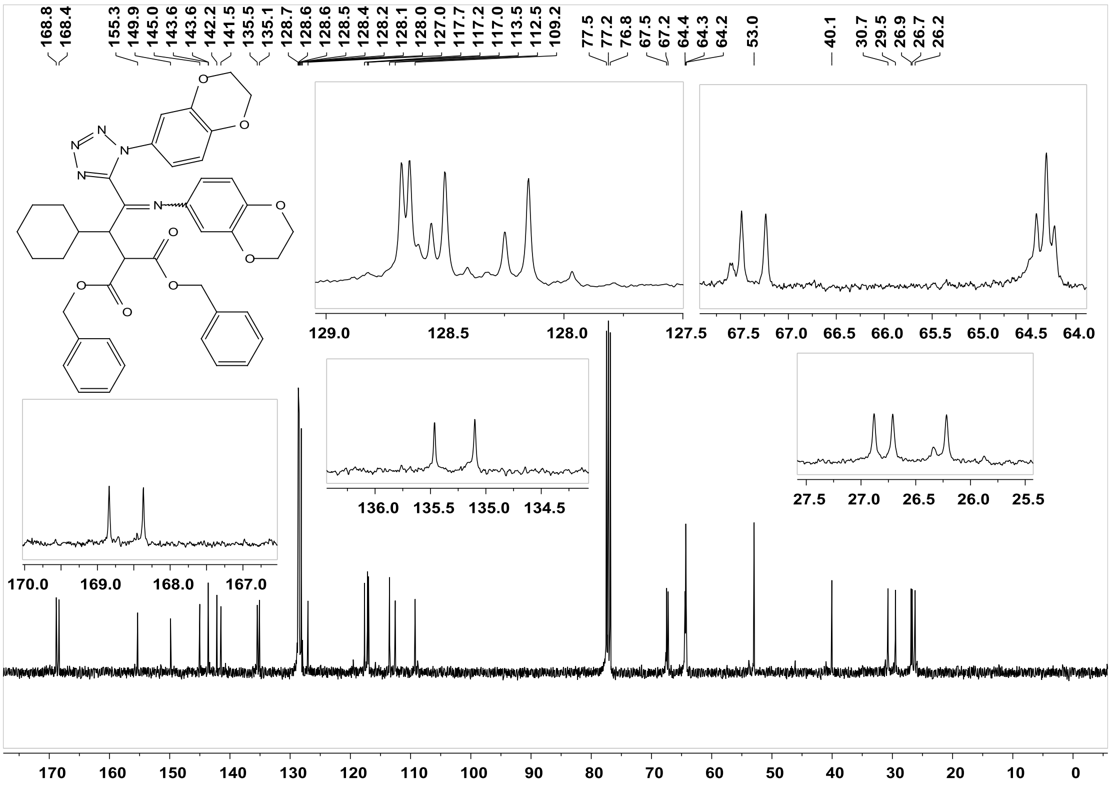

Supplementary Figure 96. ^{13}C NMR spectra for product **5l**

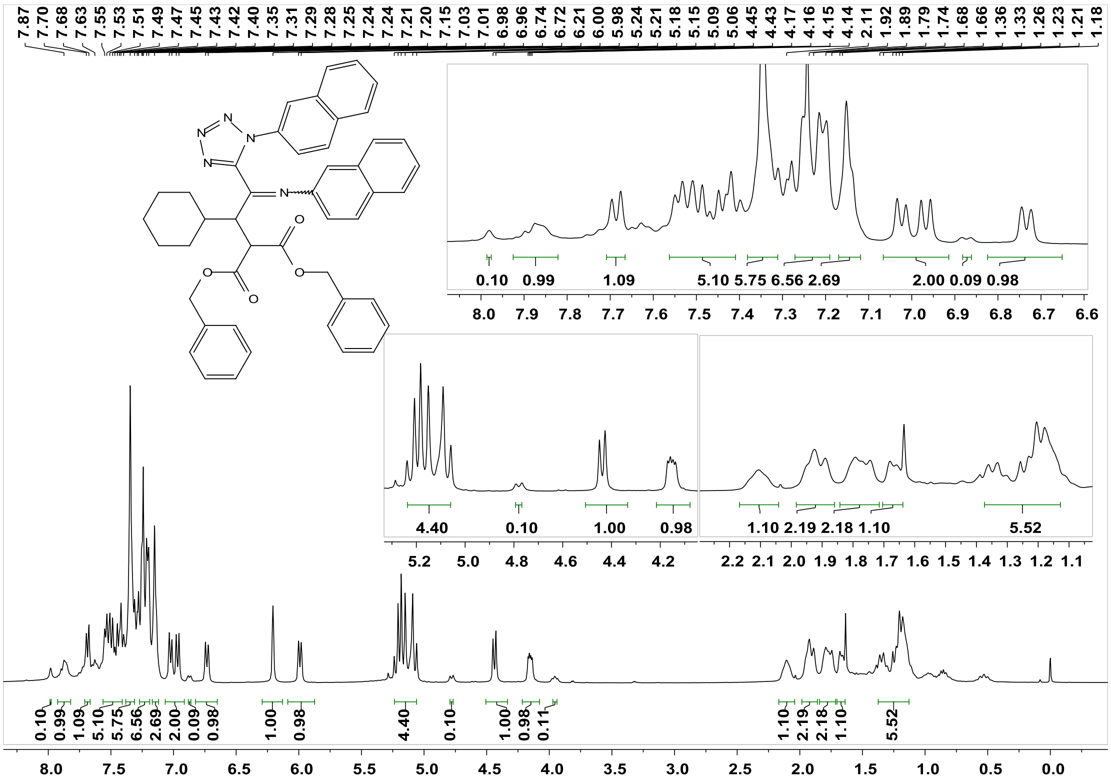

Supplementary Figure 97. ^{1}H NMR spectra for product **5m**

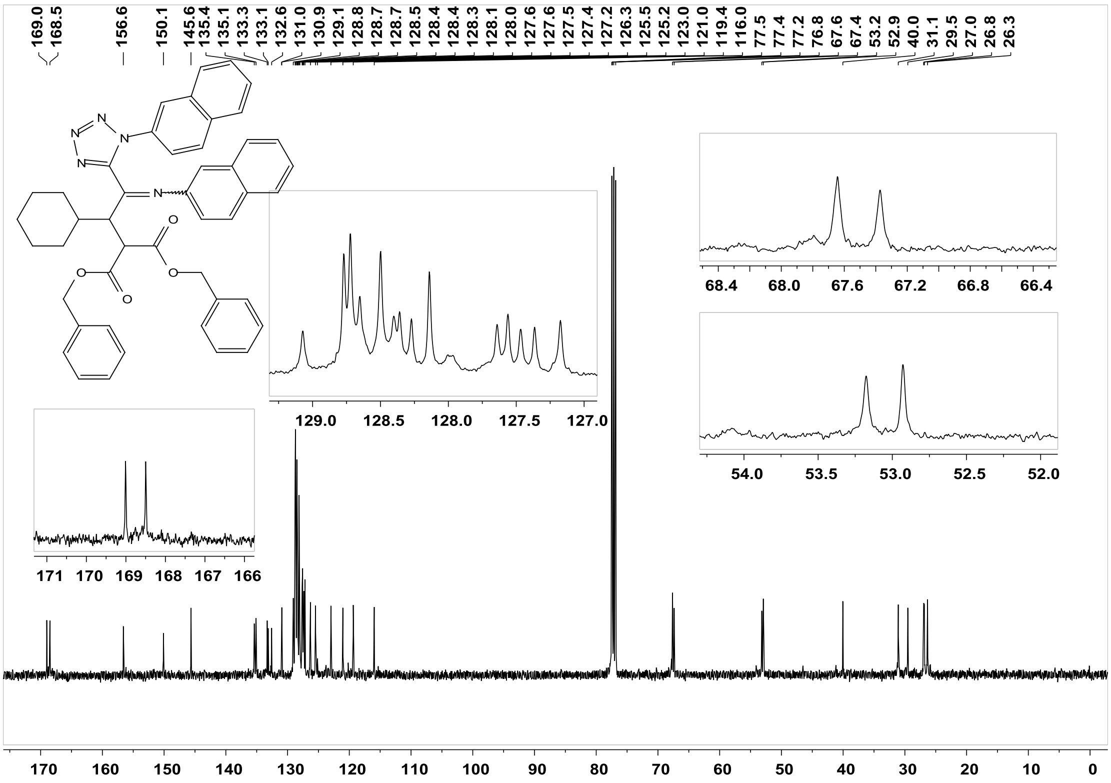

Supplementary Figure 98. ^{13}C NMR spectra for product **5m**

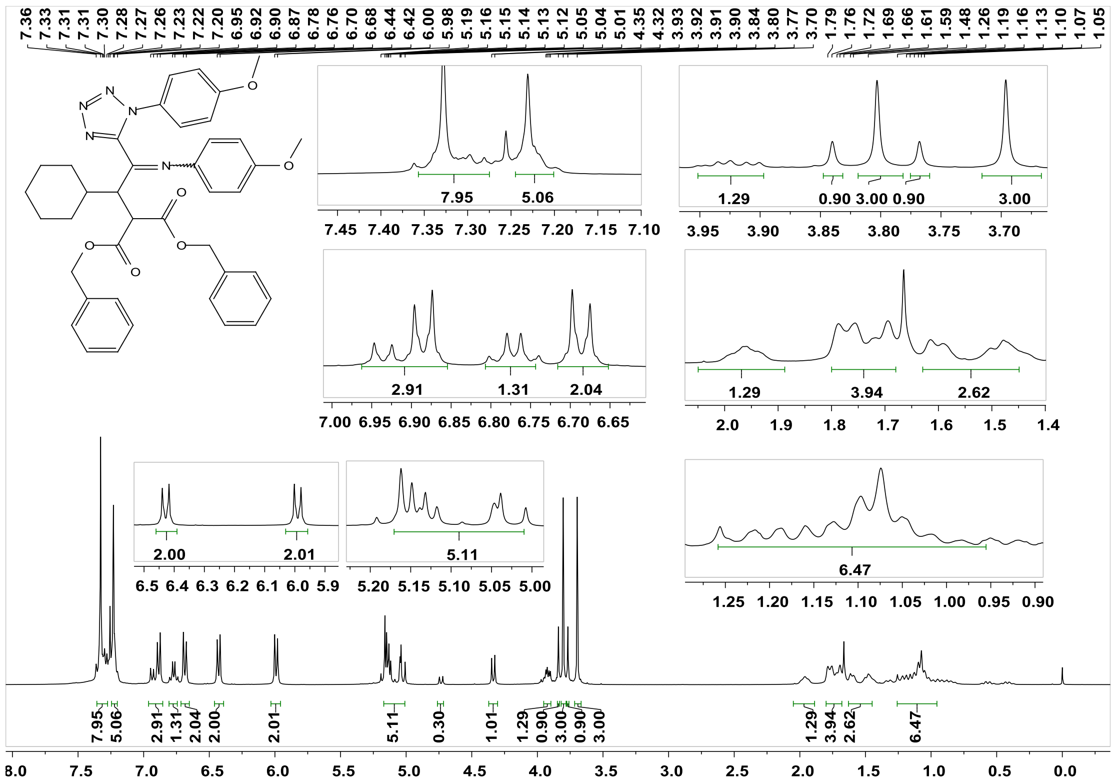

Supplementary Figure 99. ^{1}H NMR spectra for product **5n**

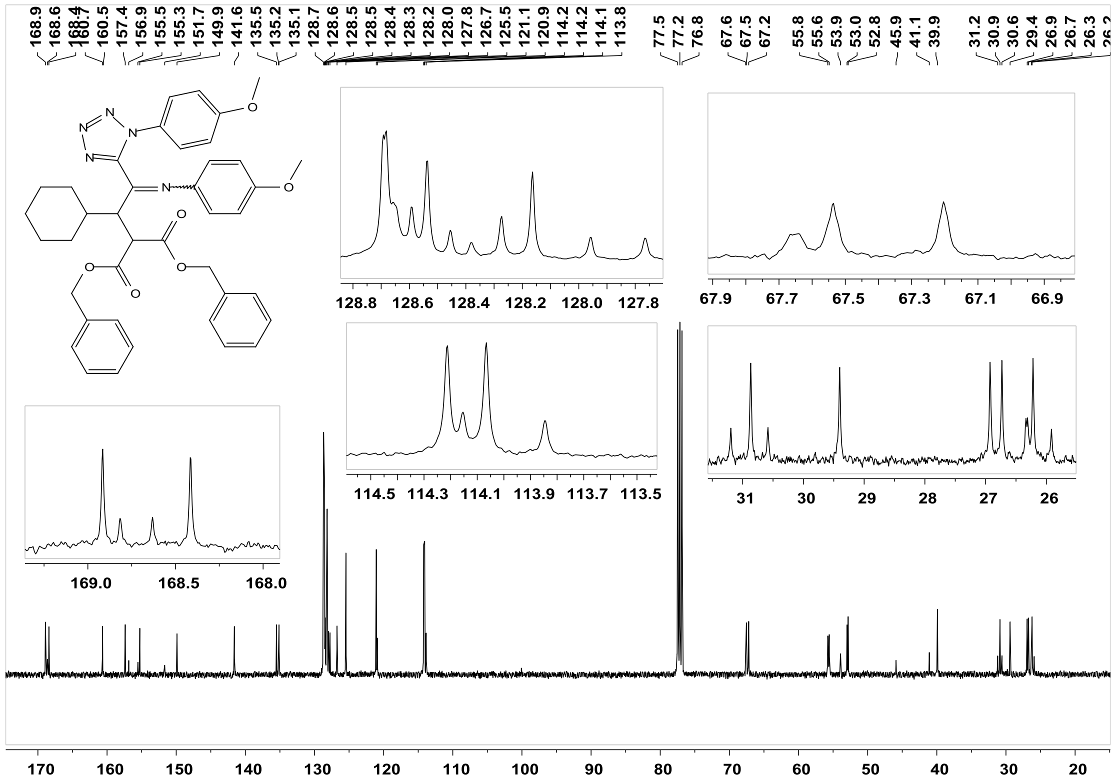

Supplementary Figure 100. ^{13}C NMR spectra for product **5n**

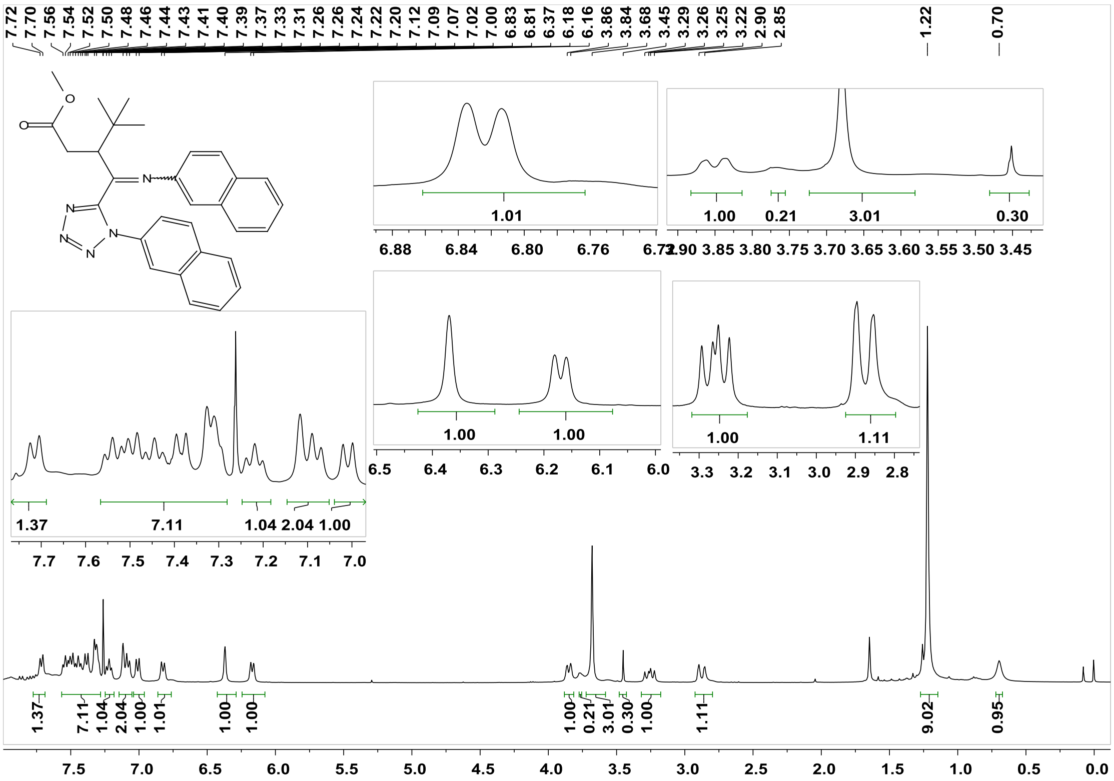

Supplementary Figure 101. ^{1}H NMR spectra for product **6e**

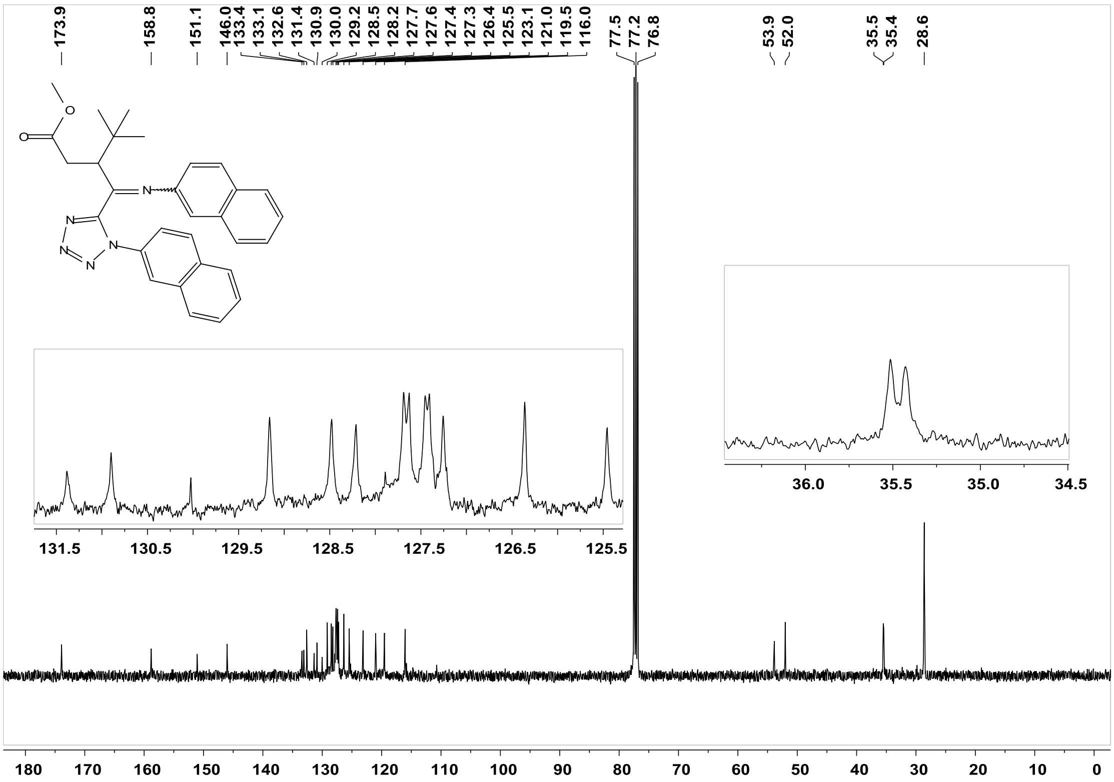

Supplementary Figure 102. ^{13}C NMR spectra for product **6e**

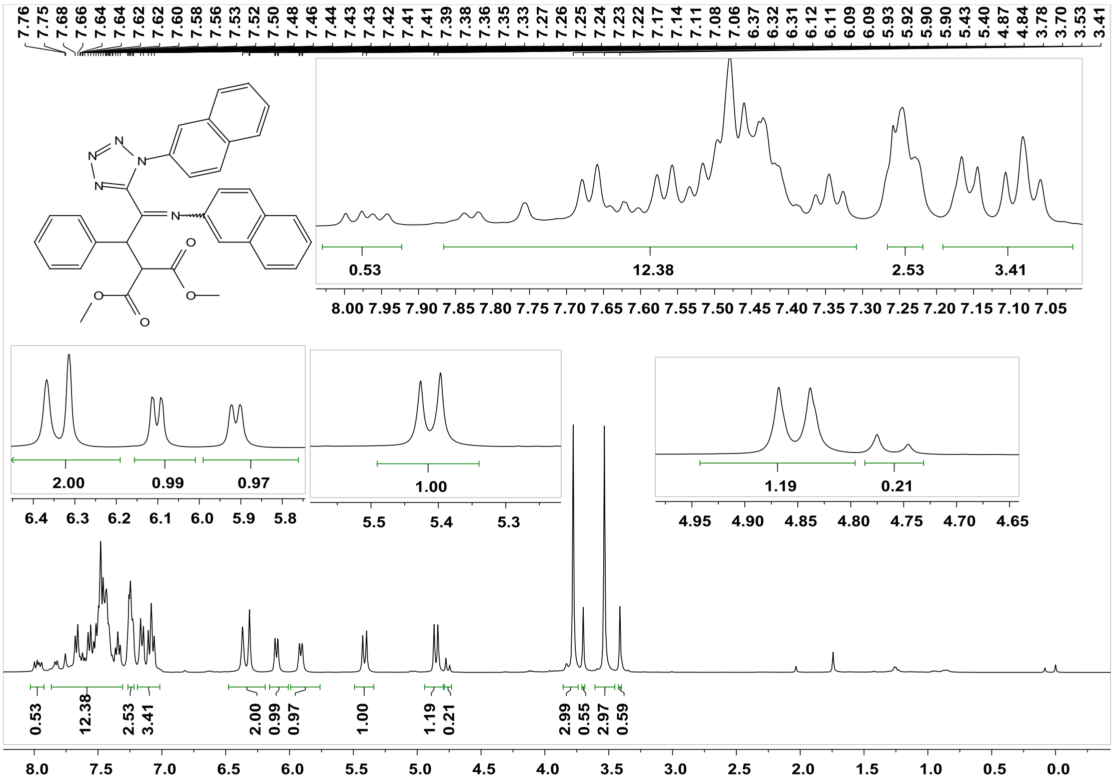

Supplementary Figure 103. ^{1}H NMR spectra for product **5o**

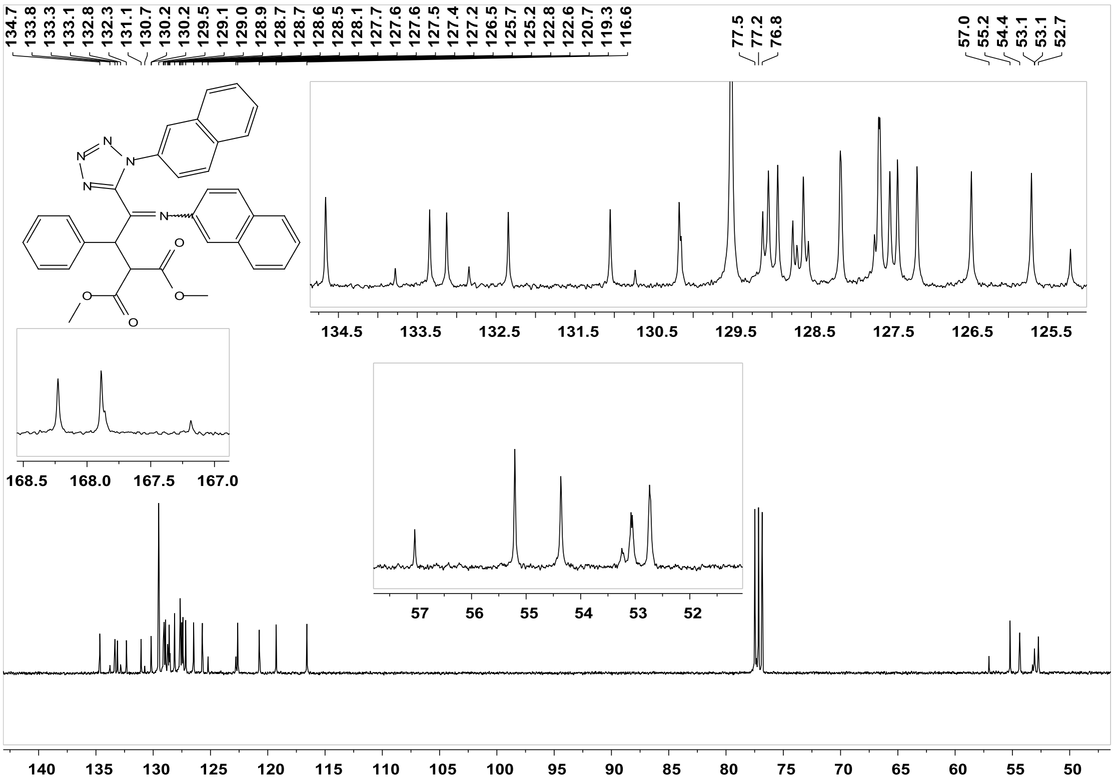

Supplementary Figure 104. ^{13}C NMR spectra for product **5o**

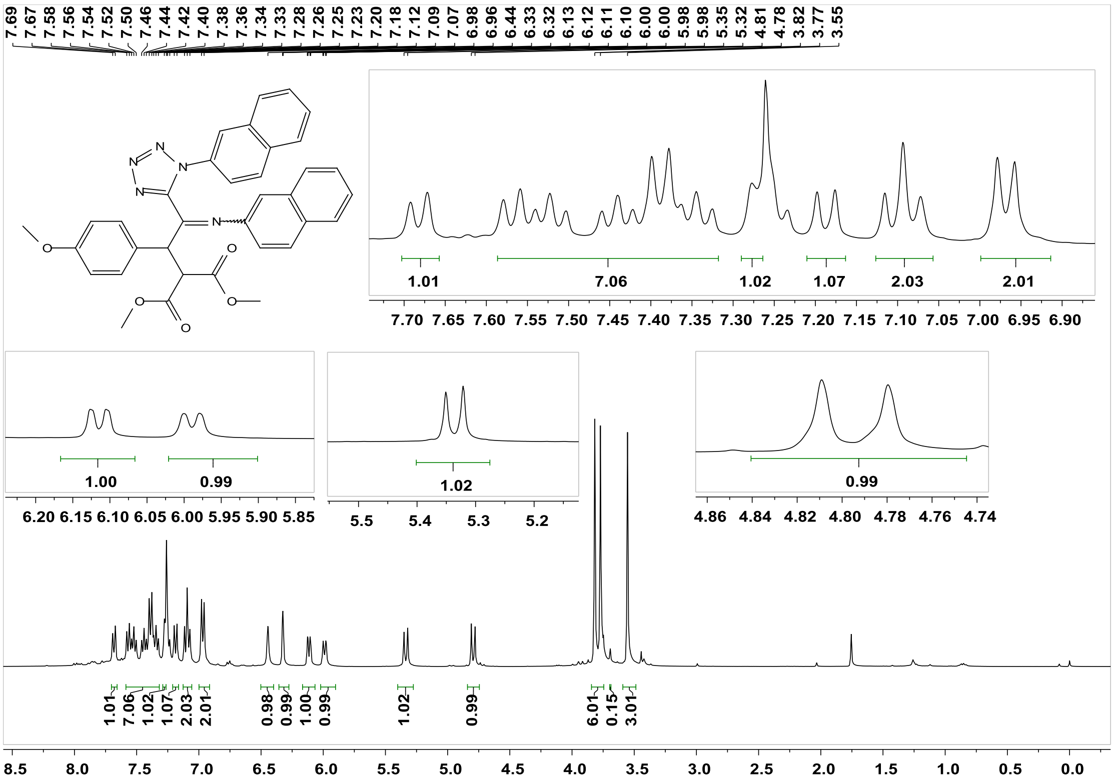

Supplementary Figure 105. ^{1}H NMR spectra for product **5p**

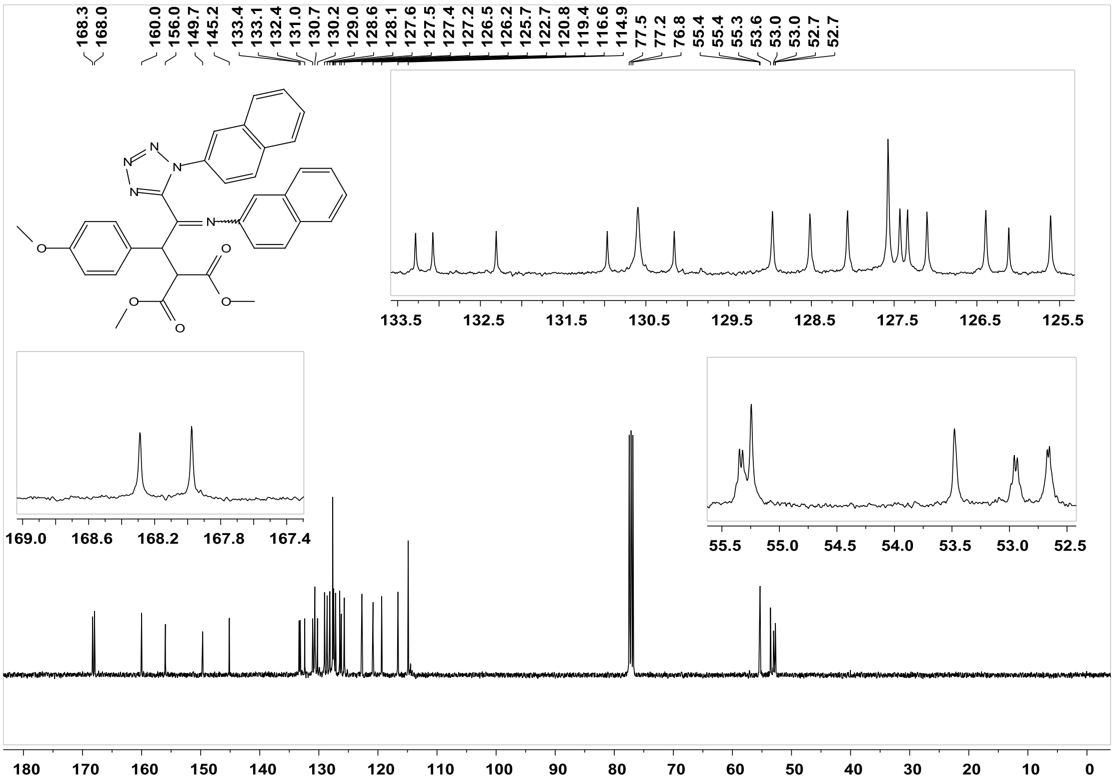

Supplementary Figure 106. ^{13}C NMR spectra for product **5p**

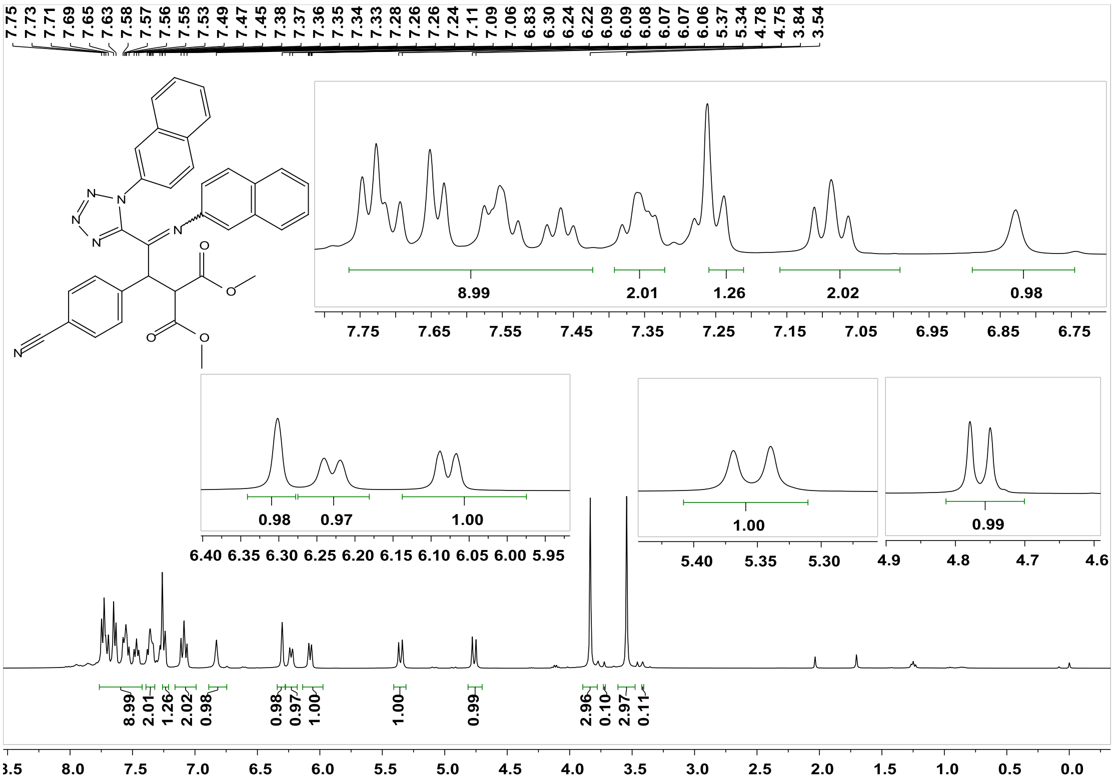

Supplementary Figure 107. ^{1}H NMR spectra for product **5q**

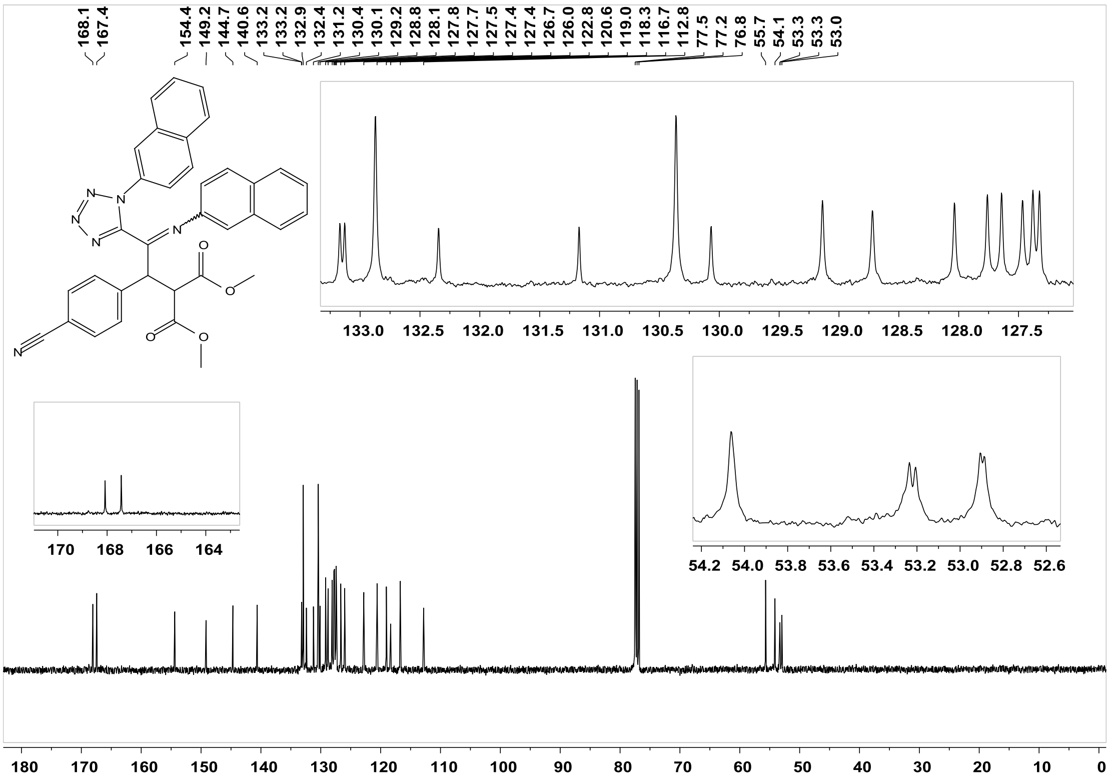

Supplementary Figure 108. ^{13}C NMR spectra for product **5q**

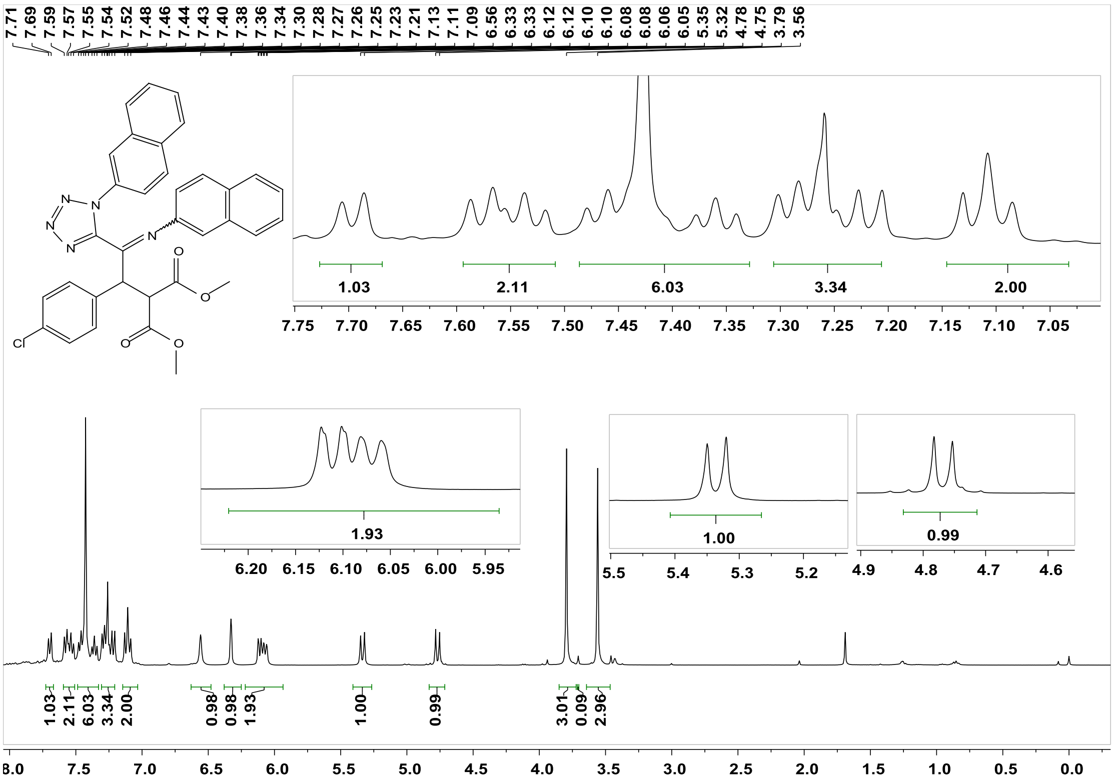

Supplementary Figure 109. ^{1}H NMR spectra for product **5r**

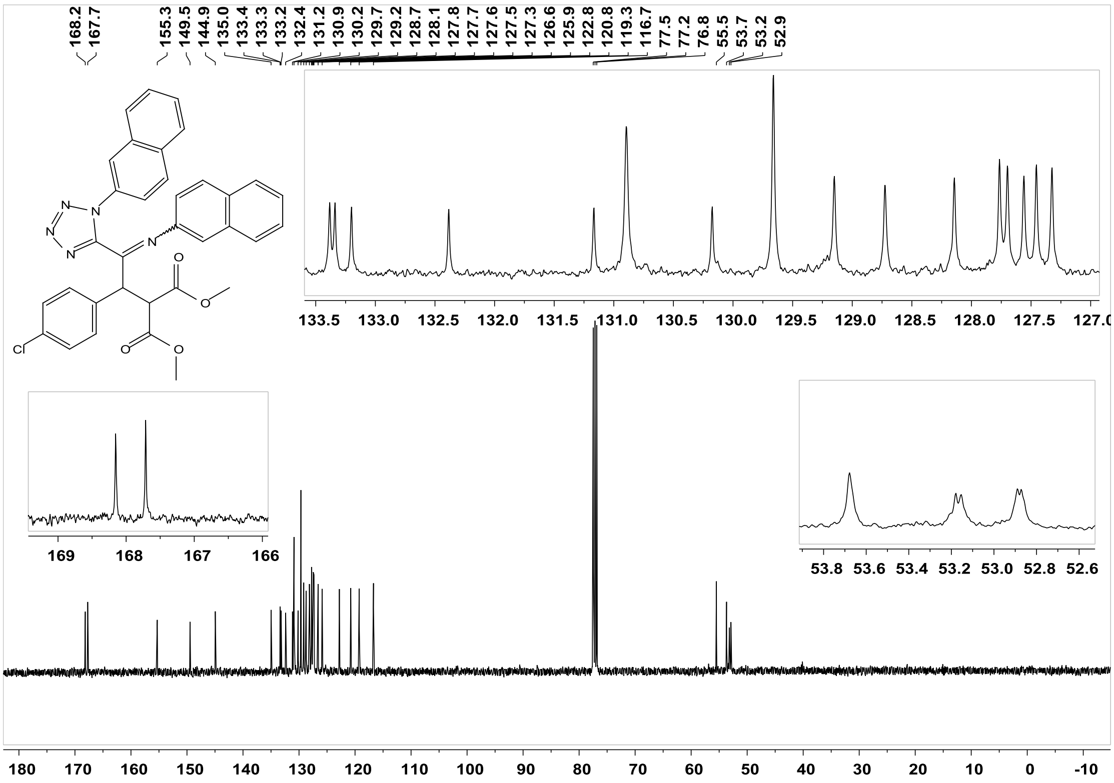

Supplementary Figure 110. ^{13}C NMR spectra for product **5r**

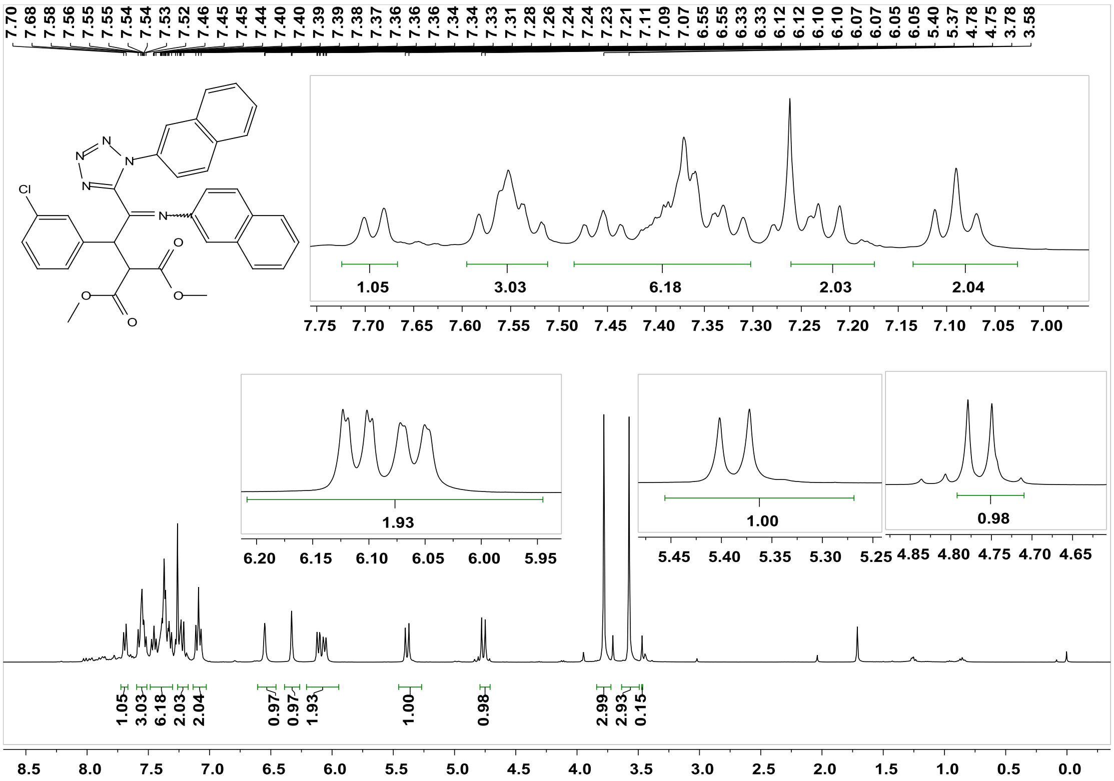

Supplementary Figure 111. ^{1}H NMR spectra for product **5s**

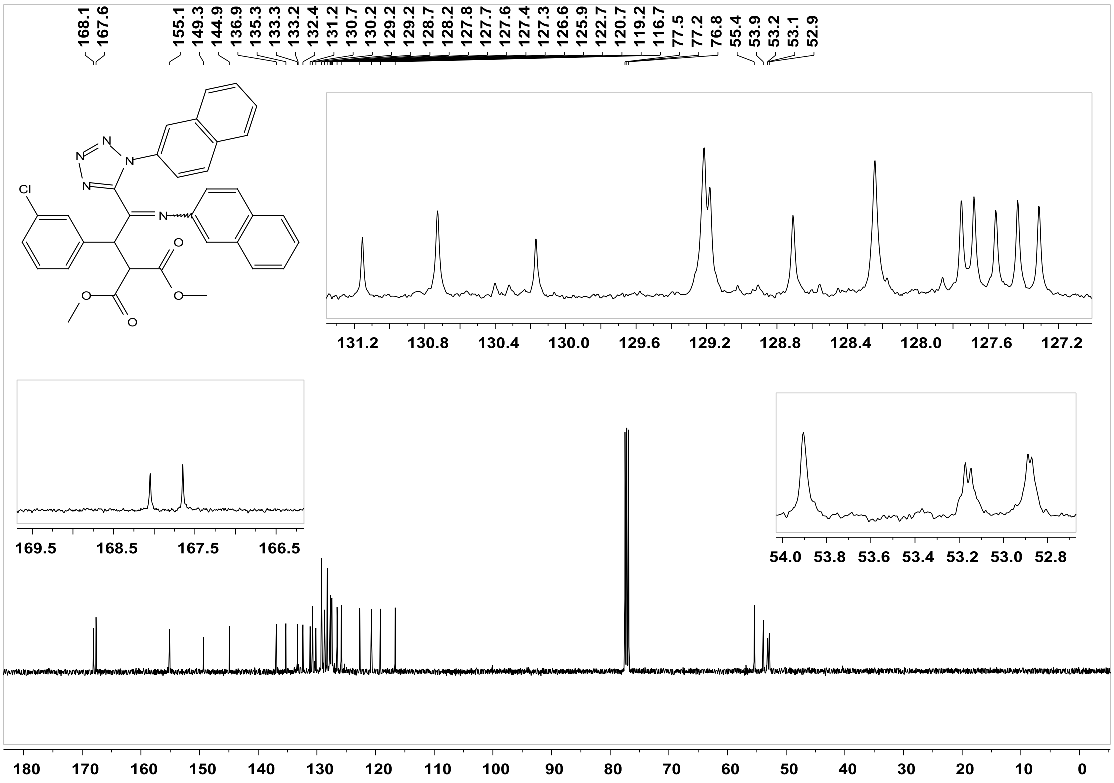

Supplementary Figure 112. ^{13}C NMR spectra for product **5s**

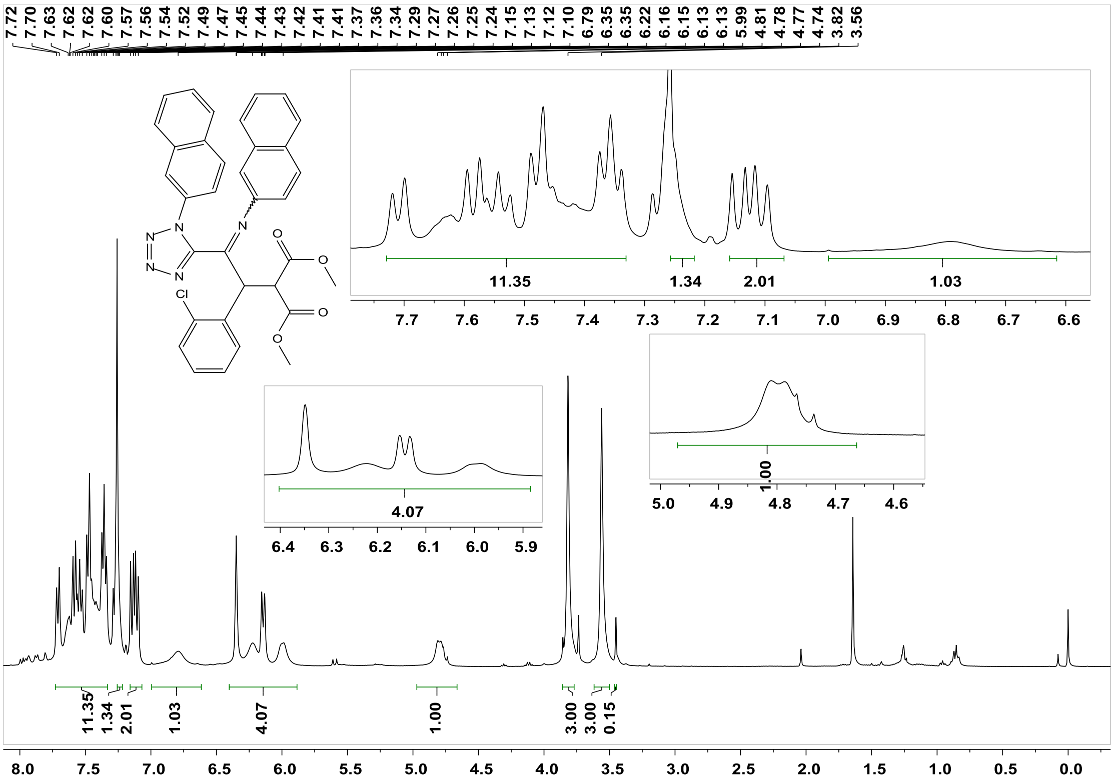

Supplementary Figure 113. ^{1}H NMR spectra for product **5t**

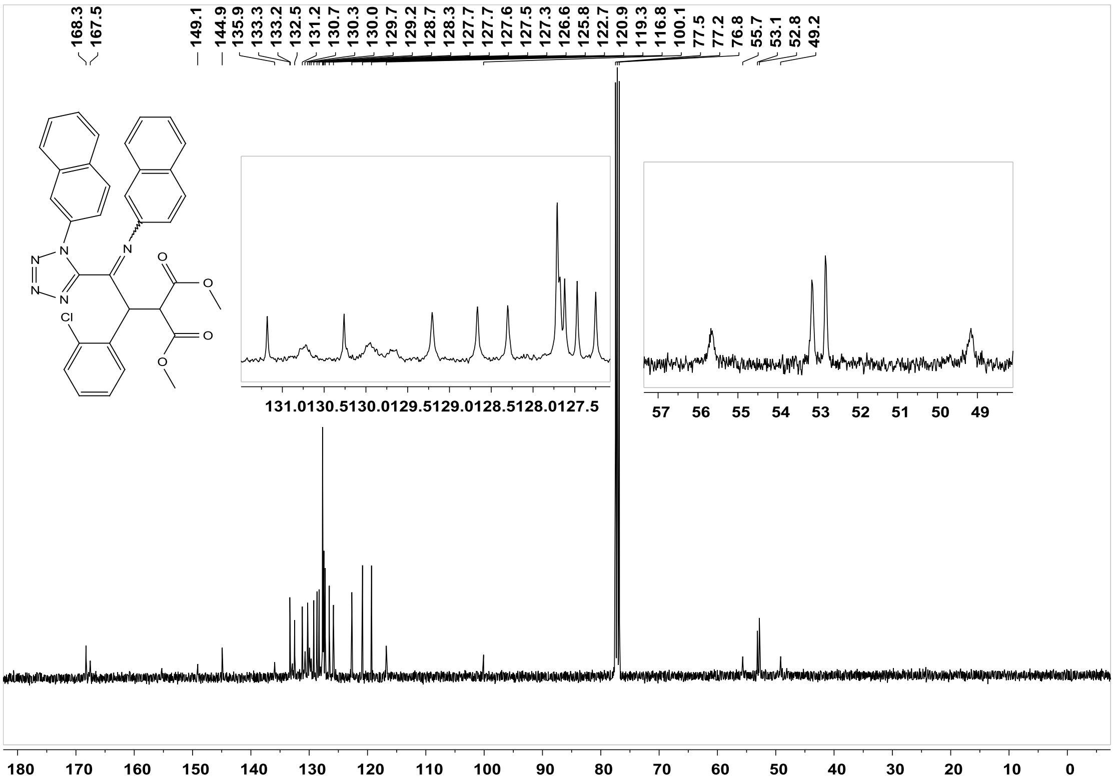

Supplementary Figure 114. ^{13}C NMR spectra for product **5t**

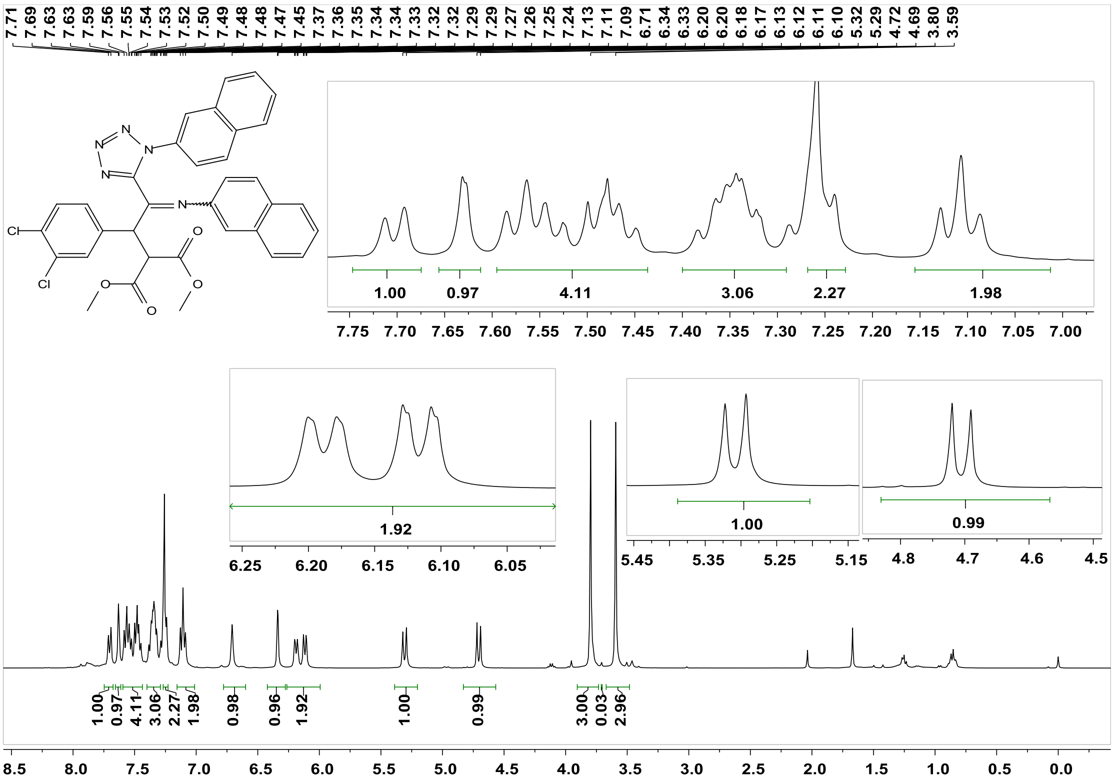

Supplementary Figure 115. ^{1}H NMR spectra for product **5u**

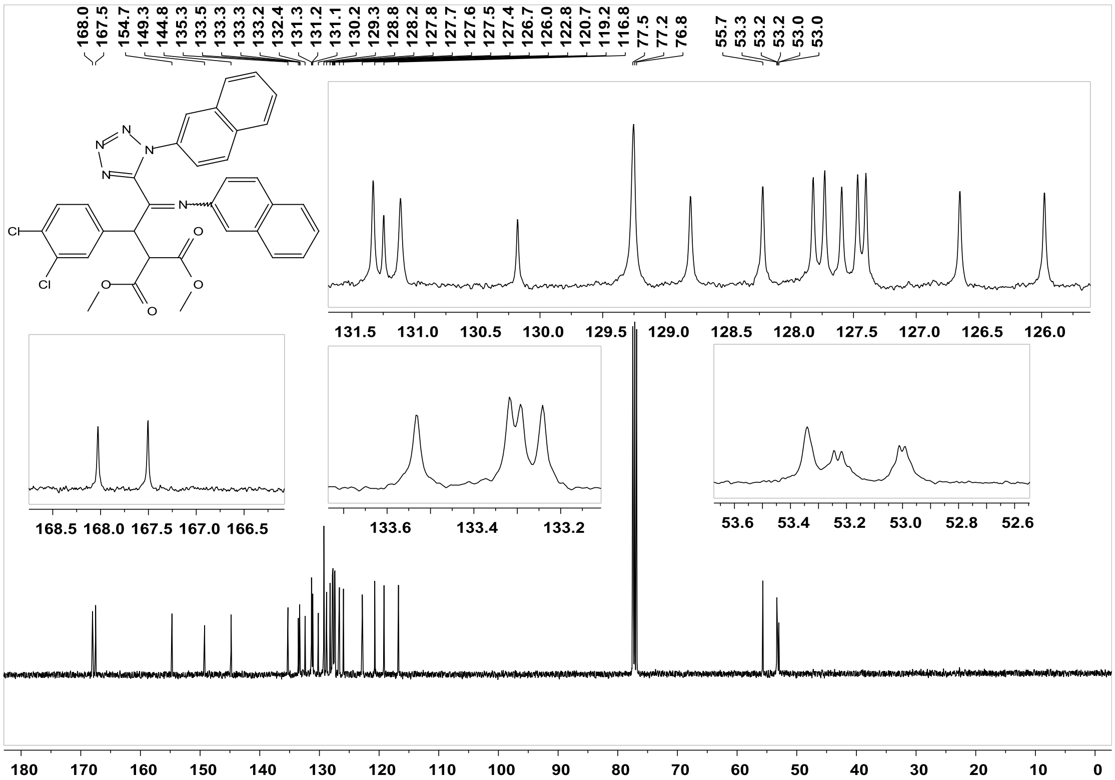

Supplementary Figure 116. ^{13}C NMR spectra for product **5u**

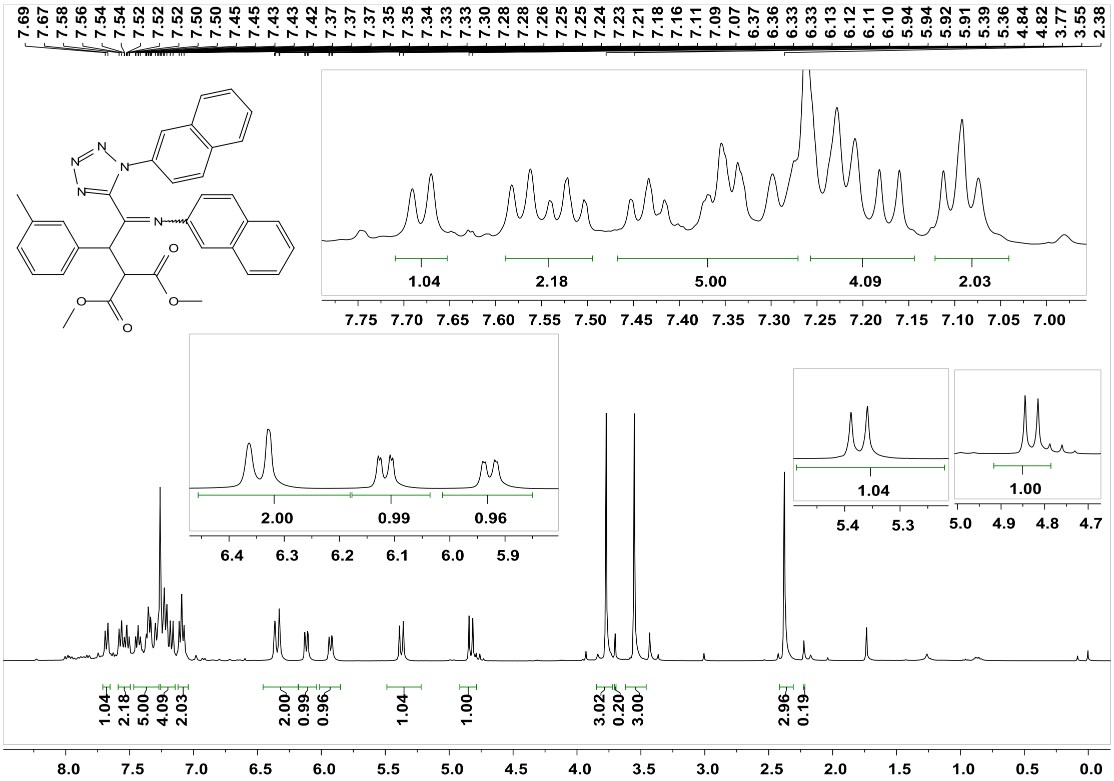

Supplementary Figure 117. ^{1}H NMR spectra for product **5v**

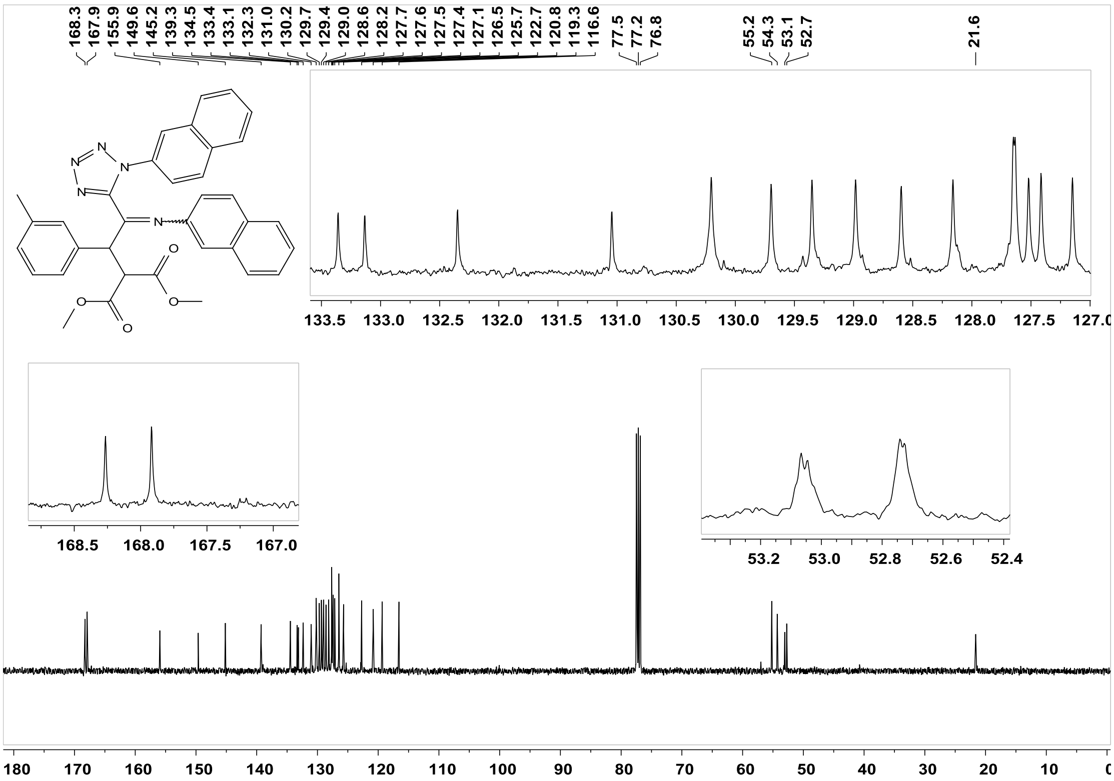

Supplementary Figure 118. ^{13}C NMR spectra for product **5v**

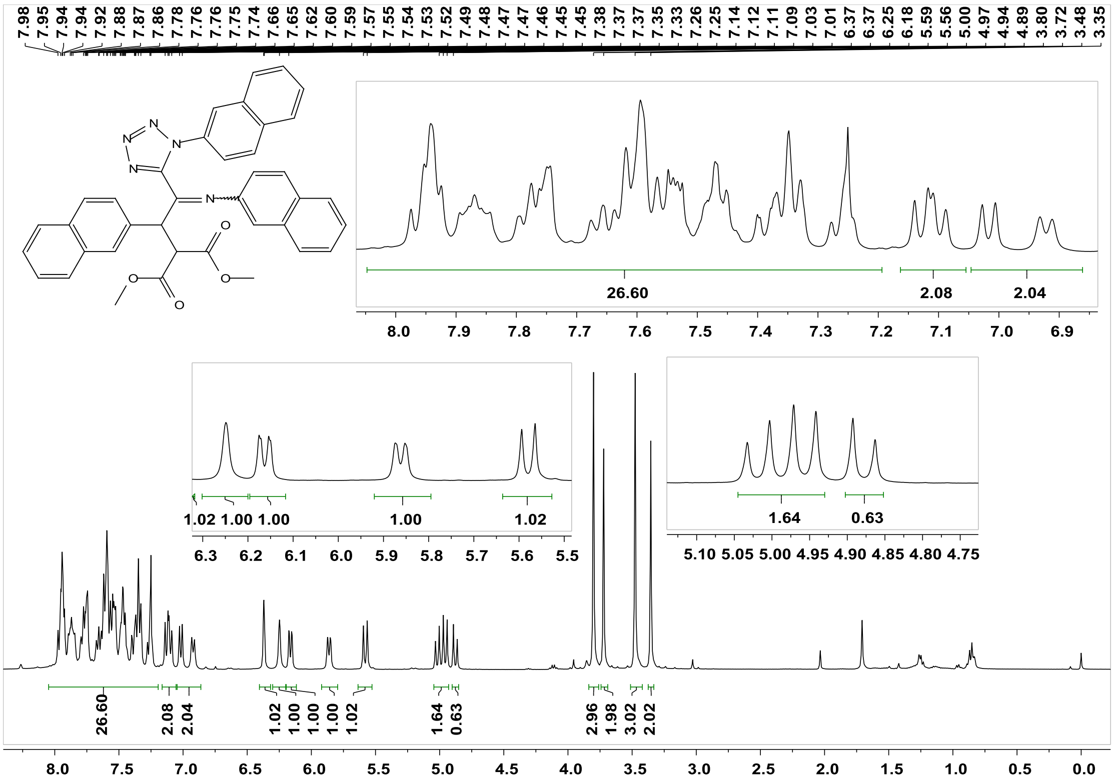

Supplementary Figure 119. ^{1}H NMR spectra for product **5w**

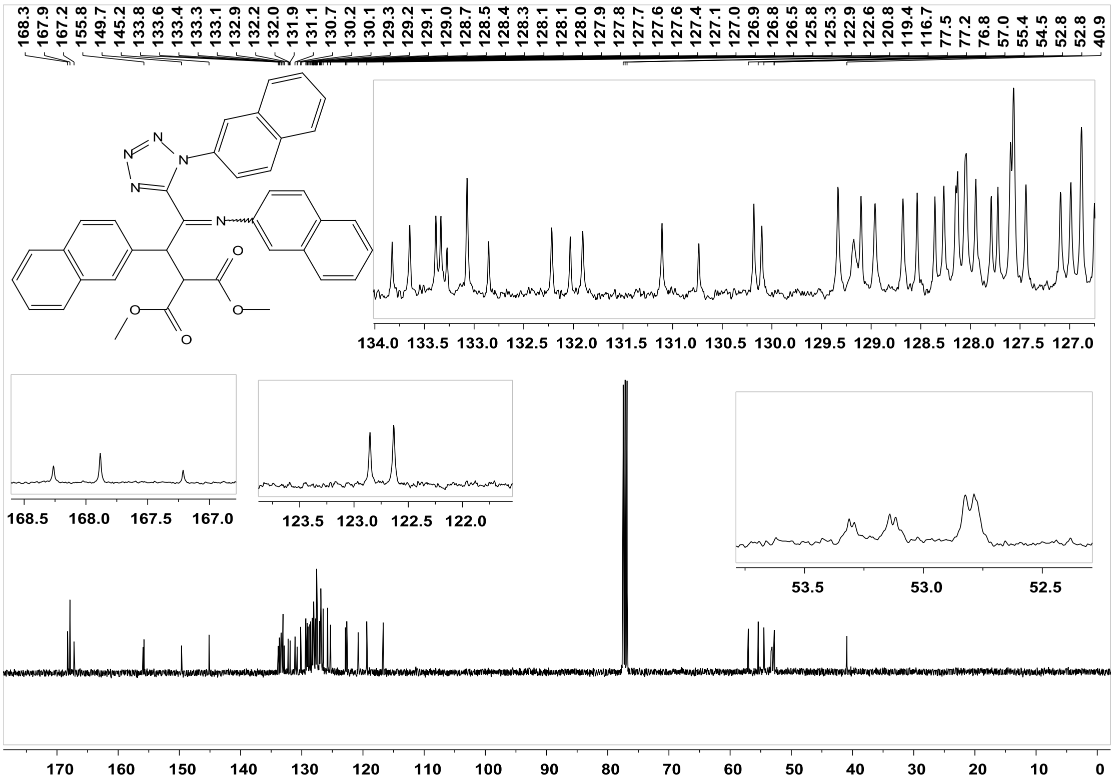

Supplementary Figure 120. ^{13}C NMR spectra for product **5w**

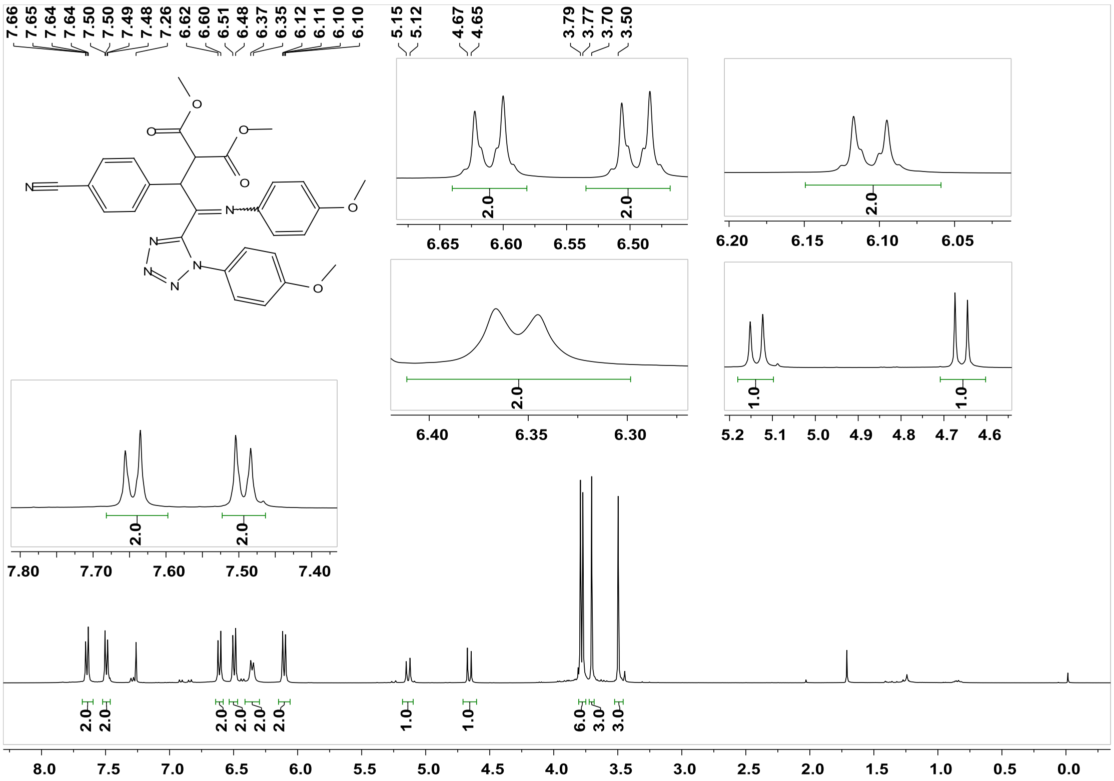

Supplementary Figure 121. ^{1}H NMR spectra for product **5x**

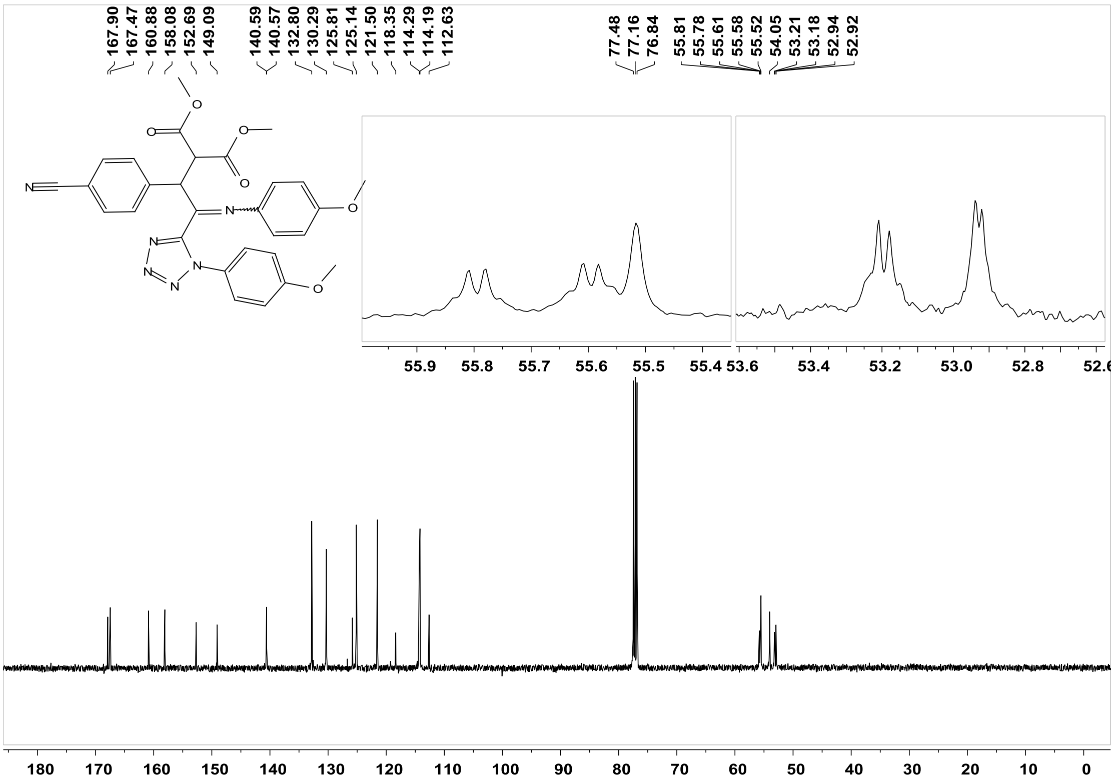

Supplementary Figure 122. ^{13}C NMR spectra for product **5x**

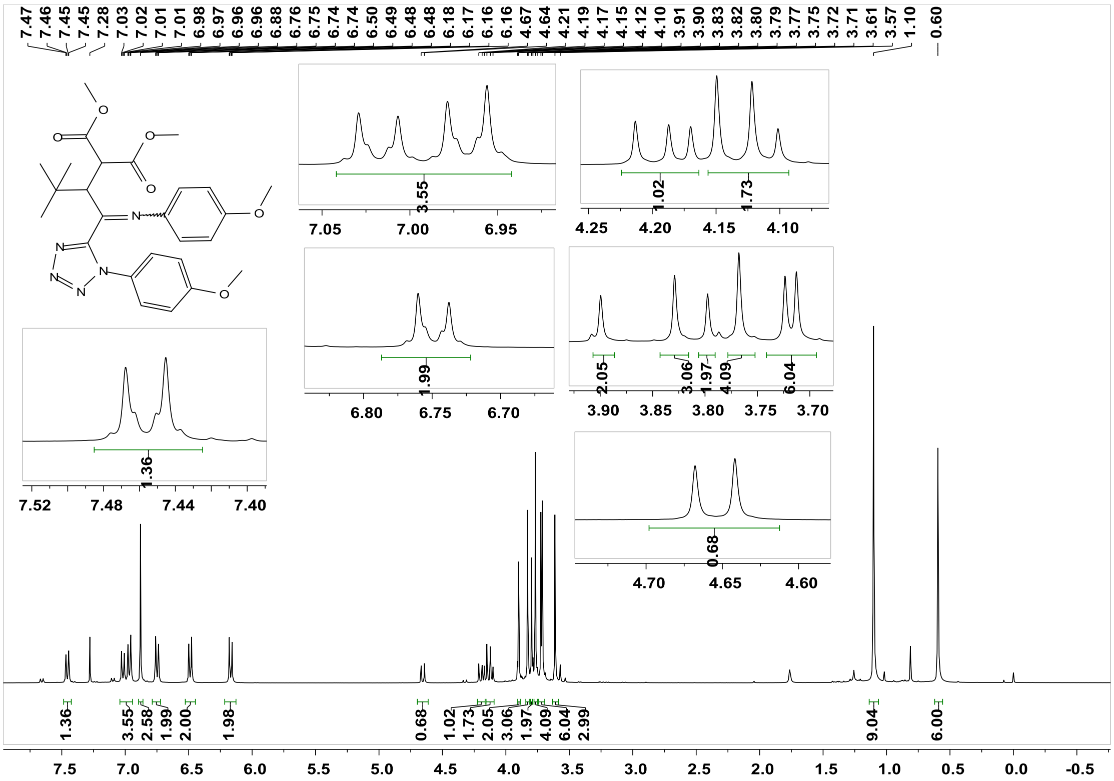

Supplementary Figure 123. ^{1}H NMR spectra for product **5z**

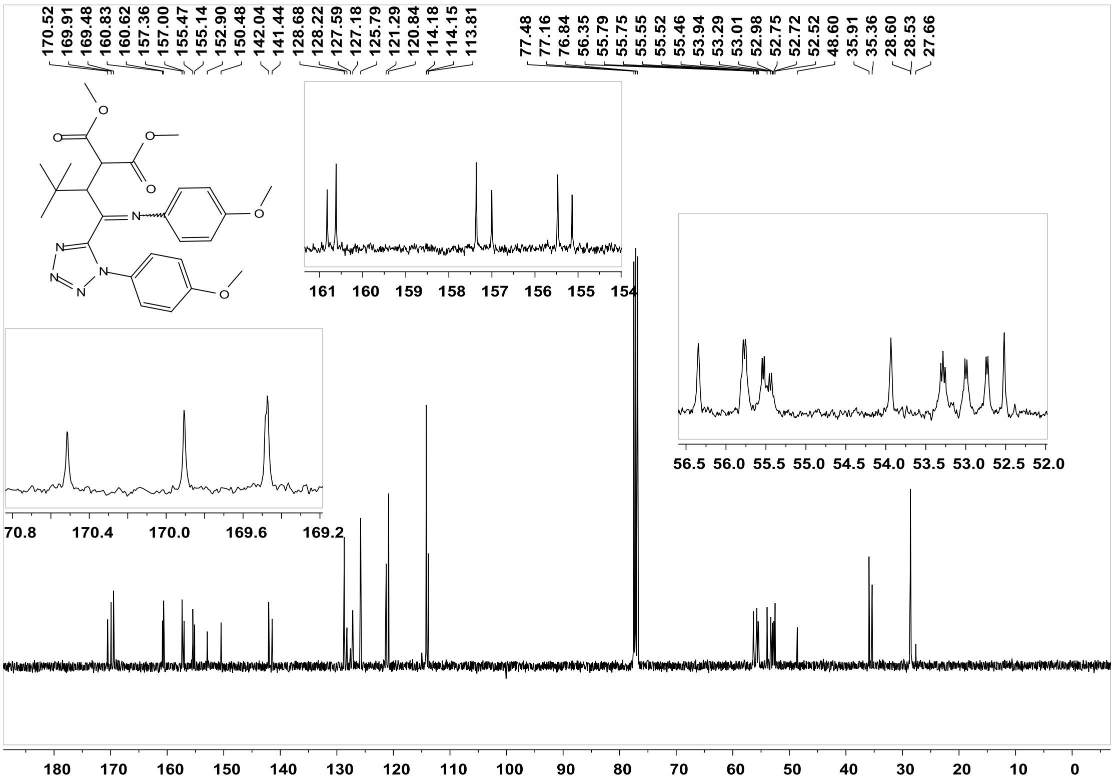

Supplementary Figure 124. ^{13}C NMR spectra for product **5z**

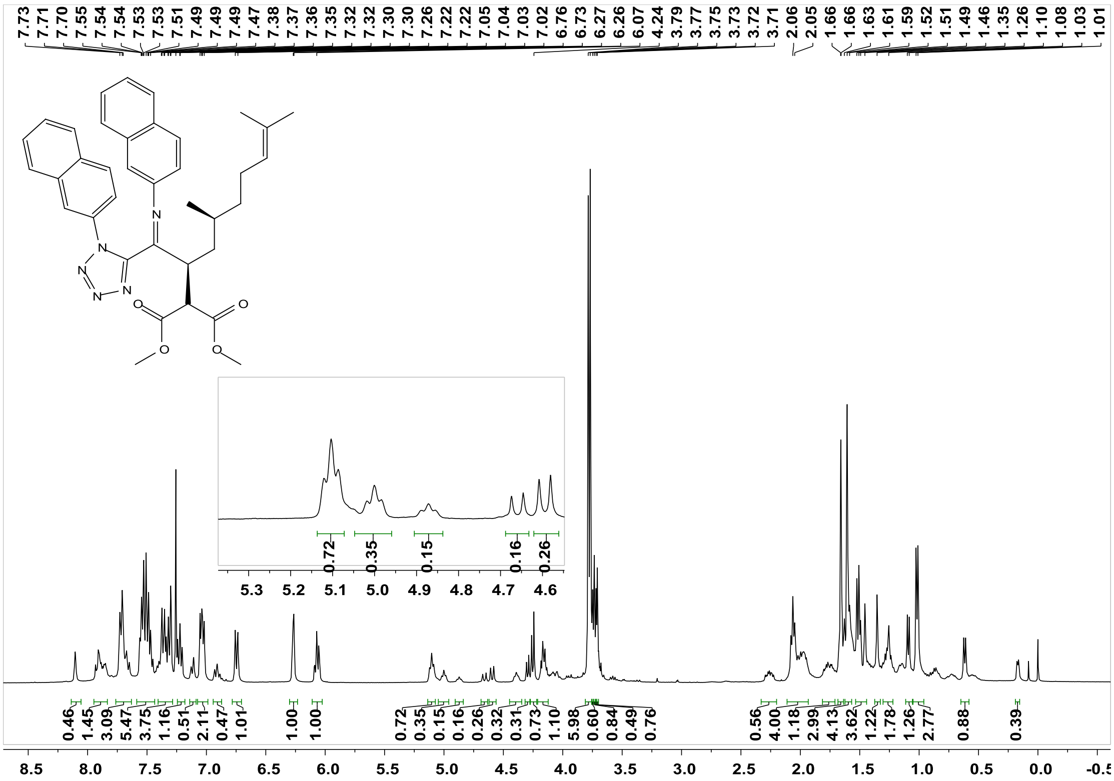

Supplementary Figure 125. ^{1}H NMR spectra for product **5y- L-RaPr$_2$**

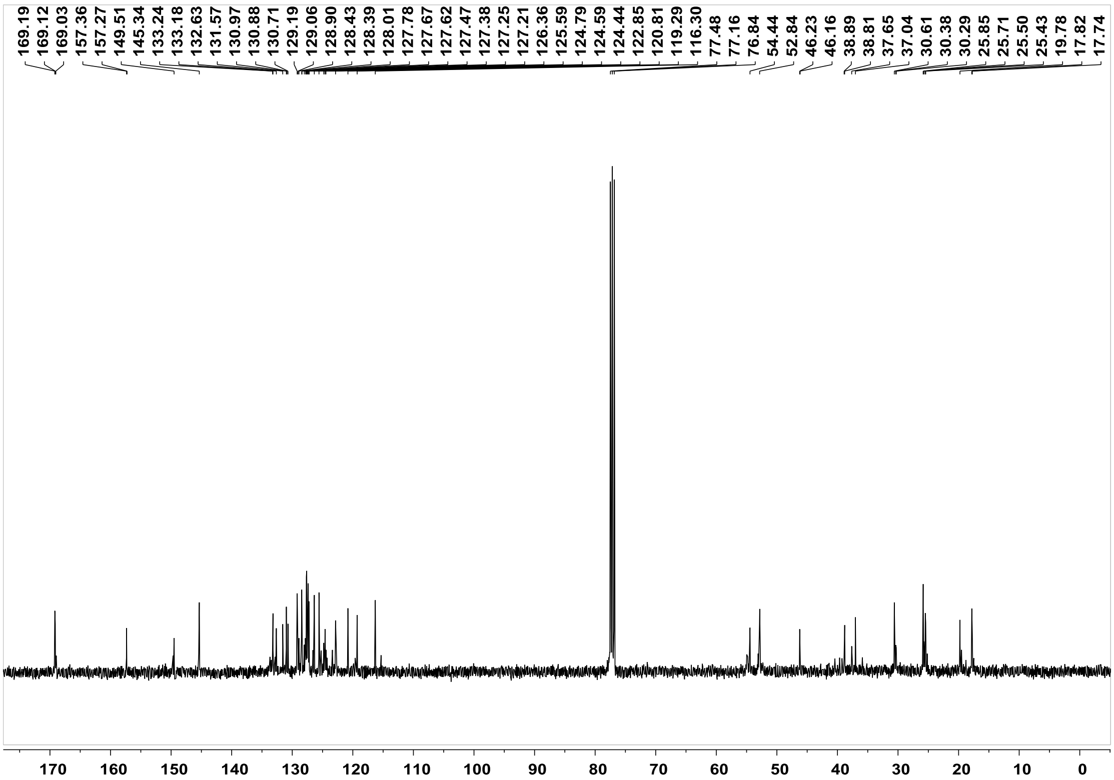

Supplementary Figure 126. ^{13}C NMR spectra for product **5y- L-RaPr$_2$**

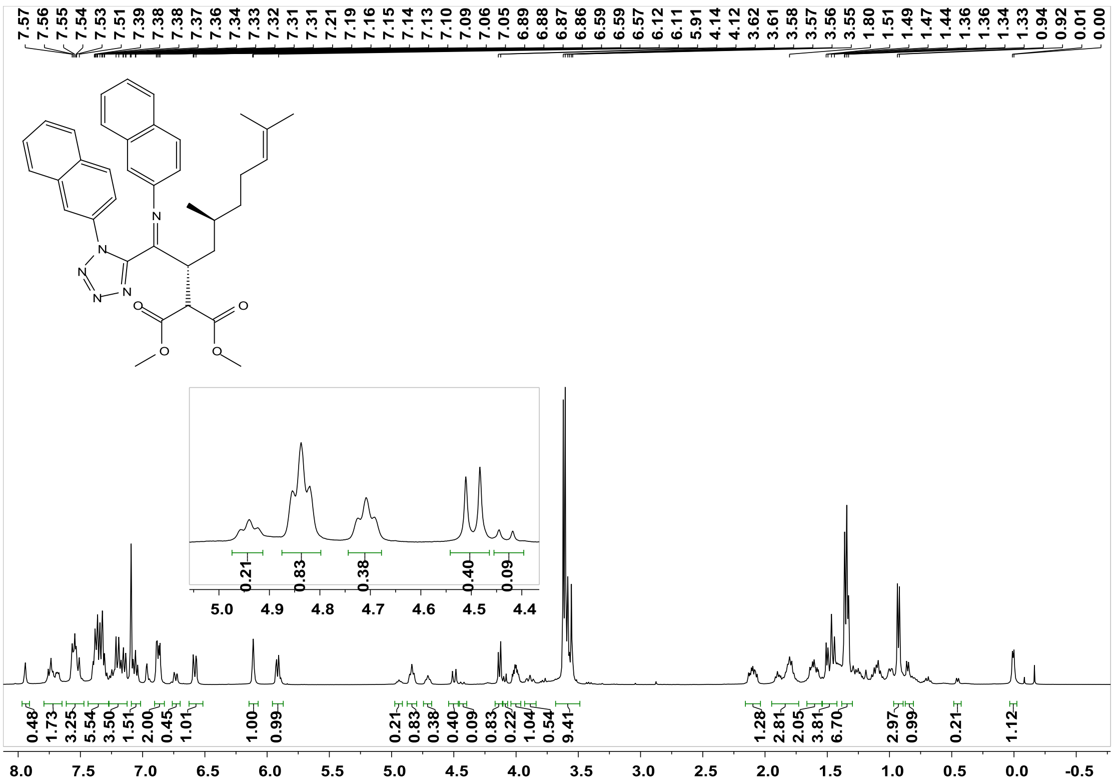

Supplementary Figure 127. ^{1}H NMR spectra for product **5y-*ent*- L-RaPr$_2$**

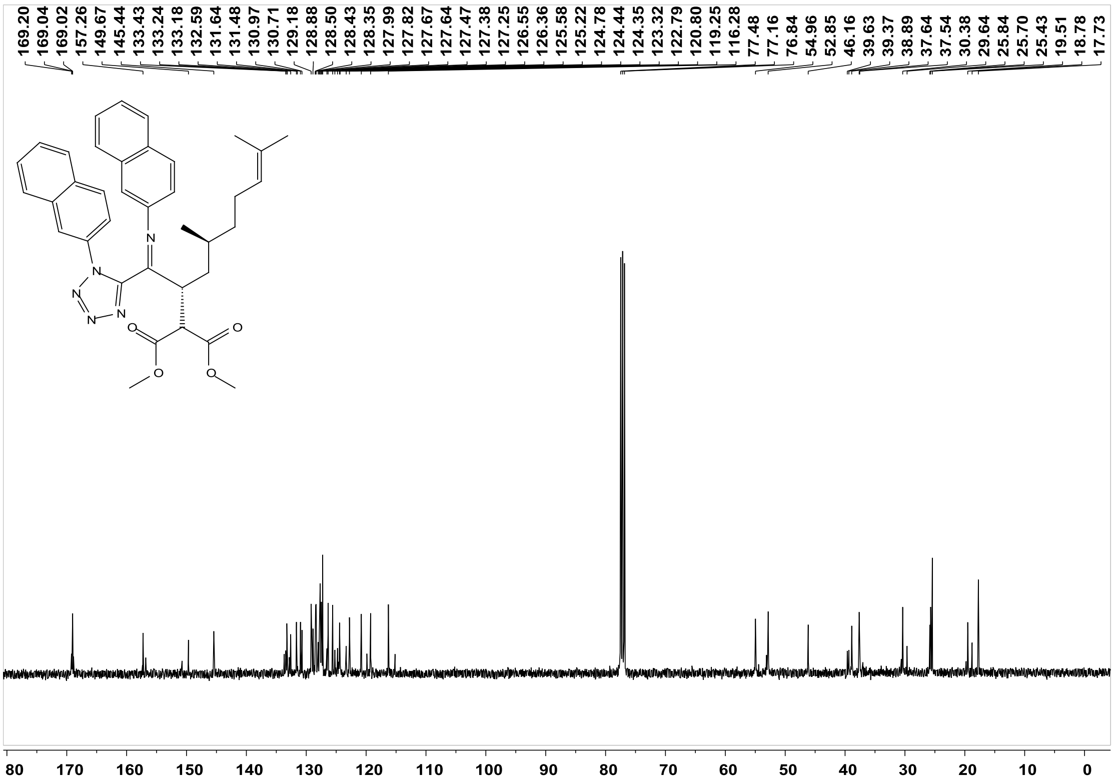

Supplementary Figure 128. ^{13}C NMR spectra for product **5y-*ent*- L-RaPr$_2$**

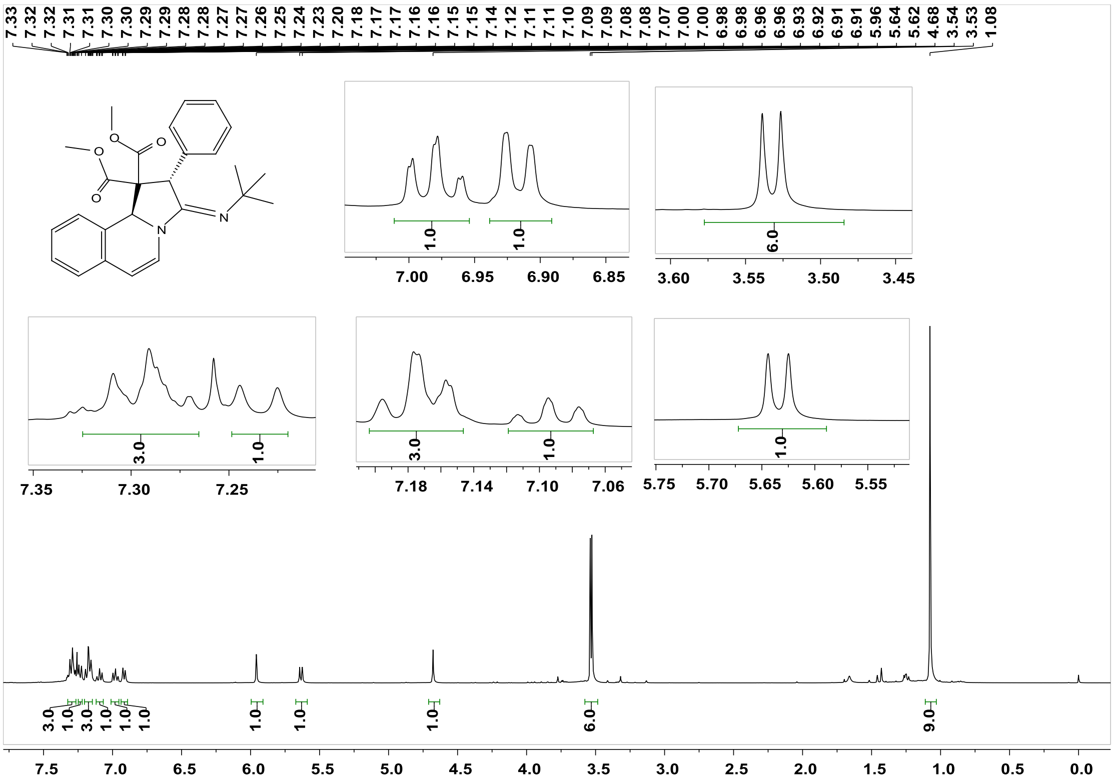

Supplementary Figure 129. ^{1}H NMR spectra for product **8a**

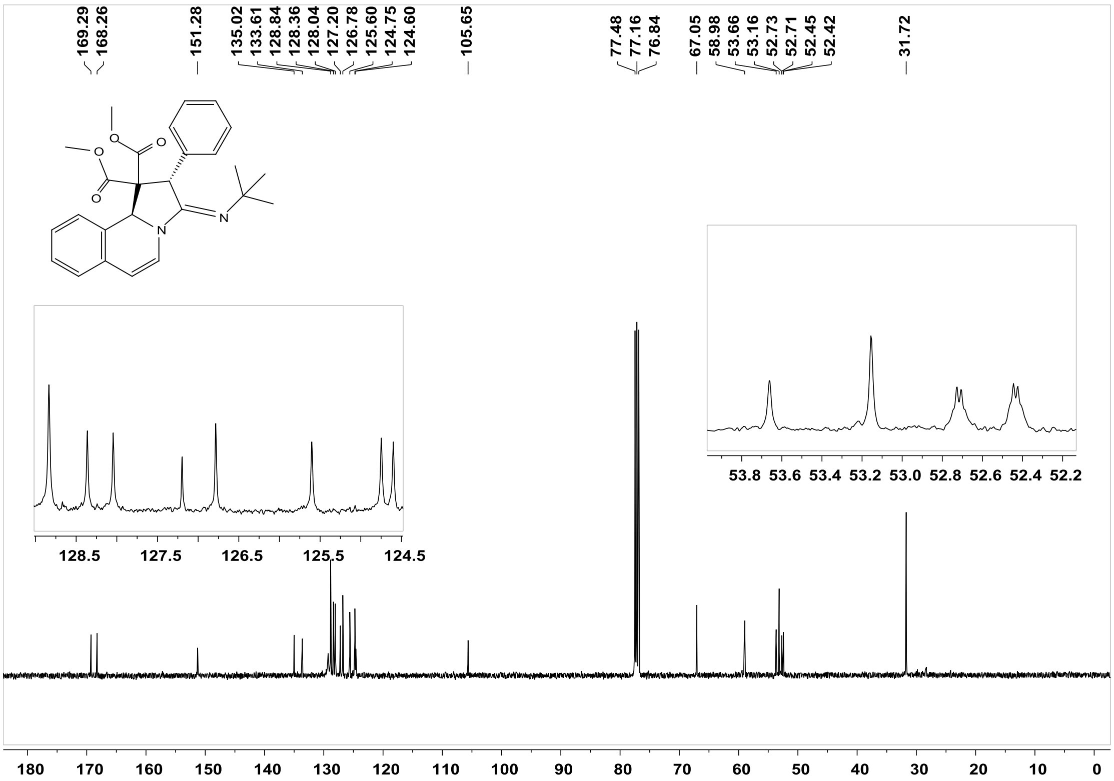

Supplementary Figure 130. ^{13}C NMR spectra for product **8a**

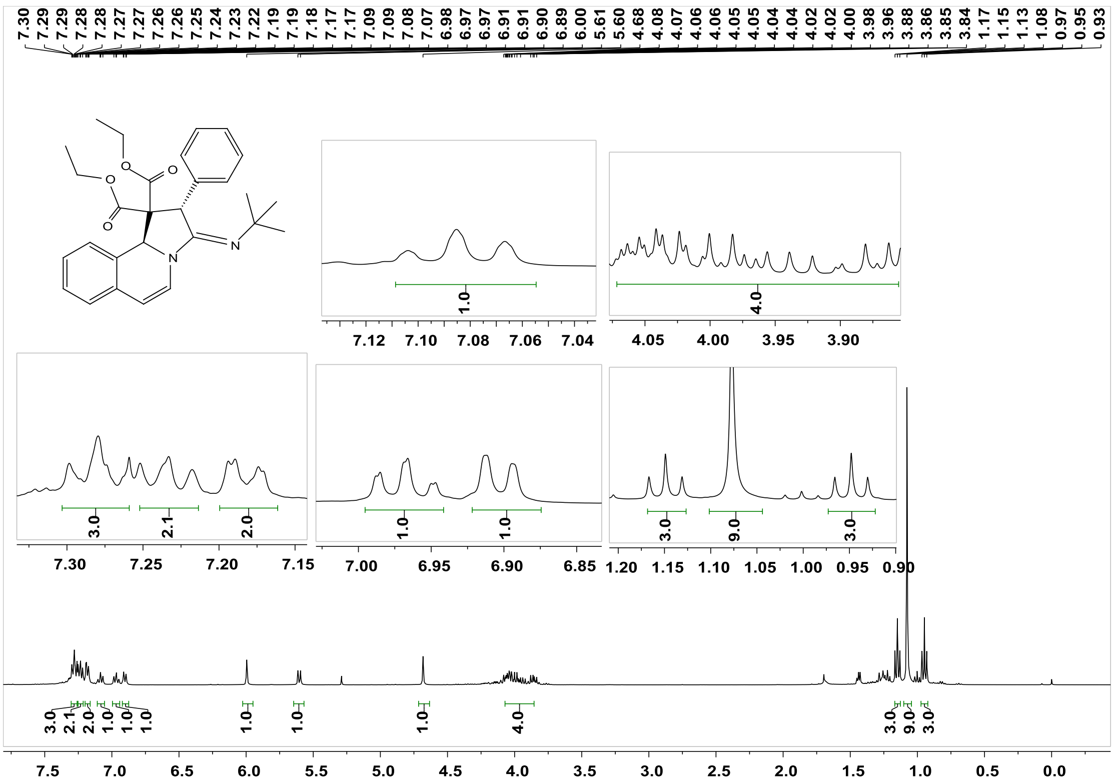

Supplementary Figure 131. ^{1}H NMR spectra for product **8b**

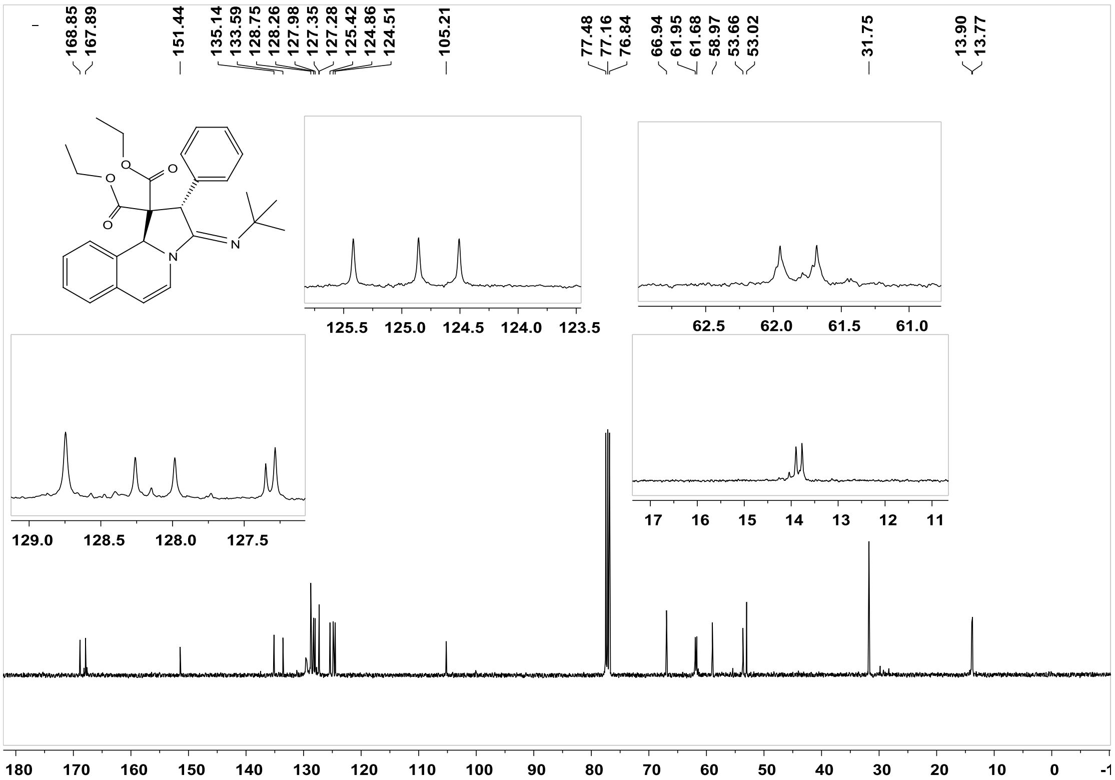

Supplementary Figure 132. ^{13}C NMR spectra for product **8b**

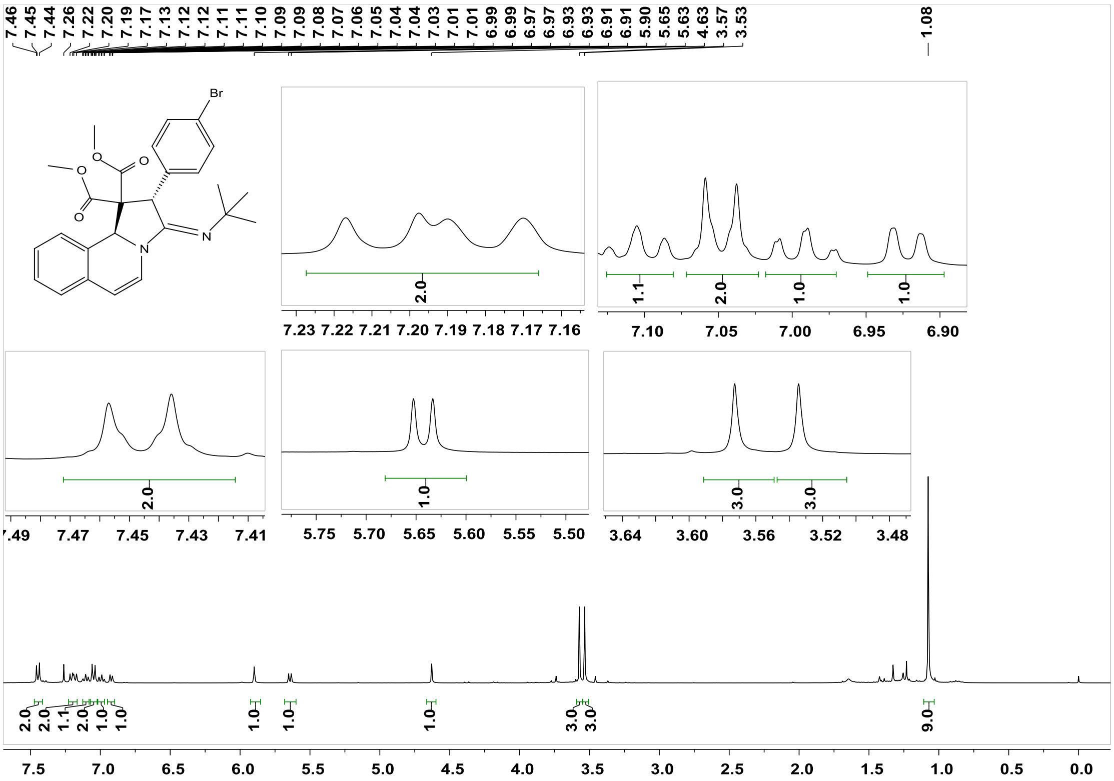

Supplementary Figure 133. ^{1}H NMR spectra for product **8c**

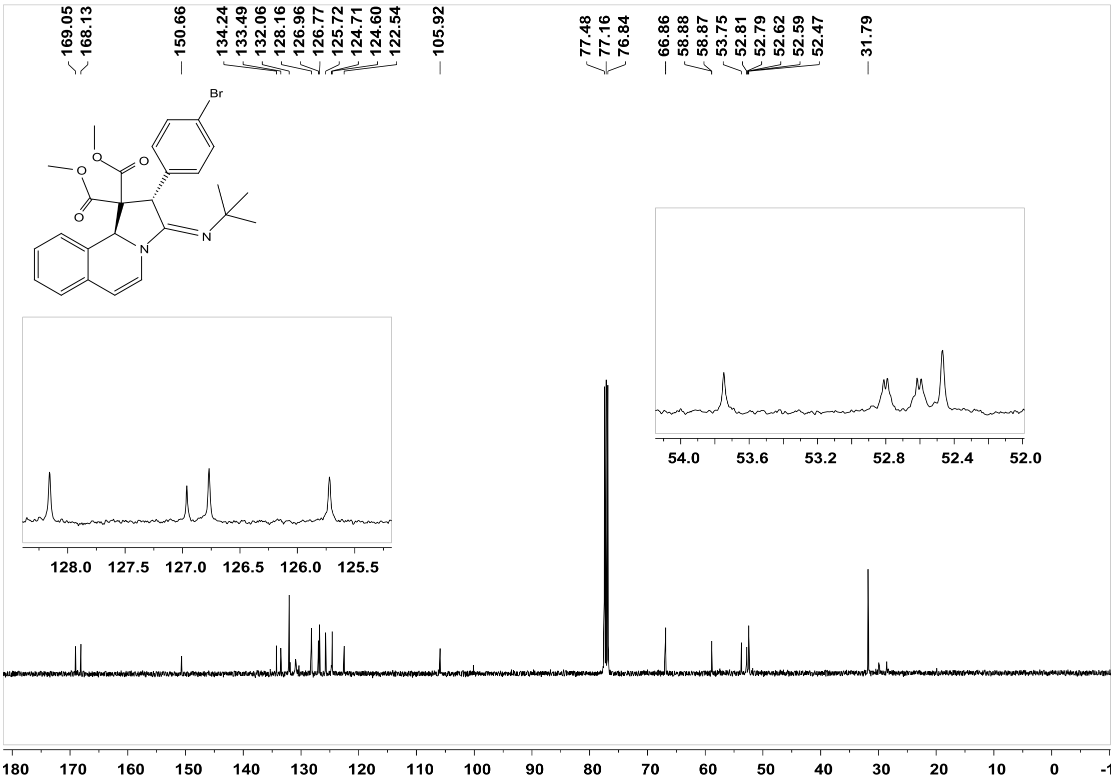

Supplementary Figure 134. ^{13}C NMR spectra for product **8c**

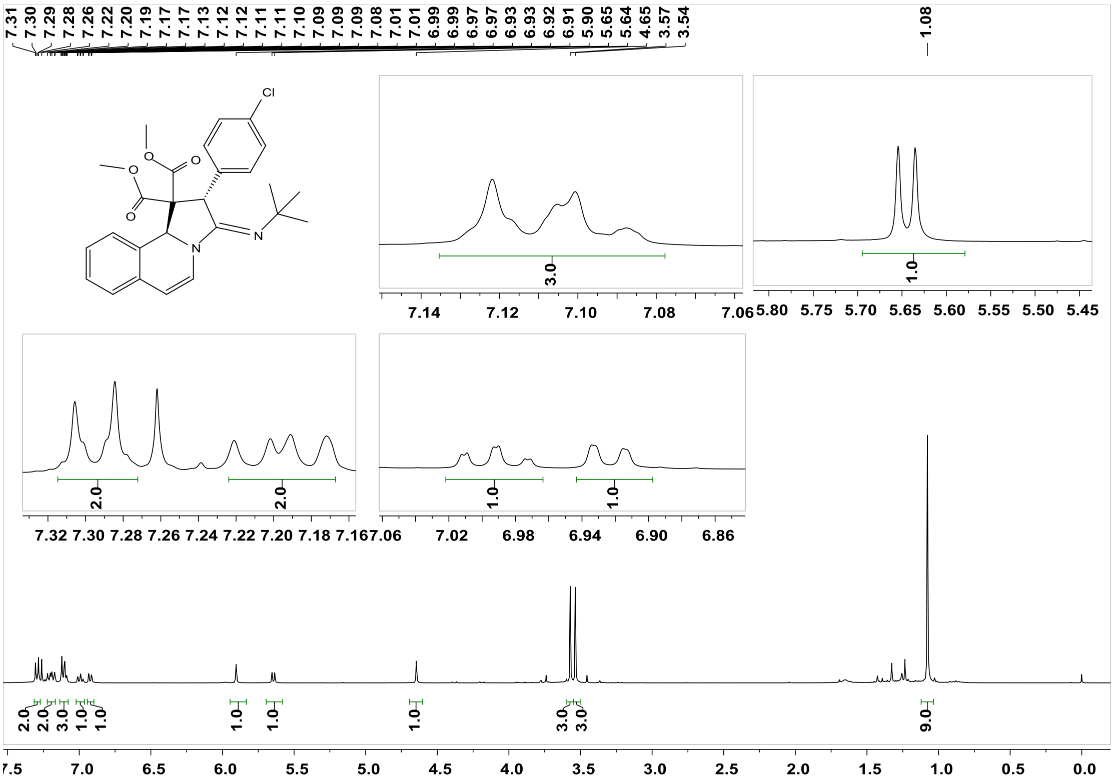

Supplementary Figure 135. ^{1}H NMR spectra for product **8d**

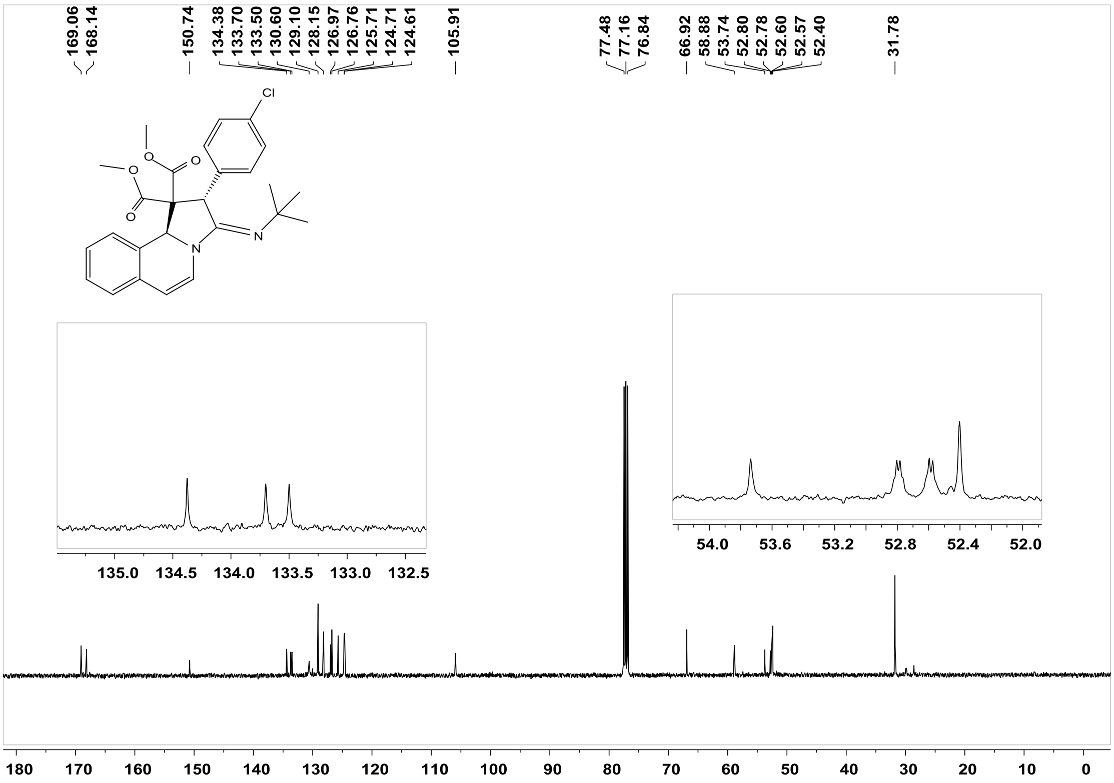

Supplementary Figure 136. ^{13}C NMR spectra for product **8d**

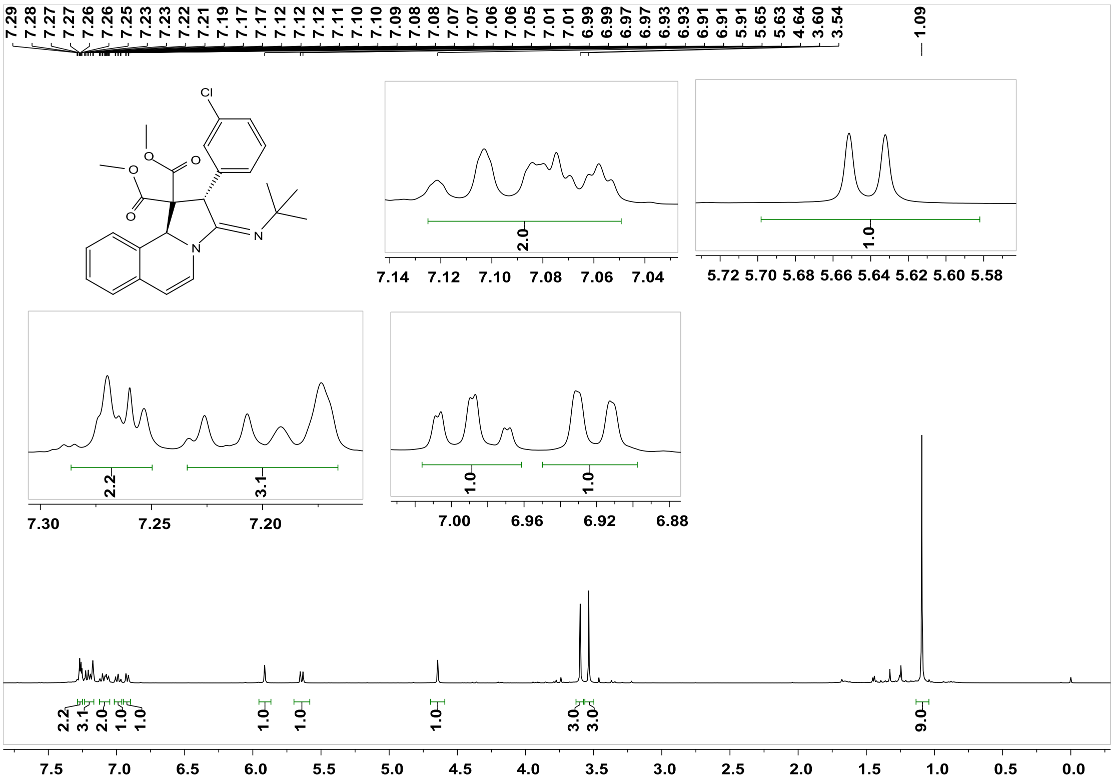

Supplementary Figure 137. ^{1}H NMR spectra for product **8e**

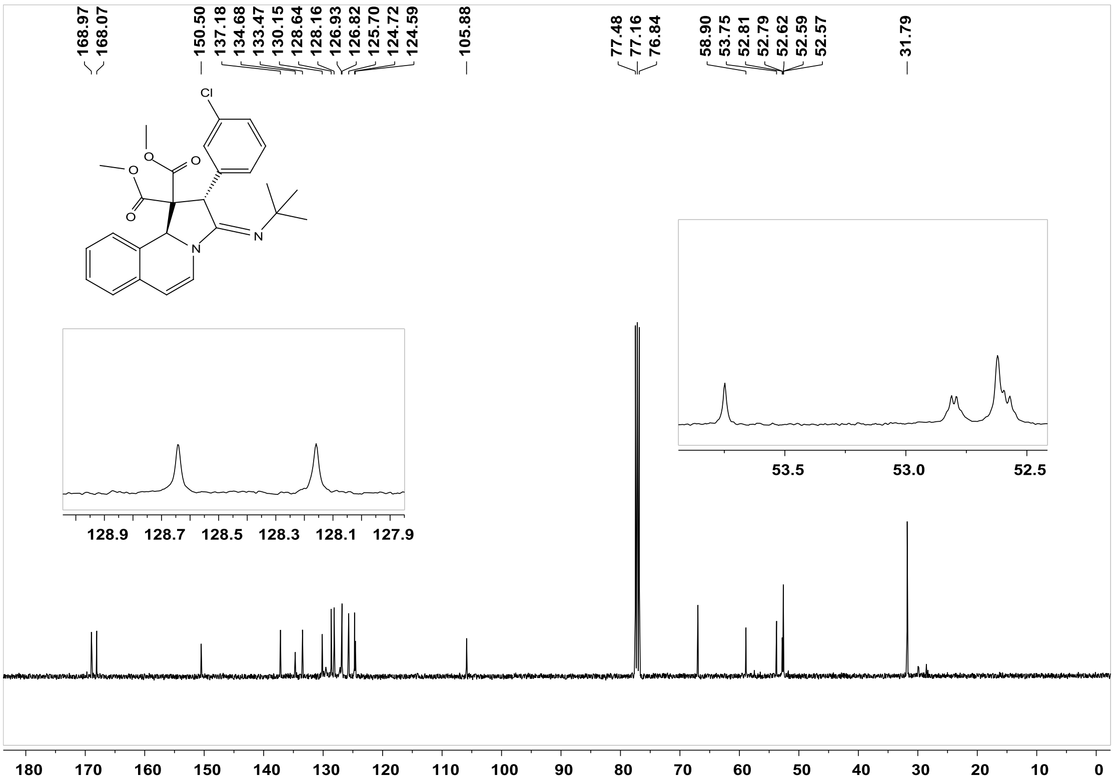

Supplementary Figure 138. ^{13}C NMR spectra for product **8e**

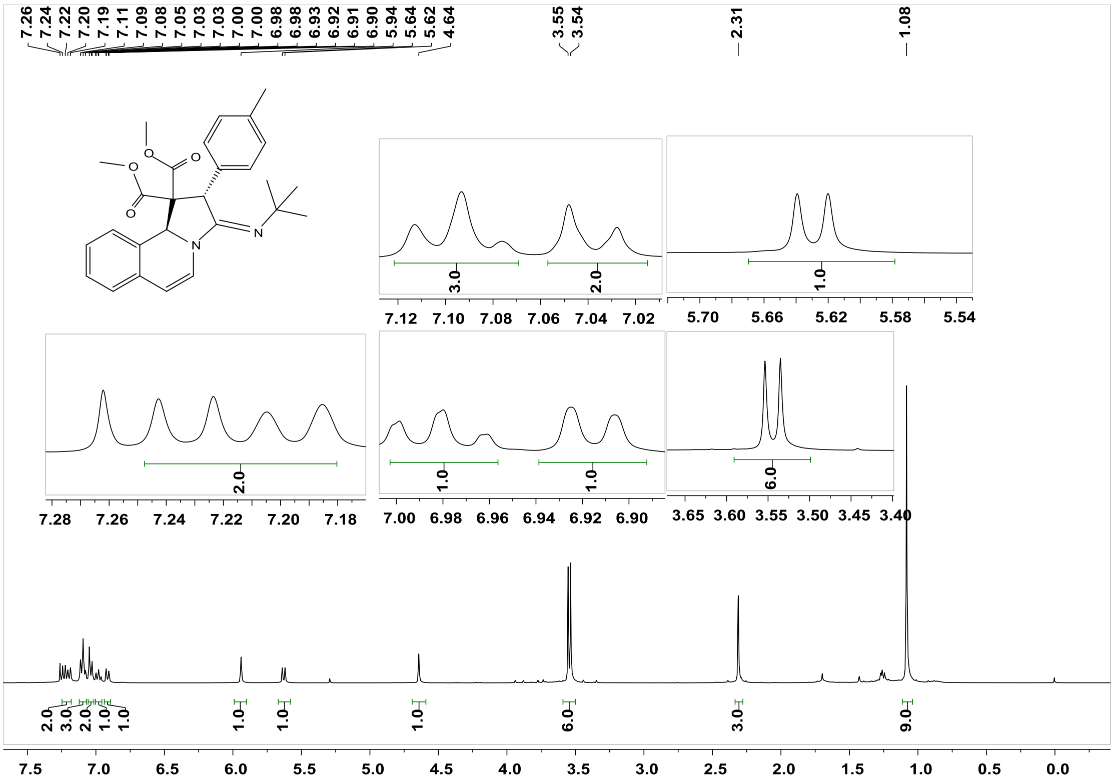

Supplementary Figure 139. ^{1}H NMR spectra for product **8f**

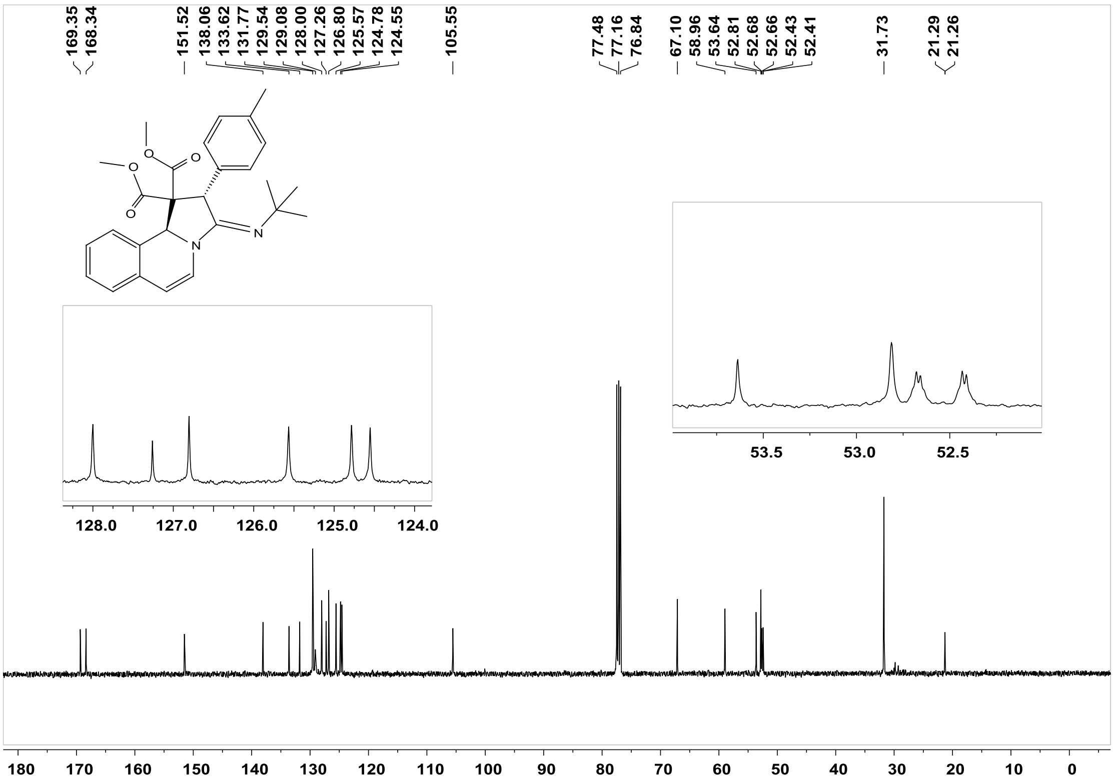

Supplementary Figure 140. ^{13}C NMR spectra for product **8f**

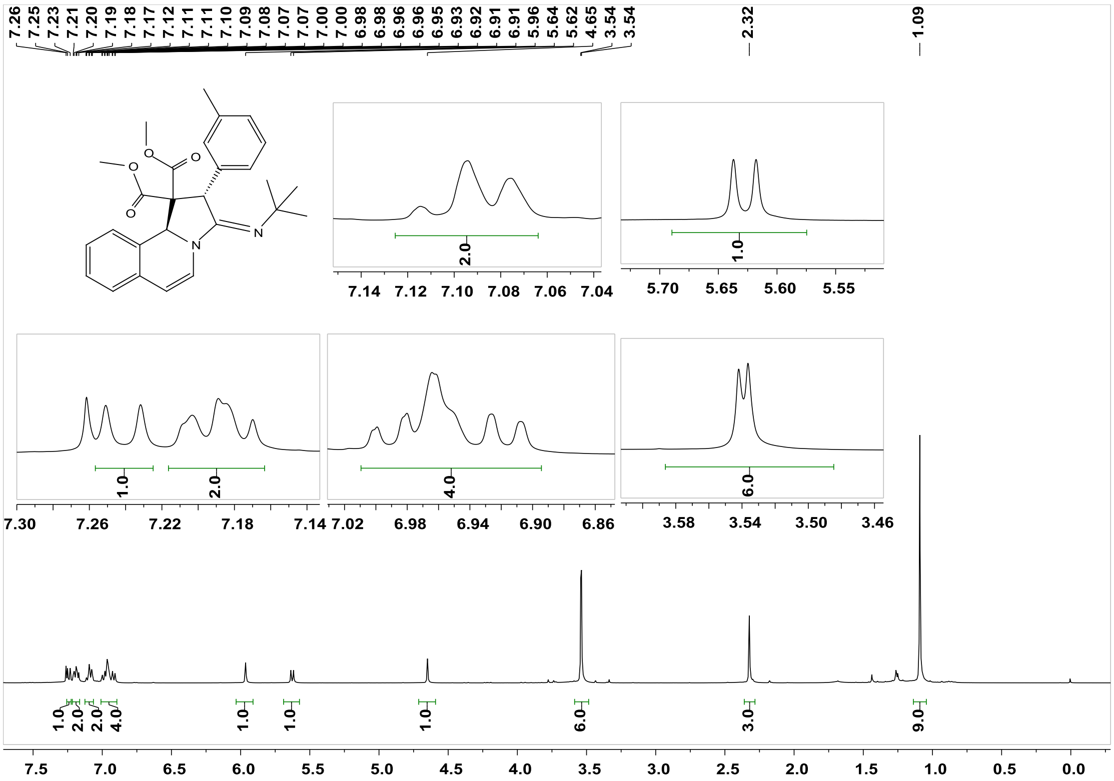

Supplementary Figure 141. ^{1}H NMR spectra for product **8g**

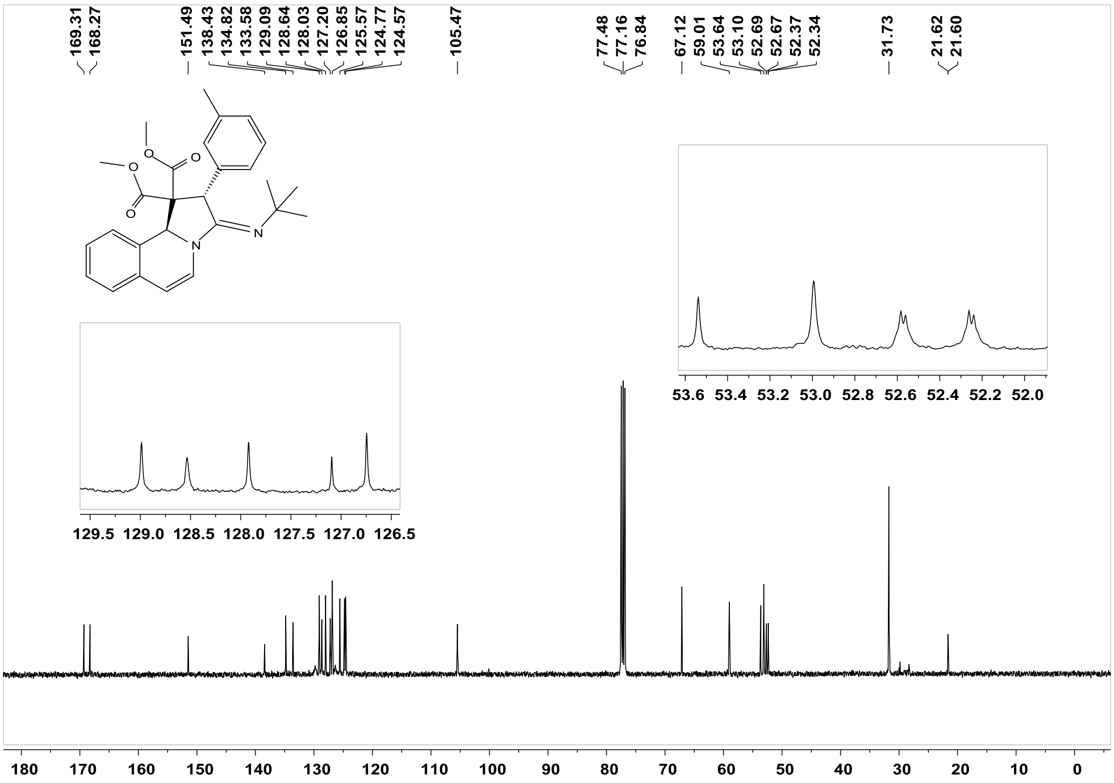

Supplementary Figure 142. ^{13}C NMR spectra for product **8g**

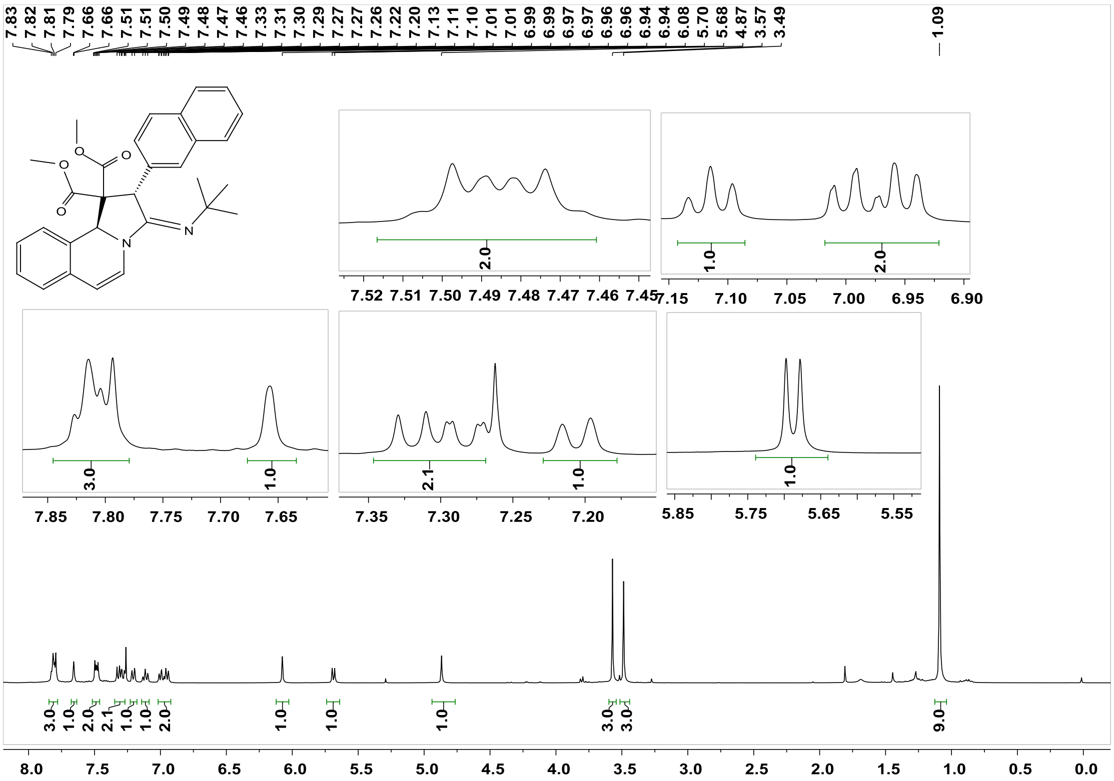

Supplementary Figure 143. ^{1}H NMR spectra for product **8h**

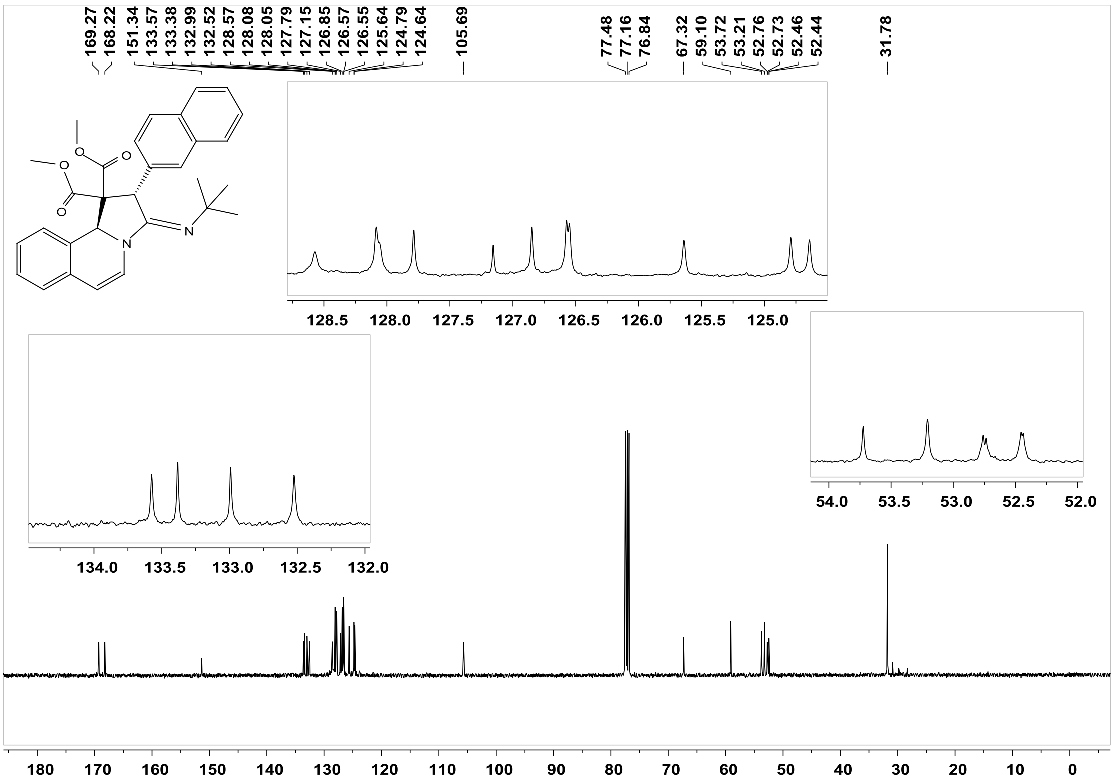

Supplementary Figure 144. ^{13}C NMR spectra for product **8h**

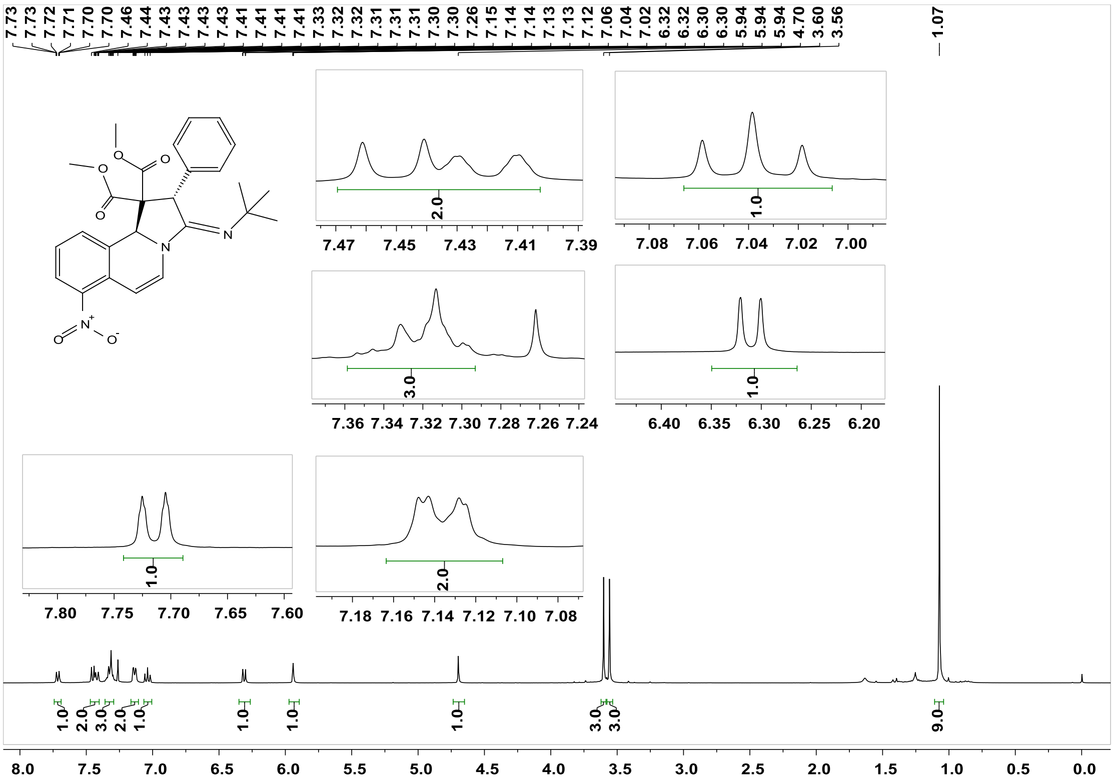

Supplementary Figure 145. ^{1}H NMR spectra for product **8i**

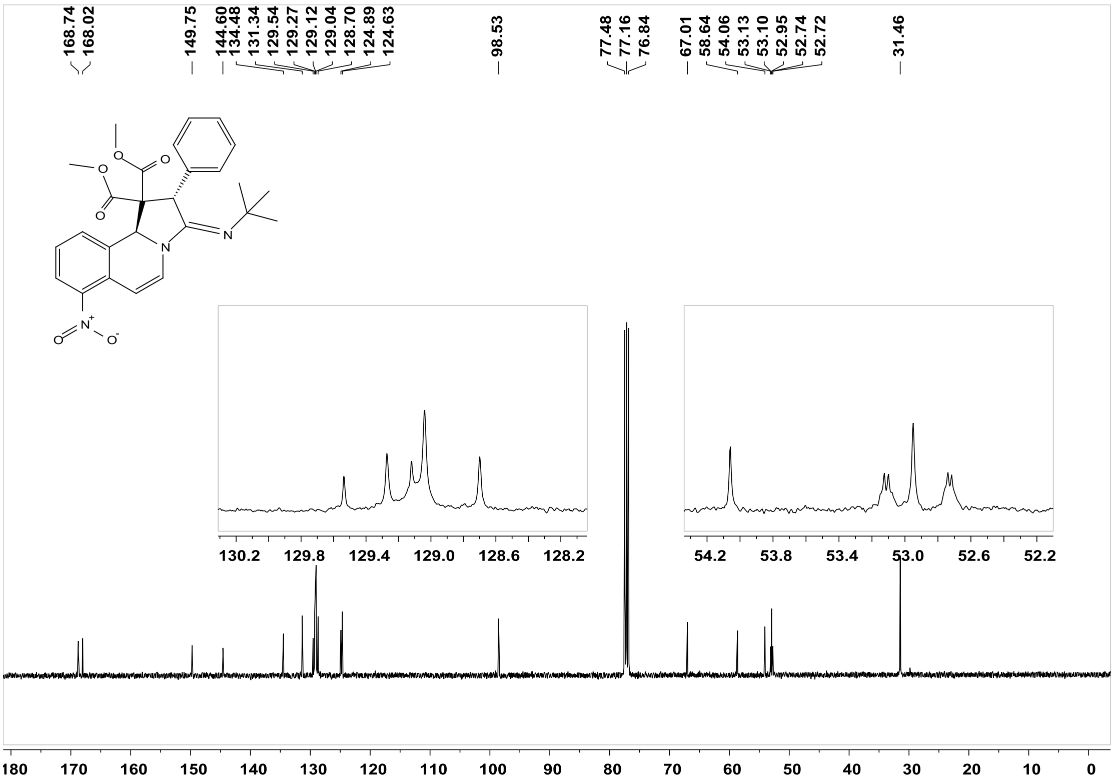

Supplementary Figure 146. ^{13}C NMR spectra for product **8i**

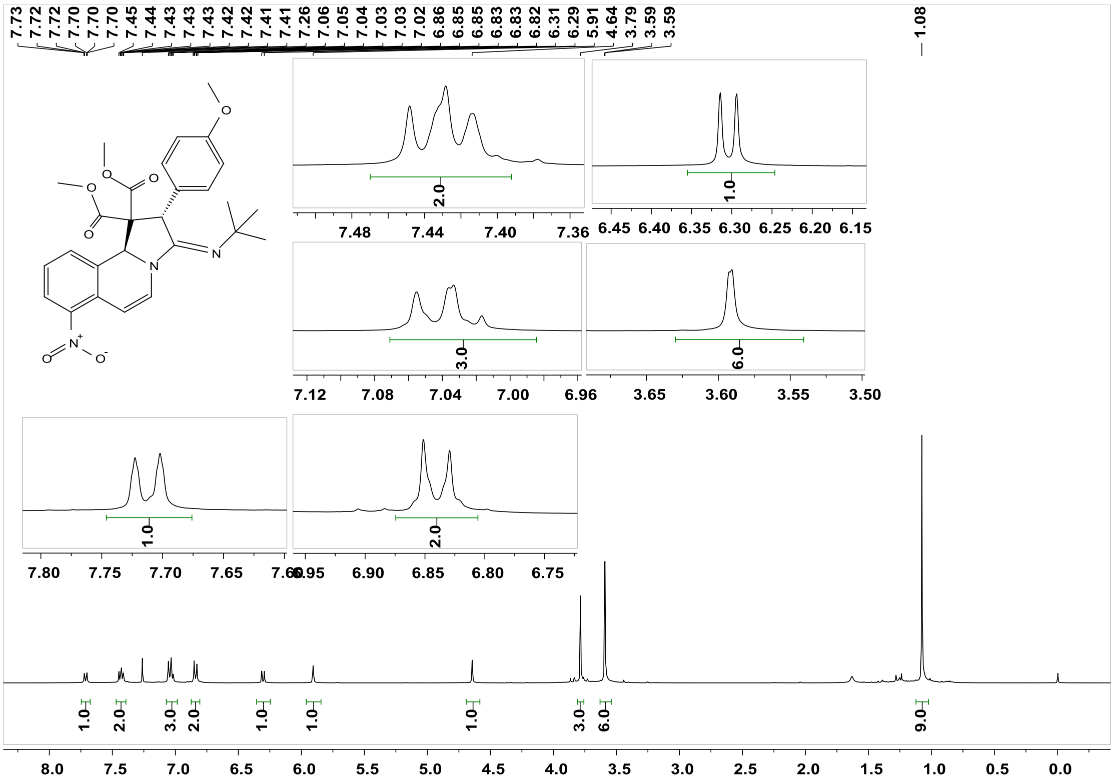

Supplementary Figure 147. ^{1}H NMR spectra for product **8j**

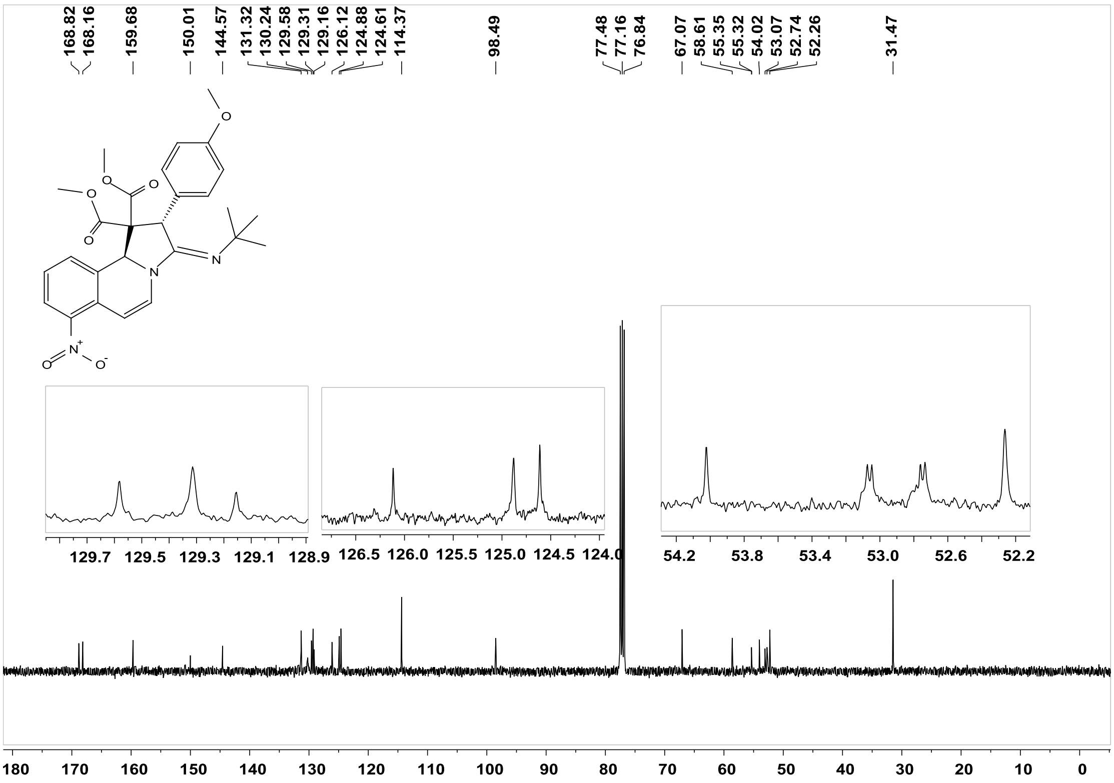

Supplementary Figure 148. ^{13}C NMR spectra for product **8j**

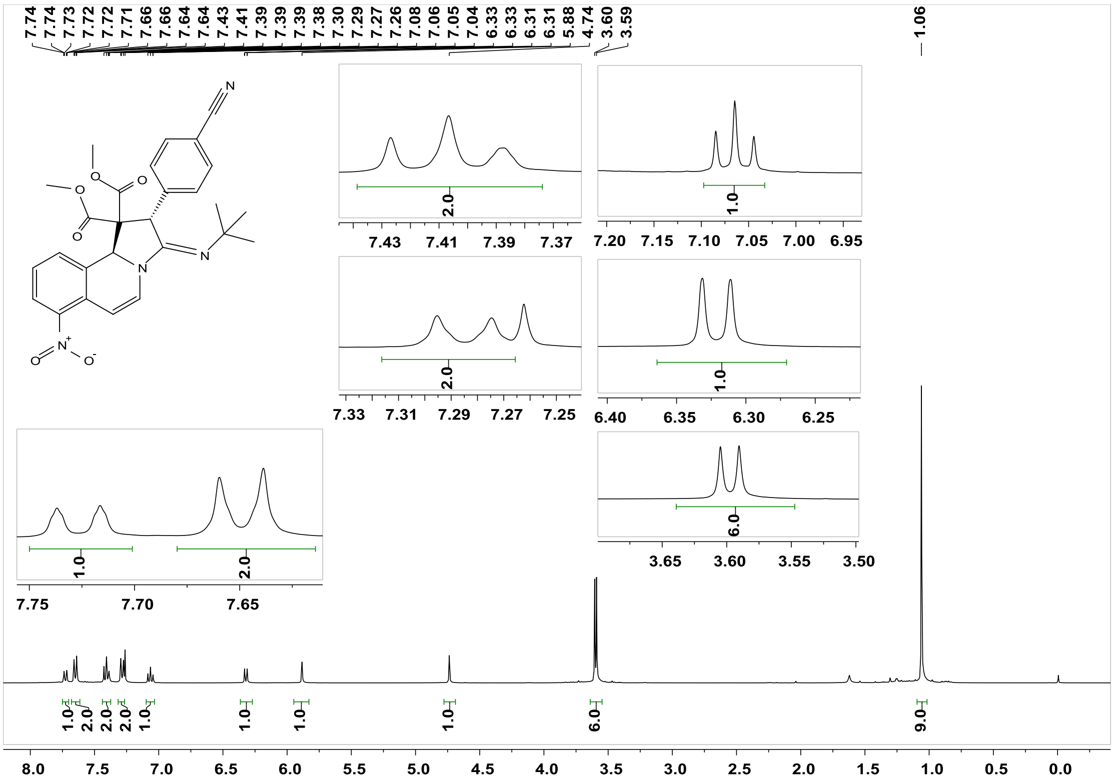

Supplementary Figure 149. ^{1}H NMR spectra for product **8k**

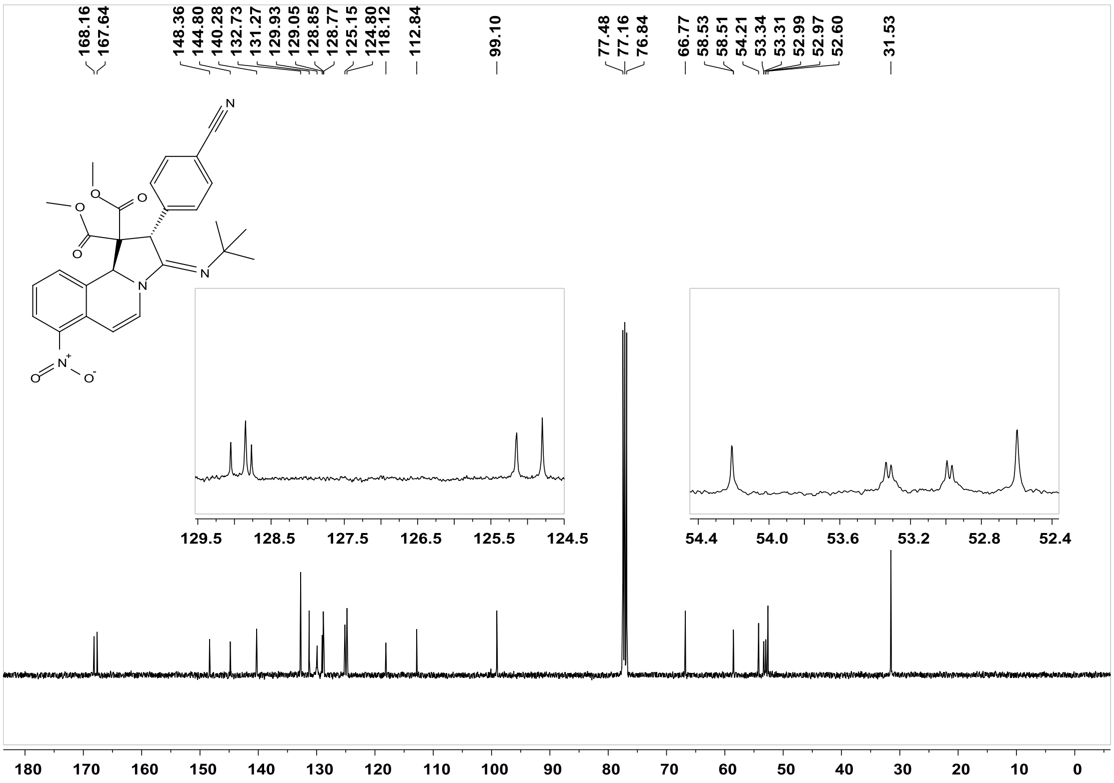

Supplementary Figure 150. ^{13}C NMR spectra for product **8k**

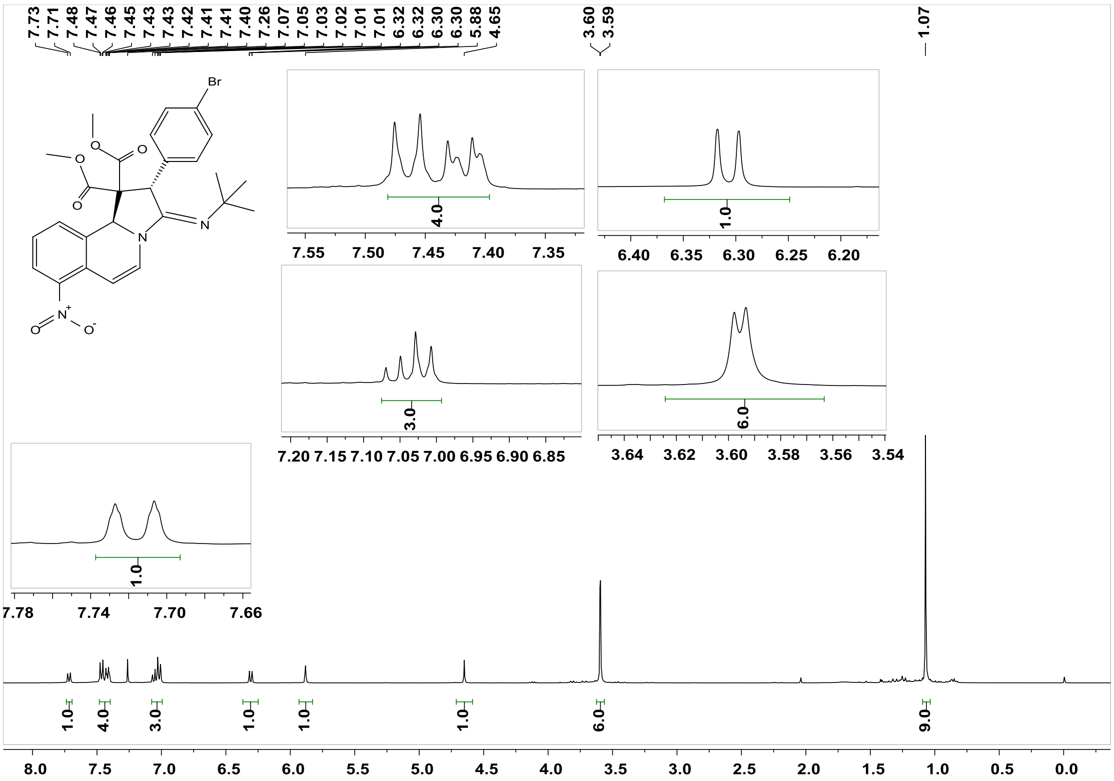

Supplementary Figure 151. ^{1}H NMR spectra for product **8l**

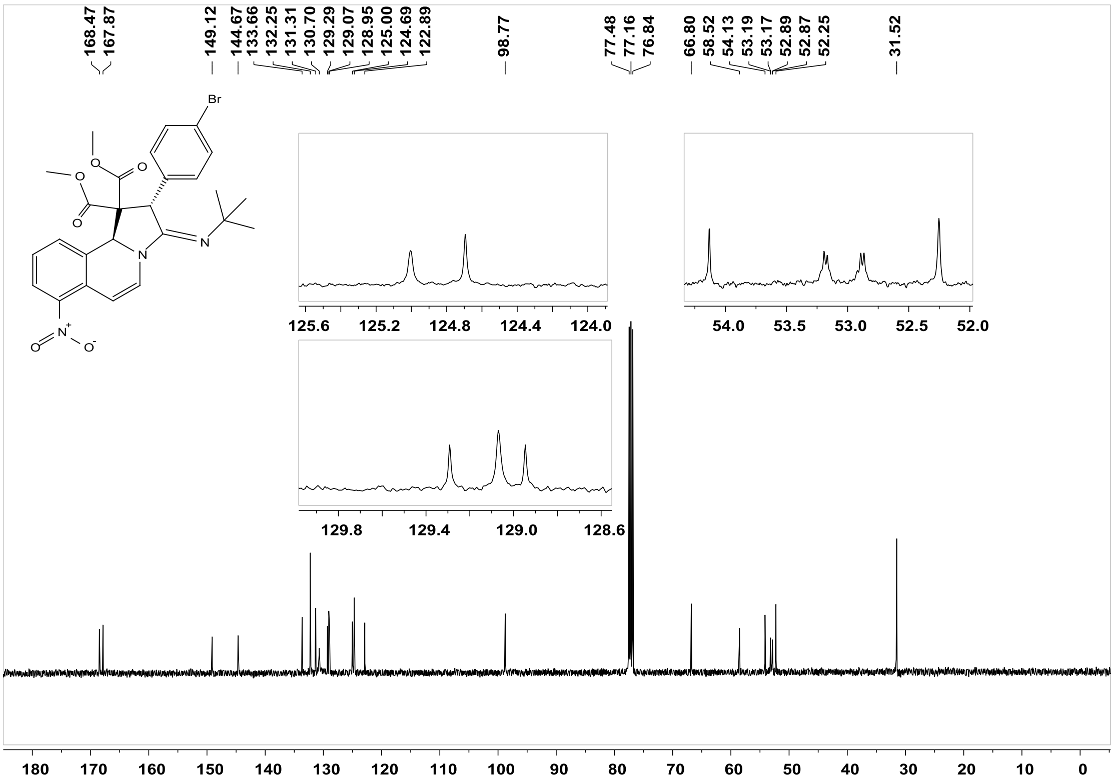

Supplementary Figure 152. ^{13}C NMR spectra for product **8l**

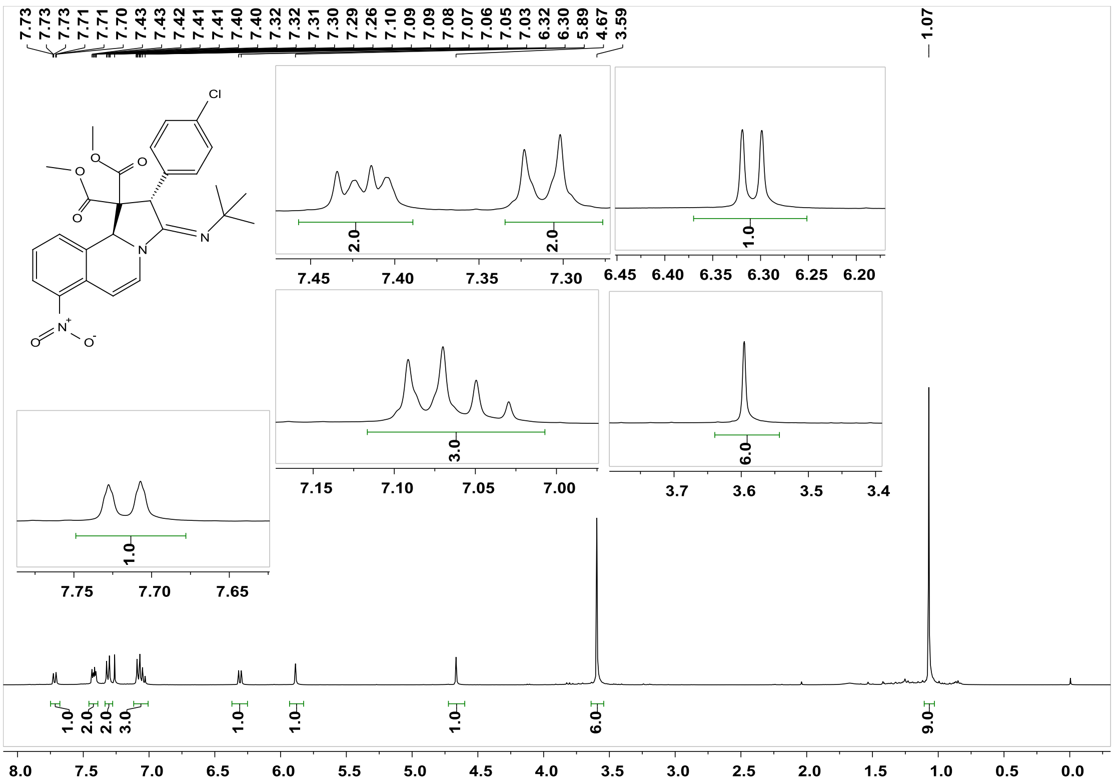

Supplementary Figure 153. ^{1}H NMR spectra for product **8m**

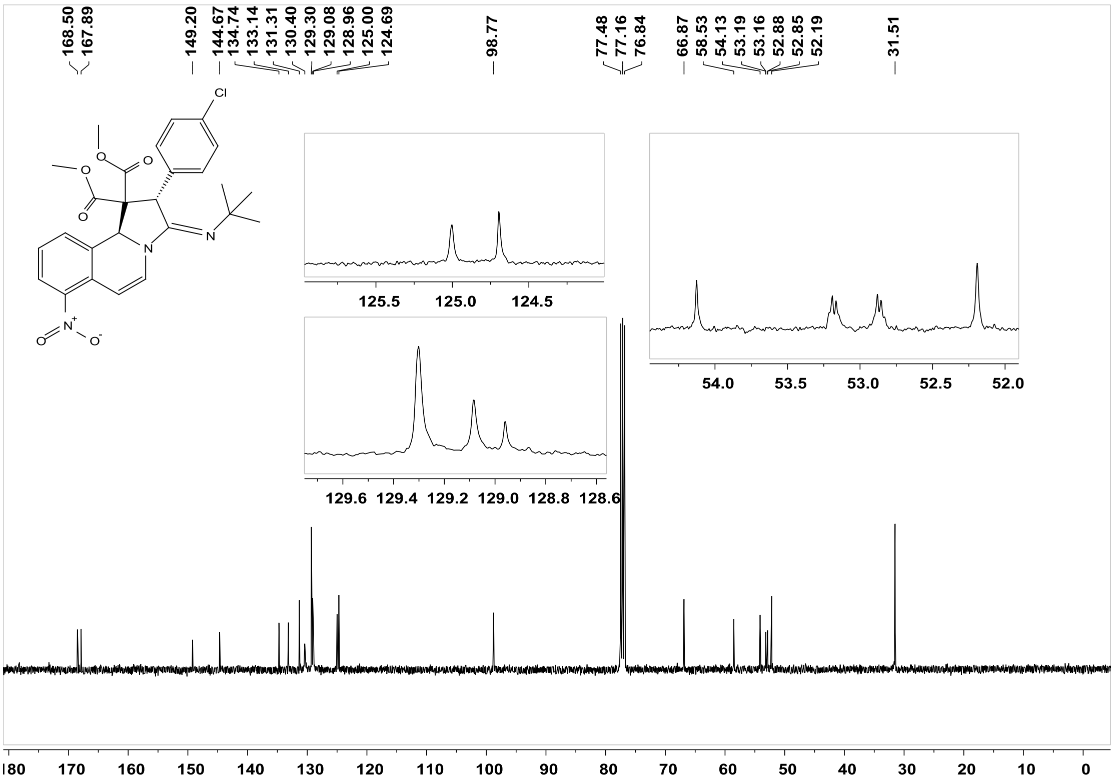

Supplementary Figure 154. ^{13}C NMR spectra for product **8m**

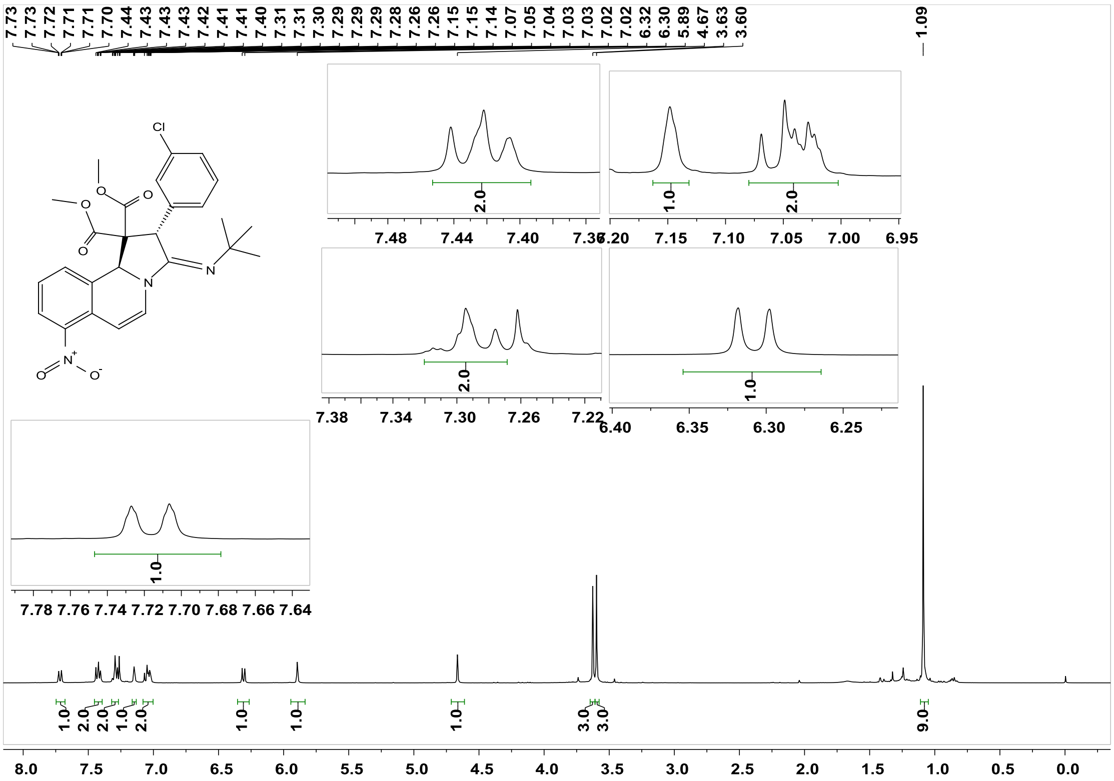

Supplementary Figure 155. ^{1}H NMR spectra for product **8n**

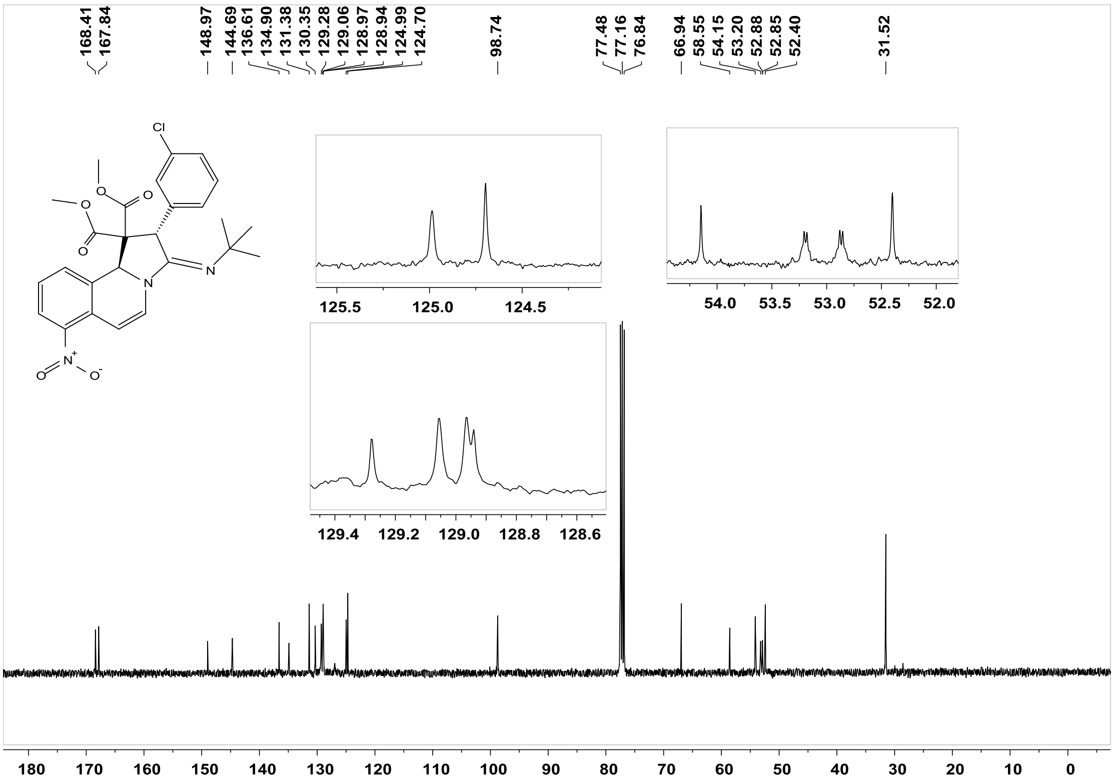

Supplementary Figure 156. ^{13}CNMR spectra for product **8n**

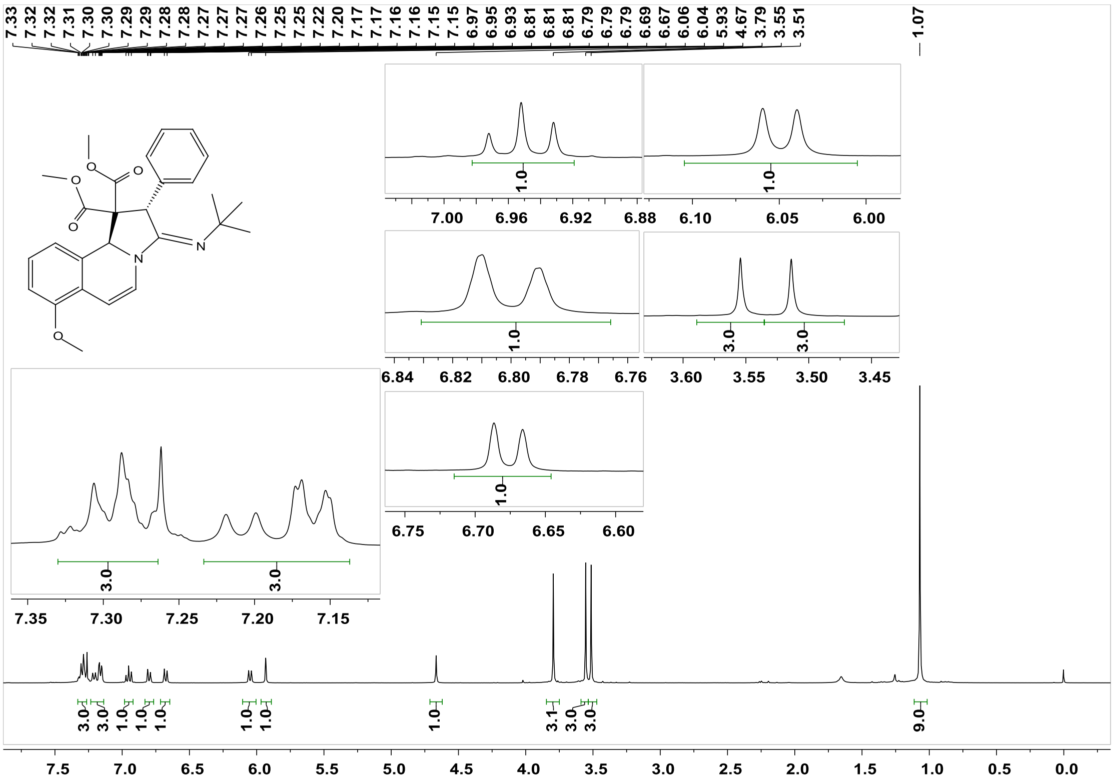

Supplementary Figure 157. ^{1}H NMR spectra for product **8o**

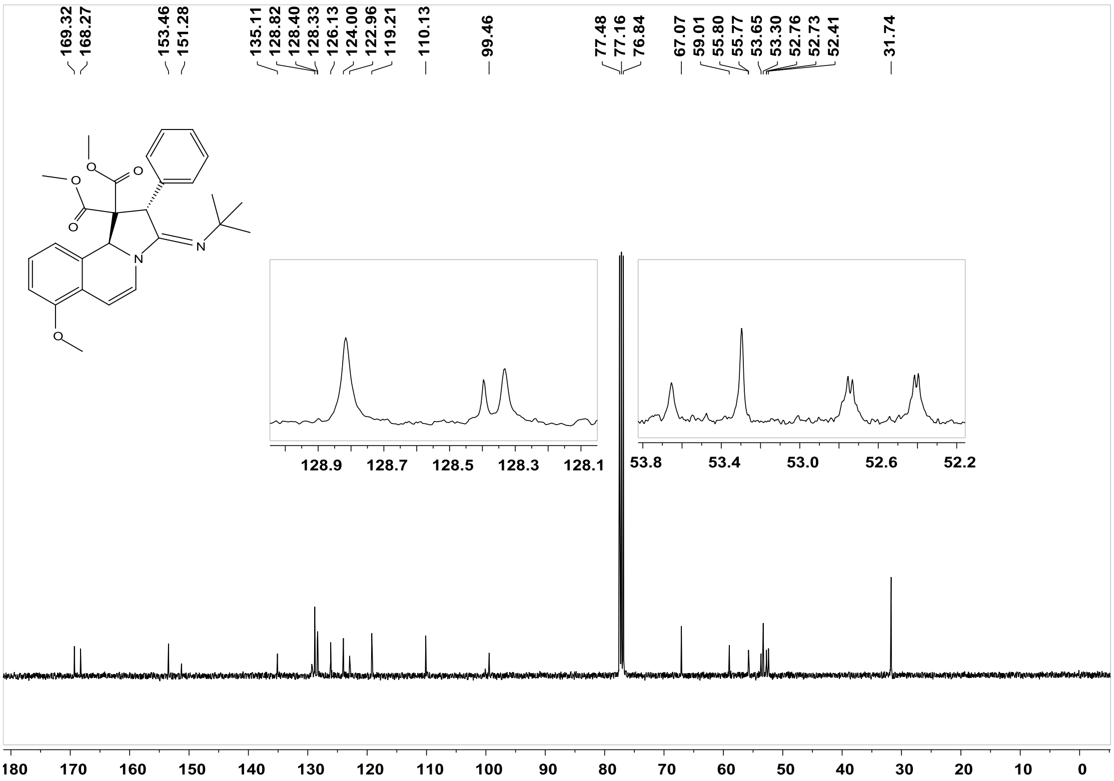

Supplementary Figure 158. ^{13}C NMR spectra for product **8o**

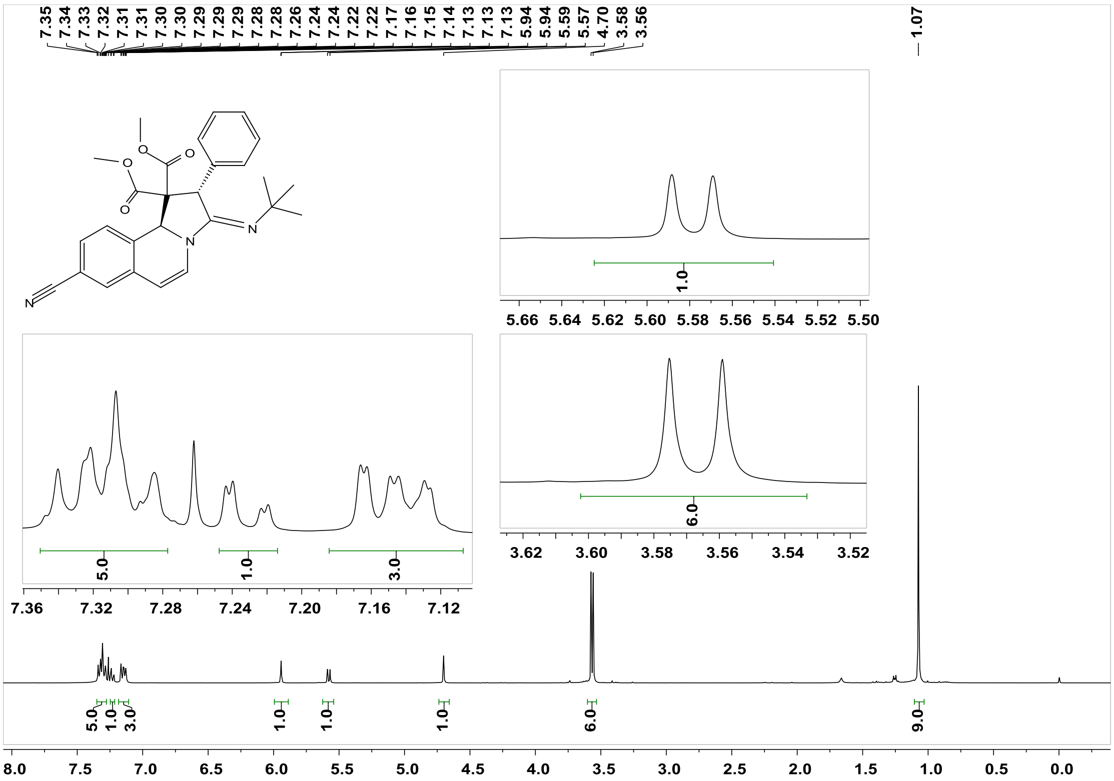

Supplementary Figure 159. ^{1}H NMR spectra for product **8p**

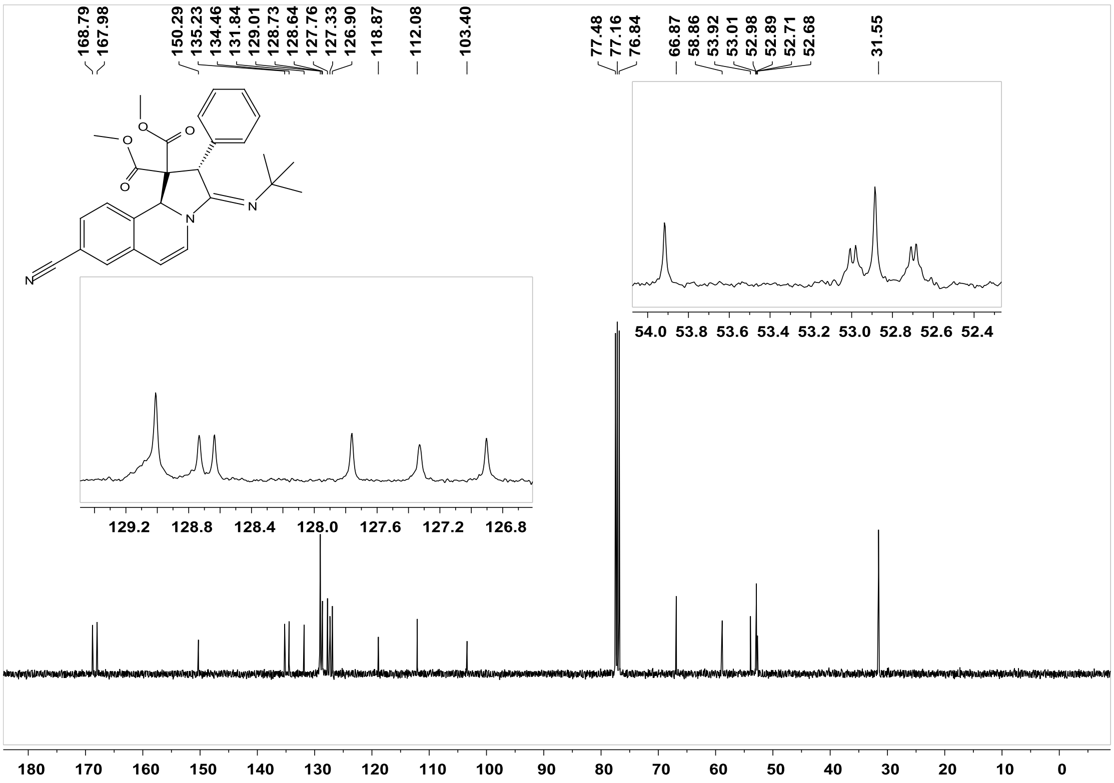

Supplementary Figure 160. ^{13}C NMR spectra for product **8p**

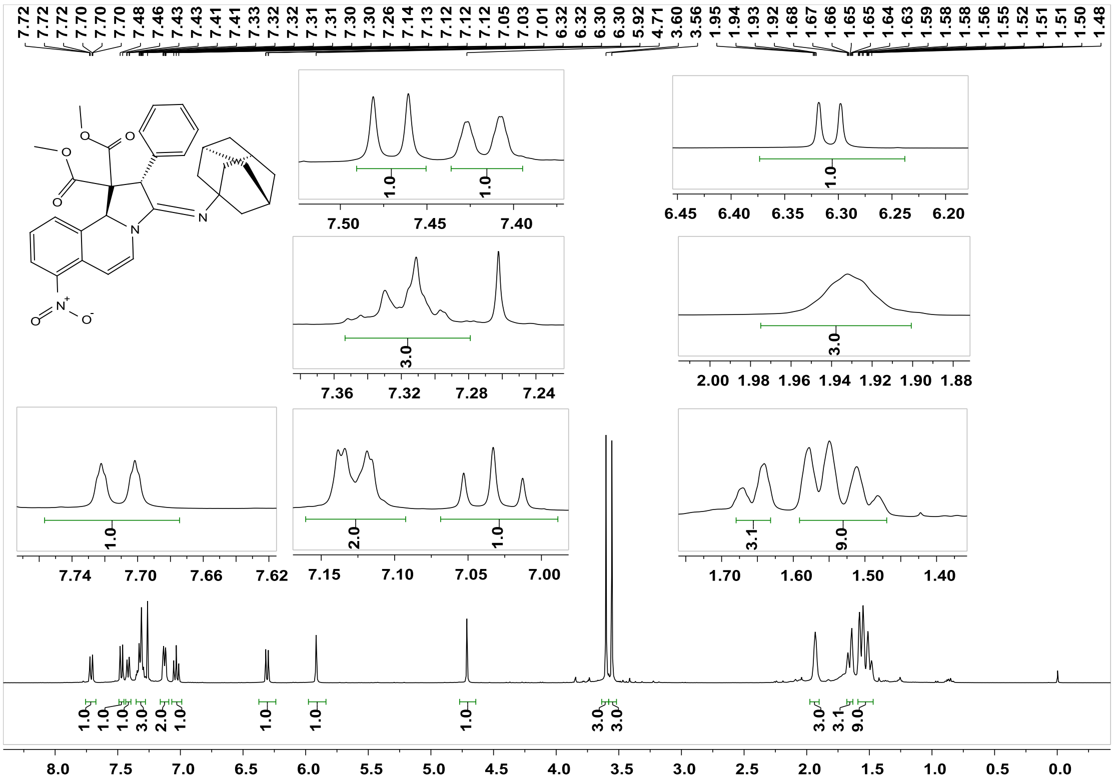

Supplementary Figure 161. ^{1}H NMR spectra for product **8q**

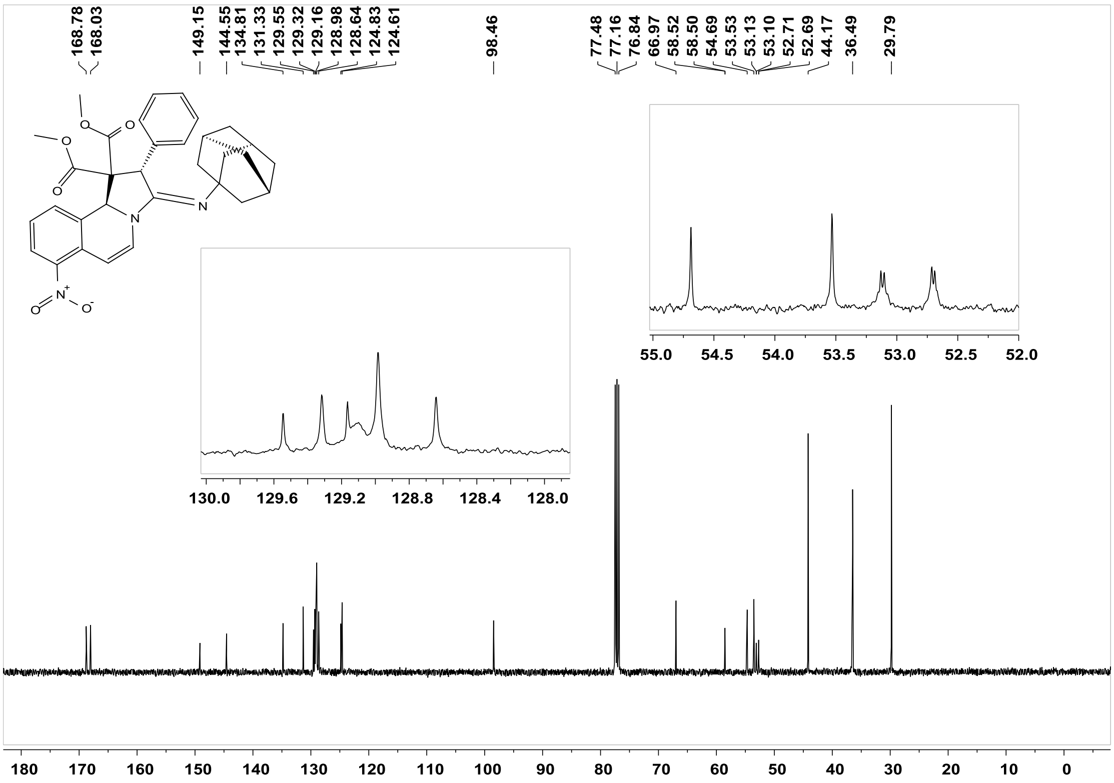

Supplementary Figure 162. ^{13}C NMR spectra for product **8q**

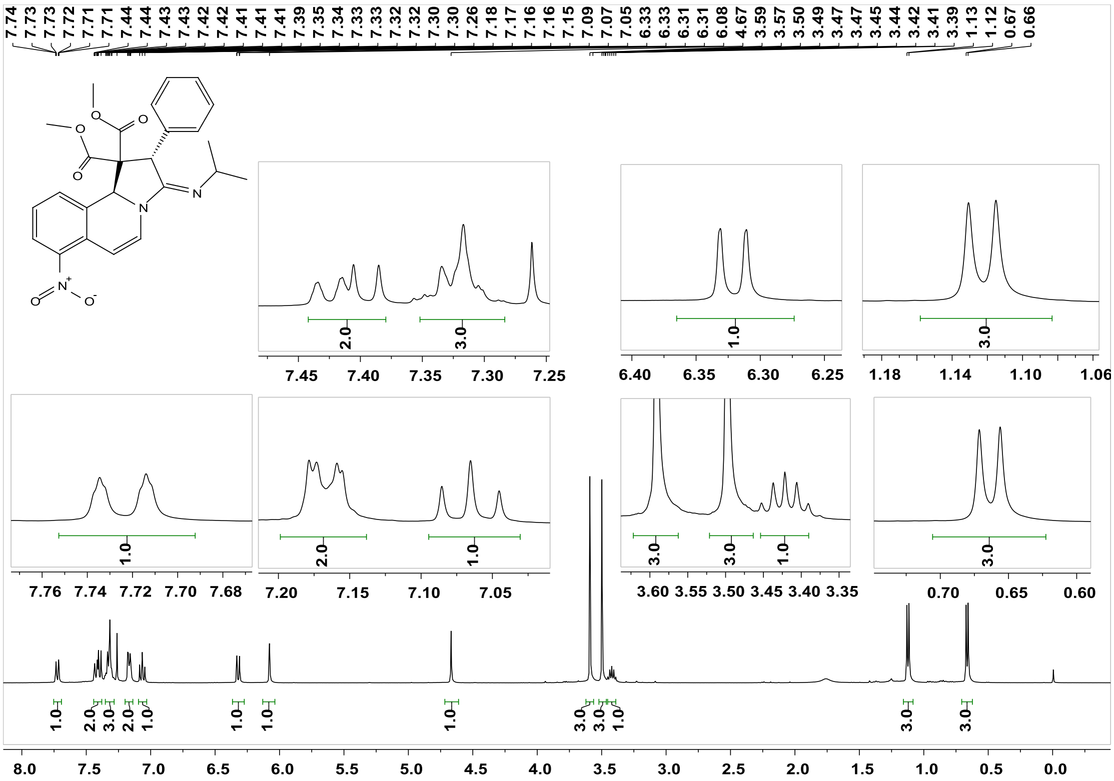

Supplementary Figure 163. ^{1}H NMR spectra for product **8r**

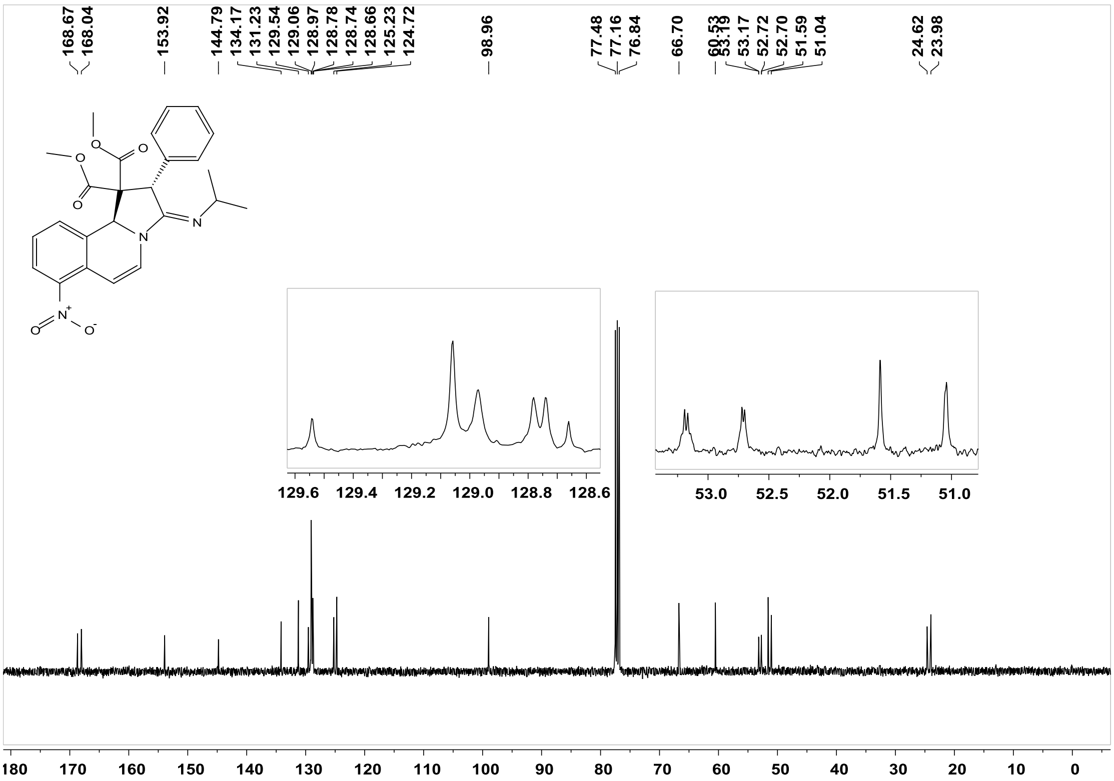

Supplementary Figure 164. ^{13}C NMR spectra for product **8r**

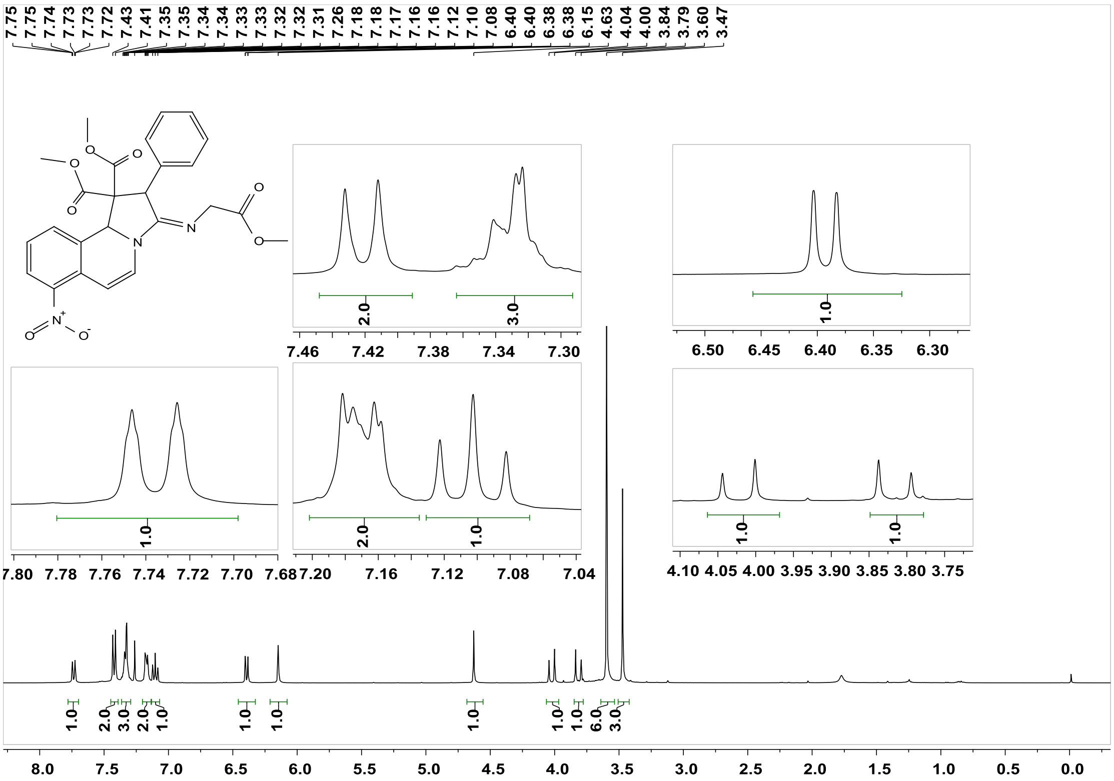

Supplementary Figure 165. ^{1}H NMR spectra for product **8s**

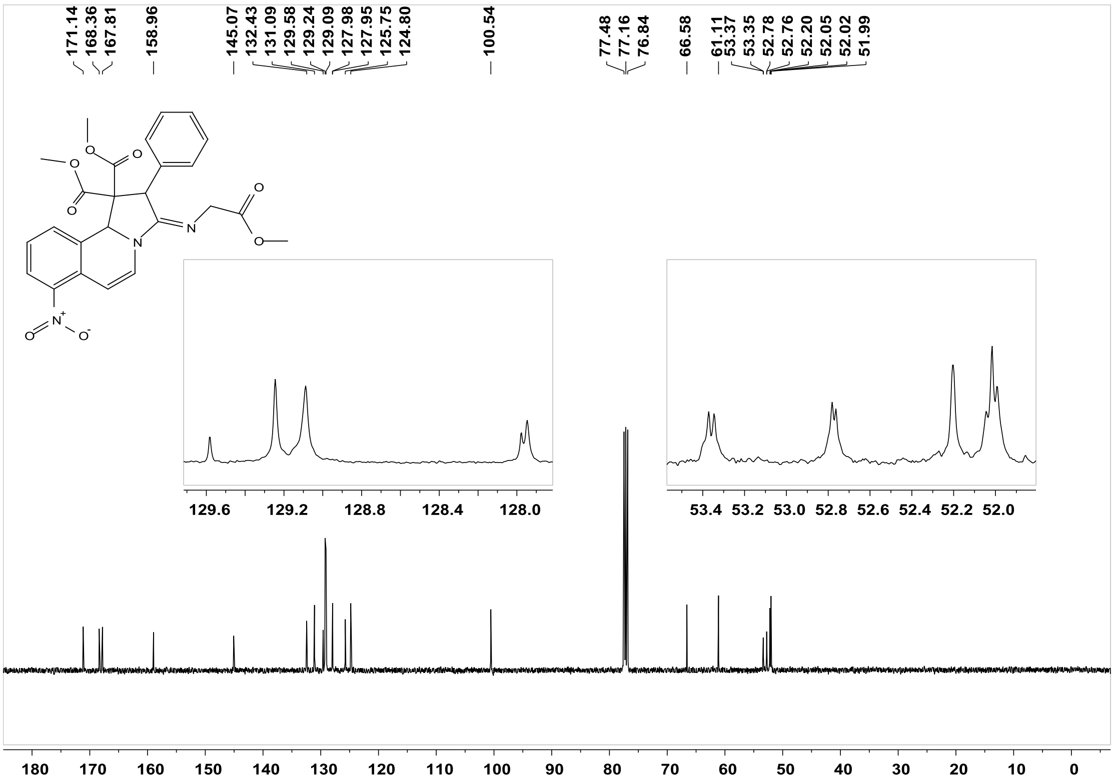

Supplementary Figure 166. ^{13}C NMR spectra for product **8s**

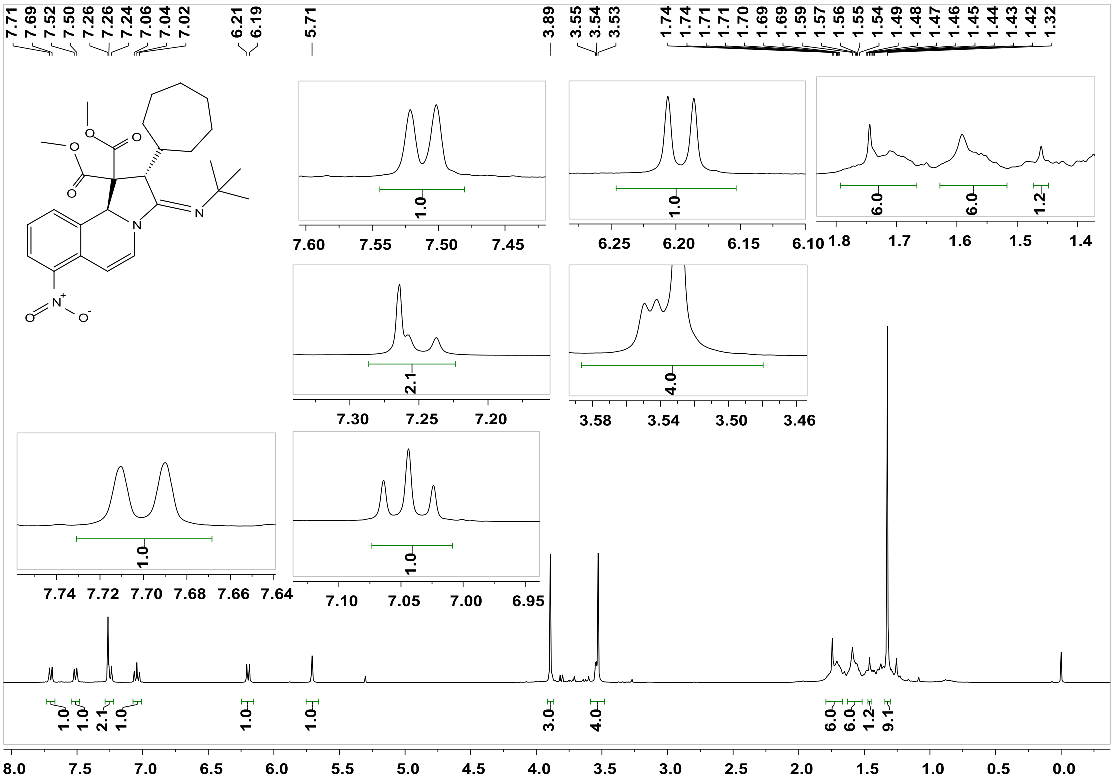

Supplementary Figure 167. ^{1}H NMR spectra for product **8t**

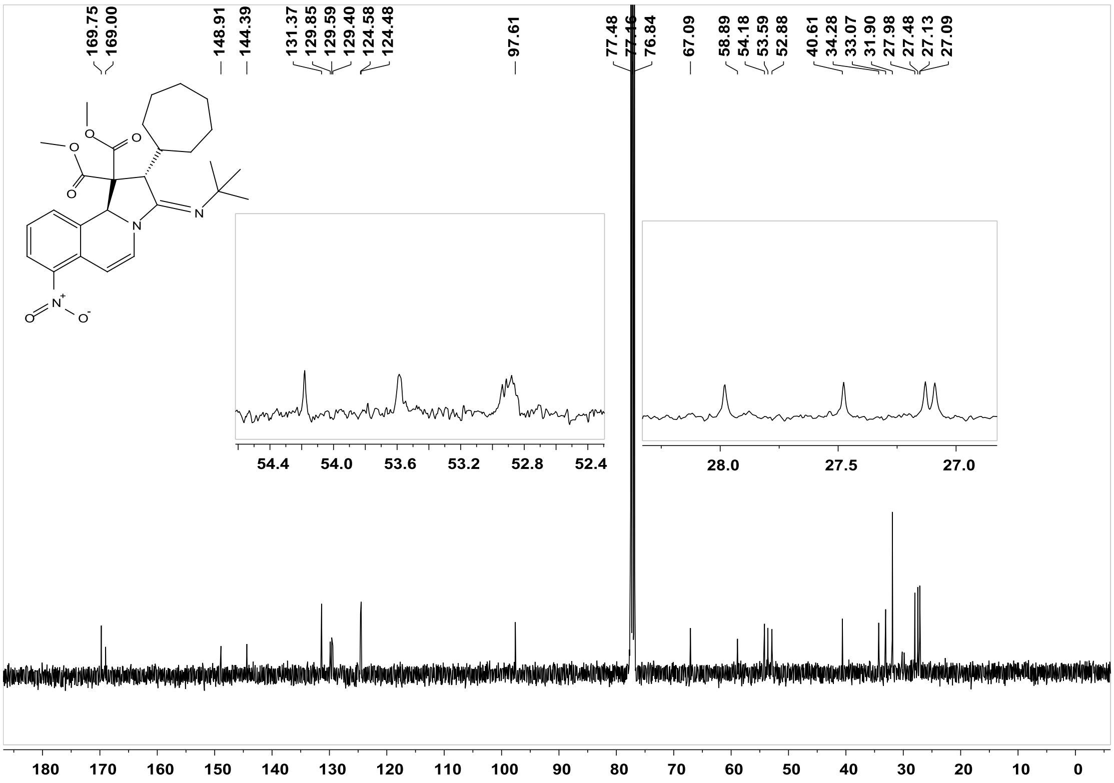

Supplementary Figure 168. ^{13}C NMR spectra for product **8t**

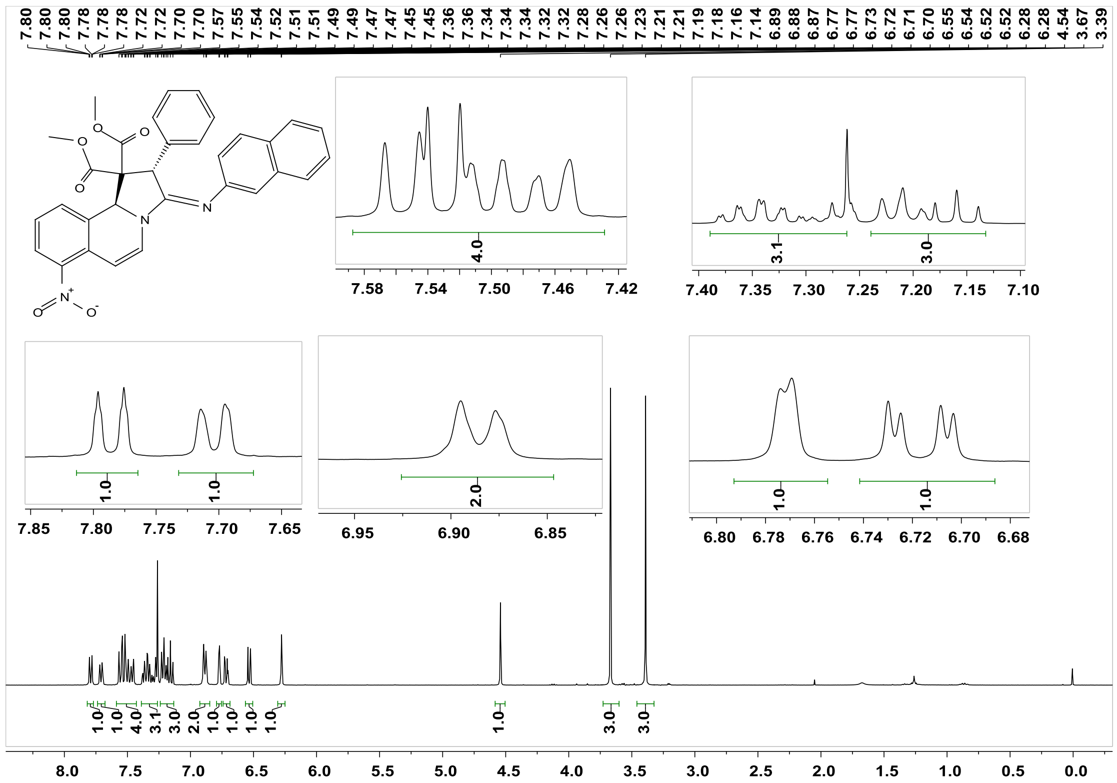

Supplementary Figure 169. ^{1}H NMR spectra for product **8u**

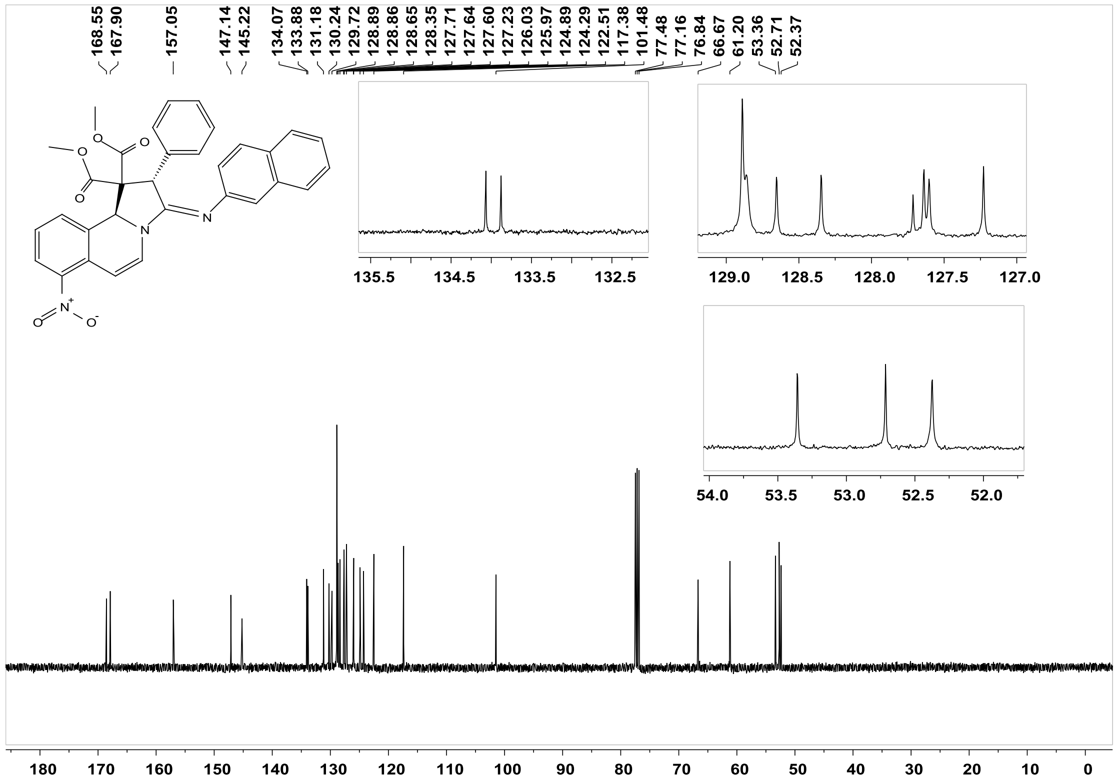

Supplementary Figure 170. ^{13}C NMR spectra for product **8u**

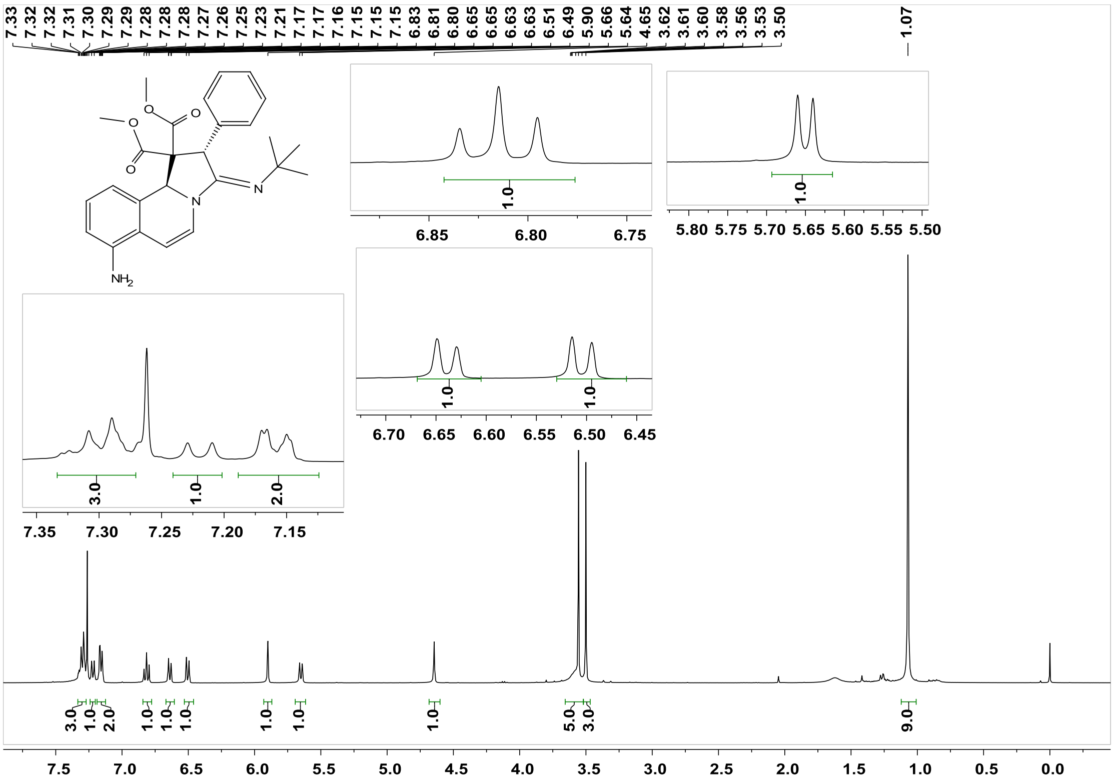

Supplementary Figure 171. ^{1}H NMR spectra for product **8v**

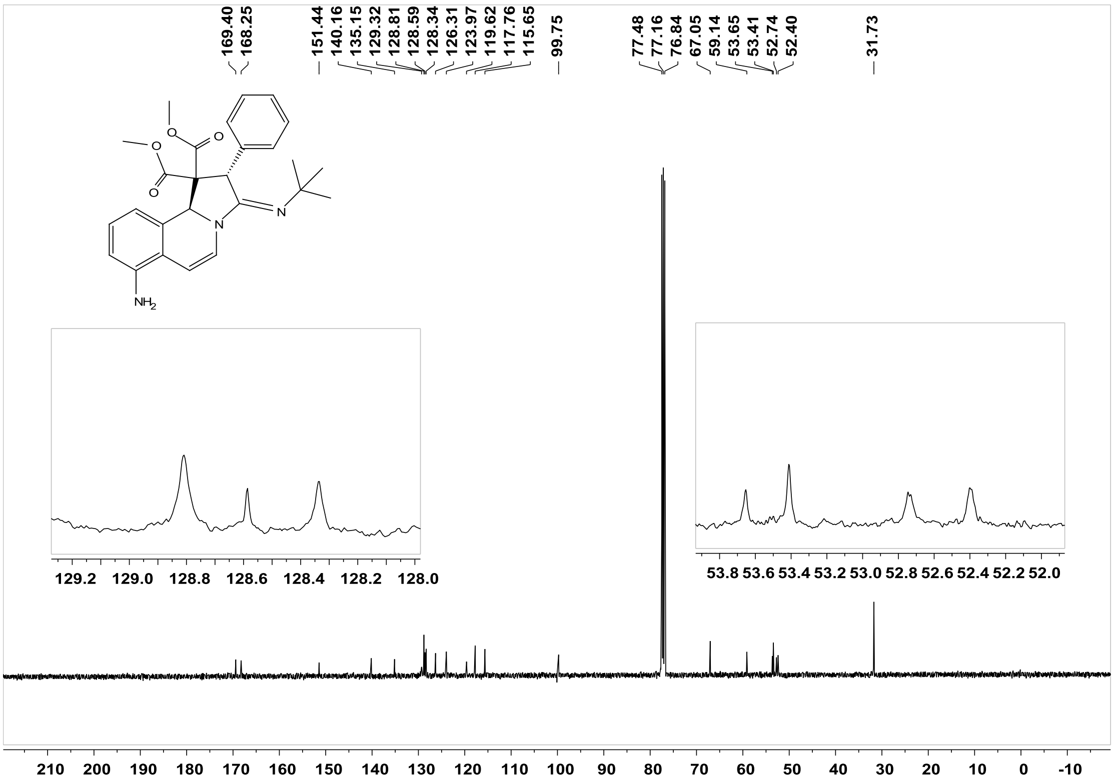

Supplementary Figure 172. ^{13}C NMR spectra for product **8v**

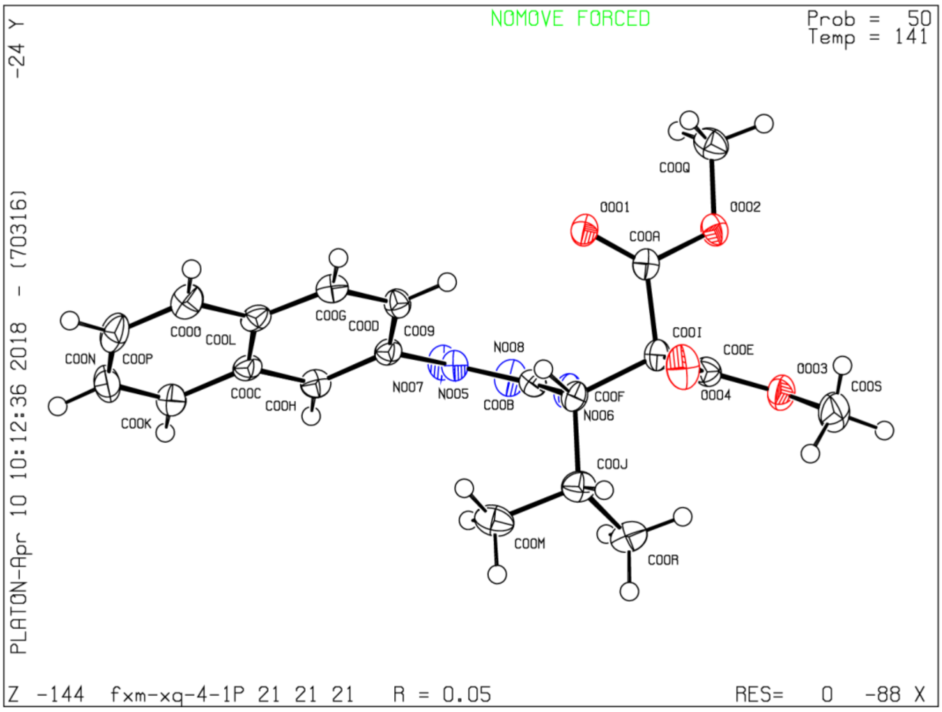

Supplementary Figure 173. ORTEP drawing of **4i**

Supplementary Table 12. Crystal date and structure refinement for **4i**

Empirical formula	$C_{20}H_{22}N_4O_4$
Formula weight	382.41
Temperature/K	141.00(10)
Crystal system	orthorhombic
Space group	$P2_12_12_1$
a/Å	8.5315(2)
b/Å	8.95968(18)
c/Å	24.5736(6)
α/°	90
β/°	90
γ/°	90
Volume/Å^3	1878.39(8)
Z	4
ρ_{calc}g/cm^3	1.352
μ/mm^{-1}	0.792
F(000)	808.0
Crystal size/mm^3	0.6 × 0.3 × 0.2
Radiation	CuKα (λ = 1.54184)
2Θ range for data collection/°	7.194 to 130.094
Index ranges	$-10 \leq h \leq 8$, $-7 \leq k \leq 10$, $-28 \leq l \leq 24$
Reflections collected	9525
Independent reflections	3199 [R_{int} = 0.0414, R_{sigma} = 0.0400]

Data/restraints/parameters	3199/0/257
Goodness-of-fit on F^2	1.031
Final R indexes [I>=2σ (I)]	R_1 = 0.0492, wR_2 = 0.1262
Final R indexes [all data]	R_1 = 0.0542, wR_2 = 0.1309
Largest diff. peak/hole / e $Å^{-3}$	0.21/-0.27
Flack parameter	0.04(16)

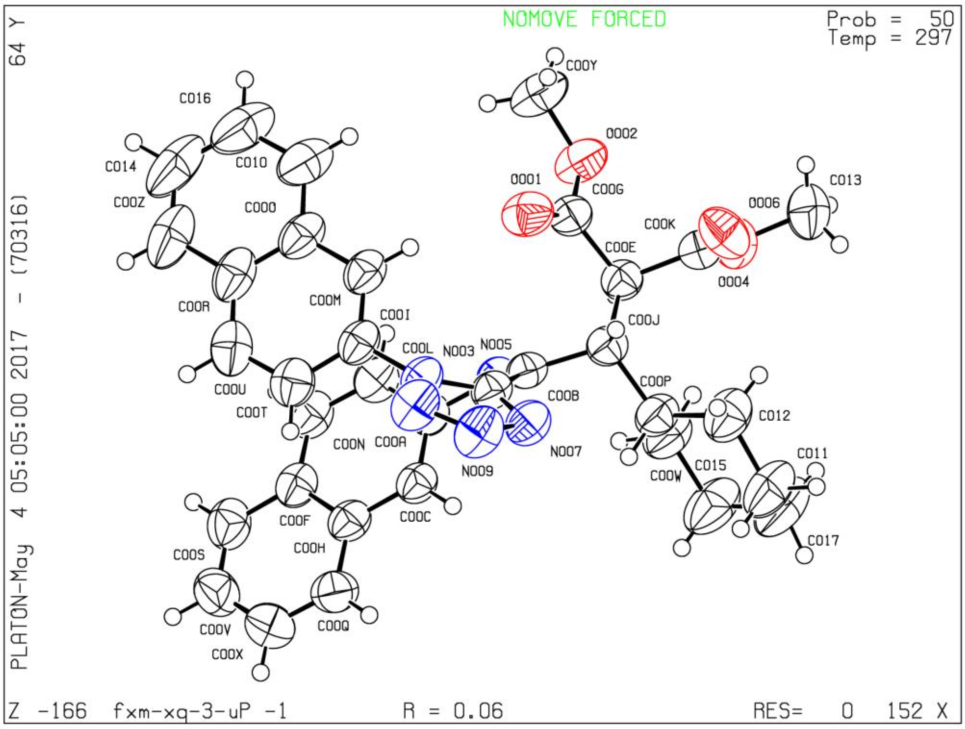

Supplementary Figure 174. ORTEP drawing of **5a**

Supplementary Table 13. Crystal date and structure refinement for **5a**

Identification code	fxm-xq-3-up
Empirical formula	$C_{34}H_{33}N_5O_4$
Formula weight	575.65
Temperature/K	296.6(5)
Crystal system	triclinic
Space group	P-1
a/Å	10.2260(4)
b/Å	12.3951(5)
c/Å	13.4645(6)
α/°	65.391(4)
β/°	77.344(4)
γ/°	80.504(3)
Volume/$Å^3$	1508.69(12)
Z	2
$\rho_{calc}g/cm^3$	1.267

μ/mm^{-1}	0.684
F(000)	608.0
Crystal size/mm^3	0.6 × 0.4 × 0.15
Radiation	CuKα (λ = 1.54184)
2Θ range for data collection/°	11.386 to 145.288
Index ranges	$-12 \le h \le 12$, $-12 \le k \le 15$, $-13 \le l \le 16$
Reflections collected	16498
Independent reflections	5850 [R_{int} = 0.0256, R_{sigma} = 0.0213]
Data/restraints/parameters	5850/0/390
Goodness-of-fit on F^2	1.038
Final R indexes [I>=2σ (I)]	R_1 = 0.0637, wR_2 = 0.1893
Final R indexes [all data]	R_1 = 0.0748, wR_2 = 0.2049
Largest diff. peak/hole / e Å^{-3}	0.53/-0.23

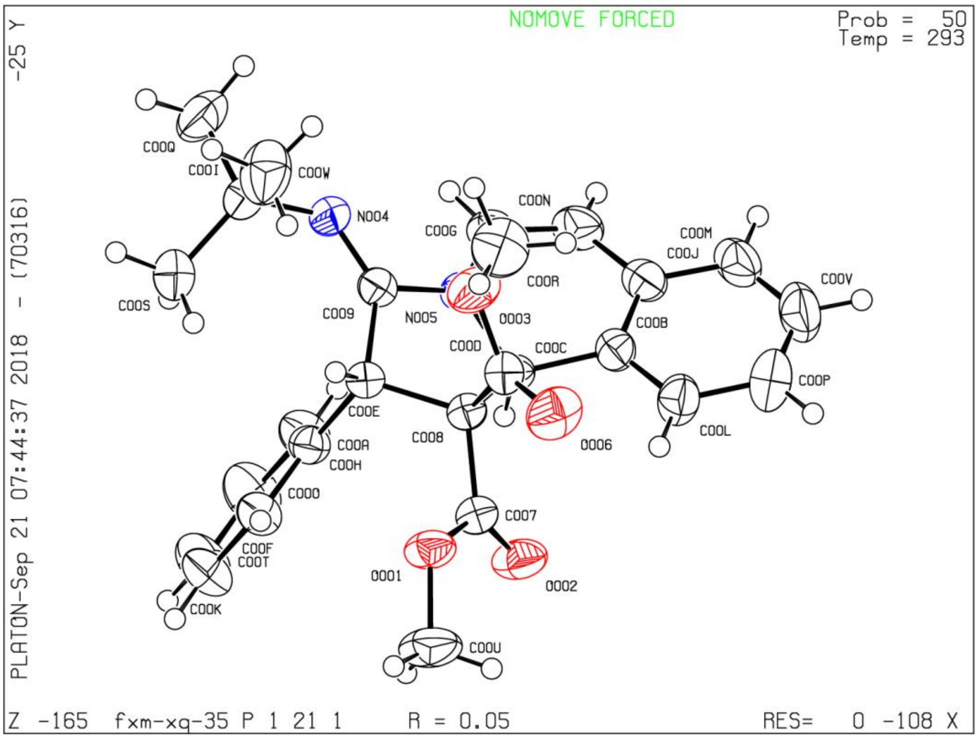

Supplementary Figure 175. ORTEP drawing of **8a**

Supplementary Table 14. Crystal date and structure refinement for **8a**

Identification code	fxm-xq-35
Empirical formula	$C_{26}H_{28}N_2O_4$
Formula weight	432.50
Temperature/K	293.01(10)
Crystal system	monoclinic
Space group	$P2_1$
a/Å	9.6898(5)
b/Å	8.0279(5)

c/Å	14.9828(8)
α/°	90
β/°	97.027(5)
γ/°	90
Volume/$Å^3$	1156.75(12)
Z	2
$\rho_{calc}g/cm^3$	1.242
μ/mm^{-1}	0.677
F(000)	460.0
Crystal size/mm^3	0.7 × 0.4 × 0.2
Radiation	CuKα (λ = 1.54184)
2Θ range for data collection/°	9.196 to 145.778
Index ranges	$-11 \le h \le 11$, $-8 \le k \le 9$, $-16 \le l \le 18$
Reflections collected	11806
Independent reflections	4045 [R_{int} = 0.0326, R_{sigma} = 0.0291]
Data/restraints/parameters	4045/1/294
Goodness-of-fit on F^2	1.056
Final R indexes [I>=2σ (I)]	R_1 = 0.0516, wR_2 = 0.1294
Final R indexes [all data]	R_1 = 0.0531, wR_2 = 0.1326
Largest diff. peak/hole / e $Å^{-3}$	0.19/-0.32
Flack parameter	0.01(13)

Supplementary References

1. Wen, Y. H., Huang, X., Huang, J. L., Xiong, Y., B. Qin & Feng, X. M. Asymmetric cyanosilylation of aldehydes catalyzed by novel organocatalysts. *Synlett*. 2445–2448 (2005).
2. Yu, Z. P., Liu, X. H., Dong, Z. H., Xie, M. S. & Feng, X. M. An *N,N'*-dioxide/In(OTf)$_3$ catalyst for the asymmetric hetero-Diels–Alder reaction between danishefsky's dienes and aldehydes: application in the total synthesis of Triketide. *Angew. Chem. Int. Ed*. **47**, 1308–1311 (2008).
3. Wang, J., Zhou, Y., Zhang, L., Li, Z., Chen, X. & Liu, H. Asymmetric Michael addition of *N*-tert-butanesulfinyl imidate with *α,β*-unsaturated diesters: scope and application to the synthesis of indanone derivatives. *Org. Lett.* **15**, 1508–1511 (2013).
4. Guan, Y., Attard, J. W., Visco, M. D., Fisher, T. J. & Mattson, A. E. Enantioselective catalyst systems from Copper(II) triflate and BINOL–Silanediol. *Chem. Eur. J.* **24**, 7123 – 7127 (2018).
5. Hu, D. X., Grice, P. & Ley, T. V. Rotamers or diastereomers? An overlooked NMR solution. *J. Org. Chem.* **77**, 5198-5202 (2012).
6. Alix, A., Lalli, C., Retailleau, P. & Masson, G. Highly enantioselective electrophilic *α*-bromination of enecarbamates: chiral phosphoric acid and calcium phosphate salt catalysts. *J. Am. Chem. Soc.* **134**, 10389-10392 (2012).
